# Supplementary material for: Reconstructing the history of founder events using genome-wide patterns of allele sharing across individuals
Source: PLoS Genet. 2022 Jun 23;18(6):e1010243. doi: 10.1371/journal.pgen.1010243 (PMC9223333; doi:10.1371/journal.pgen.1010243)
Supplement: S1 Fig — The X-axis represents the genetic distance (in cM) and the Y-axis represents the average allele sharing correlation (or weighted allele sharing covariance for populations with pseudo-haploid data, cf. S1 Table). The legend shows the mean and 95% confidence interval for the founder age (Tf) in generations before present (gBP) and the founder intensity (If), as well as the NRMSD (see Methods). The panels are grayed when the exponential fitting failed or when the evidence for the founder event was not significant (see Methods). The specific reason is highlighted in red in the legend. (PDF) [file pgen.1010243.s001.pdf]

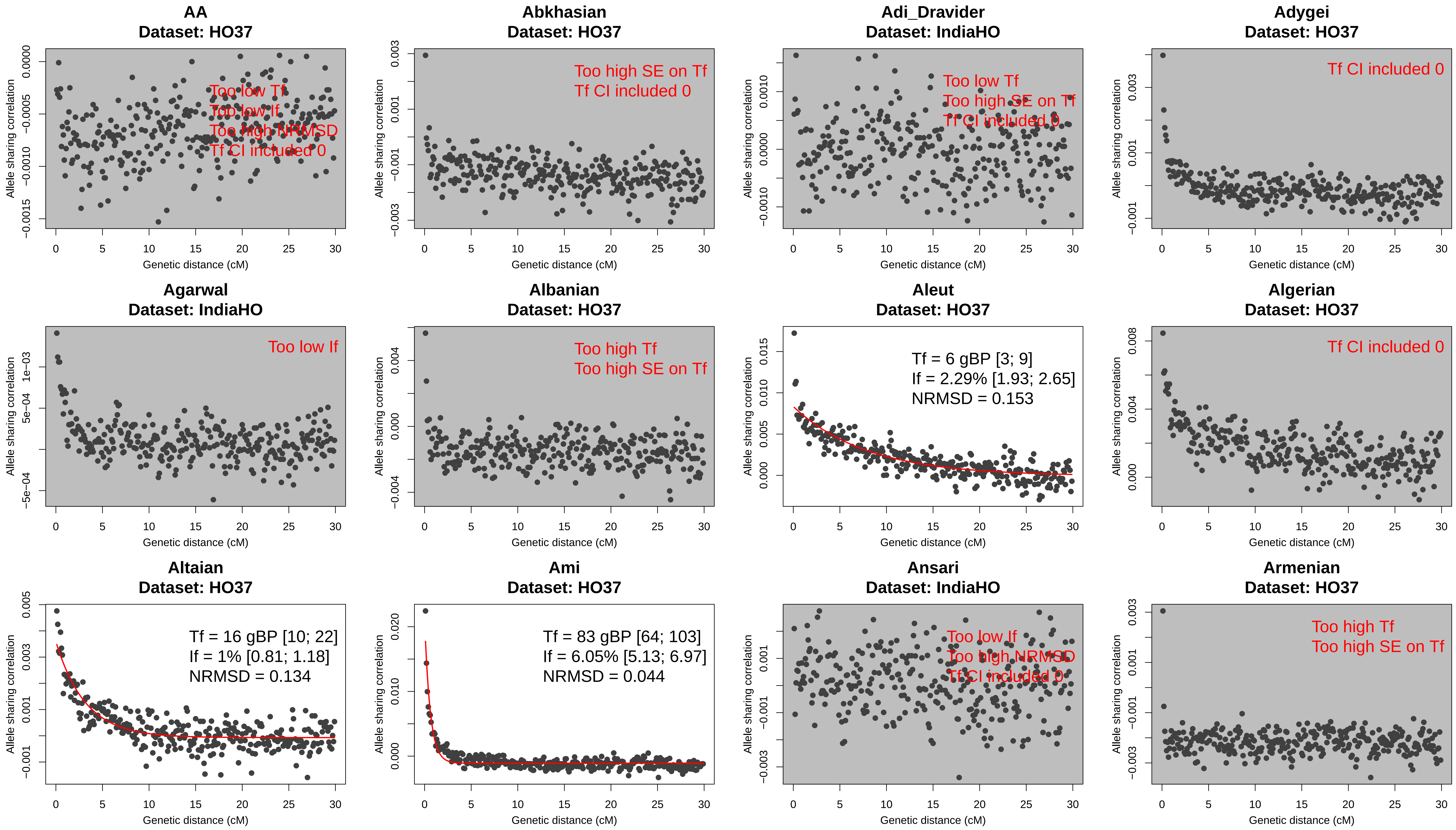
AA
Dataset: HO37
Too low Tf
Too low If
Too high NRMSD
Tf CI included 0
Abkhasian
Dataset: HO37
Too high SE on Tf
Tf CI included 0
Adi_Dravider
Dataset: IndiaHO
Too low Tf
Too high SE on Tf
Tf CI included 0
Adygei
Dataset: HO37
Tf CI included 0
Agarwal
Dataset: IndiaHO
Too low If
Albanian
Dataset: HO37
Too high Tf
Too high SE on Tf
Aleut
Dataset: HO37
Tf = 6 gBP [3; 9]
If = 2.29% [1.93; 2.65]
NRMSD = 0.153
Algerian
Dataset: HO37
Tf CI included 0
Altaian
Dataset: HO37
Tf = 16 gBP [10; 22]
If = 1% [0.81; 1.18]
NRMSD = 0.134
Ami
Dataset: HO37
Tf = 83 gBP [64; 103]
If = 6.05% [5.13; 6.97]
NRMSD = 0.044
Ansari
Dataset: IndiaHO
Too low If
Too high NRMSD
Tf CI included 0
Armenian
Dataset: HO37
Too high Tf
Too high SE on Tf
Allele sharing correlation
Genetic distance (cM)

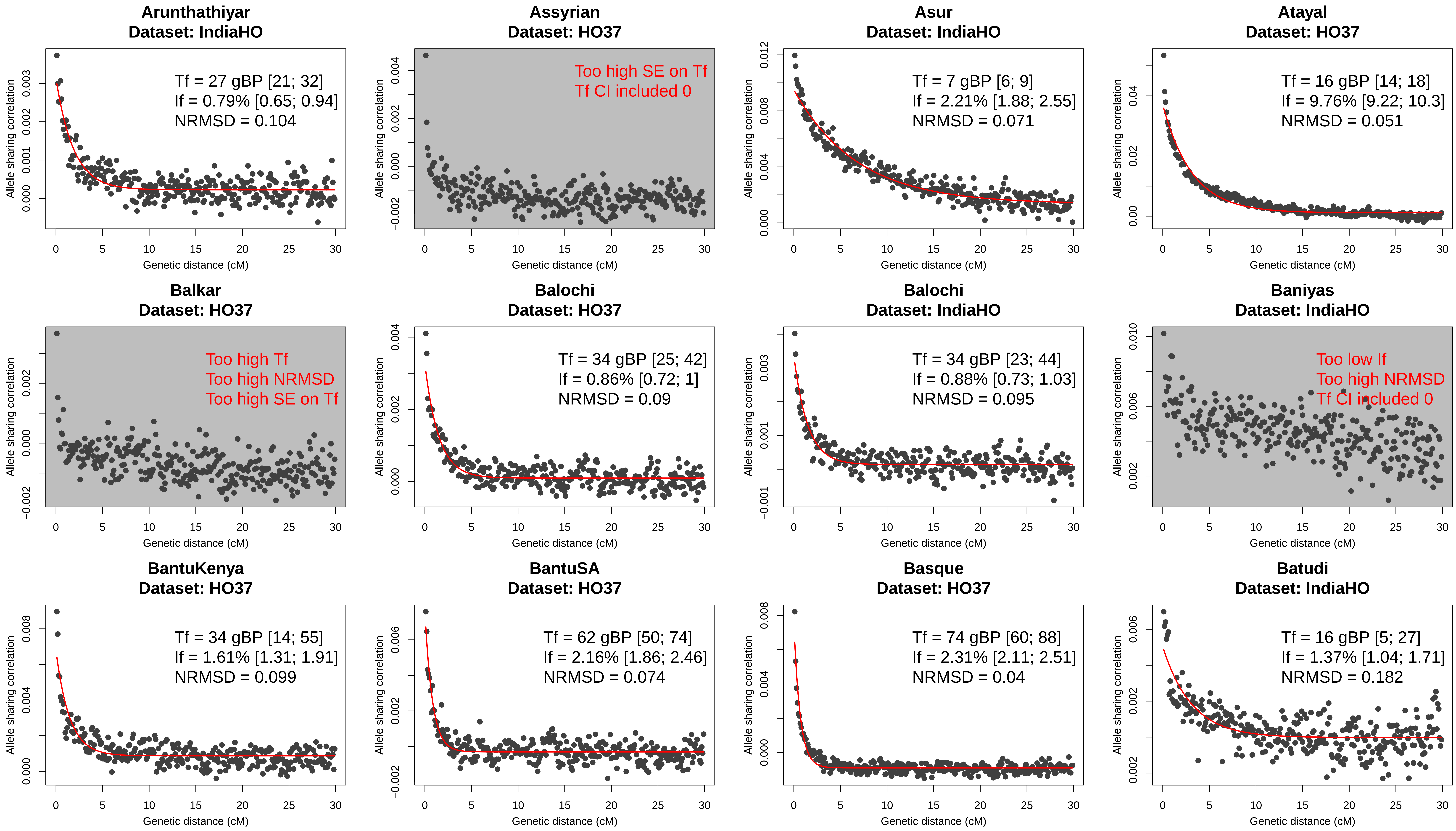
Arunthathiyar
Dataset: IndiaHO
Tf = 27 gBP [21; 32]
If = 0.79% [0.65; 0.94]
NRMSD = 0.104
Assyrian
Dataset: HO37
Too high SE on Tf
Tf CI included 0
Asur
Dataset: IndiaHO
Tf = 7 gBP [6; 9]
If = 2.21% [1.88; 2.55]
NRMSD = 0.071
Atayal
Dataset: HO37
Tf = 16 gBP [14; 18]
If = 9.76% [9.22; 10.3]
NRMSD = 0.051
Balkar
Dataset: HO37
Too high Tf
Too high NRMSD
Too high SE on Tf
Balochi
Dataset: HO37
Tf = 34 gBP [25; 42]
If = 0.86% [0.72; 1]
NRMSD = 0.09
Balochi
Dataset: IndiaHO
Tf = 34 gBP [23; 44]
If = 0.88% [0.73; 1.03]
NRMSD = 0.095
Baniyas
Dataset: IndiaHO
Too low If
Too high NRMSD
Tf CI included 0
BantuKenya
Dataset: HO37
Tf = 34 gBP [14; 55]
If = 1.61% [1.31; 1.91]
NRMSD = 0.099
BantuSA
Dataset: HO37
Tf = 62 gBP [50; 74]
If = 2.16% [1.86; 2.46]
NRMSD = 0.074
Basque
Dataset: HO37
Tf = 74 gBP [60; 88]
If = 2.31% [2.11; 2.51]
NRMSD = 0.04
Batudi
Dataset: IndiaHO
Tf = 16 gBP [5; 27]
If = 1.37% [1.04; 1.71]
NRMSD = 0.182
Allele sharing correlation
Genetic distance (cM)

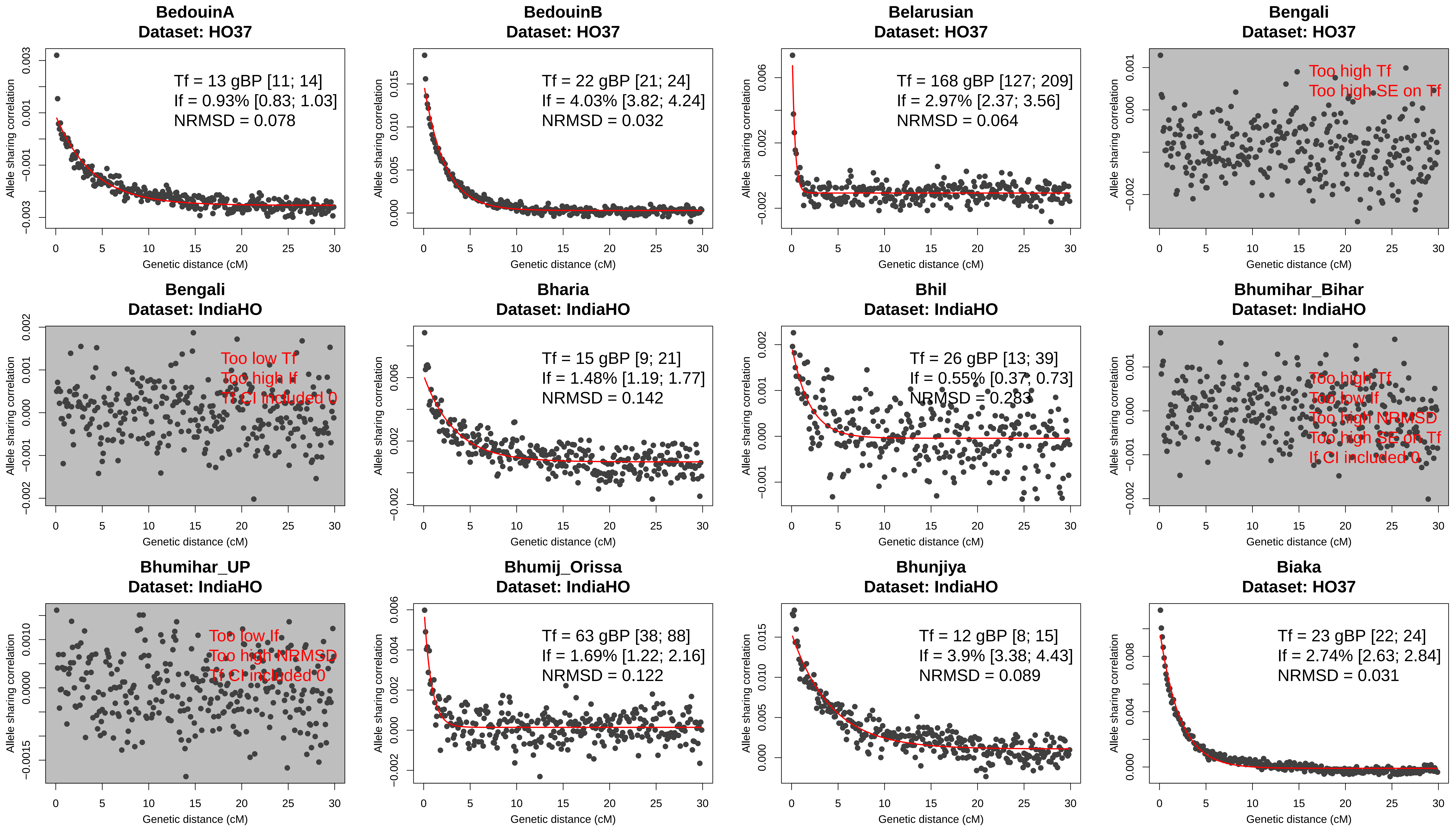
BedouinA
Dataset: HO37
Tf = 13 gBP [11; 14]
If = 0.93% [0.83; 1.03]
NRMSD = 0.078
BedouinB
Dataset: HO37
Tf = 22 gBP [21; 24]
If = 4.03% [3.82; 4.24]
NRMSD = 0.032
Belarusian
Dataset: HO37
Tf = 168 gBP [127; 209]
If = 2.97% [2.37; 3.56]
NRMSD = 0.064
Bengali
Dataset: HO37
Too high Tf
Too high SE on Tf
Bengali
Dataset: IndiaHO
Too low Tf
Too high If
Tf CI included 0
Bharia
Dataset: IndiaHO
Tf = 15 gBP [9; 21]
If = 1.48% [1.19; 1.77]
NRMSD = 0.142
Bhil
Dataset: IndiaHO
Tf = 26 gBP [13; 39]
If = 0.55% [0.37; 0.73]
NRMSD = 0.283
Bhumihar_Bihar
Dataset: IndiaHO
Too high Tf
Too low If
Too high NRMSD
Too high SE on Tf
If CI included 0
Bhumihar_UP
Dataset: IndiaHO
Too low If
Too high NRMSD
Tf CI included 0
Bhumij_Orissa
Dataset: IndiaHO
Tf = 63 gBP [38; 88]
If = 1.69% [1.22; 2.16]
NRMSD = 0.122
Bhunjiya
Dataset: IndiaHO
Tf = 12 gBP [8; 15]
If = 3.9% [3.38; 4.43]
NRMSD = 0.089
Biaka
Dataset: HO37
Tf = 23 gBP [22; 24]
If = 2.74% [2.63; 2.84]
NRMSD = 0.031
Allele sharing correlation
Genetic distance (cM)

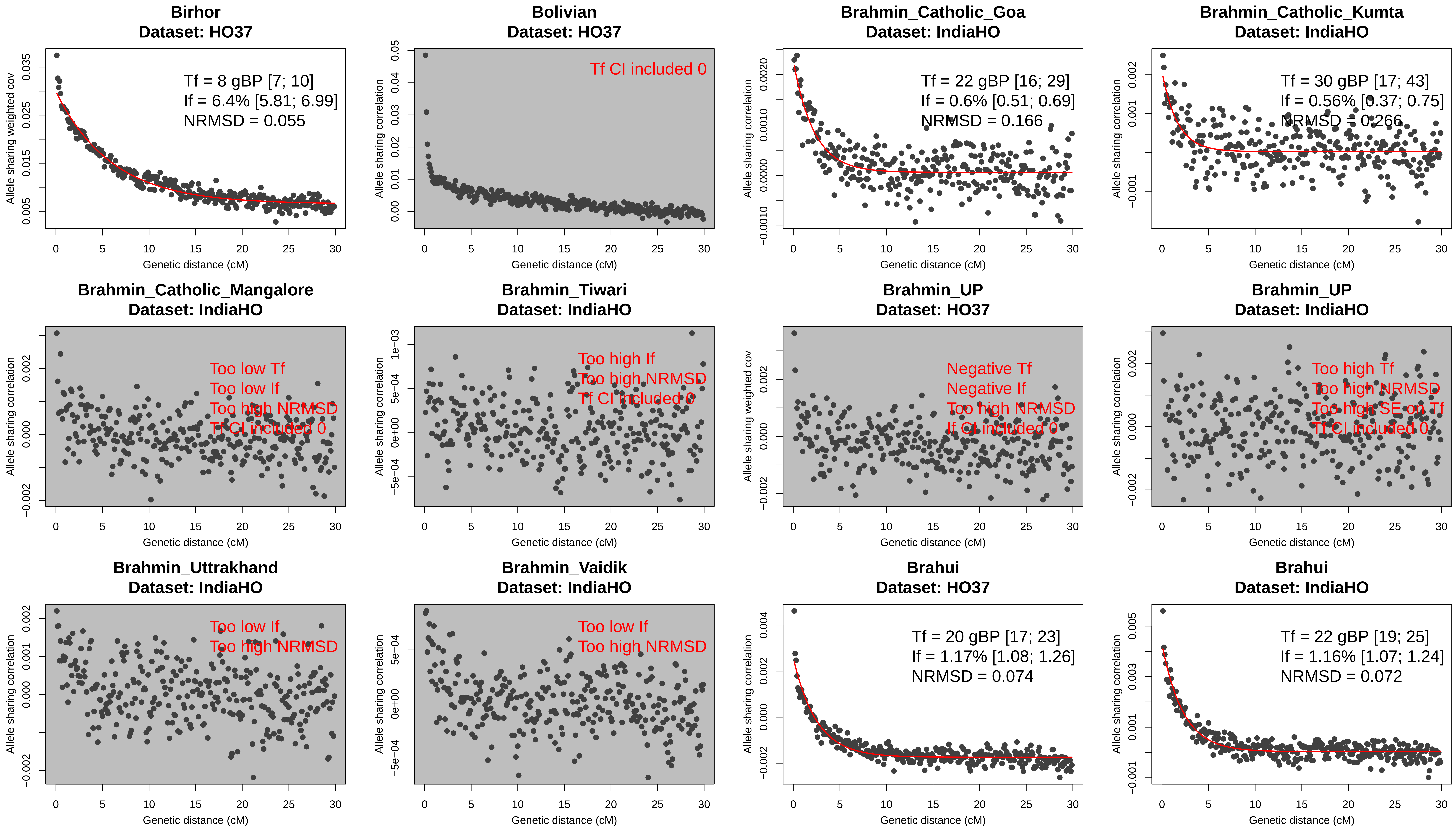
Birhor
Dataset: HO37
Tf = 8 gBP [7; 10]
If = 6.4% [5.81; 6.99]
NRMSD = 0.055
Allele sharing weighted cov
Genetic distance (cM)
Bolivian
Dataset: HO37
Tf CI included 0
Allele sharing correlation
Genetic distance (cM)
Brahmin_Catholic_Goa
Dataset: IndiaHO
Tf = 22 gBP [16; 29]
If = 0.6% [0.51; 0.69]
NRMSD = 0.166
Allele sharing correlation
Genetic distance (cM)
Brahmin_Catholic_Kumta
Dataset: IndiaHO
Tf = 30 gBP [17; 43]
If = 0.56% [0.37; 0.75]
NRMSD = 0.266
Allele sharing correlation
Genetic distance (cM)
Brahmin_Catholic_Mangalore
Dataset: IndiaHO
Too low Tf
Too low If
Too high NRMSD
Tf CI included 0
Allele sharing correlation
Genetic distance (cM)
Brahmin_Tiwari
Dataset: IndiaHO
Too high If
Too high NRMSD
Tf CI included 0
Allele sharing correlation
Genetic distance (cM)
Brahmin_UP
Dataset: HO37
Negative Tf
Negative If
Too high NRMSD
If CI included 0
Allele sharing weighted cov
Genetic distance (cM)
Brahmin_UP
Dataset: IndiaHO
Too high Tf
Too high NRMSD
Too high SE on Tf
Tf CI included 0
Allele sharing correlation
Genetic distance (cM)
Brahmin_Uttrakhand
Dataset: IndiaHO
Too low If
Too high NRMSD
Allele sharing correlation
Genetic distance (cM)
Brahmin_Vaidik
Dataset: IndiaHO
Too low If
Too high NRMSD
Allele sharing correlation
Genetic distance (cM)
Brahui
Dataset: HO37
Tf = 20 gBP [17; 23]
If = 1.17% [1.08; 1.26]
NRMSD = 0.074
Allele sharing correlation
Genetic distance (cM)
Brahui
Dataset: IndiaHO
Tf = 22 gBP [19; 25]
If = 1.16% [1.07; 1.24]
NRMSD = 0.072
Allele sharing correlation
Genetic distance (cM)

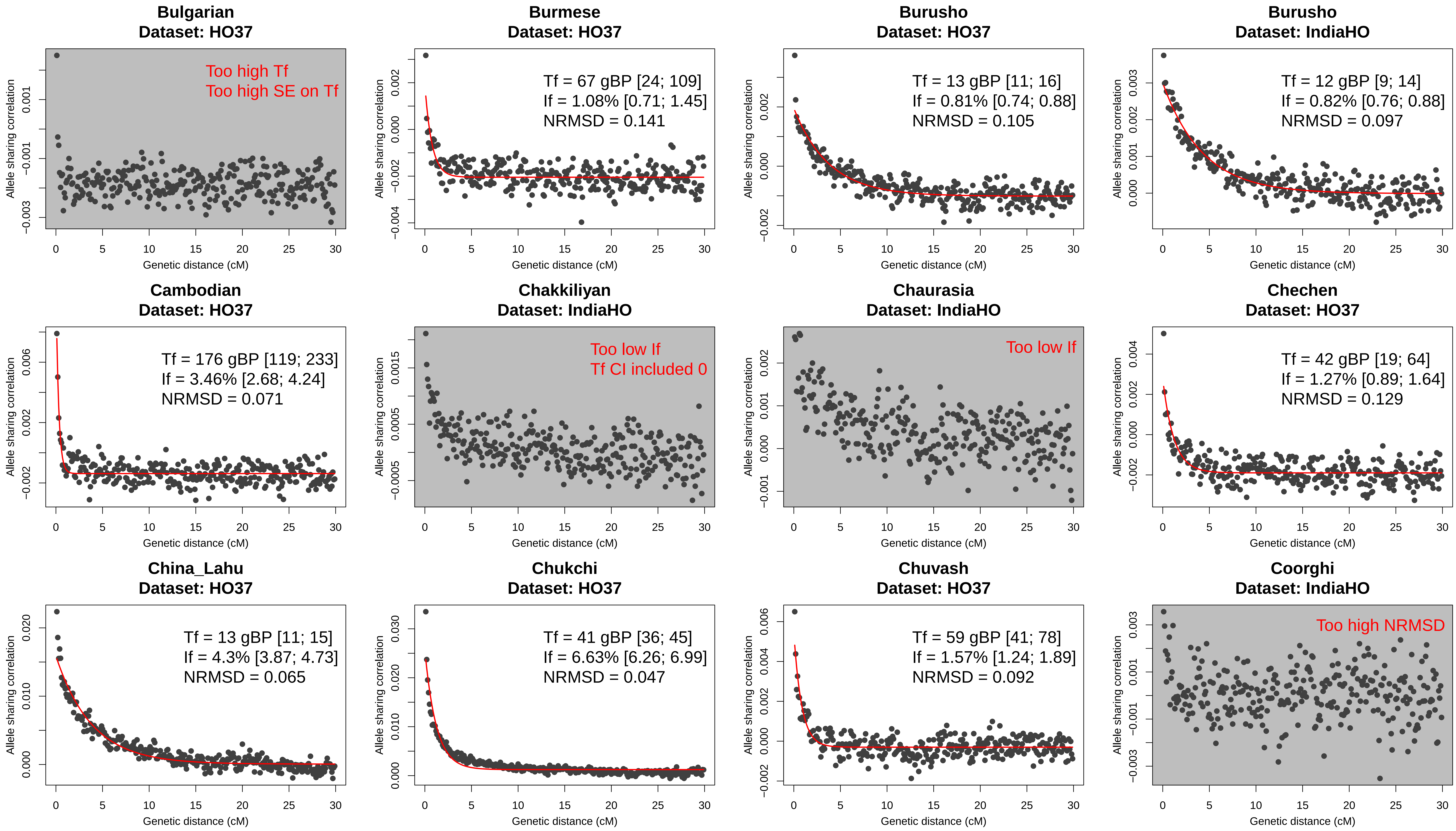
Bulgarian
Dataset: HO37
Too high Tf
Too high SE on Tf
Burmese
Dataset: HO37
Tf = 67 gBP [24; 109]
If = 1.08% [0.71; 1.45]
NRMSD = 0.141
Burusho
Dataset: HO37
Tf = 13 gBP [11; 16]
If = 0.81% [0.74; 0.88]
NRMSD = 0.105
Burusho
Dataset: IndiaHO
Tf = 12 gBP [9; 14]
If = 0.82% [0.76; 0.88]
NRMSD = 0.097
Cambodian
Dataset: HO37
Tf = 176 gBP [119; 233]
If = 3.46% [2.68; 4.24]
NRMSD = 0.071
Chakkiliyan
Dataset: IndiaHO
Too low If
Tf CI included 0
Chaurasia
Dataset: IndiaHO
Too low If
Chechen
Dataset: HO37
Tf = 42 gBP [19; 64]
If = 1.27% [0.89; 1.64]
NRMSD = 0.129
China_Lahu
Dataset: HO37
Tf = 13 gBP [11; 15]
If = 4.3% [3.87; 4.73]
NRMSD = 0.065
Chukchi
Dataset: HO37
Tf = 41 gBP [36; 45]
If = 6.63% [6.26; 6.99]
NRMSD = 0.047
Chuvash
Dataset: HO37
Tf = 59 gBP [41; 78]
If = 1.57% [1.24; 1.89]
NRMSD = 0.092
Coorghi
Dataset: IndiaHO
Too high NRMSD
Allele sharing correlation
Genetic distance (cM)

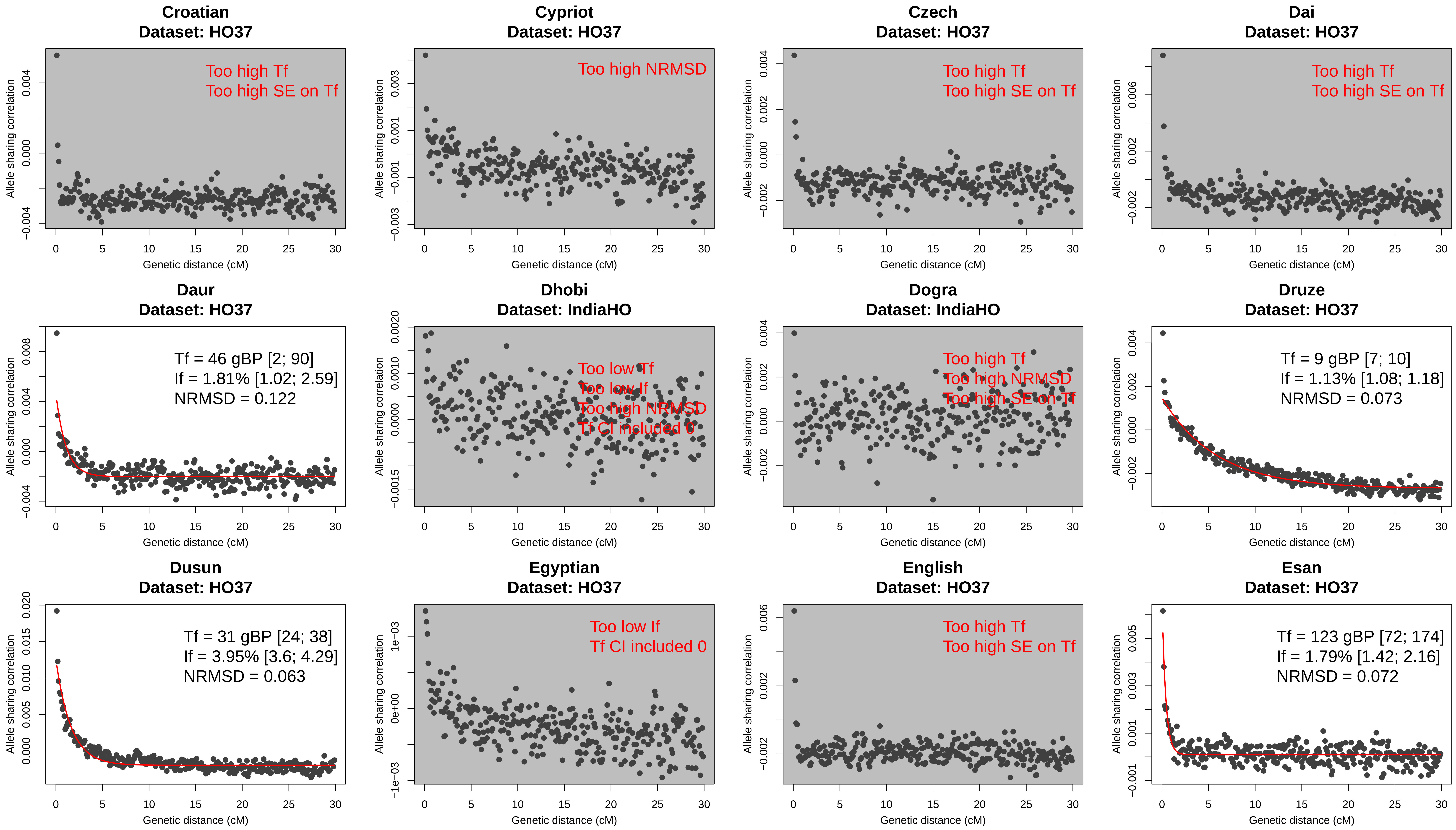
Croatian
Dataset: HO37
Too high Tf
Too high SE on Tf
Cypriot
Dataset: HO37
Too high NRMSD
Czech
Dataset: HO37
Too high Tf
Too high SE on Tf
Dai
Dataset: HO37
Too high Tf
Too high SE on Tf
Daur
Dataset: HO37
Tf = 46 gBP [2; 90]
If = 1.81% [1.02; 2.59]
NRMSD = 0.122
Dhobi
Dataset: IndiaHO
Too low Tf
Too low If
Too high NRMSD
Tf CI included 0
Dogra
Dataset: IndiaHO
Too high Tf
Too high NRMSD
Too high SE on Tf
Druze
Dataset: HO37
Tf = 9 gBP [7; 10]
If = 1.13% [1.08; 1.18]
NRMSD = 0.073
Dusun
Dataset: HO37
Tf = 31 gBP [24; 38]
If = 3.95% [3.6; 4.29]
NRMSD = 0.063
Egyptian
Dataset: HO37
Too low If
Tf CI included 0
English
Dataset: HO37
Too high Tf
Too high SE on Tf
Esan
Dataset: HO37
Tf = 123 gBP [72; 174]
If = 1.79% [1.42; 2.16]
NRMSD = 0.072
Allele sharing correlation
Genetic distance (cM)

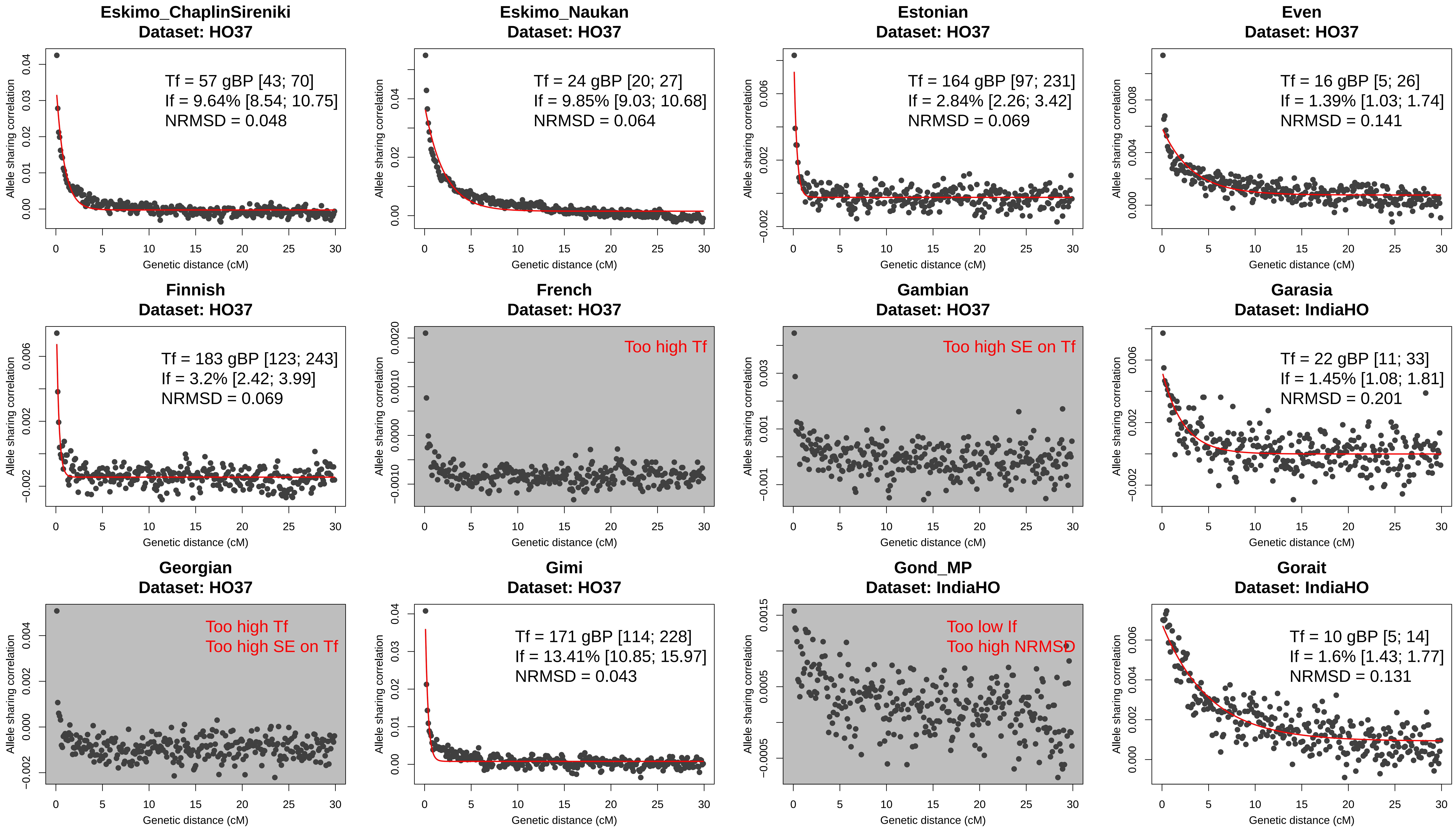
Eskimo_ChaplinSireniki
Dataset: HO37
Tf = 57 gBP [43; 70]
If = 9.64% [8.54; 10.75]
NRMSD = 0.048
Eskimo_Naukan
Dataset: HO37
Tf = 24 gBP [20; 27]
If = 9.85% [9.03; 10.68]
NRMSD = 0.064
Estonian
Dataset: HO37
Tf = 164 gBP [97; 231]
If = 2.84% [2.26; 3.42]
NRMSD = 0.069
Even
Dataset: HO37
Tf = 16 gBP [5; 26]
If = 1.39% [1.03; 1.74]
NRMSD = 0.141
Finnish
Dataset: HO37
Tf = 183 gBP [123; 243]
If = 3.2% [2.42; 3.99]
NRMSD = 0.069
French
Dataset: HO37
Too high Tf
Gambian
Dataset: HO37
Too high SE on Tf
Garasia
Dataset: IndiaHO
Tf = 22 gBP [11; 33]
If = 1.45% [1.08; 1.81]
NRMSD = 0.201
Georgian
Dataset: HO37
Too high Tf
Too high SE on Tf
Gimi
Dataset: HO37
Tf = 171 gBP [114; 228]
If = 13.41% [10.85; 15.97]
NRMSD = 0.043
Gond_MP
Dataset: IndiaHO
Too low If
Too high NRMSD
Gorait
Dataset: IndiaHO
Tf = 10 gBP [5; 14]
If = 1.6% [1.43; 1.77]
NRMSD = 0.131
Allele sharing correlation
Genetic distance (cM)

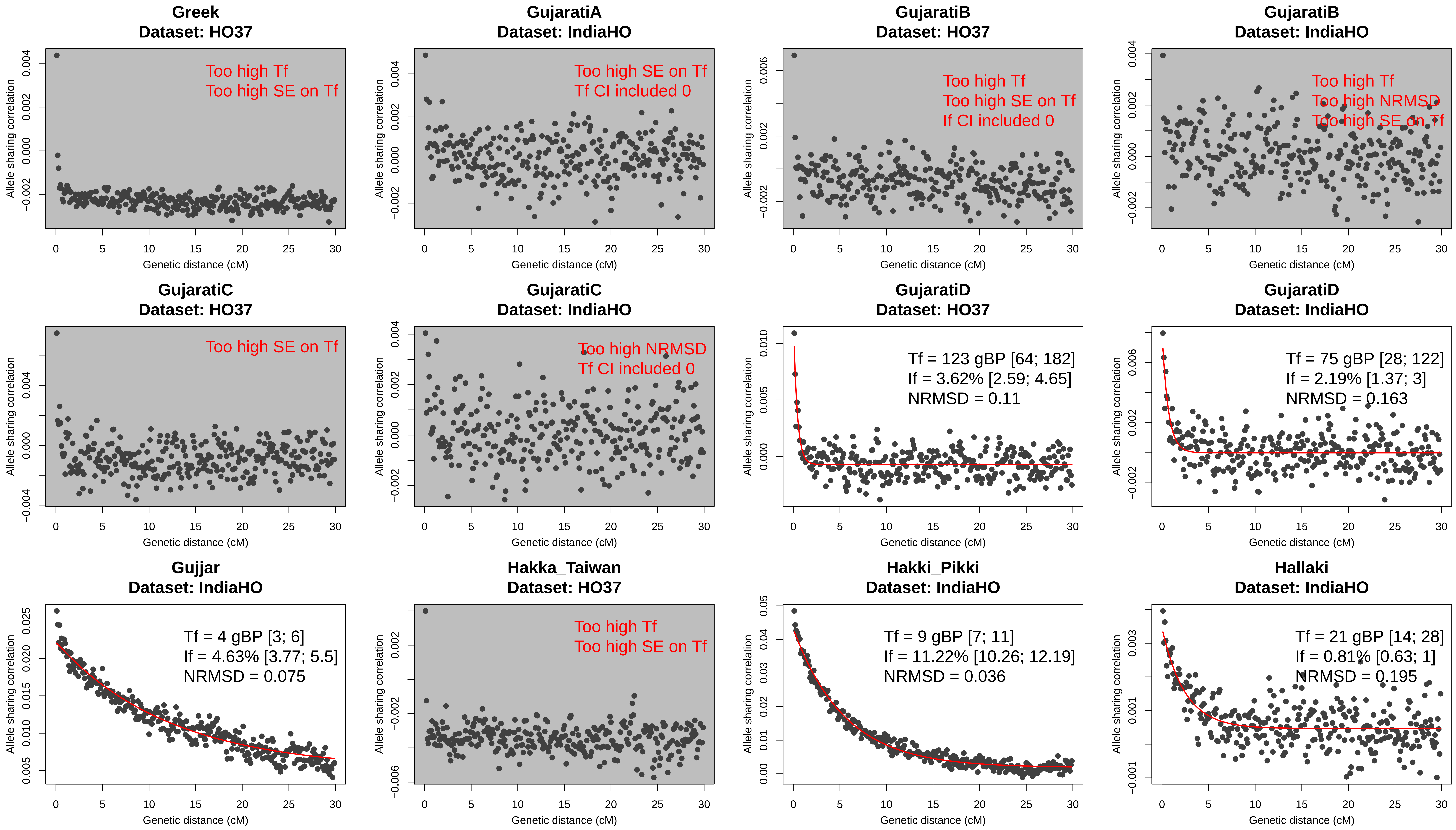
Greek
Dataset: HO37
Too high Tf
Too high SE on Tf
GujaratiA
Dataset: IndiaHO
Too high SE on Tf
Tf CI included 0
GujaratiB
Dataset: HO37
Too high Tf
Too high SE on Tf
If CI included 0
GujaratiB
Dataset: IndiaHO
Too high Tf
Too high NRMSD
Too high SE on Tf
GujaratiC
Dataset: HO37
Too high SE on Tf
GujaratiC
Dataset: IndiaHO
Too high NRMSD
Tf CI included 0
GujaratiD
Dataset: HO37
Tf = 123 gBP [64; 182]
If = 3.62% [2.59; 4.65]
NRMSD = 0.11
GujaratiD
Dataset: IndiaHO
Tf = 75 gBP [28; 122]
If = 2.19% [1.37; 3]
NRMSD = 0.163
Gujjar
Dataset: IndiaHO
Tf = 4 gBP [3; 6]
If = 4.63% [3.77; 5.5]
NRMSD = 0.075
Hakka_Taiwan
Dataset: HO37
Too high Tf
Too high SE on Tf
Hakki_Pikki
Dataset: IndiaHO
Tf = 9 gBP [7; 11]
If = 11.22% [10.26; 12.19]
NRMSD = 0.036
Hallaki
Dataset: IndiaHO
Tf = 21 gBP [14; 28]
If = 0.81% [0.63; 1]
NRMSD = 0.195
Allele sharing correlation
Genetic distance (cM)

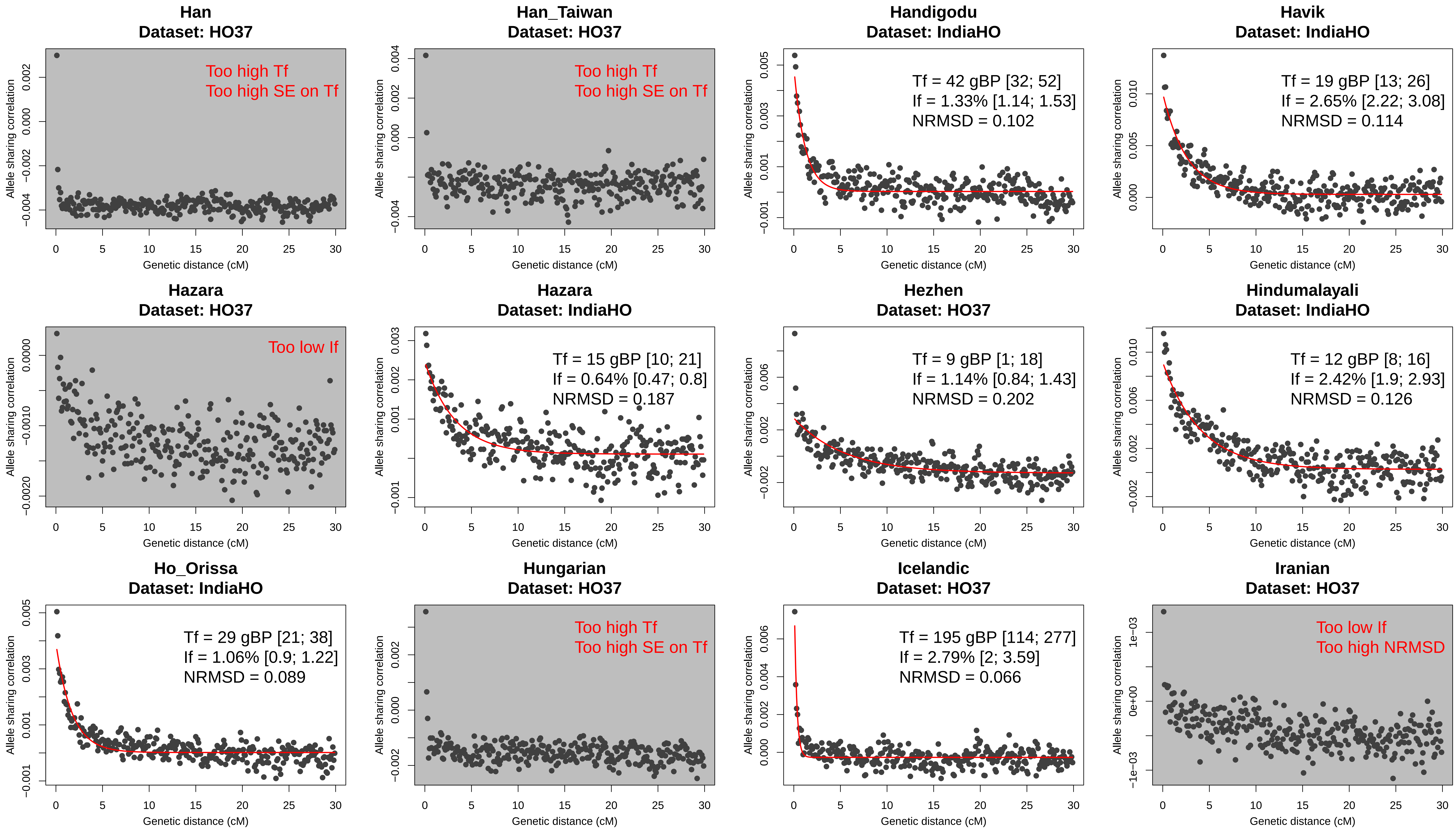
Han
Dataset: HO37
Too high Tf
Too high SE on Tf
Han_Taiwan
Dataset: HO37
Too high Tf
Too high SE on Tf
Handigodu
Dataset: IndiaHO
Tf = 42 gBP [32; 52]
If = 1.33% [1.14; 1.53]
NRMSD = 0.102
Havik
Dataset: IndiaHO
Tf = 19 gBP [13; 26]
If = 2.65% [2.22; 3.08]
NRMSD = 0.114
Hazara
Dataset: HO37
Too low If
Hazara
Dataset: IndiaHO
Tf = 15 gBP [10; 21]
If = 0.64% [0.47; 0.8]
NRMSD = 0.187
Hezhen
Dataset: HO37
Tf = 9 gBP [1; 18]
If = 1.14% [0.84; 1.43]
NRMSD = 0.202
Hindumalayali
Dataset: IndiaHO
Tf = 12 gBP [8; 16]
If = 2.42% [1.9; 2.93]
NRMSD = 0.126
Ho_Orissa
Dataset: IndiaHO
Tf = 29 gBP [21; 38]
If = 1.06% [0.9; 1.22]
NRMSD = 0.089
Hungarian
Dataset: HO37
Too high Tf
Too high SE on Tf
Icelandic
Dataset: HO37
Tf = 195 gBP [114; 277]
If = 2.79% [2; 3.59]
NRMSD = 0.066
Iranian
Dataset: HO37
Too low If
Too high NRMSD
Allele sharing correlation
Genetic distance (cM)

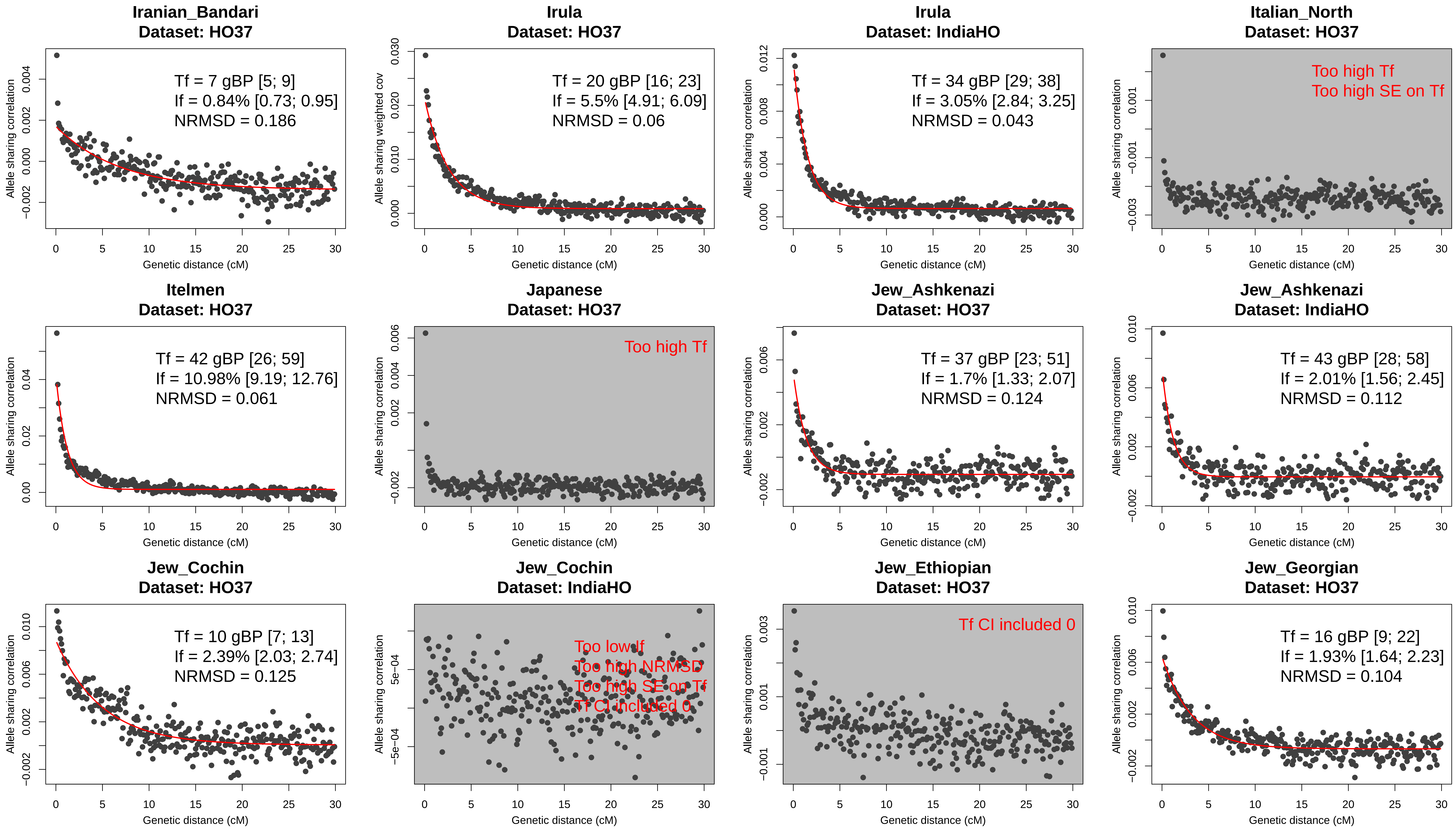

Iranian_Bandari
Dataset: HO37
Tf = 7 gBP [5; 9]
If = 0.84% [0.73; 0.95]
NRMSD = 0.186
Irula
Dataset: HO37
Tf = 20 gBP [16; 23]
If = 5.5% [4.91; 6.09]
NRMSD = 0.06
Irula
Dataset: IndiaHO
Tf = 34 gBP [29; 38]
If = 3.05% [2.84; 3.25]
NRMSD = 0.043
Italian_North
Dataset: HO37
Too high Tf
Too high SE on Tf
Itelmen
Dataset: HO37
Tf = 42 gBP [26; 59]
If = 10.98% [9.19; 12.76]
NRMSD = 0.061
Japanese
Dataset: HO37
Too high Tf
Jew_Ashkenazi
Dataset: HO37
Tf = 37 gBP [23; 51]
If = 1.7% [1.33; 2.07]
NRMSD = 0.124
Jew_Ashkenazi
Dataset: IndiaHO
Tf = 43 gBP [28; 58]
If = 2.01% [1.56; 2.45]
NRMSD = 0.112
Jew_Cochin
Dataset: HO37
Tf = 10 gBP [7; 13]
If = 2.39% [2.03; 2.74]
NRMSD = 0.125
Jew_Cochin
Dataset: IndiaHO
Too low If
Too high NRMSD
Too high SE on Tf
Tf CI included 0
Jew_Ethiopian
Dataset: HO37
Tf CI included 0
Jew_Georgian
Dataset: HO37
Tf = 16 gBP [9; 22]
If = 1.93% [1.64; 2.23]
NRMSD = 0.104
Allele sharing correlation
Allele sharing weighted cov
Genetic distance (cM)

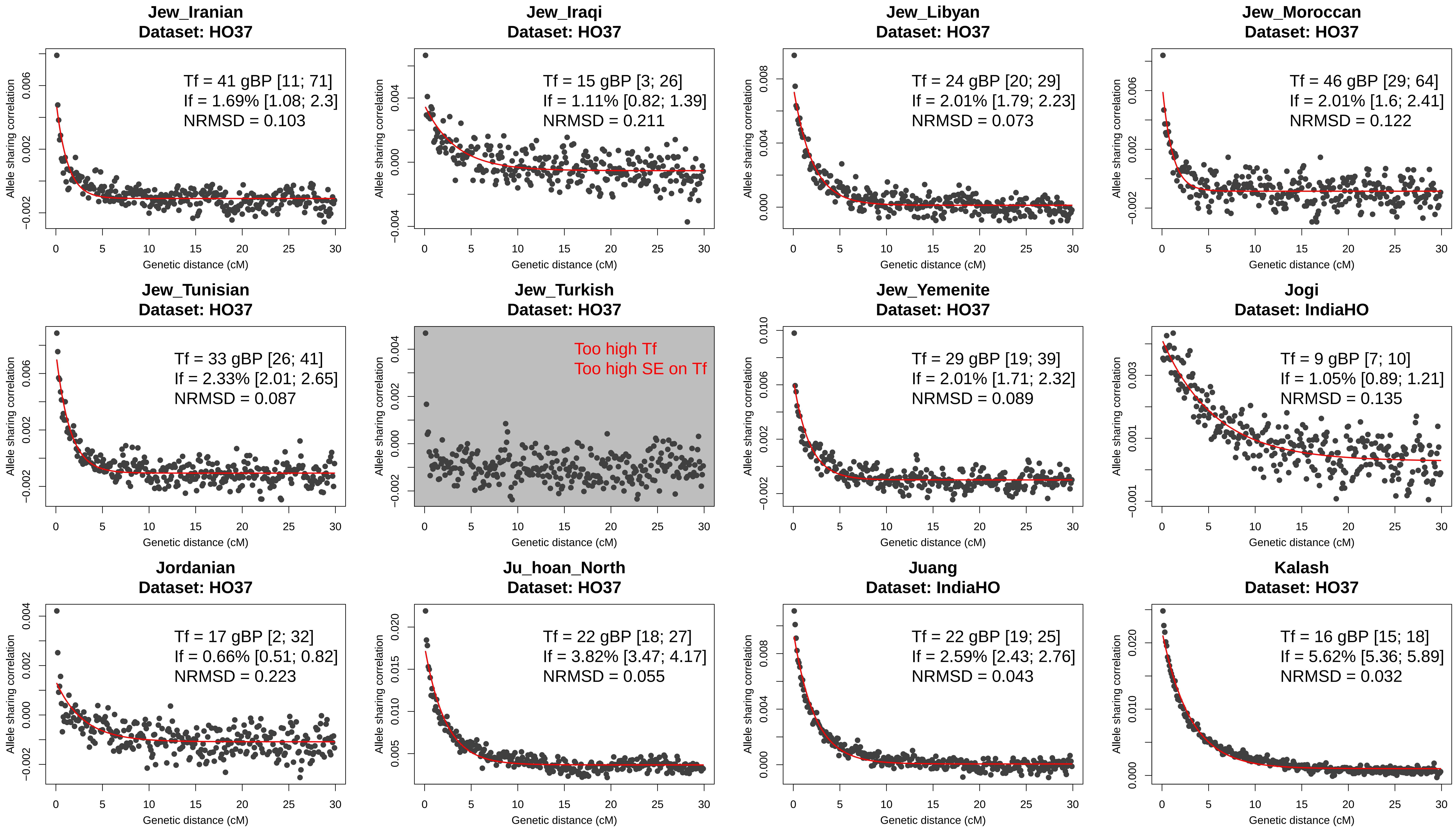
Jew_Iranian
Dataset: HO37
Tf = 41 gBP [11; 71]
If = 1.69% [1.08; 2.3]
NRMSD = 0.103
Jew_Iraqi
Dataset: HO37
Tf = 15 gBP [3; 26]
If = 1.11% [0.82; 1.39]
NRMSD = 0.211
Jew_Libyan
Dataset: HO37
Tf = 24 gBP [20; 29]
If = 2.01% [1.79; 2.23]
NRMSD = 0.073
Jew_Moroccan
Dataset: HO37
Tf = 46 gBP [29; 64]
If = 2.01% [1.6; 2.41]
NRMSD = 0.122
Jew_Tunisian
Dataset: HO37
Tf = 33 gBP [26; 41]
If = 2.33% [2.01; 2.65]
NRMSD = 0.087
Jew_Turkish
Dataset: HO37
Too high Tf
Too high SE on Tf
Jew_Yemenite
Dataset: HO37
Tf = 29 gBP [19; 39]
If = 2.01% [1.71; 2.32]
NRMSD = 0.089
Jogi
Dataset: IndiaHO
Tf = 9 gBP [7; 10]
If = 1.05% [0.89; 1.21]
NRMSD = 0.135
Jordanian
Dataset: HO37
Tf = 17 gBP [2; 32]
If = 0.66% [0.51; 0.82]
NRMSD = 0.223
Ju_hoan_North
Dataset: HO37
Tf = 22 gBP [18; 27]
If = 3.82% [3.47; 4.17]
NRMSD = 0.055
Juang
Dataset: IndiaHO
Tf = 22 gBP [19; 25]
If = 2.59% [2.43; 2.76]
NRMSD = 0.043
Kalash
Dataset: HO37
Tf = 16 gBP [15; 18]
If = 5.62% [5.36; 5.89]
NRMSD = 0.032
Allele sharing correlation
Genetic distance (cM)

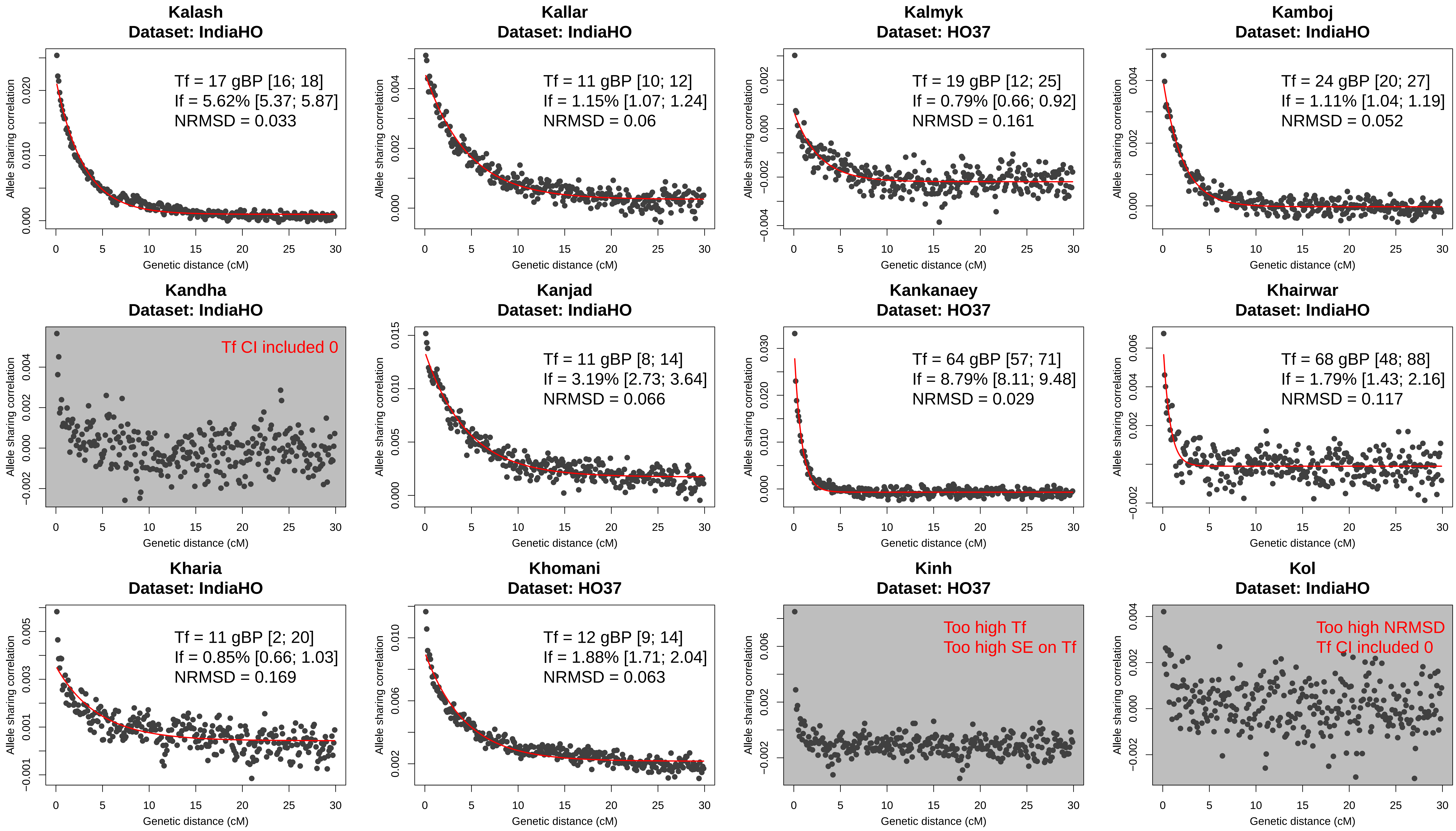
Kalash
Dataset: IndiaHO
Tf = 17 gBP [16; 18]
If = 5.62% [5.37; 5.87]
NRMSD = 0.033
Kallar
Dataset: IndiaHO
Tf = 11 gBP [10; 12]
If = 1.15% [1.07; 1.24]
NRMSD = 0.06
Kalmyk
Dataset: HO37
Tf = 19 gBP [12; 25]
If = 0.79% [0.66; 0.92]
NRMSD = 0.161
Kamboj
Dataset: IndiaHO
Tf = 24 gBP [20; 27]
If = 1.11% [1.04; 1.19]
NRMSD = 0.052
Kandha
Dataset: IndiaHO
Tf CI included 0
Kanjad
Dataset: IndiaHO
Tf = 11 gBP [8; 14]
If = 3.19% [2.73; 3.64]
NRMSD = 0.066
Kankanaey
Dataset: HO37
Tf = 64 gBP [57; 71]
If = 8.79% [8.11; 9.48]
NRMSD = 0.029
Khairwar
Dataset: IndiaHO
Tf = 68 gBP [48; 88]
If = 1.79% [1.43; 2.16]
NRMSD = 0.117
Kharia
Dataset: IndiaHO
Tf = 11 gBP [2; 20]
If = 0.85% [0.66; 1.03]
NRMSD = 0.169
Khomani
Dataset: HO37
Tf = 12 gBP [9; 14]
If = 1.88% [1.71; 2.04]
NRMSD = 0.063
Kinh
Dataset: HO37
Too high Tf
Too high SE on Tf
Kol
Dataset: IndiaHO
Too high NRMSD
Tf CI included 0
Allele sharing correlation
Genetic distance (cM)

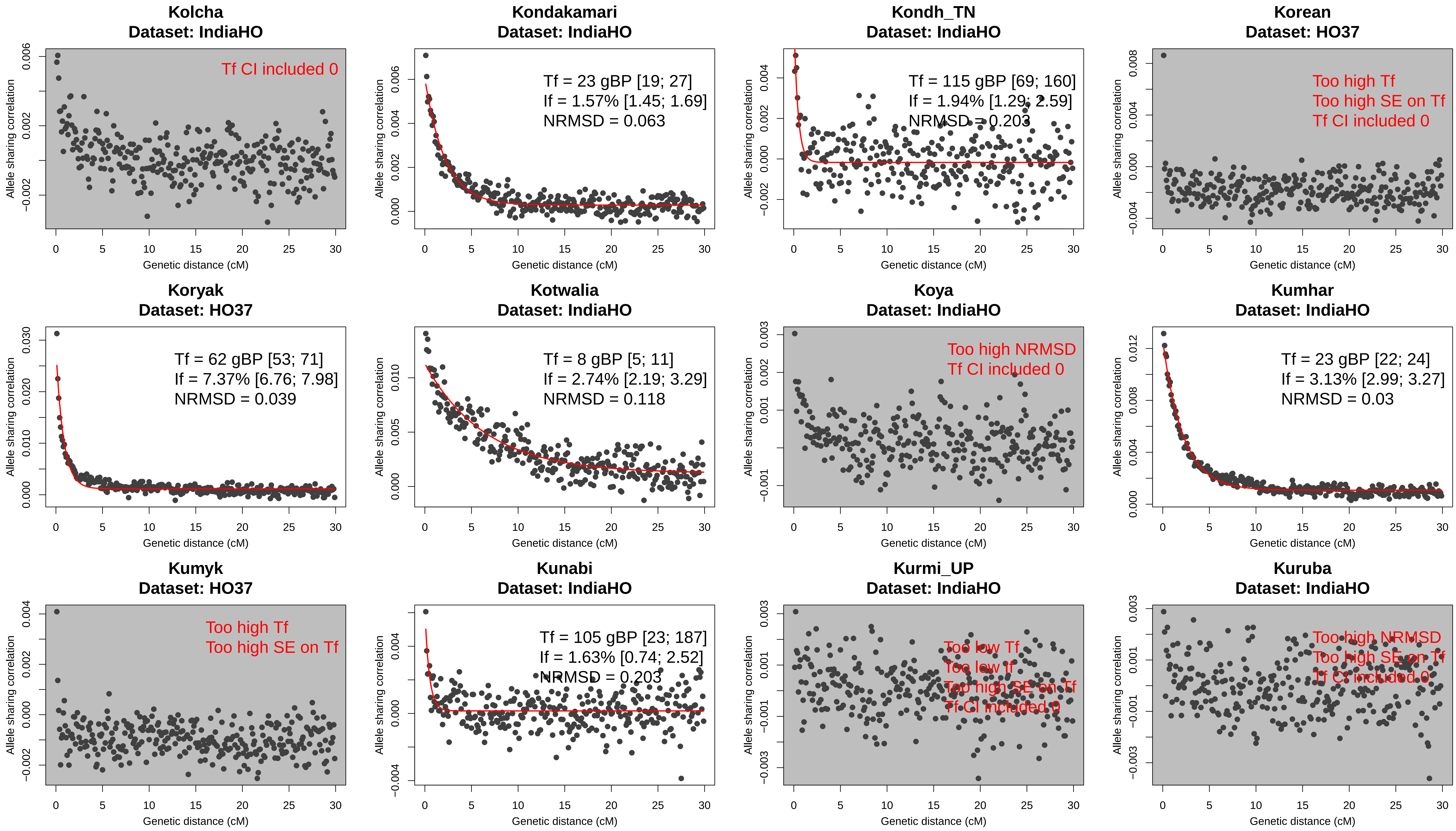
Kolcha
Dataset: IndiaHO
Tf CI included 0
Kondakamari
Dataset: IndiaHO
Tf = 23 gBP [19; 27]
If = 1.57% [1.45; 1.69]
NRMSD = 0.063
Kondh_TN
Dataset: IndiaHO
Tf = 115 gBP [69; 160]
If = 1.94% [1.29; 2.59]
NRMSD = 0.203
Korean
Dataset: HO37
Too high Tf
Too high SE on Tf
Tf CI included 0
Koryak
Dataset: HO37
Tf = 62 gBP [53; 71]
If = 7.37% [6.76; 7.98]
NRMSD = 0.039
Kotwalia
Dataset: IndiaHO
Tf = 8 gBP [5; 11]
If = 2.74% [2.19; 3.29]
NRMSD = 0.118
Koya
Dataset: IndiaHO
Too high NRMSD
Tf CI included 0
Kumhar
Dataset: IndiaHO
Tf = 23 gBP [22; 24]
If = 3.13% [2.99; 3.27]
NRMSD = 0.03
Kumyk
Dataset: HO37
Too high Tf
Too high SE on Tf
Kunabi
Dataset: IndiaHO
Tf = 105 gBP [23; 187]
If = 1.63% [0.74; 2.52]
NRMSD = 0.203
Kurmi_UP
Dataset: IndiaHO
Too low Tf
Too low If
Too high SE on Tf
Tf CI included 0
Kuruba
Dataset: IndiaHO
Too high NRMSD
Too high SE on Tf
Tf CI included 0
Allele sharing correlation
Genetic distance (cM)

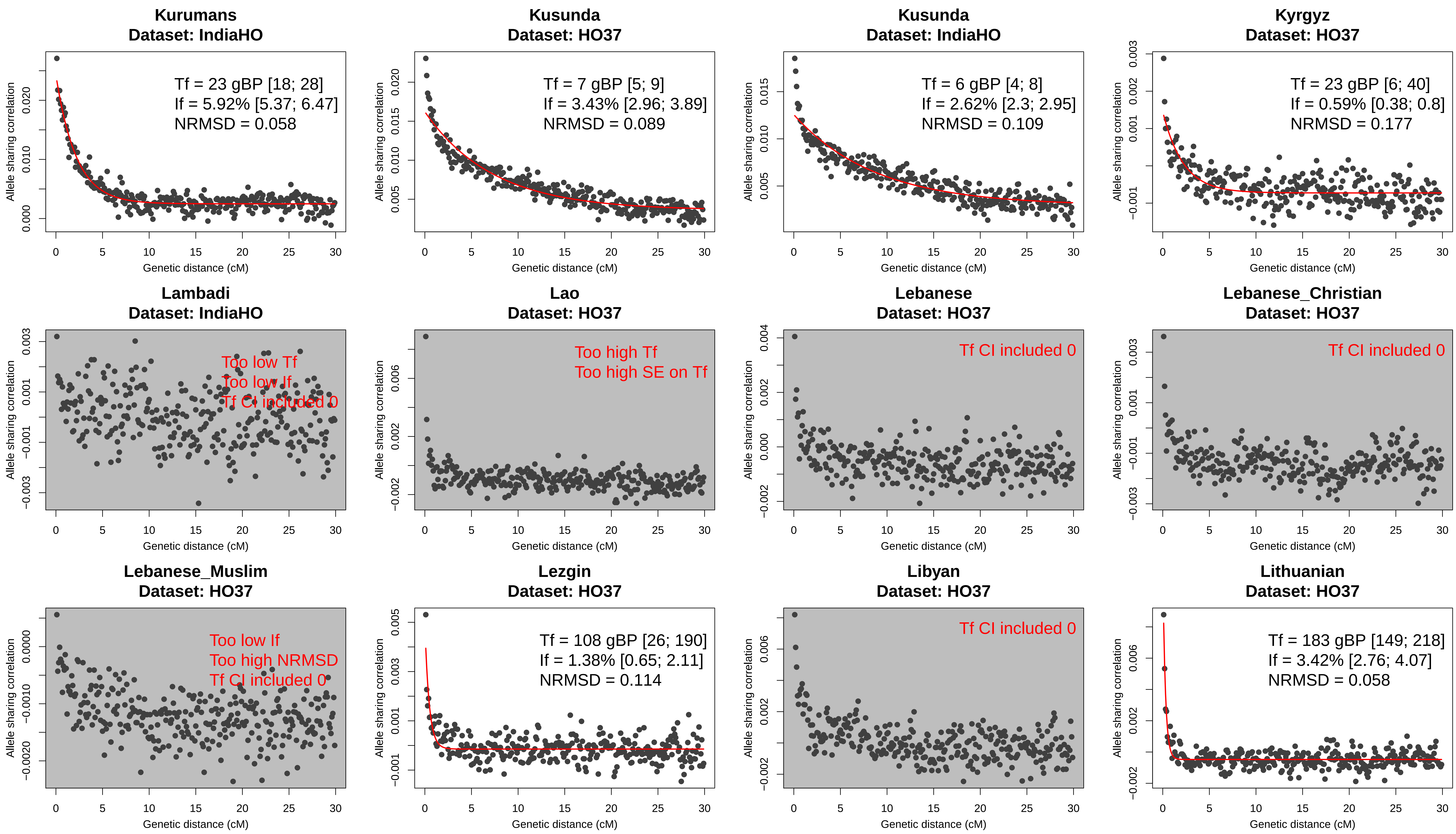
Kurumans
Dataset: IndiaHO
Tf = 23 gBP [18; 28]
If = 5.92% [5.37; 6.47]
NRMSD = 0.058
Kusunda
Dataset: HO37
Tf = 7 gBP [5; 9]
If = 3.43% [2.96; 3.89]
NRMSD = 0.089
Kusunda
Dataset: IndiaHO
Tf = 6 gBP [4; 8]
If = 2.62% [2.3; 2.95]
NRMSD = 0.109
Kyrgyz
Dataset: HO37
Tf = 23 gBP [6; 40]
If = 0.59% [0.38; 0.8]
NRMSD = 0.177
Lambadi
Dataset: IndiaHO
Too low Tf
Too low If
Tf CI included 0
Lao
Dataset: HO37
Too high Tf
Too high SE on Tf
Lebanese
Dataset: HO37
Tf CI included 0
Lebanese_Christian
Dataset: HO37
Tf CI included 0
Lebanese_Muslim
Dataset: HO37
Too low If
Too high NRMSD
Tf CI included 0
Lezgin
Dataset: HO37
Tf = 108 gBP [26; 190]
If = 1.38% [0.65; 2.11]
NRMSD = 0.114
Libyan
Dataset: HO37
Tf CI included 0
Lithuanian
Dataset: HO37
Tf = 183 gBP [149; 218]
If = 3.42% [2.76; 4.07]
NRMSD = 0.058
Allele sharing correlation
Genetic distance (cM)

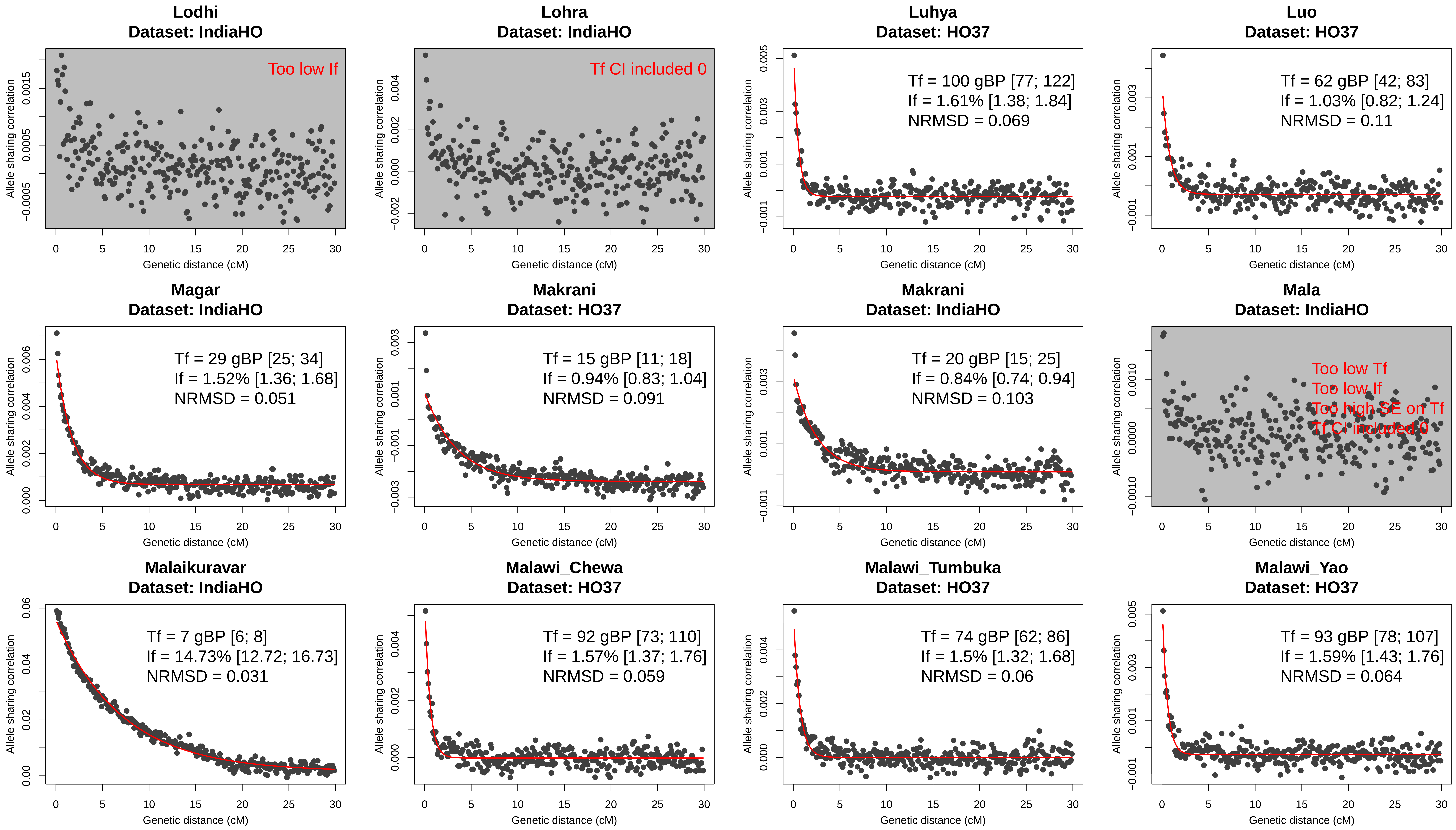

Lodhi
Dataset: IndiaHO
Too low If
Lohra
Dataset: IndiaHO
Tf CI included 0
Luhya
Dataset: HO37
Tf = 100 gBP [77; 122]
If = 1.61% [1.38; 1.84]
NRMSD = 0.069
Luo
Dataset: HO37
Tf = 62 gBP [42; 83]
If = 1.03% [0.82; 1.24]
NRMSD = 0.11
Magar
Dataset: IndiaHO
Tf = 29 gBP [25; 34]
If = 1.52% [1.36; 1.68]
NRMSD = 0.051
Makrani
Dataset: HO37
Tf = 15 gBP [11; 18]
If = 0.94% [0.83; 1.04]
NRMSD = 0.091
Makrani
Dataset: IndiaHO
Tf = 20 gBP [15; 25]
If = 0.84% [0.74; 0.94]
NRMSD = 0.103
Mala
Dataset: IndiaHO
Too low Tf
Too low If
Too high SE on Tf
Tf CI included 0
Malaikuravar
Dataset: IndiaHO
Tf = 7 gBP [6; 8]
If = 14.73% [12.72; 16.73]
NRMSD = 0.031
Malawi_Chewa
Dataset: HO37
Tf = 92 gBP [73; 110]
If = 1.57% [1.37; 1.76]
NRMSD = 0.059
Malawi_Tumbuka
Dataset: HO37
Tf = 74 gBP [62; 86]
If = 1.5% [1.32; 1.68]
NRMSD = 0.06
Malawi_Yao
Dataset: HO37
Tf = 93 gBP [78; 107]
If = 1.59% [1.43; 1.76]
NRMSD = 0.064
Allele sharing correlation
Genetic distance (cM)

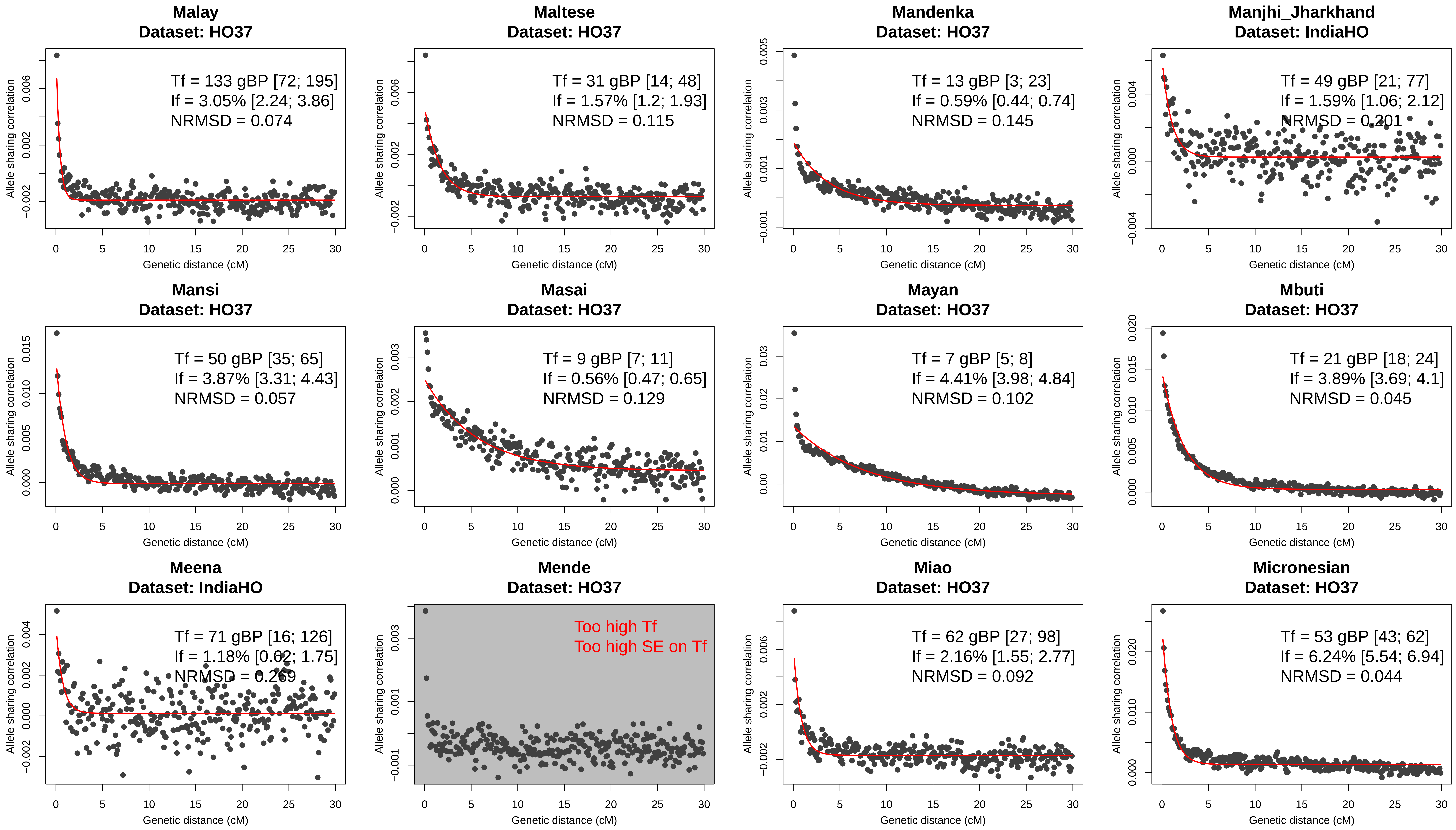
Malay
Dataset: HO37
Tf = 133 gBP [72; 195]
If = 3.05% [2.24; 3.86]
NRMSD = 0.074
Maltese
Dataset: HO37
Tf = 31 gBP [14; 48]
If = 1.57% [1.2; 1.93]
NRMSD = 0.115
Mandenka
Dataset: HO37
Tf = 13 gBP [3; 23]
If = 0.59% [0.44; 0.74]
NRMSD = 0.145
Manjhi_Jharkhand
Dataset: IndiaHO
Tf = 49 gBP [21; 77]
If = 1.59% [1.06; 2.12]
NRMSD = 0.201
Mansi
Dataset: HO37
Tf = 50 gBP [35; 65]
If = 3.87% [3.31; 4.43]
NRMSD = 0.057
Masai
Dataset: HO37
Tf = 9 gBP [7; 11]
If = 0.56% [0.47; 0.65]
NRMSD = 0.129
Mayan
Dataset: HO37
Tf = 7 gBP [5; 8]
If = 4.41% [3.98; 4.84]
NRMSD = 0.102
Mbuti
Dataset: HO37
Tf = 21 gBP [18; 24]
If = 3.89% [3.69; 4.1]
NRMSD = 0.045
Meena
Dataset: IndiaHO
Tf = 71 gBP [16; 126]
If = 1.18% [0.62; 1.75]
NRMSD = 0.269
Mende
Dataset: HO37
Too high Tf
Too high SE on Tf
Miao
Dataset: HO37
Tf = 62 gBP [27; 98]
If = 2.16% [1.55; 2.77]
NRMSD = 0.092
Micronesian
Dataset: HO37
Tf = 53 gBP [43; 62]
If = 6.24% [5.54; 6.94]
NRMSD = 0.044
Allele sharing correlation
Genetic distance (cM)

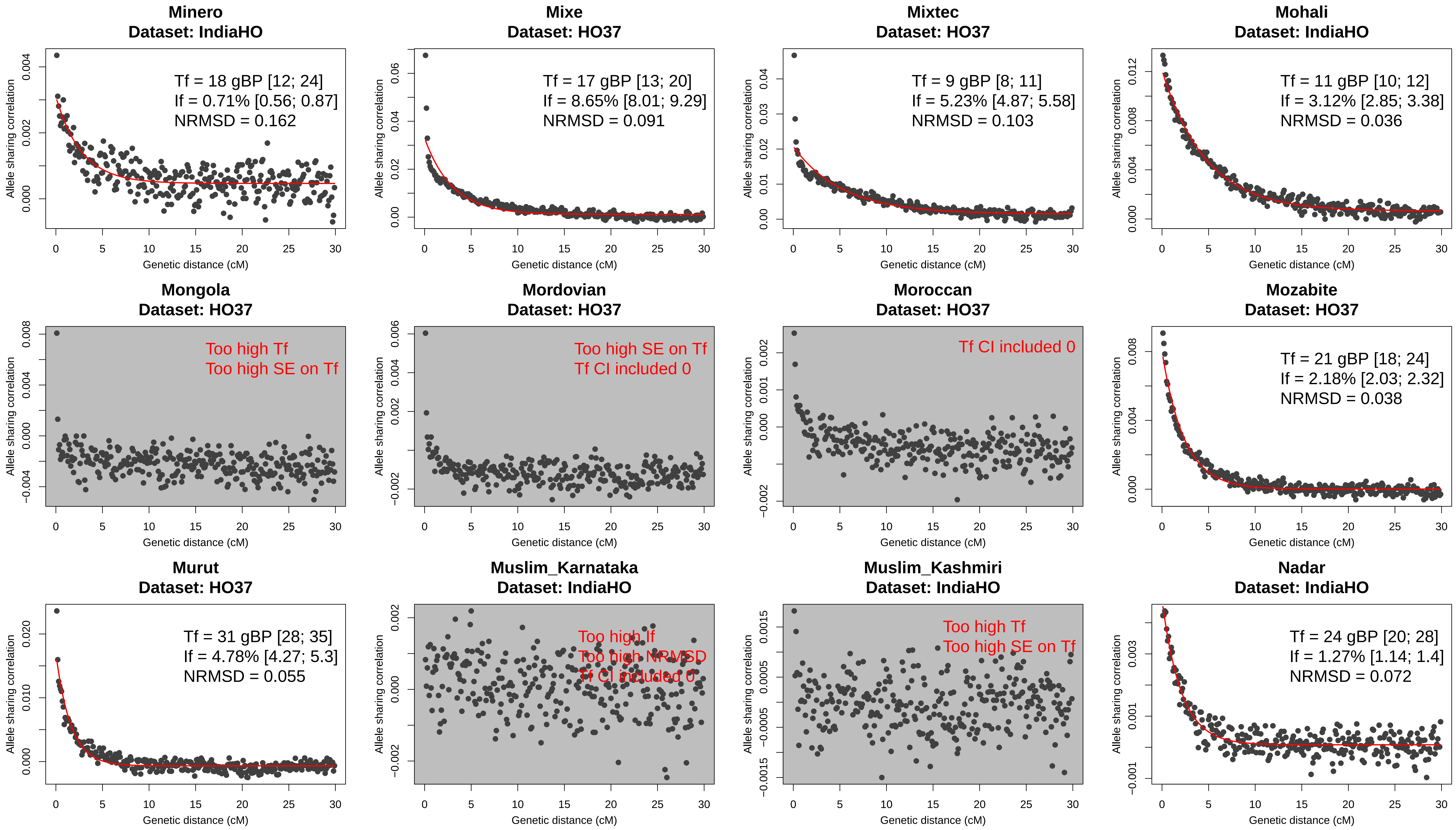
Minero
Dataset: IndiaHO
Tf = 18 gBP [12; 24]
If = 0.71% [0.56; 0.87]
NRMSD = 0.162
Mixe
Dataset: HO37
Tf = 17 gBP [13; 20]
If = 8.65% [8.01; 9.29]
NRMSD = 0.091
Mixtec
Dataset: HO37
Tf = 9 gBP [8; 11]
If = 5.23% [4.87; 5.58]
NRMSD = 0.103
Mohali
Dataset: IndiaHO
Tf = 11 gBP [10; 12]
If = 3.12% [2.85; 3.38]
NRMSD = 0.036
Mongola
Dataset: HO37
Too high Tf
Too high SE on Tf
Mordovian
Dataset: HO37
Too high SE on Tf
Tf CI included 0
Moroccan
Dataset: HO37
Tf CI included 0
Mozabite
Dataset: HO37
Tf = 21 gBP [18; 24]
If = 2.18% [2.03; 2.32]
NRMSD = 0.038
Murut
Dataset: HO37
Tf = 31 gBP [28; 35]
If = 4.78% [4.27; 5.3]
NRMSD = 0.055
Muslim_Karnataka
Dataset: IndiaHO
Too high If
Too high NRMSD
Tf CI included 0
Muslim_Kashmiri
Dataset: IndiaHO
Too high Tf
Too high SE on Tf
Nadar
Dataset: IndiaHO
Tf = 24 gBP [20; 28]
If = 1.27% [1.14; 1.4]
NRMSD = 0.072
Allele sharing correlation
Genetic distance (cM)

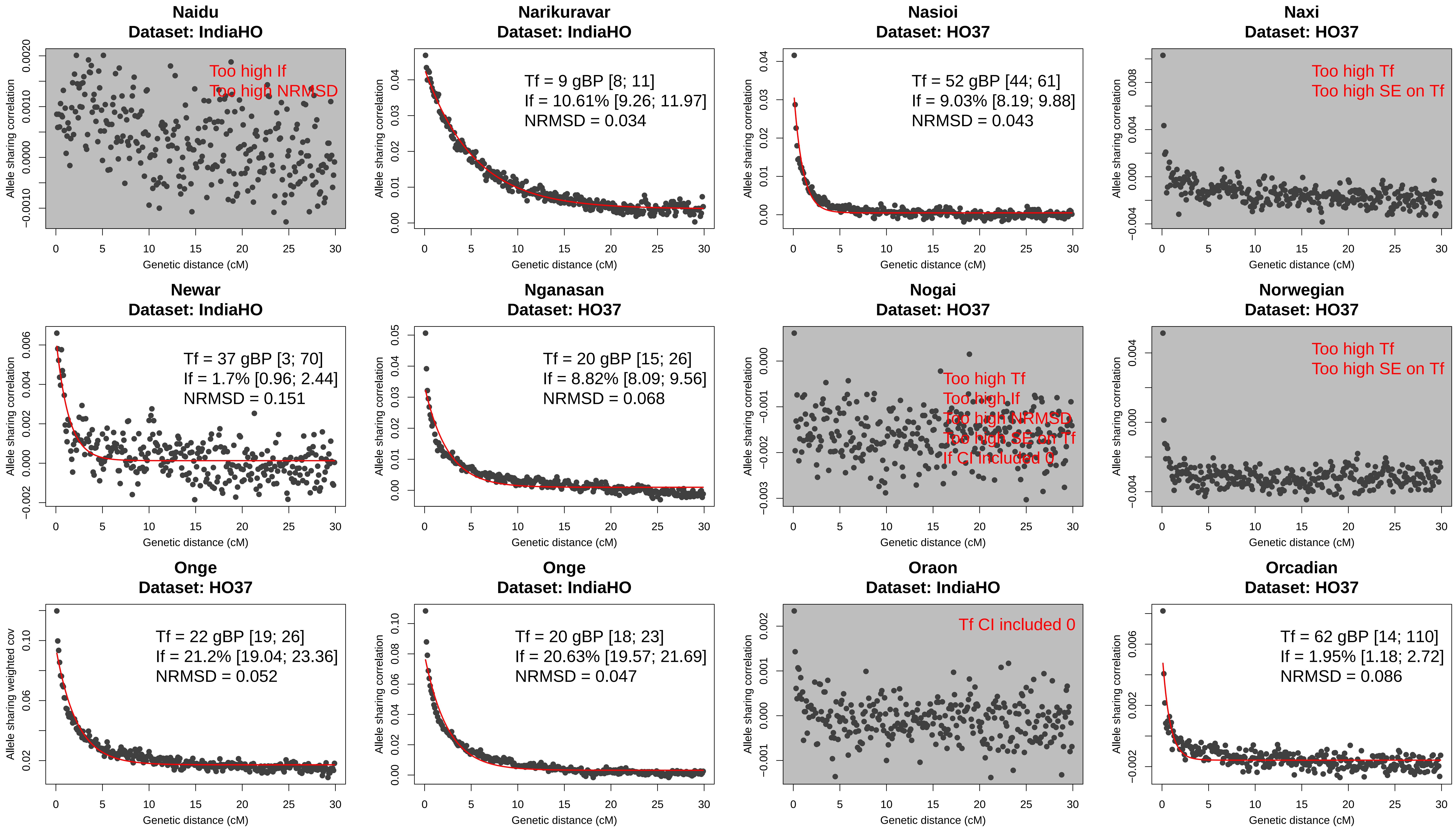
Naidu
Dataset: IndiaHO
Too high If
Too high NRMSD
Narikuravar
Dataset: IndiaHO
Tf = 9 gBP [8; 11]
If = 10.61% [9.26; 11.97]
NRMSD = 0.034
Nasioi
Dataset: HO37
Tf = 52 gBP [44; 61]
If = 9.03% [8.19; 9.88]
NRMSD = 0.043
Naxi
Dataset: HO37
Too high Tf
Too high SE on Tf
Newar
Dataset: IndiaHO
Tf = 37 gBP [3; 70]
If = 1.7% [0.96; 2.44]
NRMSD = 0.151
Nganasan
Dataset: HO37
Tf = 20 gBP [15; 26]
If = 8.82% [8.09; 9.56]
NRMSD = 0.068
Nogai
Dataset: HO37
Too high Tf
Too high If
Too high NRMSD
Too high SE on Tf
If CI included 0
Norwegian
Dataset: HO37
Too high Tf
Too high SE on Tf
Onge
Dataset: HO37
Tf = 22 gBP [19; 26]
If = 21.2% [19.04; 23.36]
NRMSD = 0.052
Onge
Dataset: IndiaHO
Tf = 20 gBP [18; 23]
If = 20.63% [19.57; 21.69]
NRMSD = 0.047
Oraon
Dataset: IndiaHO
Tf CI included 0
Orcadian
Dataset: HO37
Tf = 62 gBP [14; 110]
If = 1.95% [1.18; 2.72]
NRMSD = 0.086
Allele sharing correlation
Allele sharing weighted cov
Genetic distance (cM)

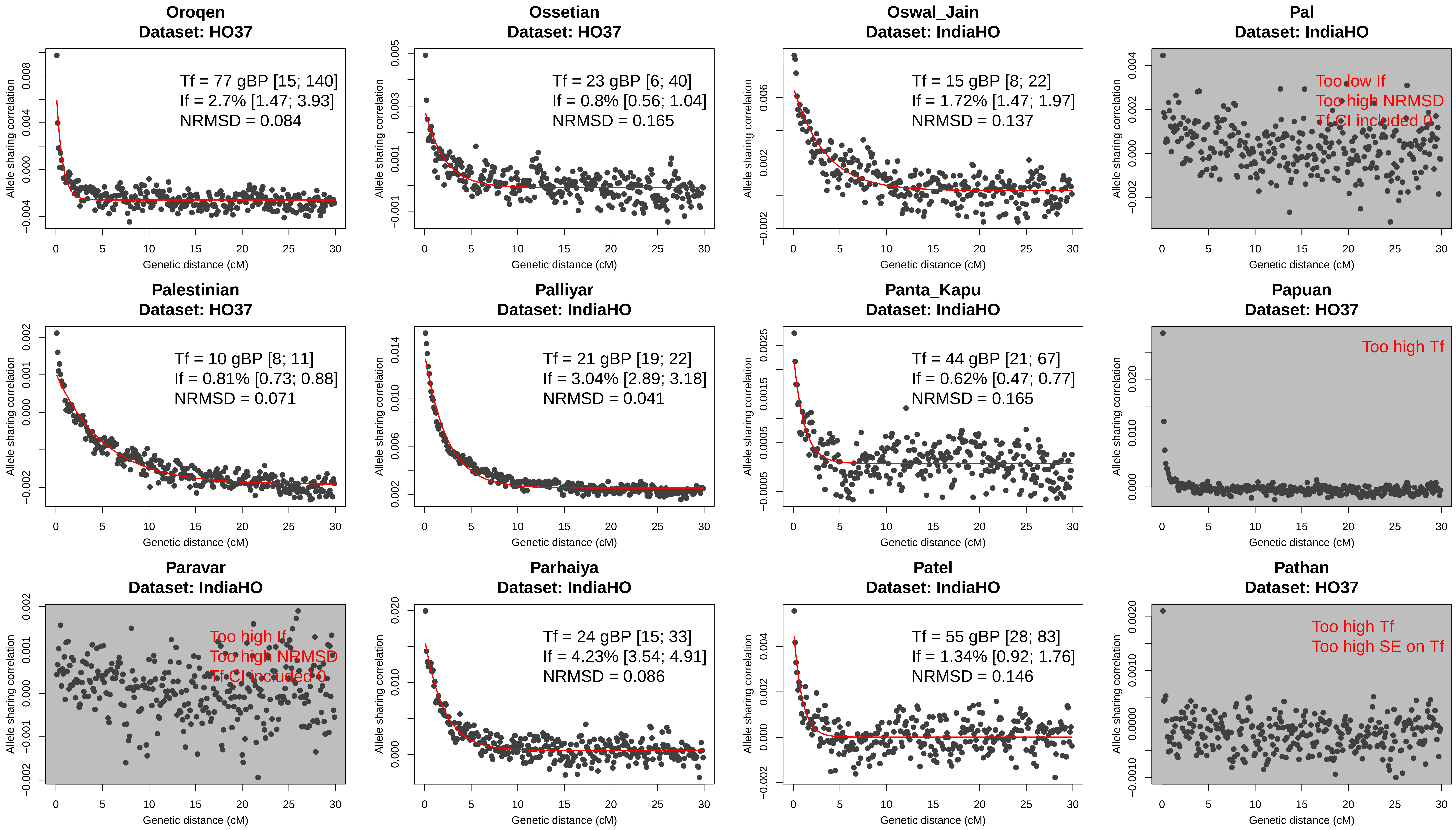
Oroqen
Dataset: HO37
Tf = 77 gBP [15; 140]
If = 2.7% [1.47; 3.93]
NRMSD = 0.084
Ossetian
Dataset: HO37
Tf = 23 gBP [6; 40]
If = 0.8% [0.56; 1.04]
NRMSD = 0.165
Oswal_Jain
Dataset: IndiaHO
Tf = 15 gBP [8; 22]
If = 1.72% [1.47; 1.97]
NRMSD = 0.137
Pal
Dataset: IndiaHO
Too low If
Too high NRMSD
Tf CI included 0
Palestinian
Dataset: HO37
Tf = 10 gBP [8; 11]
If = 0.81% [0.73; 0.88]
NRMSD = 0.071
Palliyar
Dataset: IndiaHO
Tf = 21 gBP [19; 22]
If = 3.04% [2.89; 3.18]
NRMSD = 0.041
Panta_Kapu
Dataset: IndiaHO
Tf = 44 gBP [21; 67]
If = 0.62% [0.47; 0.77]
NRMSD = 0.165
Papuan
Dataset: HO37
Too high Tf
Paravar
Dataset: IndiaHO
Too high If
Too high NRMSD
Tf CI included 0
Parhaiya
Dataset: IndiaHO
Tf = 24 gBP [15; 33]
If = 4.23% [3.54; 4.91]
NRMSD = 0.086
Patel
Dataset: IndiaHO
Tf = 55 gBP [28; 83]
If = 1.34% [0.92; 1.76]
NRMSD = 0.146
Pathan
Dataset: HO37
Too high Tf
Too high SE on Tf
Allele sharing correlation
Genetic distance (cM)

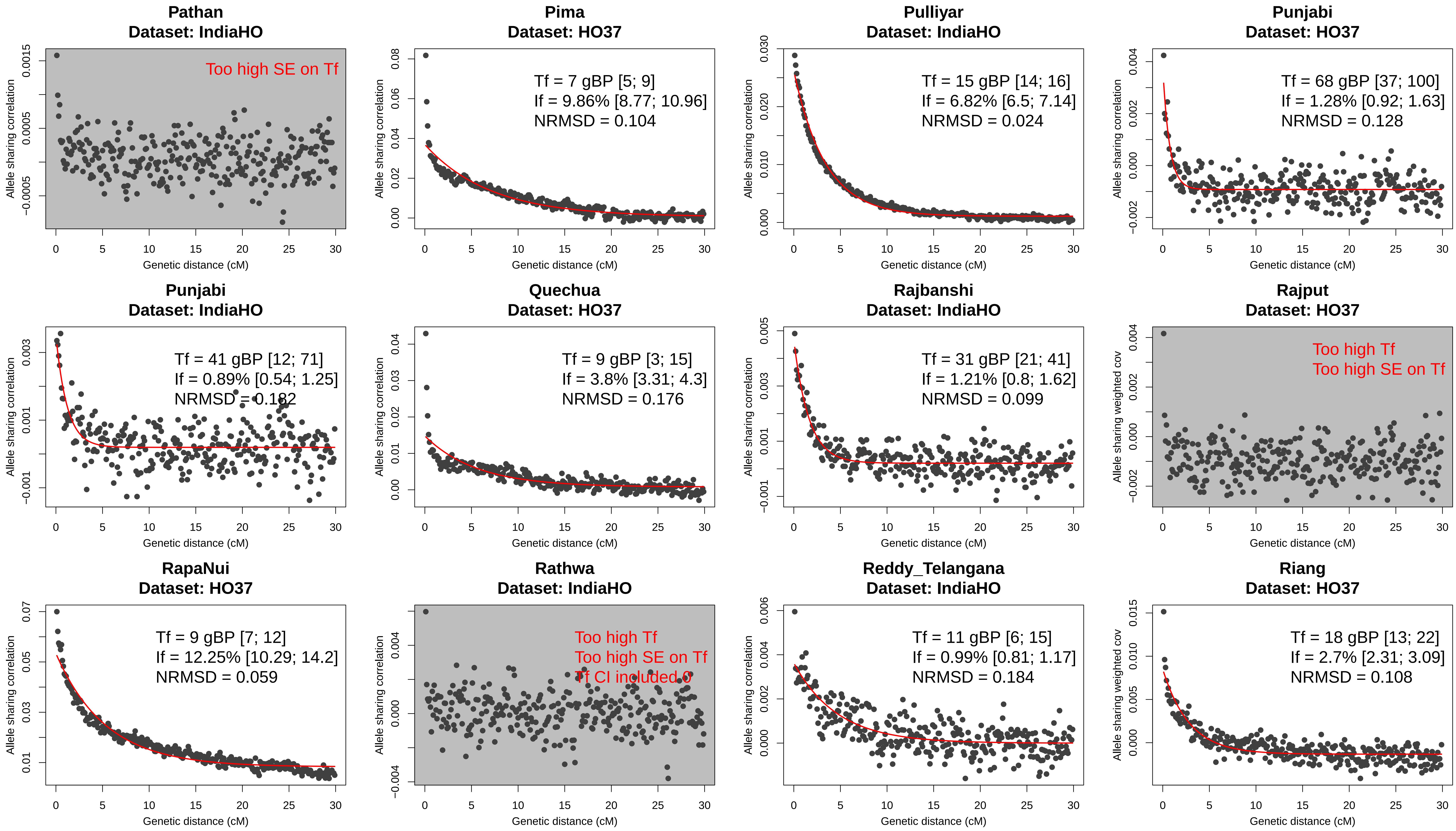
Pathan
Dataset: IndiaHO
Too high SE on Tf
Pima
Dataset: HO37
Tf = 7 gBP [5; 9]
If = 9.86% [8.77; 10.96]
NRMSD = 0.104
Pulliyar
Dataset: IndiaHO
Tf = 15 gBP [14; 16]
If = 6.82% [6.5; 7.14]
NRMSD = 0.024
Punjabi
Dataset: HO37
Tf = 68 gBP [37; 100]
If = 1.28% [0.92; 1.63]
NRMSD = 0.128
Punjabi
Dataset: IndiaHO
Tf = 41 gBP [12; 71]
If = 0.89% [0.54; 1.25]
NRMSD = 0.182
Quechua
Dataset: HO37
Tf = 9 gBP [3; 15]
If = 3.8% [3.31; 4.3]
NRMSD = 0.176
Rajbanshi
Dataset: IndiaHO
Tf = 31 gBP [21; 41]
If = 1.21% [0.8; 1.62]
NRMSD = 0.099
Rajput
Dataset: HO37
Too high Tf
Too high SE on Tf
RapaNui
Dataset: HO37
Tf = 9 gBP [7; 12]
If = 12.25% [10.29; 14.2]
NRMSD = 0.059
Rathwa
Dataset: IndiaHO
Too high Tf
Too high SE on Tf
Tf CI included 0
Reddy_Telangana
Dataset: IndiaHO
Tf = 11 gBP [6; 15]
If = 0.99% [0.81; 1.17]
NRMSD = 0.184
Riang
Dataset: HO37
Tf = 18 gBP [13; 22]
If = 2.7% [2.31; 3.09]
NRMSD = 0.108
Allele sharing correlation
Allele sharing weighted cov
Genetic distance (cM)

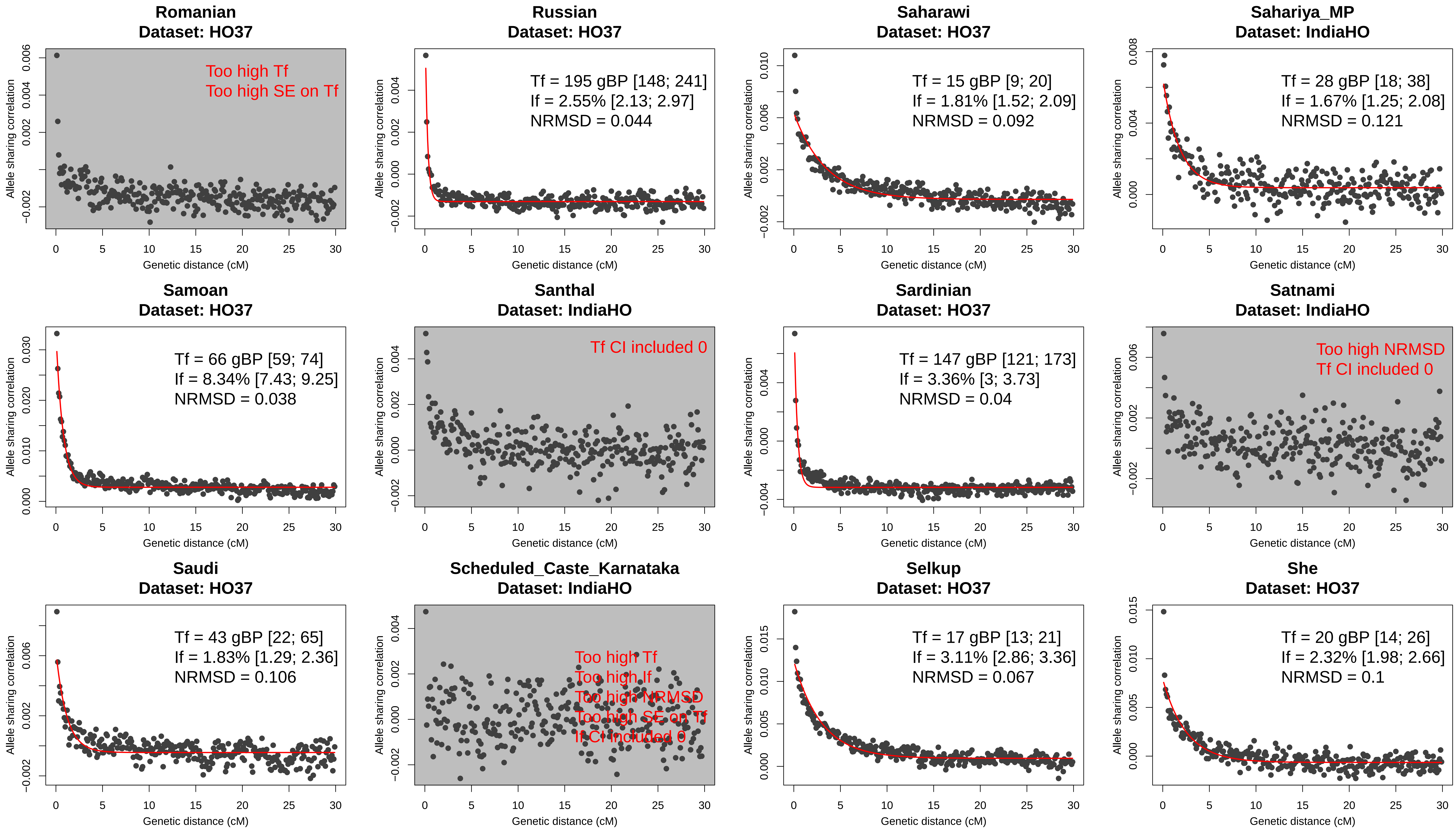
Romanian
Dataset: HO37
Too high Tf
Too high SE on Tf
Russian
Dataset: HO37
Tf = 195 gBP [148; 241]
If = 2.55% [2.13; 2.97]
NRMSD = 0.044
Saharawi
Dataset: HO37
Tf = 15 gBP [9; 20]
If = 1.81% [1.52; 2.09]
NRMSD = 0.092
Sahariya_MP
Dataset: IndiaHO
Tf = 28 gBP [18; 38]
If = 1.67% [1.25; 2.08]
NRMSD = 0.121
Samoan
Dataset: HO37
Tf = 66 gBP [59; 74]
If = 8.34% [7.43; 9.25]
NRMSD = 0.038
Santhal
Dataset: IndiaHO
Tf CI included 0
Sardinian
Dataset: HO37
Tf = 147 gBP [121; 173]
If = 3.36% [3; 3.73]
NRMSD = 0.04
Satnami
Dataset: IndiaHO
Too high NRMSD
Tf CI included 0
Saudi
Dataset: HO37
Tf = 43 gBP [22; 65]
If = 1.83% [1.29; 2.36]
NRMSD = 0.106
Scheduled_Caste_Karnataka
Dataset: IndiaHO
Too high Tf
Too high If
Too high NRMSD
Too high SE on Tf
If CI included 0
Selkup
Dataset: HO37
Tf = 17 gBP [13; 21]
If = 3.11% [2.86; 3.36]
NRMSD = 0.067
She
Dataset: HO37
Tf = 20 gBP [14; 26]
If = 2.32% [1.98; 2.66]
NRMSD = 0.1
Allele sharing correlation
Genetic distance (cM)

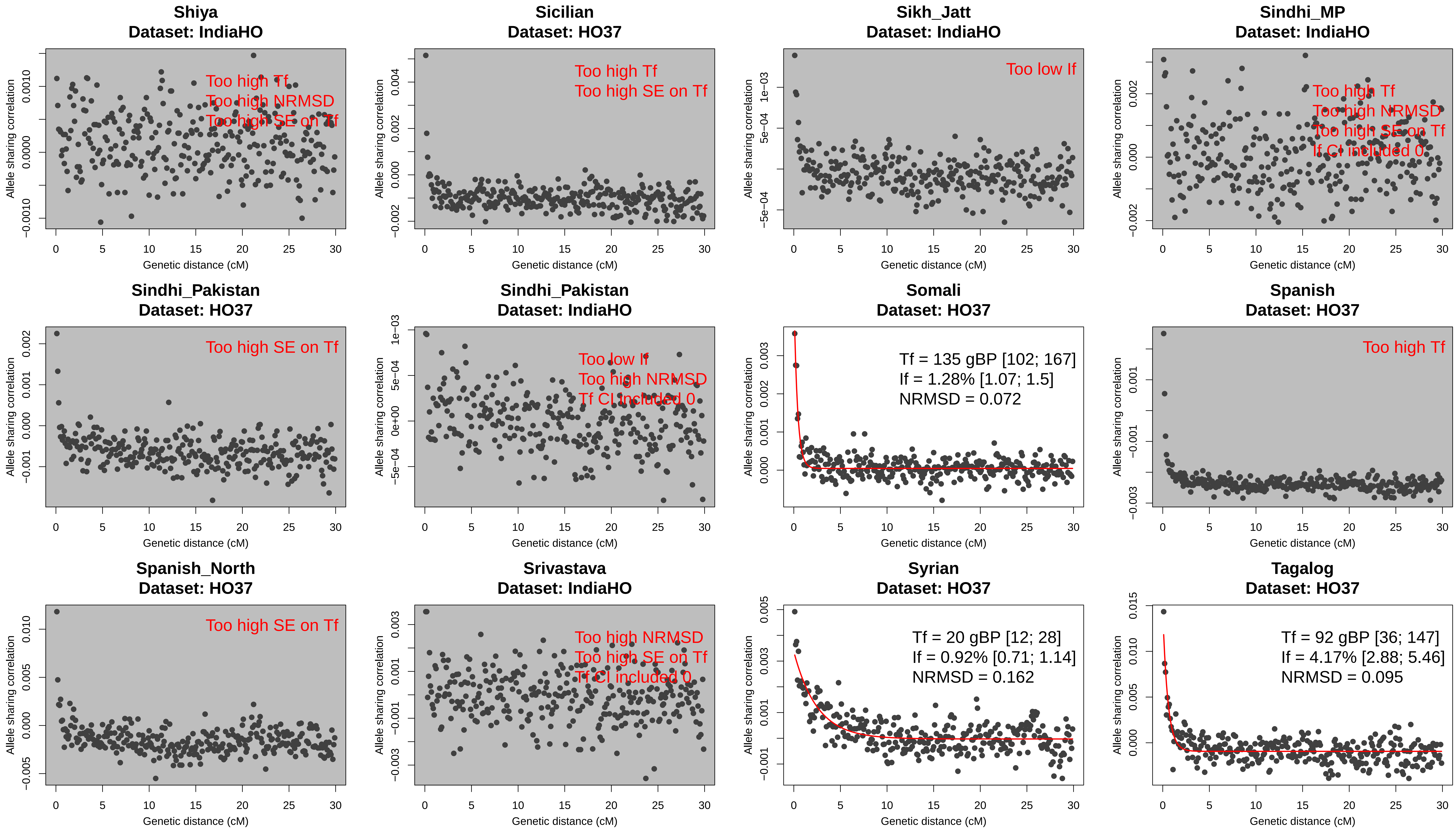
Shiya
Dataset: IndiaHO
Too high Tf
Too high NRMSD
Too high SE on Tf
Sicilian
Dataset: HO37
Too high Tf
Too high SE on Tf
Sikh_Jatt
Dataset: IndiaHO
Too low If
Sindhi_MP
Dataset: IndiaHO
Too high Tf
Too high NRMSD
Too high SE on Tf
If CI included 0
Sindhi_Pakistan
Dataset: HO37
Too high SE on Tf
Sindhi_Pakistan
Dataset: IndiaHO
Too low If
Too high NRMSD
Tf CI included 0
Somali
Dataset: HO37
Tf = 135 gBP [102; 167]
If = 1.28% [1.07; 1.5]
NRMSD = 0.072
Spanish
Dataset: HO37
Too high Tf
Spanish_North
Dataset: HO37
Too high SE on Tf
Srivastava
Dataset: IndiaHO
Too high NRMSD
Too high SE on Tf
Tf CI included 0
Syrian
Dataset: HO37
Tf = 20 gBP [12; 28]
If = 0.92% [0.71; 1.14]
NRMSD = 0.162
Tagalog
Dataset: HO37
Tf = 92 gBP [36; 147]
If = 4.17% [2.88; 5.46]
NRMSD = 0.095
Allele sharing correlation
Genetic distance (cM)

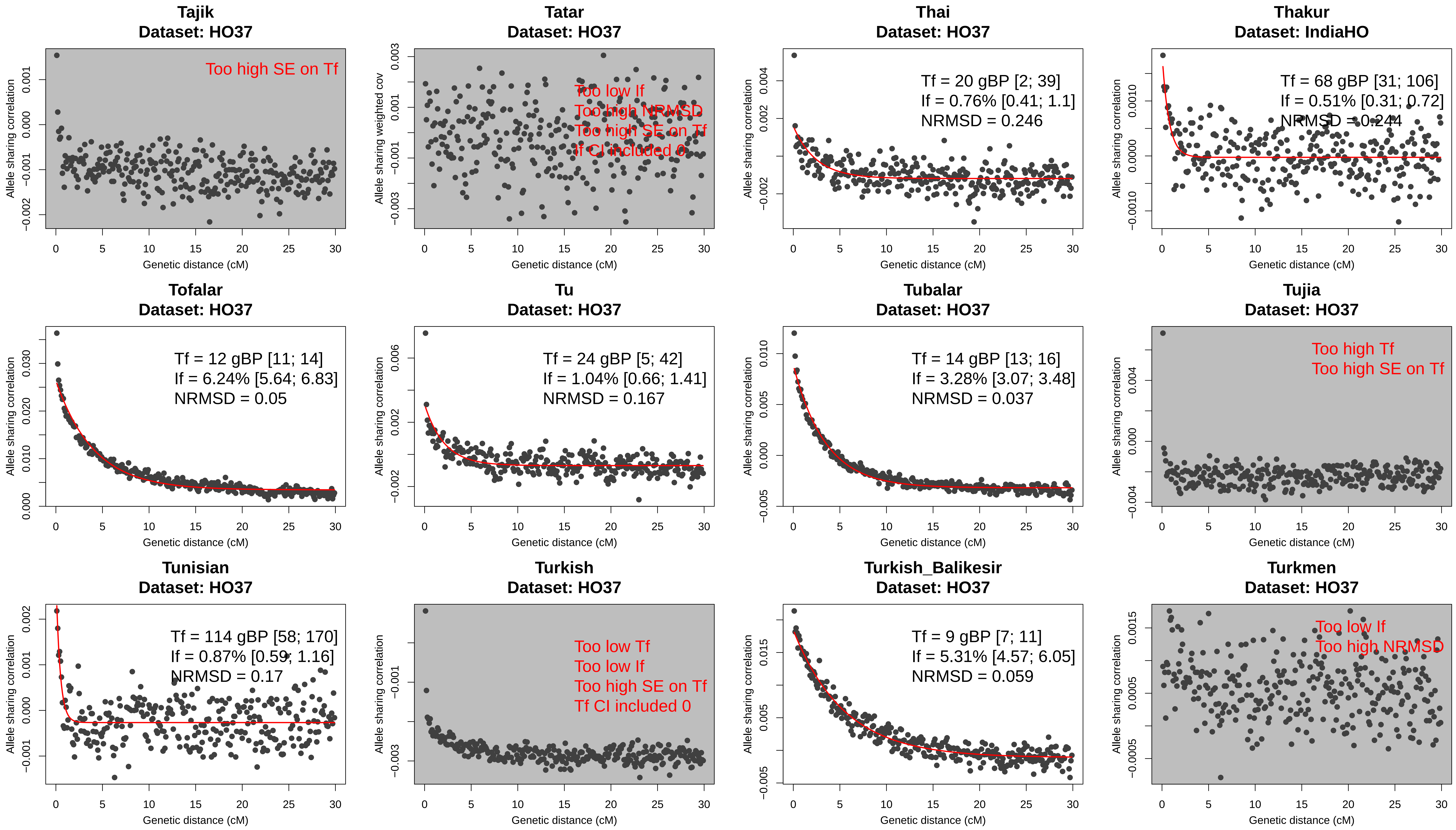

Tajik
Dataset: HO37
Too high SE on Tf
Tatar
Dataset: HO37
Too low If
Too high NRMSD
Too high SE on Tf
If CI included 0
Thai
Dataset: HO37
Tf = 20 gBP [2; 39]
If = 0.76% [0.41; 1.1]
NRMSD = 0.246
Thakur
Dataset: IndiaHO
Tf = 68 gBP [31; 106]
If = 0.51% [0.31; 0.72]
NRMSD = 0.244
Tofalar
Dataset: HO37
Tf = 12 gBP [11; 14]
If = 6.24% [5.64; 6.83]
NRMSD = 0.05
Tu
Dataset: HO37
Tf = 24 gBP [5; 42]
If = 1.04% [0.66; 1.41]
NRMSD = 0.167
Tubalar
Dataset: HO37
Tf = 14 gBP [13; 16]
If = 3.28% [3.07; 3.48]
NRMSD = 0.037
Tujia
Dataset: HO37
Too high Tf
Too high SE on Tf
Tunisian
Dataset: HO37
Tf = 114 gBP [58; 170]
If = 0.87% [0.59; 1.16]
NRMSD = 0.17
Turkish
Dataset: HO37
Too low Tf
Too low If
Too high SE on Tf
Tf CI included 0
Turkish_Balikesir
Dataset: HO37
Tf = 9 gBP [7; 11]
If = 5.31% [4.57; 6.05]
NRMSD = 0.059
Turkmen
Dataset: HO37
Too low If
Too high NRMSD
Allele sharing correlation
Allele sharing weighted cov
Genetic distance (cM)

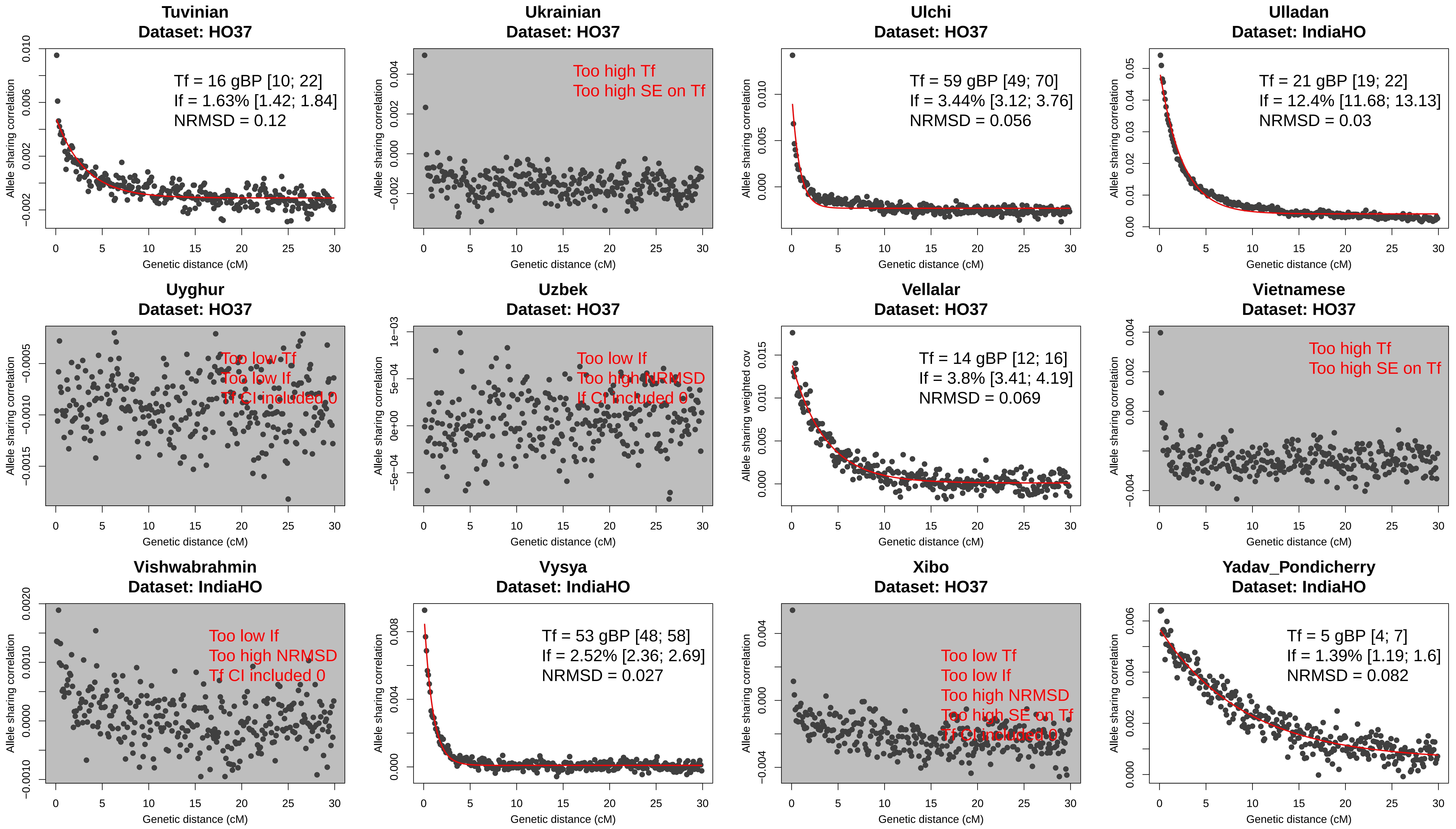
Tuvinian
Dataset: HO37
Tf = 16 gBP [10; 22]
If = 1.63% [1.42; 1.84]
NRMSD = 0.12
Ukrainian
Dataset: HO37
Too high Tf
Too high SE on Tf
Ulchi
Dataset: HO37
Tf = 59 gBP [49; 70]
If = 3.44% [3.12; 3.76]
NRMSD = 0.056
Ulladan
Dataset: IndiaHO
Tf = 21 gBP [19; 22]
If = 12.4% [11.68; 13.13]
NRMSD = 0.03
Uyghur
Dataset: HO37
Too low Tf
Too low If
Tf CI included 0
Uzbek
Dataset: HO37
Too low If
Too high NRMSD
If CI included 0
Vellalar
Dataset: HO37
Tf = 14 gBP [12; 16]
If = 3.8% [3.41; 4.19]
NRMSD = 0.069
Vietnamese
Dataset: HO37
Too high Tf
Too high SE on Tf
Vishwabrahmin
Dataset: IndiaHO
Too low If
Too high NRMSD
Tf CI included 0
Vysya
Dataset: IndiaHO
Tf = 53 gBP [48; 58]
If = 2.52% [2.36; 2.69]
NRMSD = 0.027
Xibo
Dataset: HO37
Too low Tf
Too low If
Too high NRMSD
Too high SE on Tf
Tf CI included 0
Yadav_Pondicherry
Dataset: IndiaHO
Tf = 5 gBP [4; 7]
If = 1.39% [1.19; 1.6]
NRMSD = 0.082
Allele sharing correlation
Allele sharing weighted cov
Genetic distance (cM)

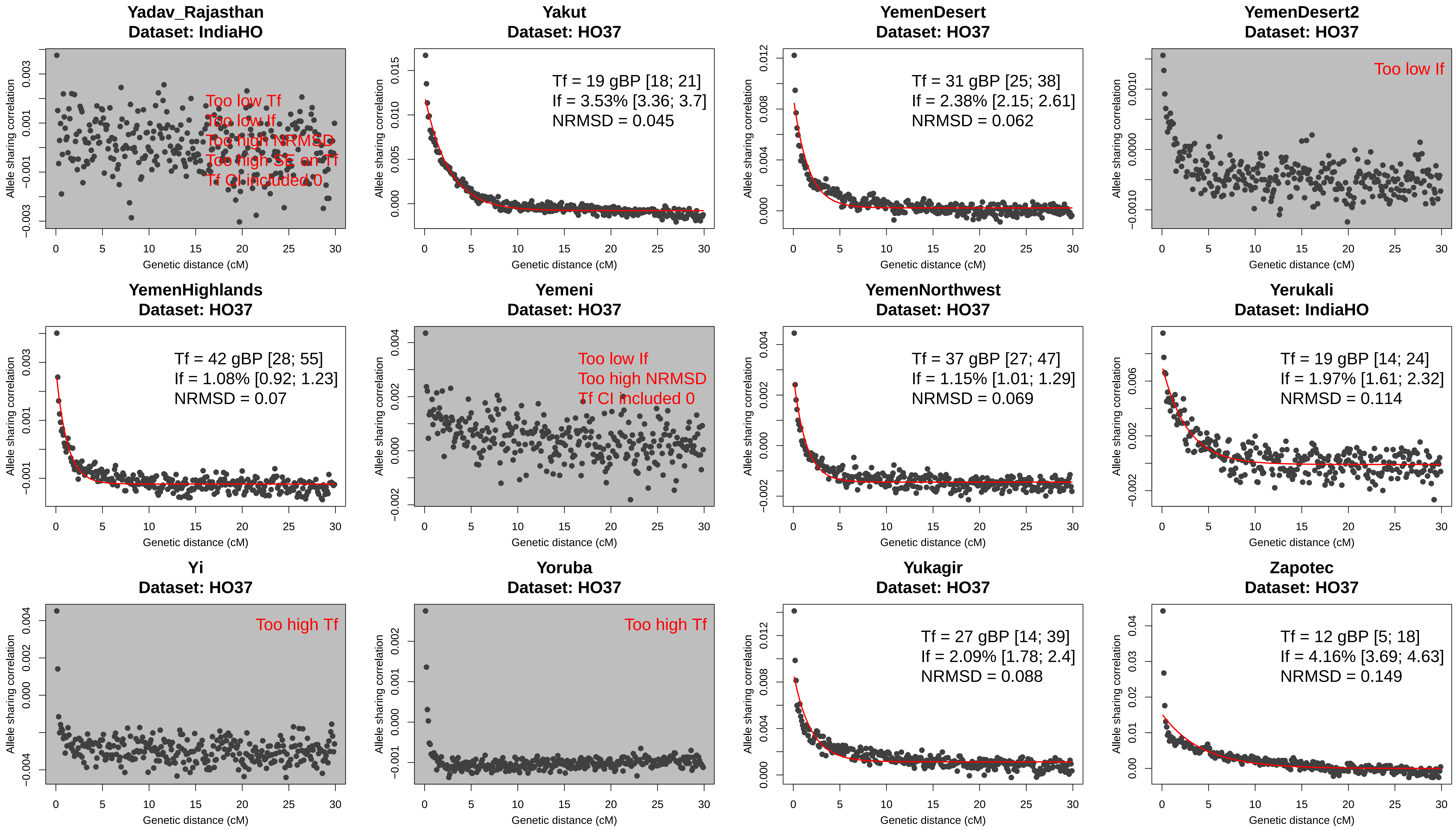

Yadav_Rajasthan
Dataset: IndiaHO
Too low Tf
Too low If
Too high NRMSD
Too high SE on Tf
Tf CI included 0
Yakut
Dataset: HO37
Tf = 19 gBP [18; 21]
If = 3.53% [3.36; 3.7]
NRMSD = 0.045
YemenDesert
Dataset: HO37
Tf = 31 gBP [25; 38]
If = 2.38% [2.15; 2.61]
NRMSD = 0.062
YemenDesert2
Dataset: HO37
Too low If
YemenHighlands
Dataset: HO37
Tf = 42 gBP [28; 55]
If = 1.08% [0.92; 1.23]
NRMSD = 0.07
Yemeni
Dataset: HO37
Too low If
Too high NRMSD
Tf CI included 0
YemenNorthwest
Dataset: HO37
Tf = 37 gBP [27; 47]
If = 1.15% [1.01; 1.29]
NRMSD = 0.069
Yerukali
Dataset: IndiaHO
Tf = 19 gBP [14; 24]
If = 1.97% [1.61; 2.32]
NRMSD = 0.114
Yi
Dataset: HO37
Too high Tf
Yoruba
Dataset: HO37
Too high Tf
Yukagir
Dataset: HO37
Tf = 27 gBP [14; 39]
If = 2.09% [1.78; 2.4]
NRMSD = 0.088
Zapotec
Dataset: HO37
Tf = 12 gBP [5; 18]
If = 4.16% [3.69; 4.63]
NRMSD = 0.149
Allele sharing correlation
Genetic distance (cM)
