# Supplementary figures and images for: Reconstructing the history of founder events using genome-wide patterns of allele sharing across individuals
Source: PLoS Genet. 2022 Jun 23;18(6):e1010243. doi: 10.1371/journal.pgen.1010243 (PMC9223333; doi:10.1371/journal.pgen.1010243)

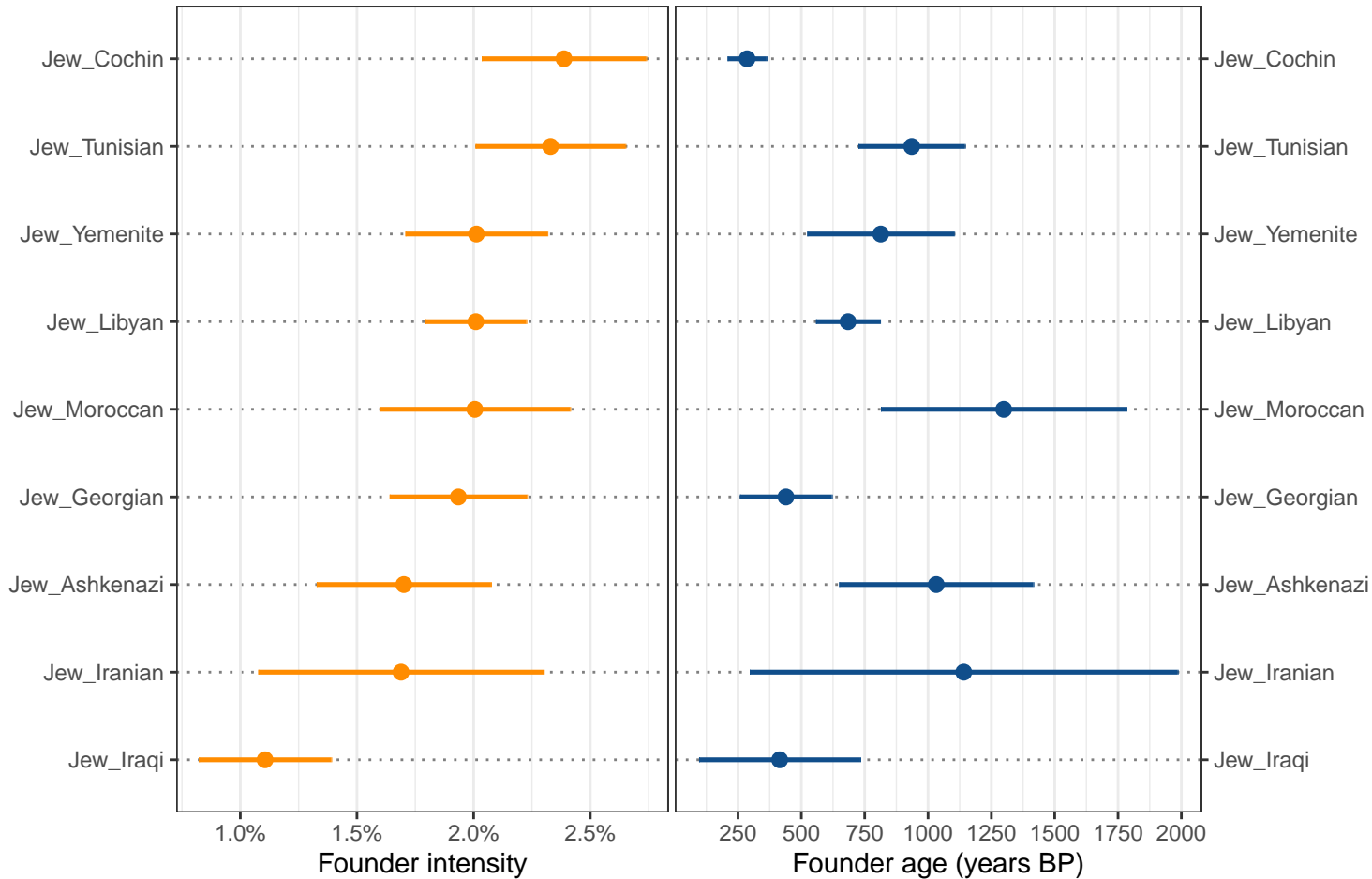

Supplement: S2 Fig — Panel (A) shows the distribution of founder intensity, and panel (B) shows the distribution of founder ages for all Jewish populations that passed our filtering criteria in the HO37 dataset and showed evidence for a significant founder event (see Methods). The populations are ordered by decreasing order of founder intensity, from top to bottom. (A) Distribution of the estimated founder intensities. We show the mean founder intensities (points) and their associated 95% confidence intervals. (B) Distribution of the estimated founder ages. We show the estimated founder ages and their associated 95% confidence intervals. The estimated ages were converted from generations to years before present by using a generation time of 28 years [29,30]. (PDF) [file pgen.1010243.s002.pdf]

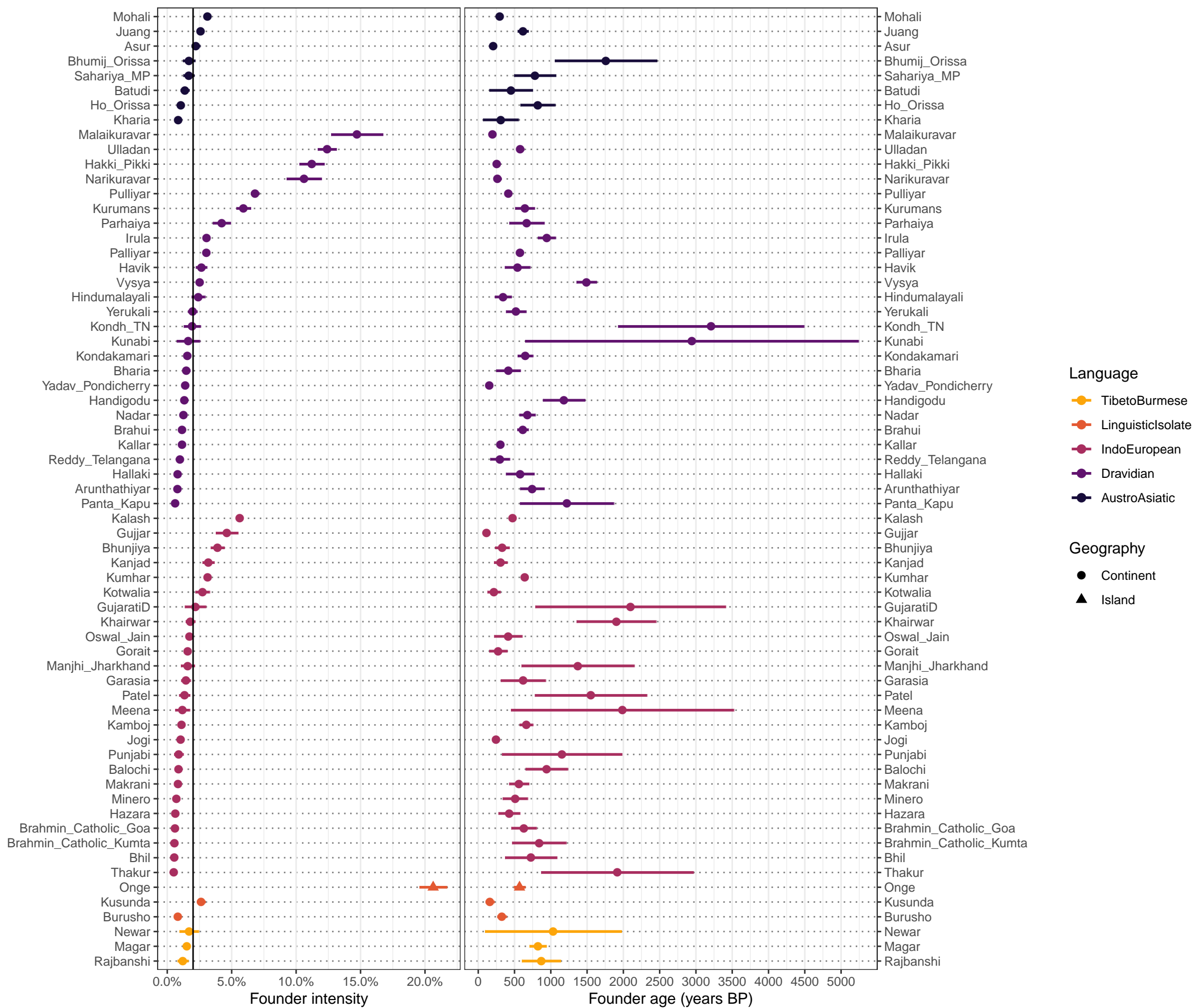

Supplement: S3 Fig — Panel (A) shows the distribution of founder intensity and panel (B) shows the distribution of founder ages for all present-day populations that passed our filtering criteria and showed evidence for a significant founder event in the IndiaHO dataset (see Methods). Each point represents a population and the shape of the points indicates whether the population lives on an island (triangle) or land (circular). The colors represent the linguistic affiliation of the groups. The populations are ordered, first by their linguistic affiliation and then, by decreasing order of estimated founder intensity, from top to bottom. (A) Distribution of the estimated founder intensities. We show the founder intensities (points) and their associated 95% confidence intervals. The black horizontal line shows the estimated founder intensity in the Ashkenazi Jew population from the IndiaHO dataset (2.0%). (B) Distribution of the estimated founder ages. We show the estimated founder ages and their associated 95% confidence intervals. The estimated ages were converted from generations to years assuming an average generation time of 28 years [29,30]. (PDF) [file pgen.1010243.s003.pdf]

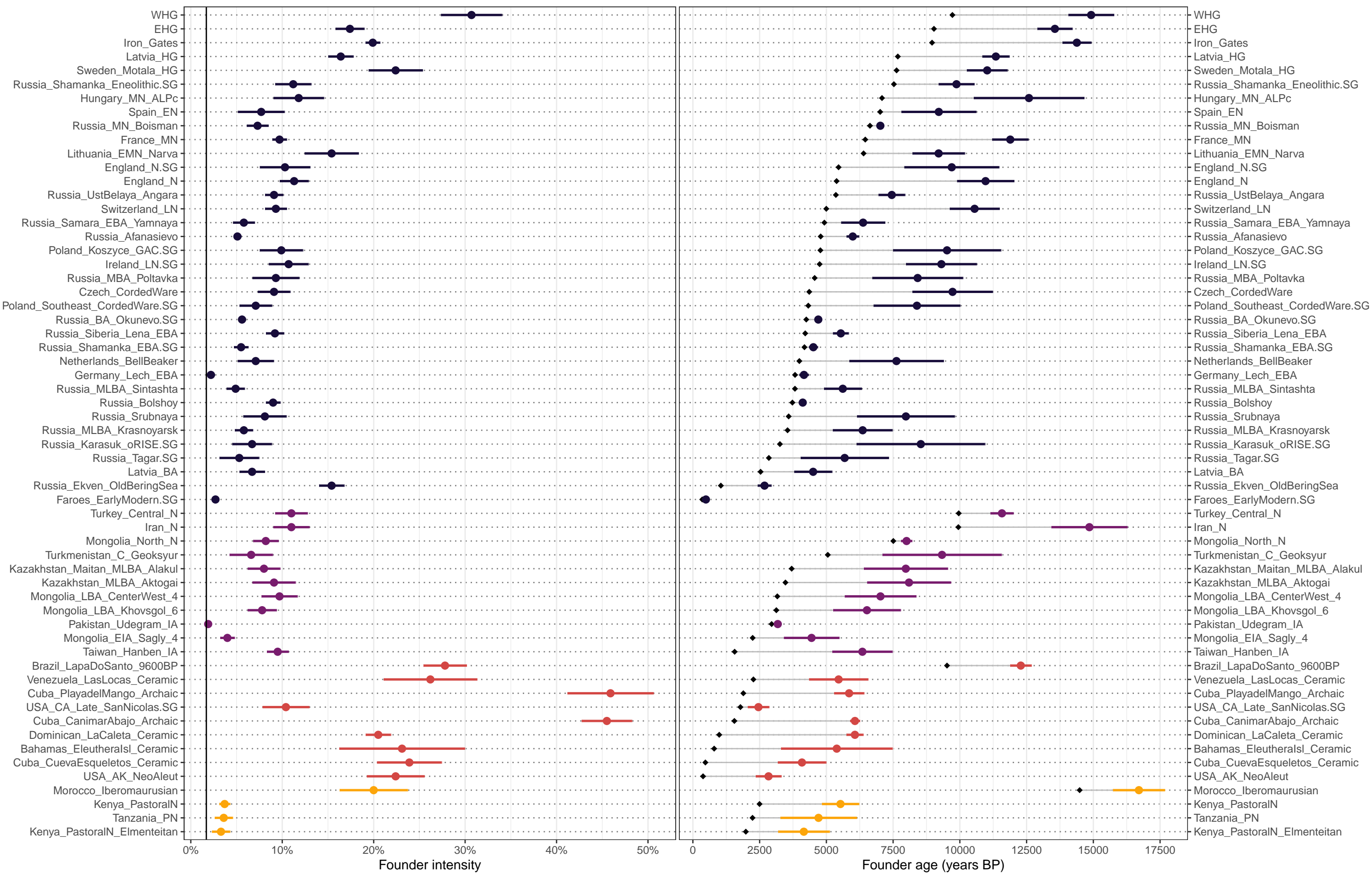

Supplement: S5 Fig — Panel (A) shows the distribution of founder intensity, and panel (B) shows the distribution of founder ages for all ancient populations that passed our filtering criteria in the HO44 dataset and showed evidence for a significant founder event (see Methods). Each point represents a population and the color of the points indicates the geographical (continent) location of the population. The populations are ordered, first by their continent and then, in increasing order of radiocarbon sample age, from top to bottom. (A) Distribution of the estimated founder intensities. We show the mean founder intensities (points) and their associated 95% confidence intervals. The black horizontal line shows the inferred founder intensity in the present-day Ashkenazi Jewish population from the HO37 dataset (1.7%, CI95: [1.3%–2.1%]). (B) Distribution of the estimated founder ages. We show the estimated founder ages and their associated 95% confidence intervals. The estimated ages were converted from generations to years before present by using a generation time of 28 years [29,30] and by adding the radiocarbon sample date (black diamond-shaped point) of the specimens. (PDF) [file pgen.1010243.s005.pdf]

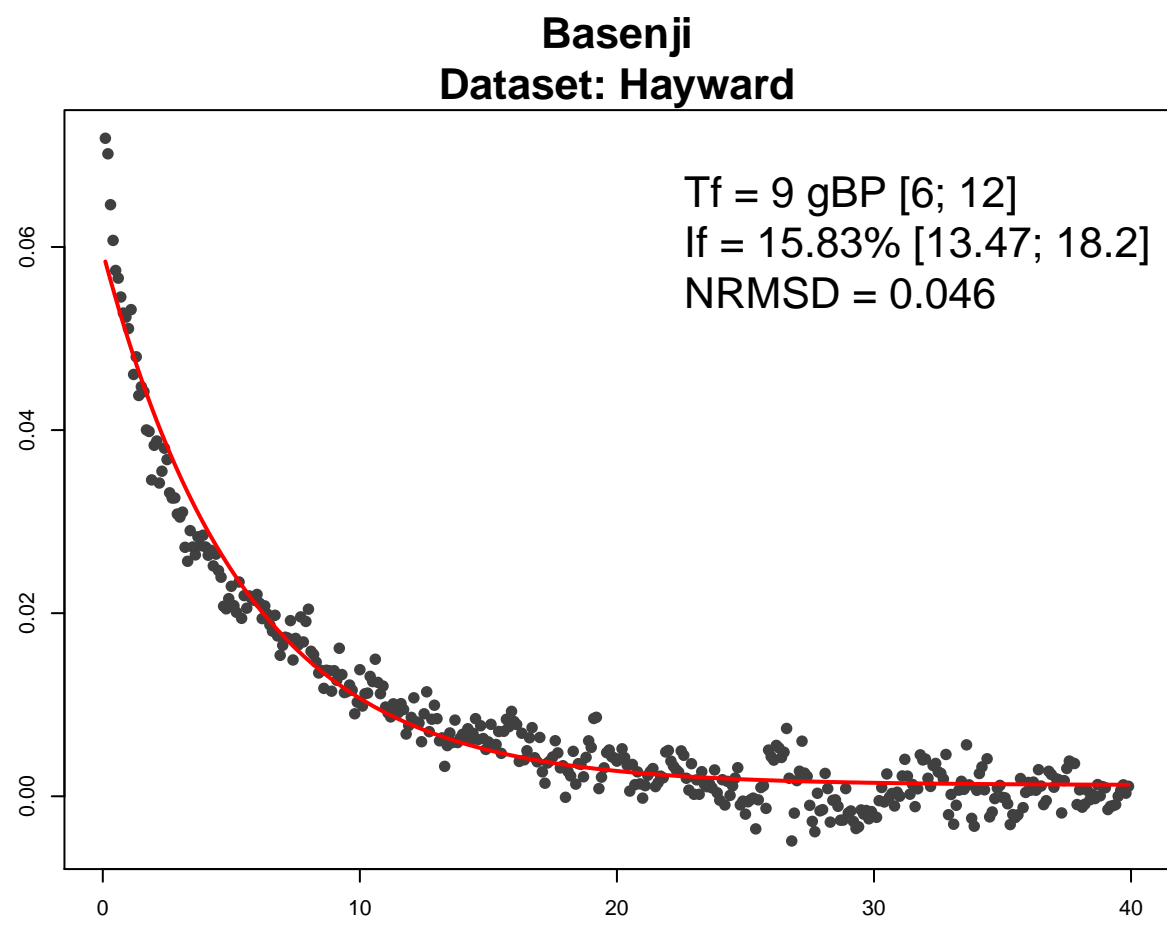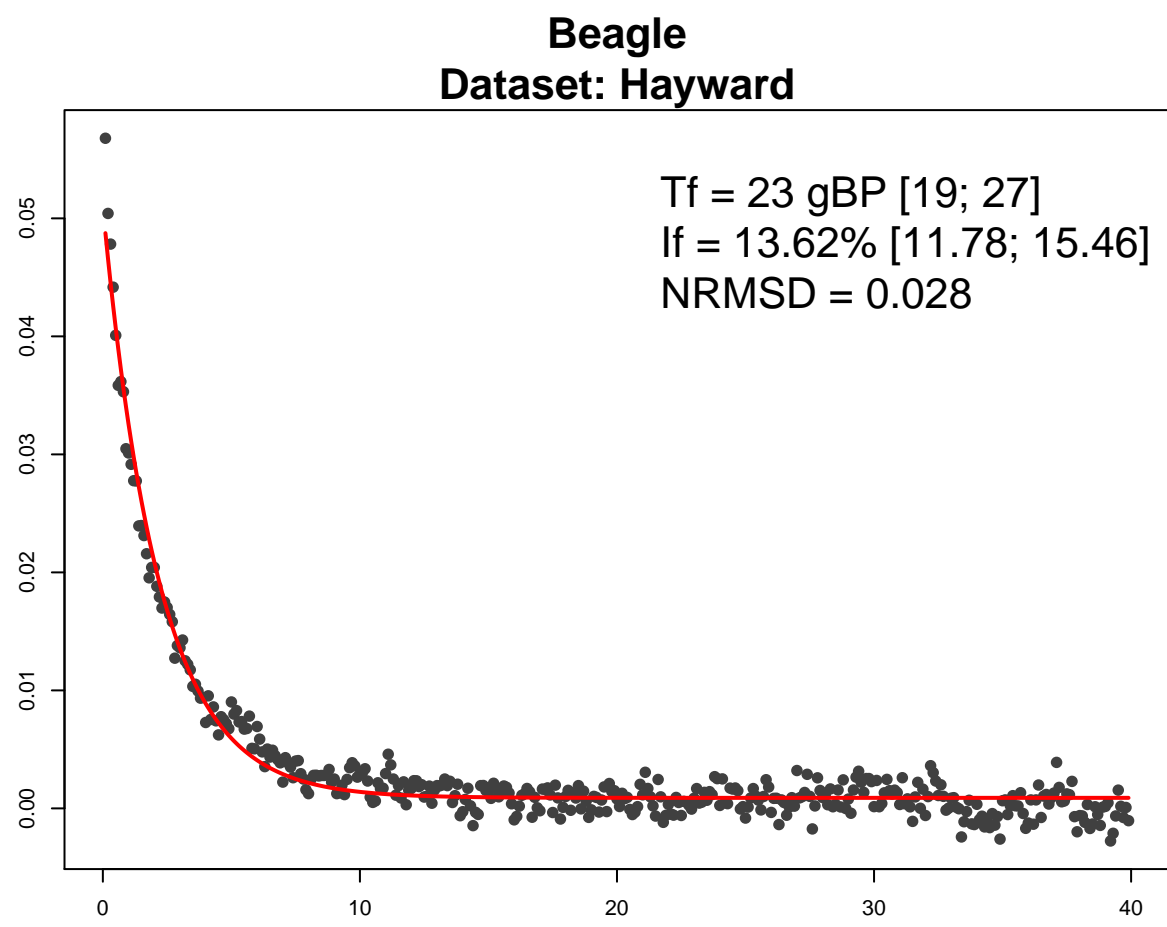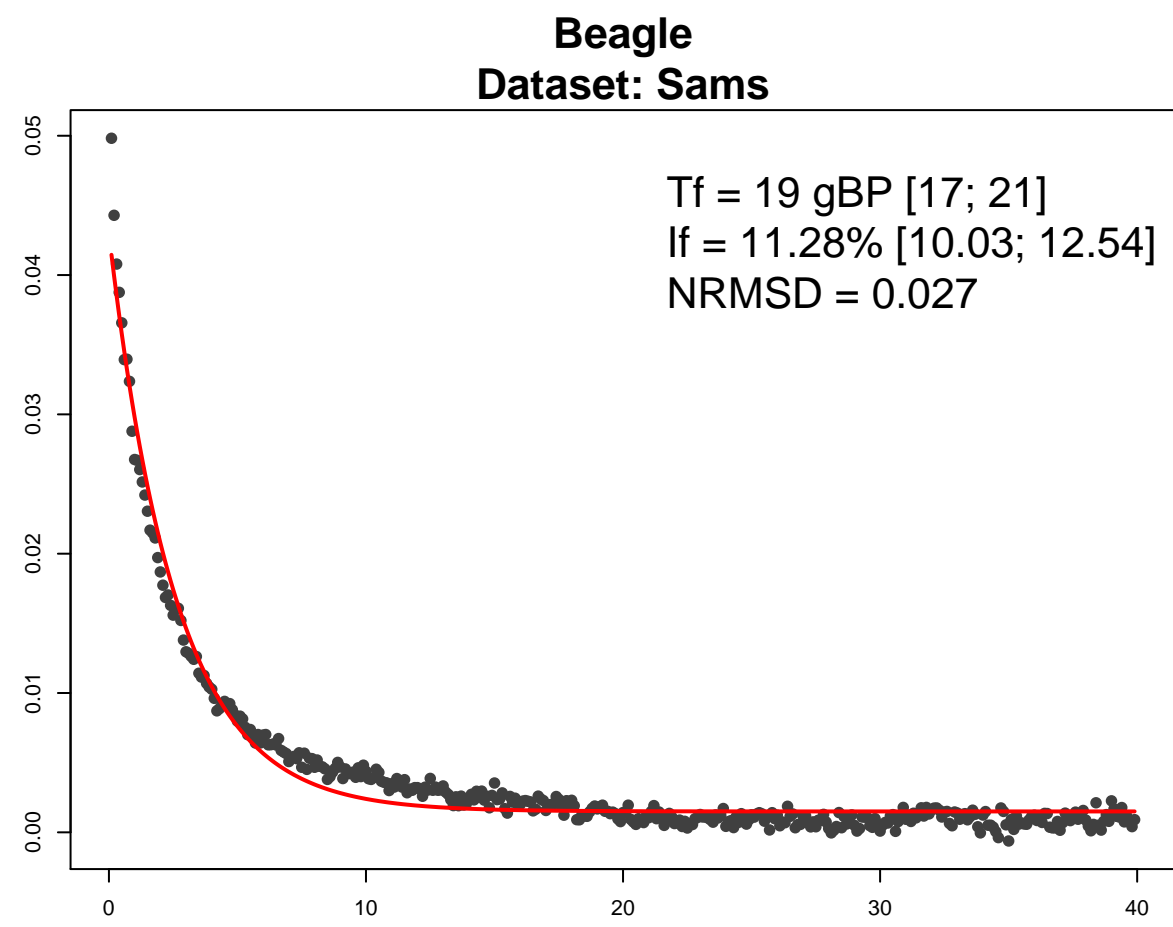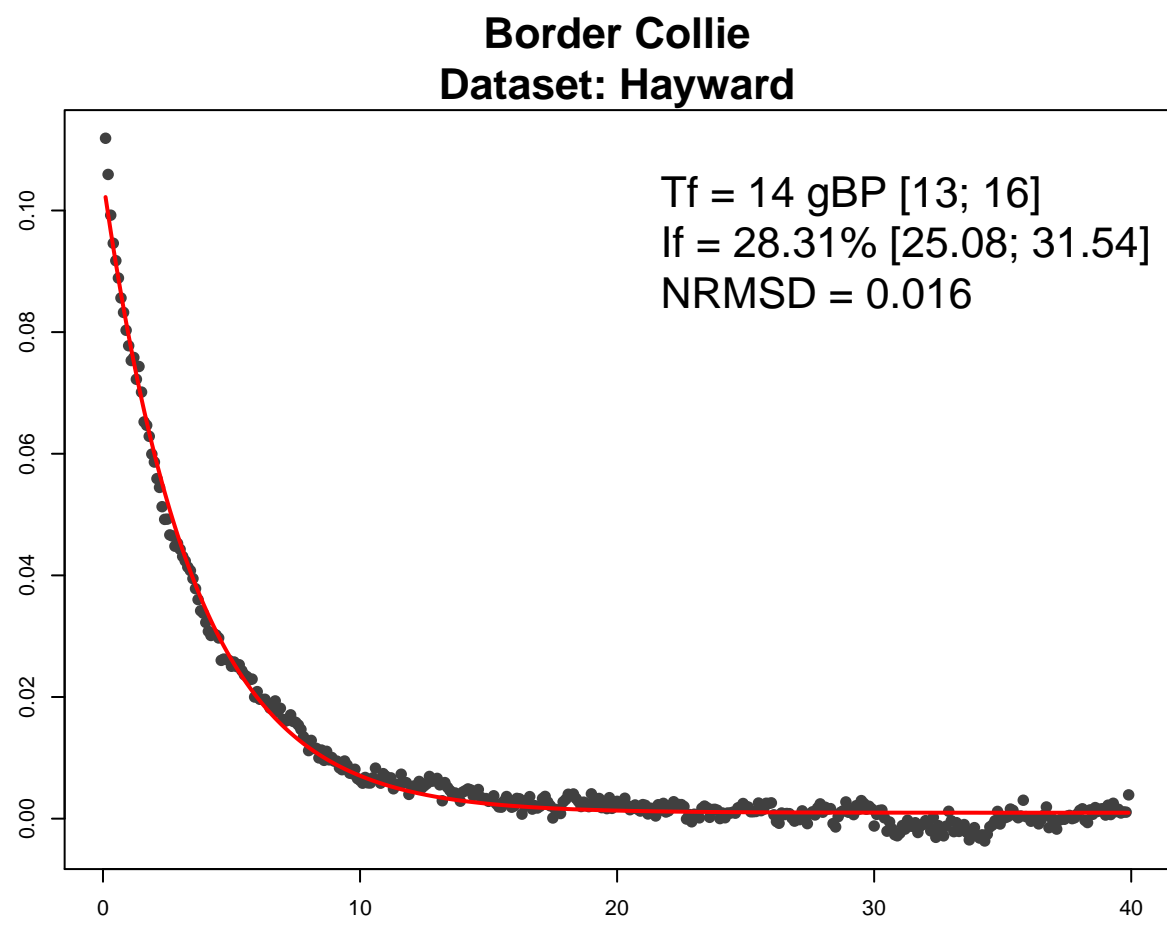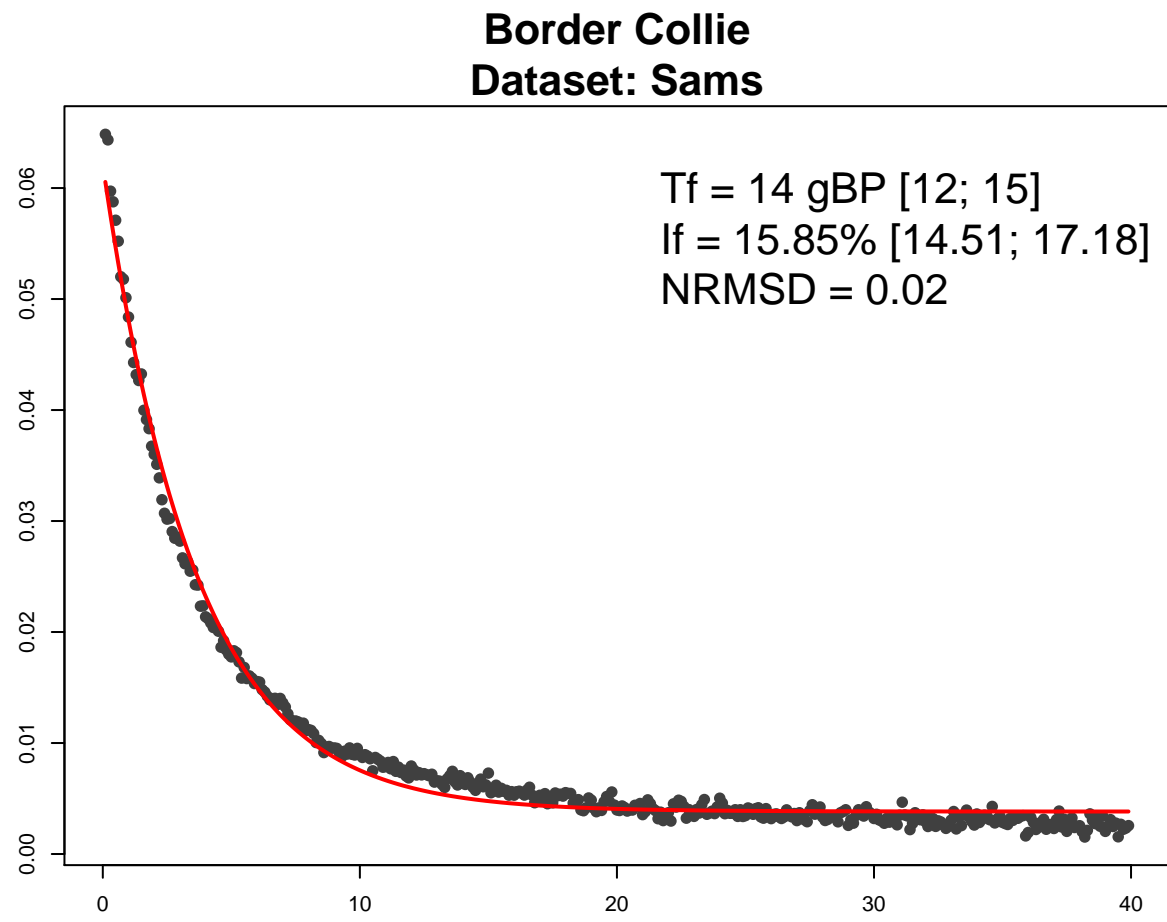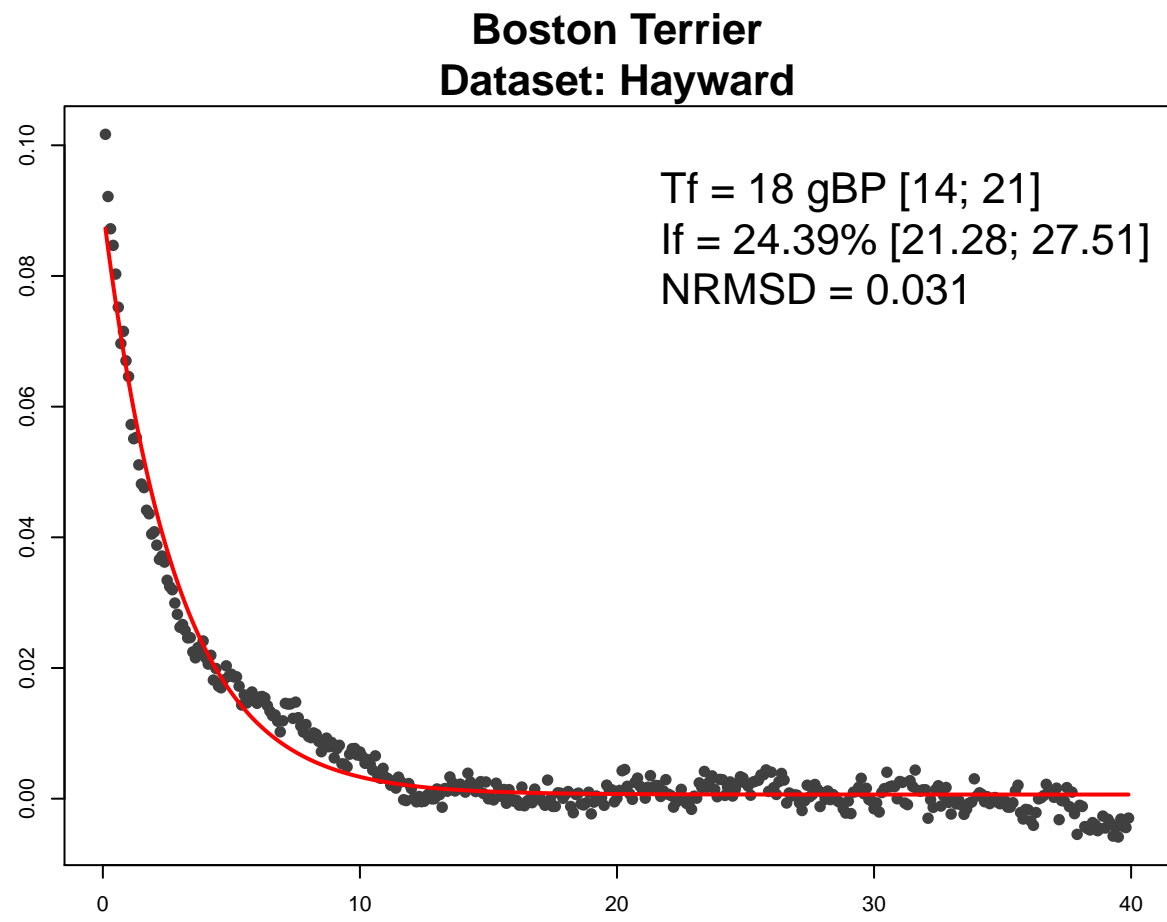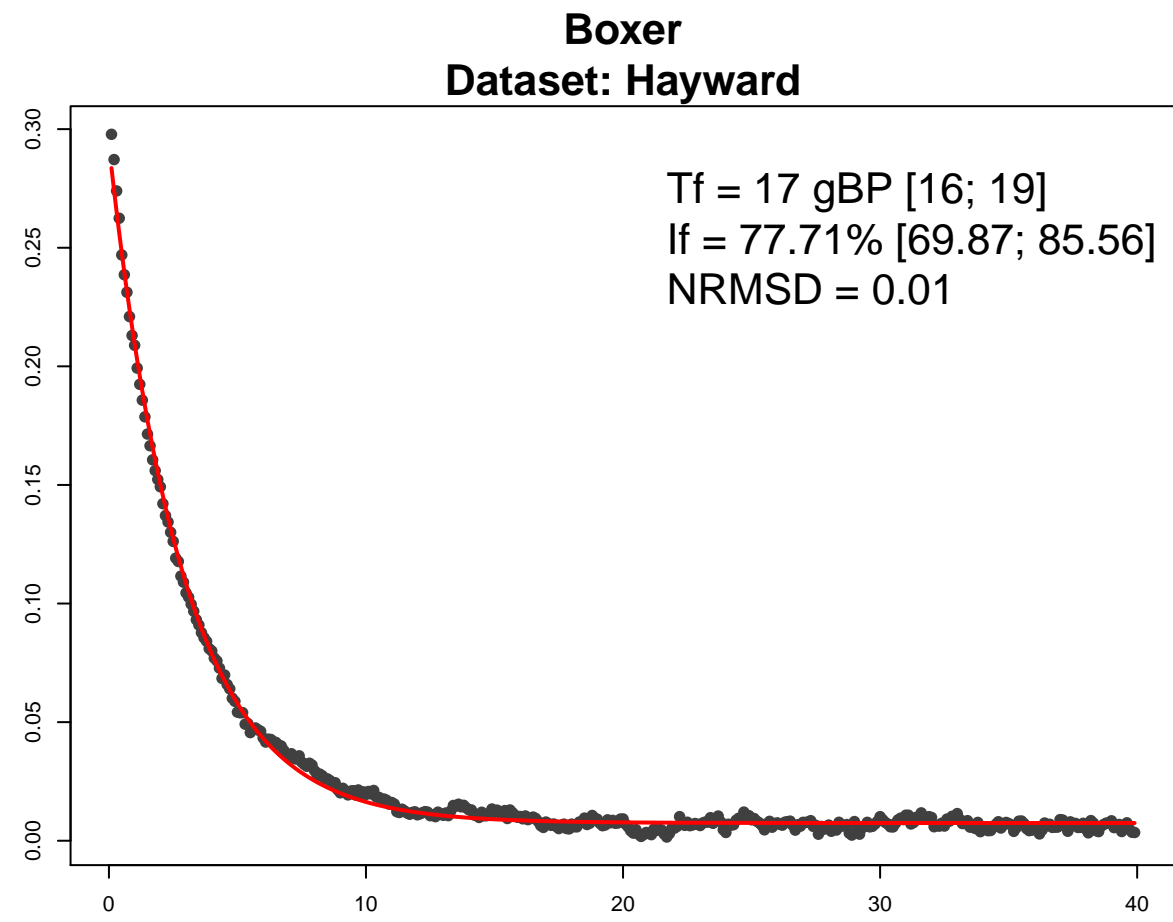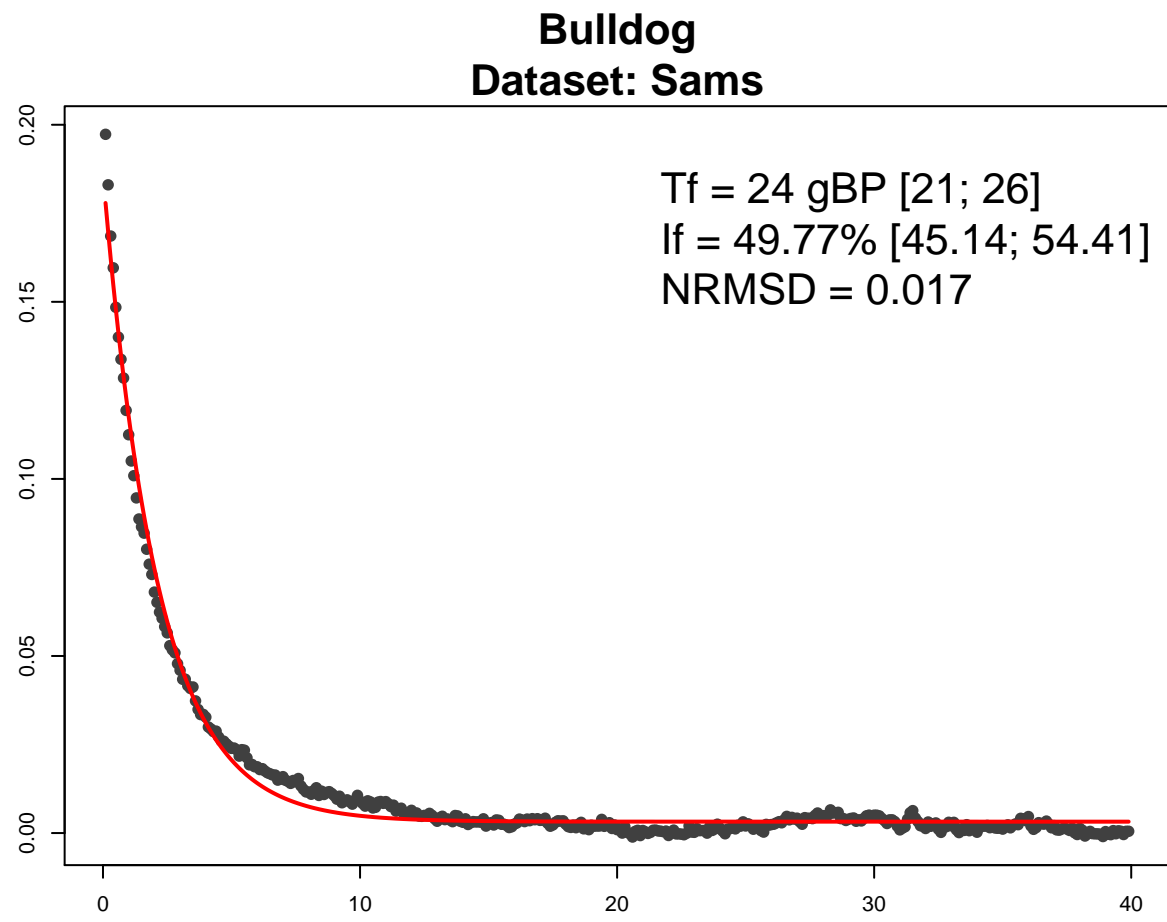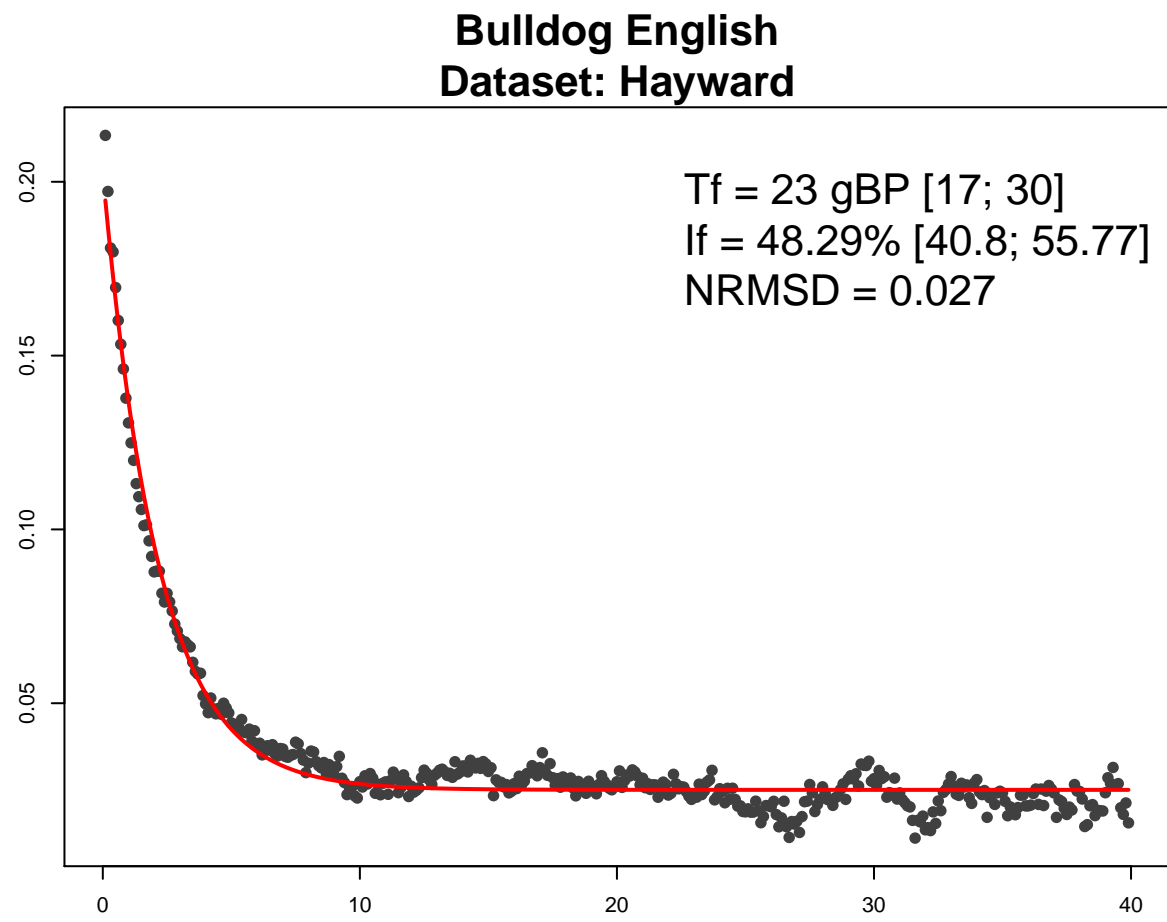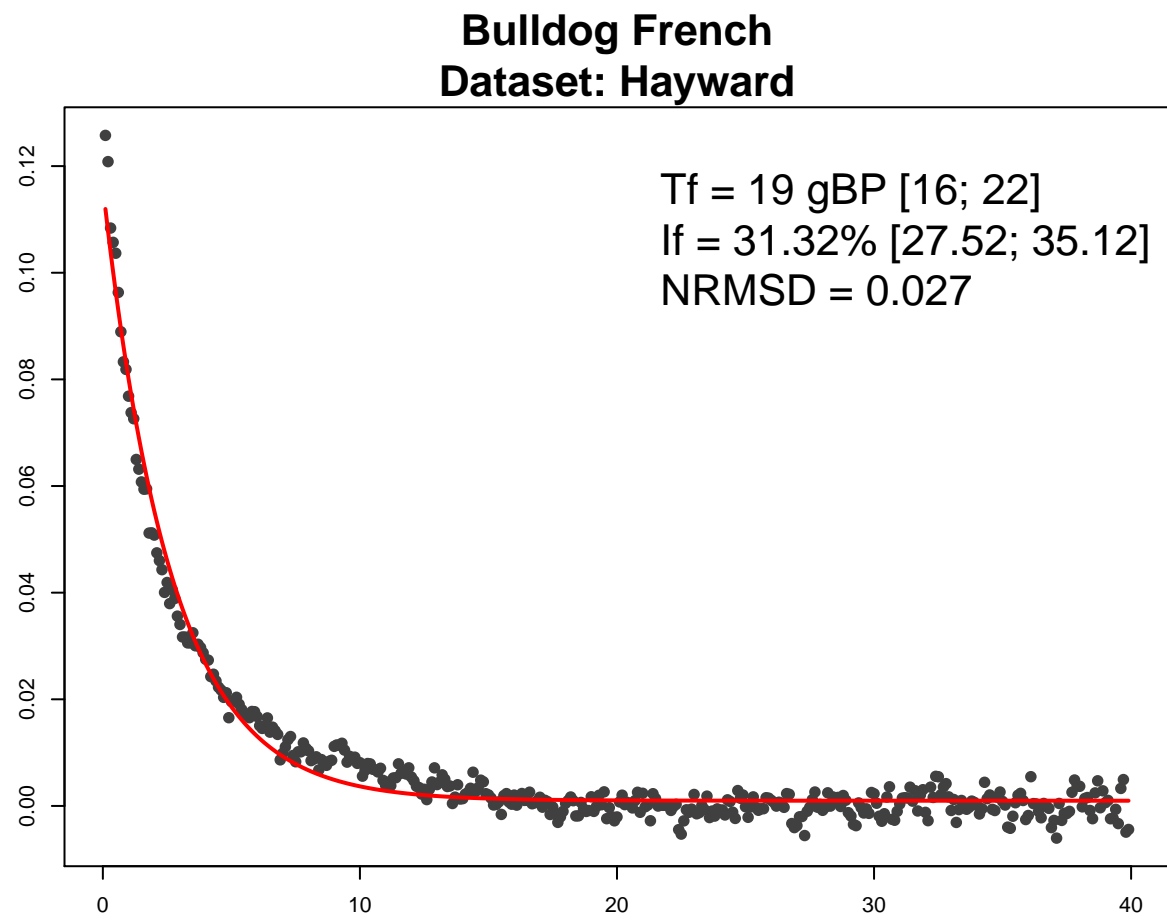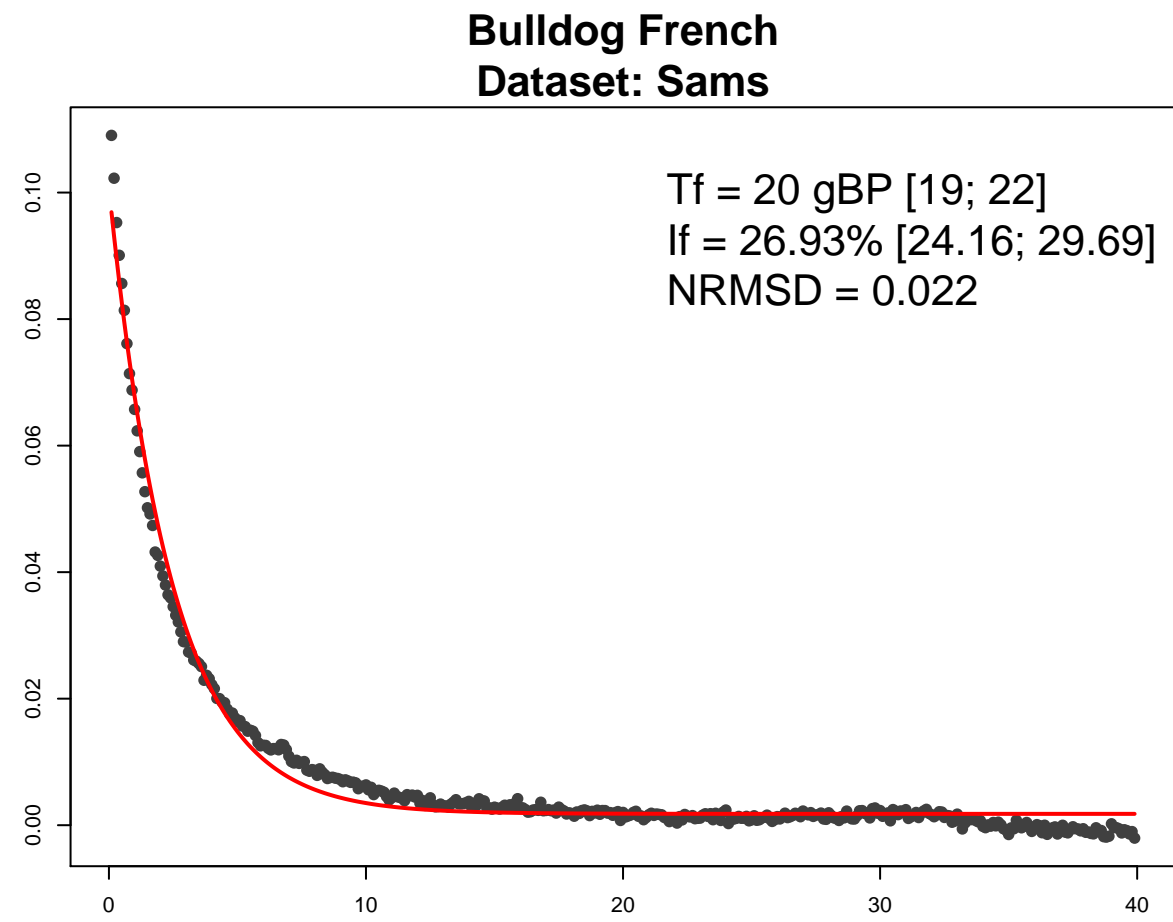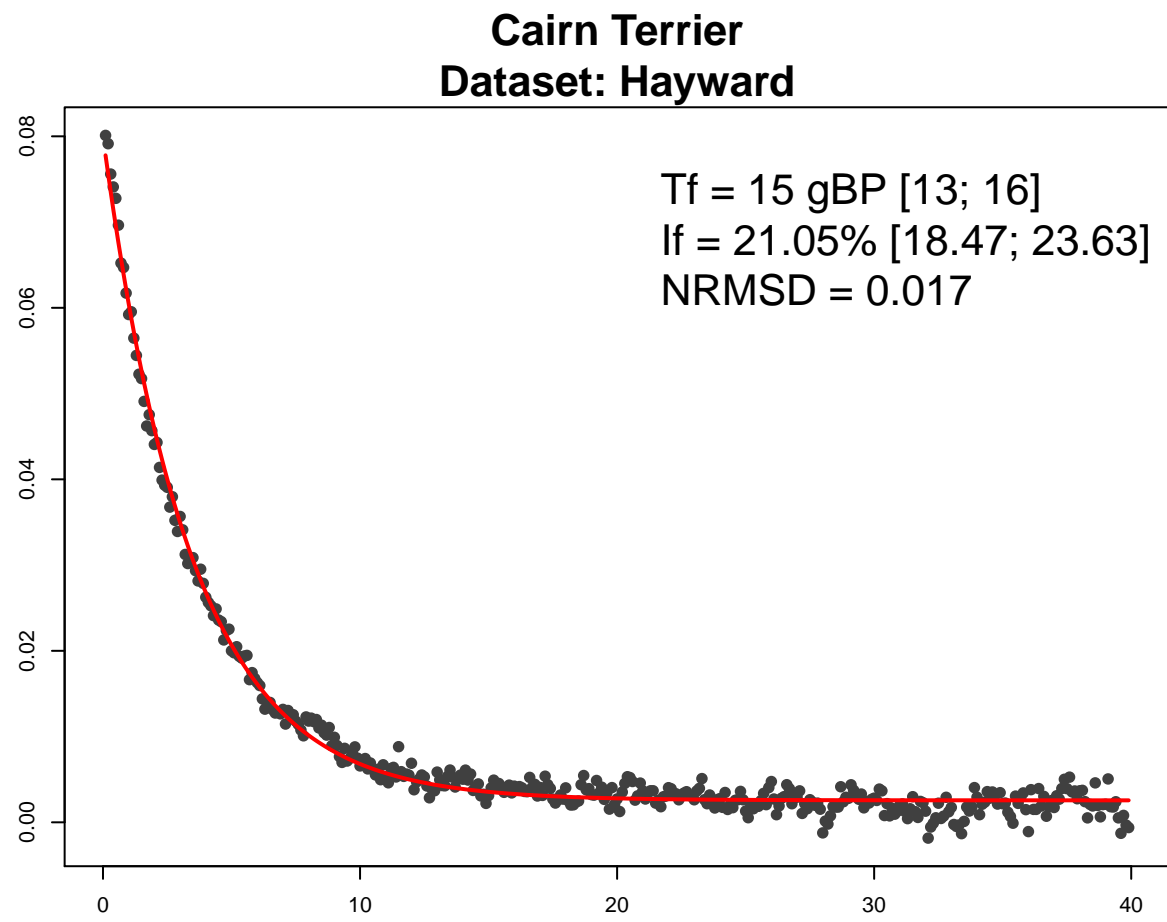

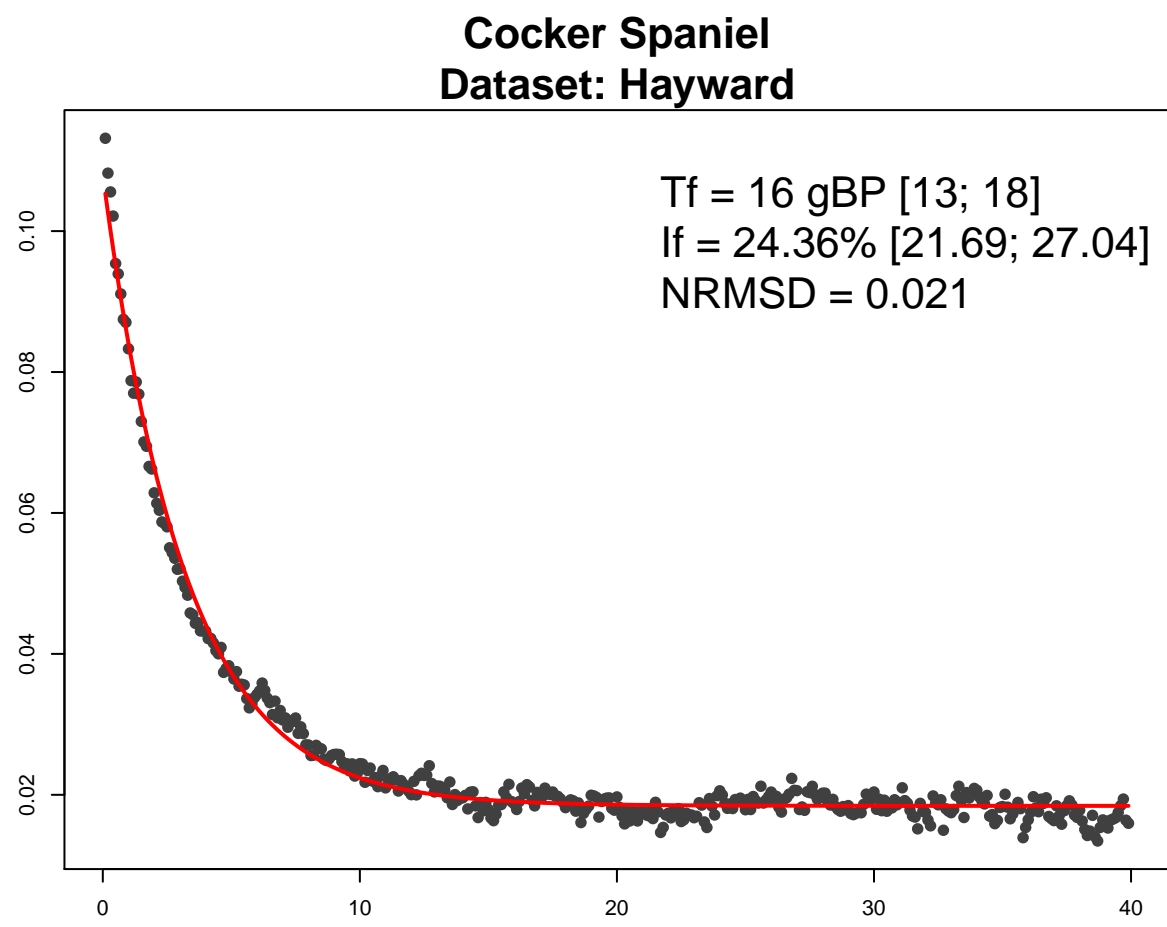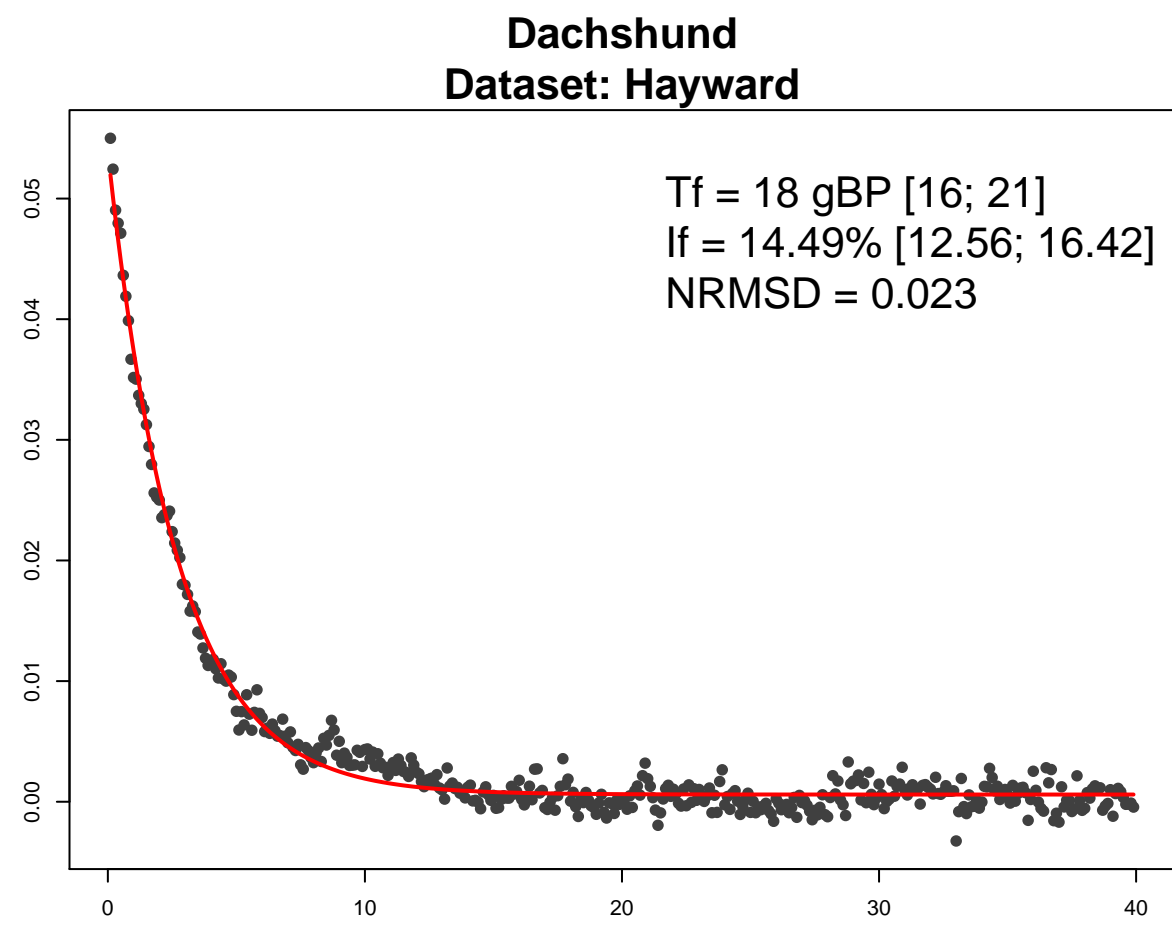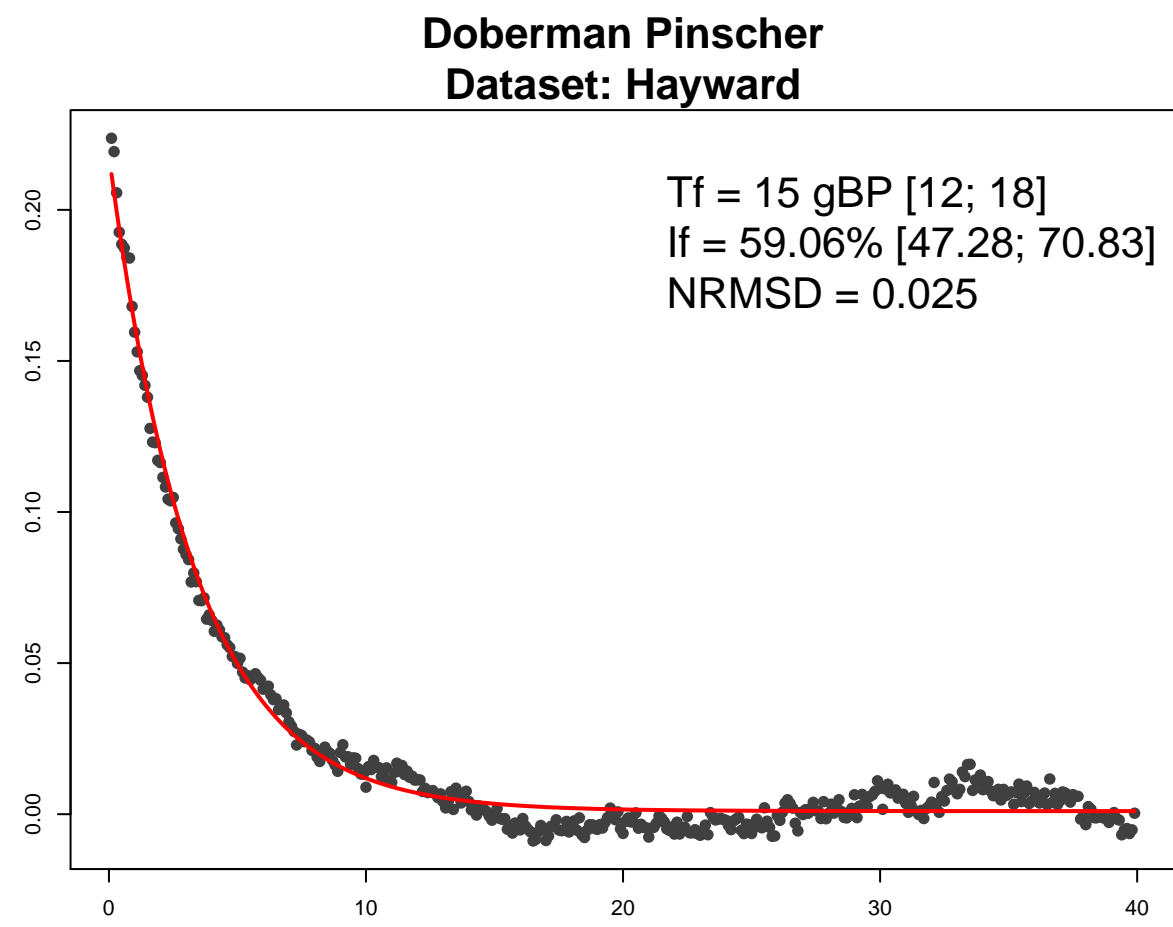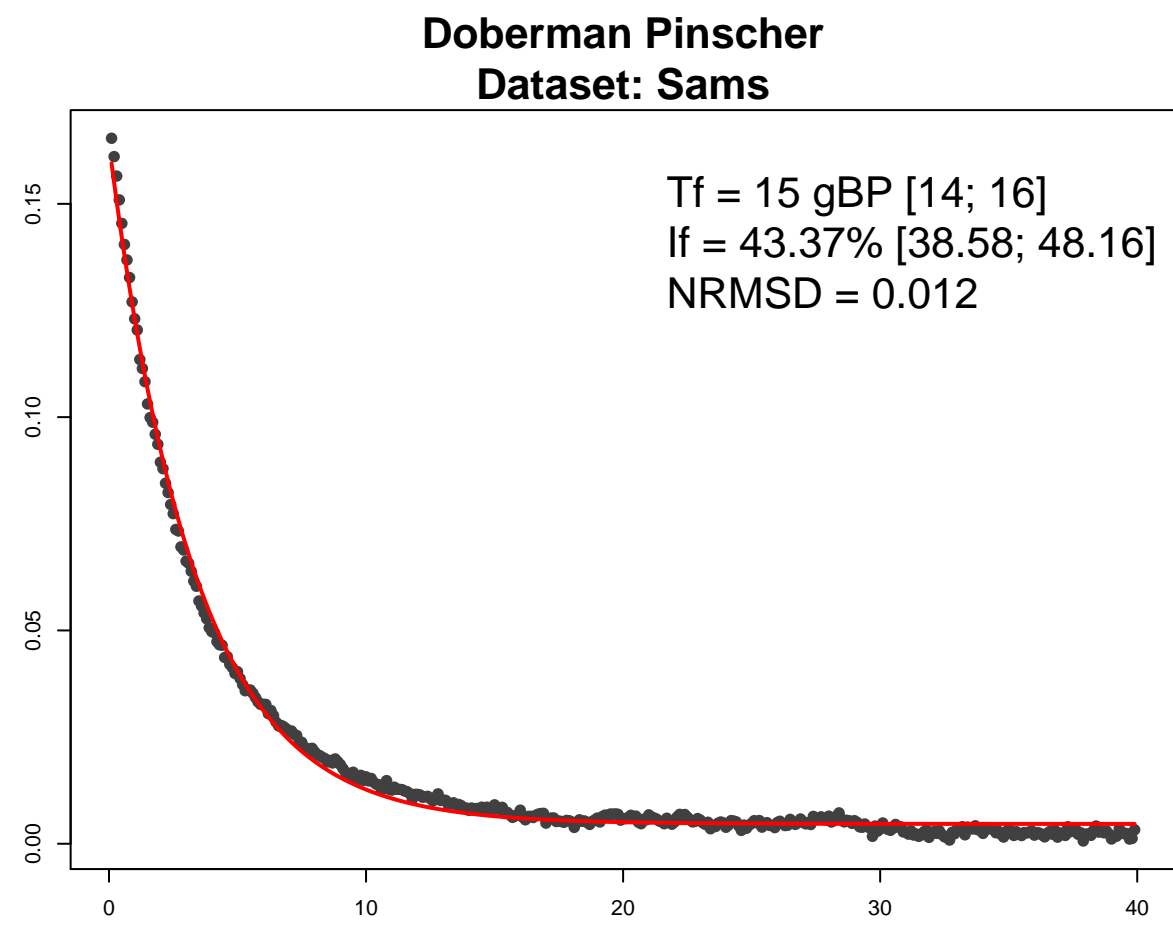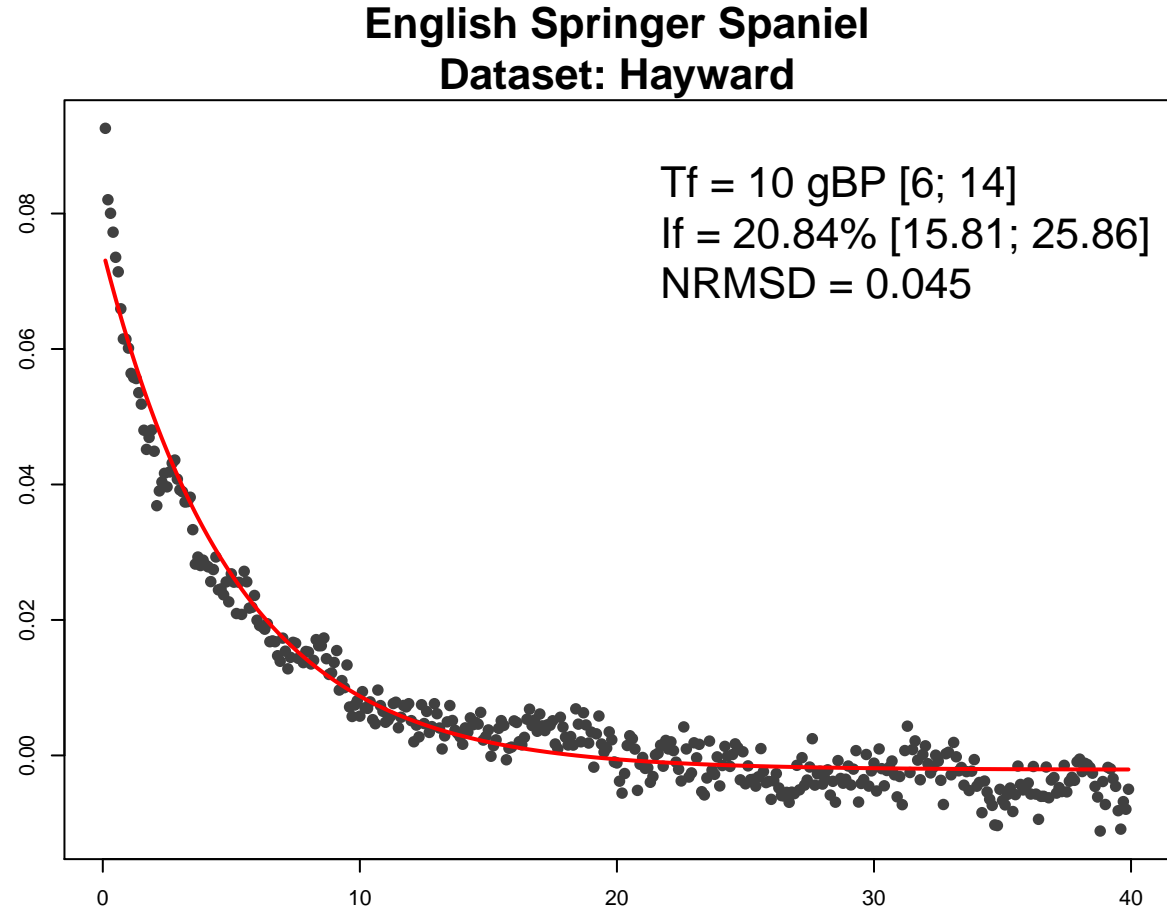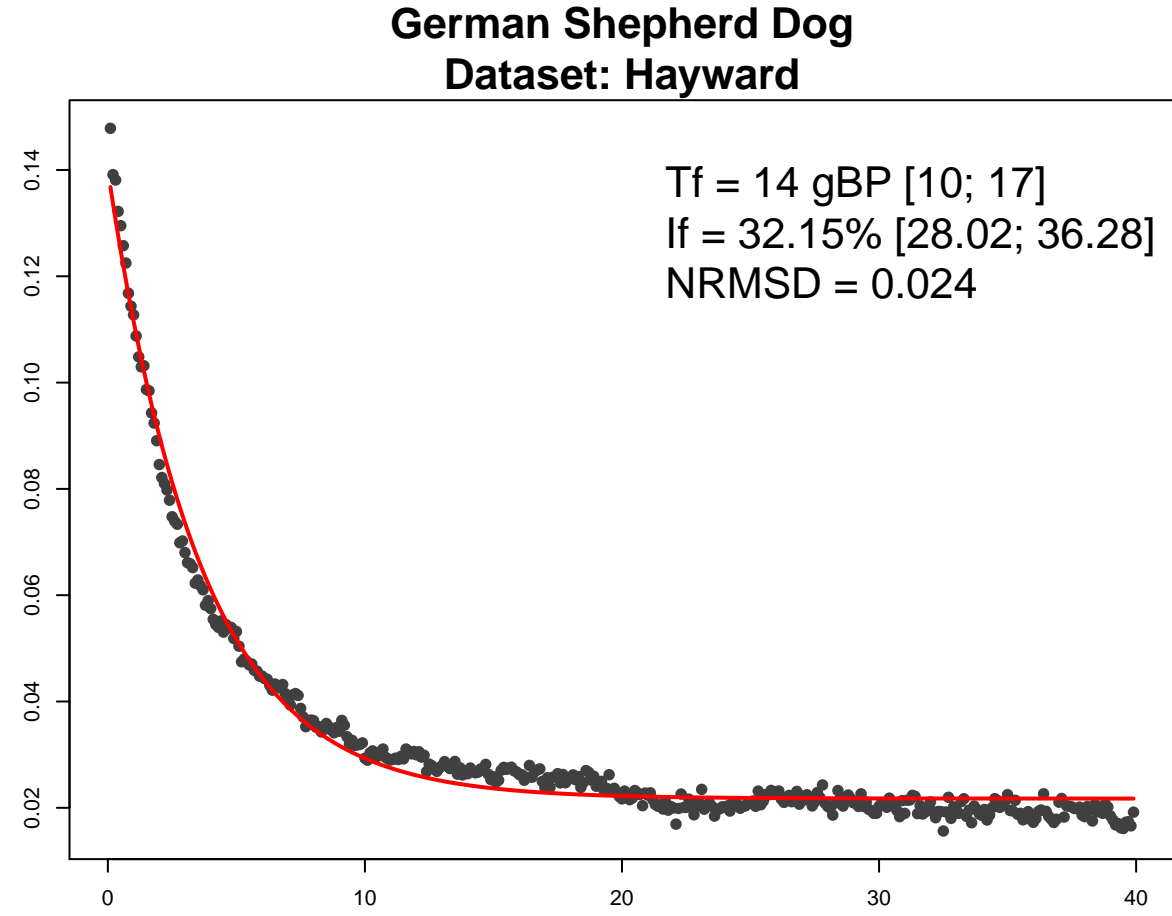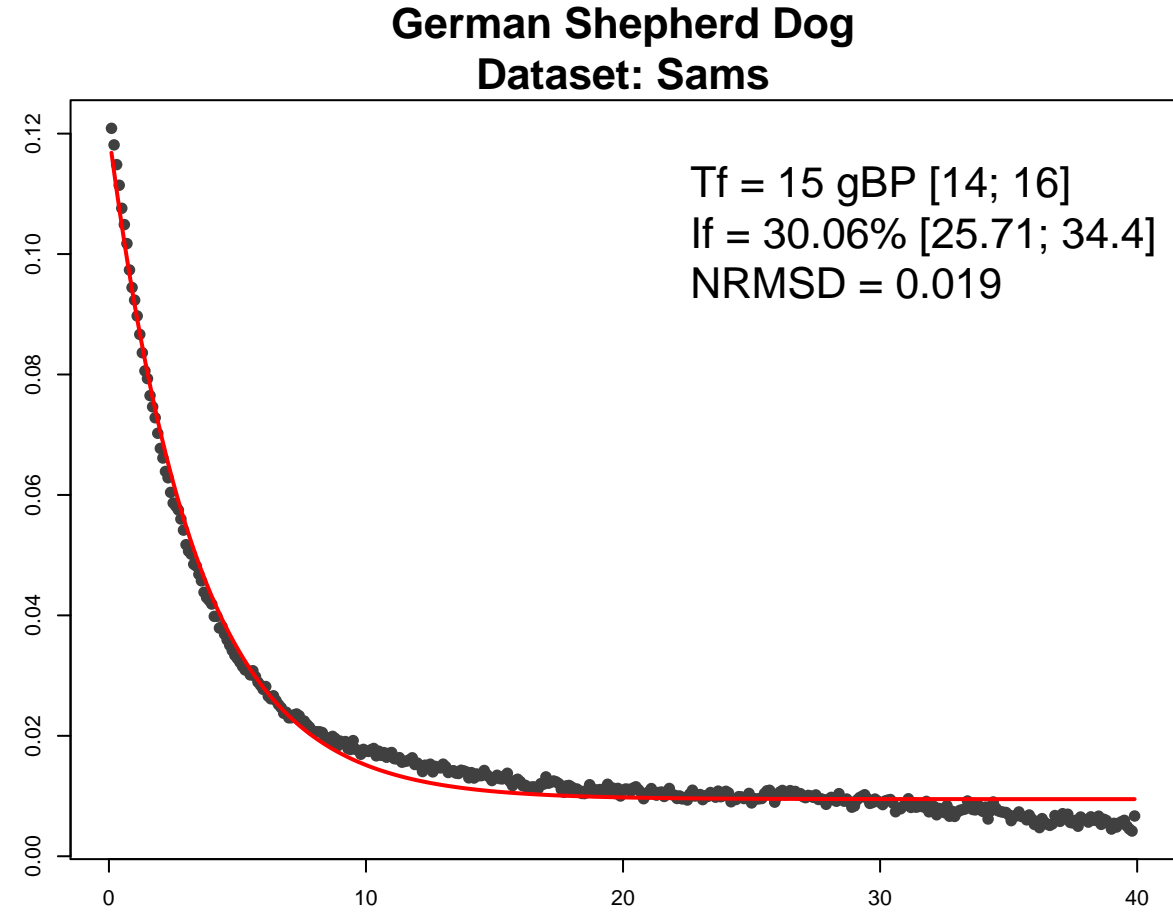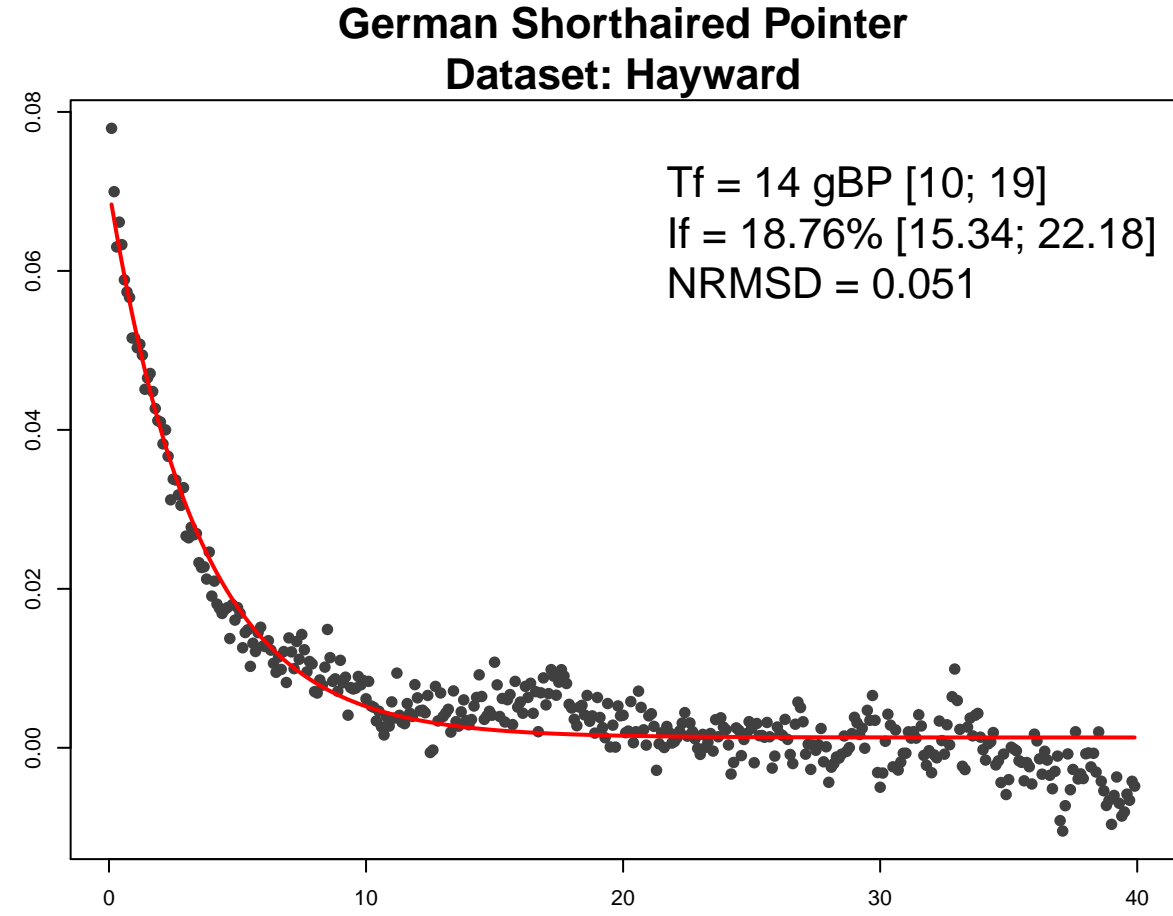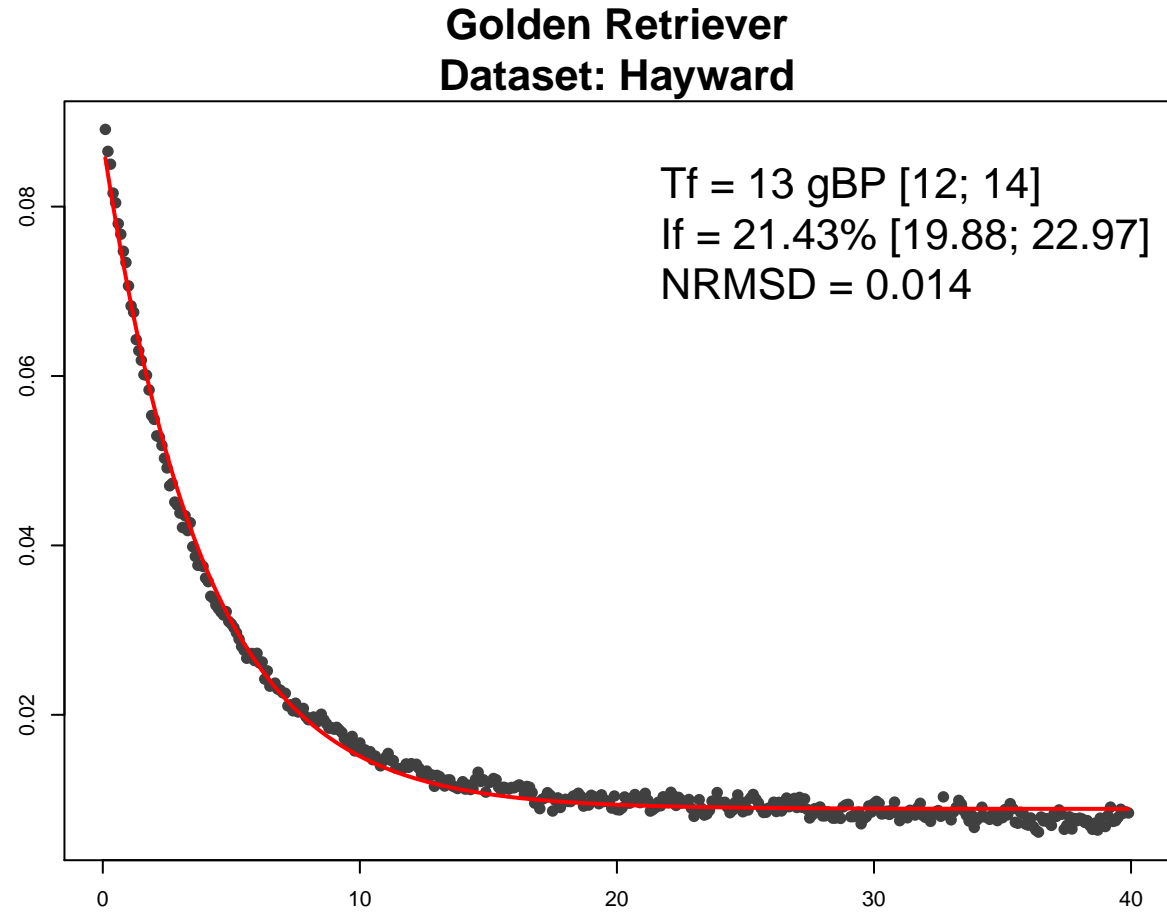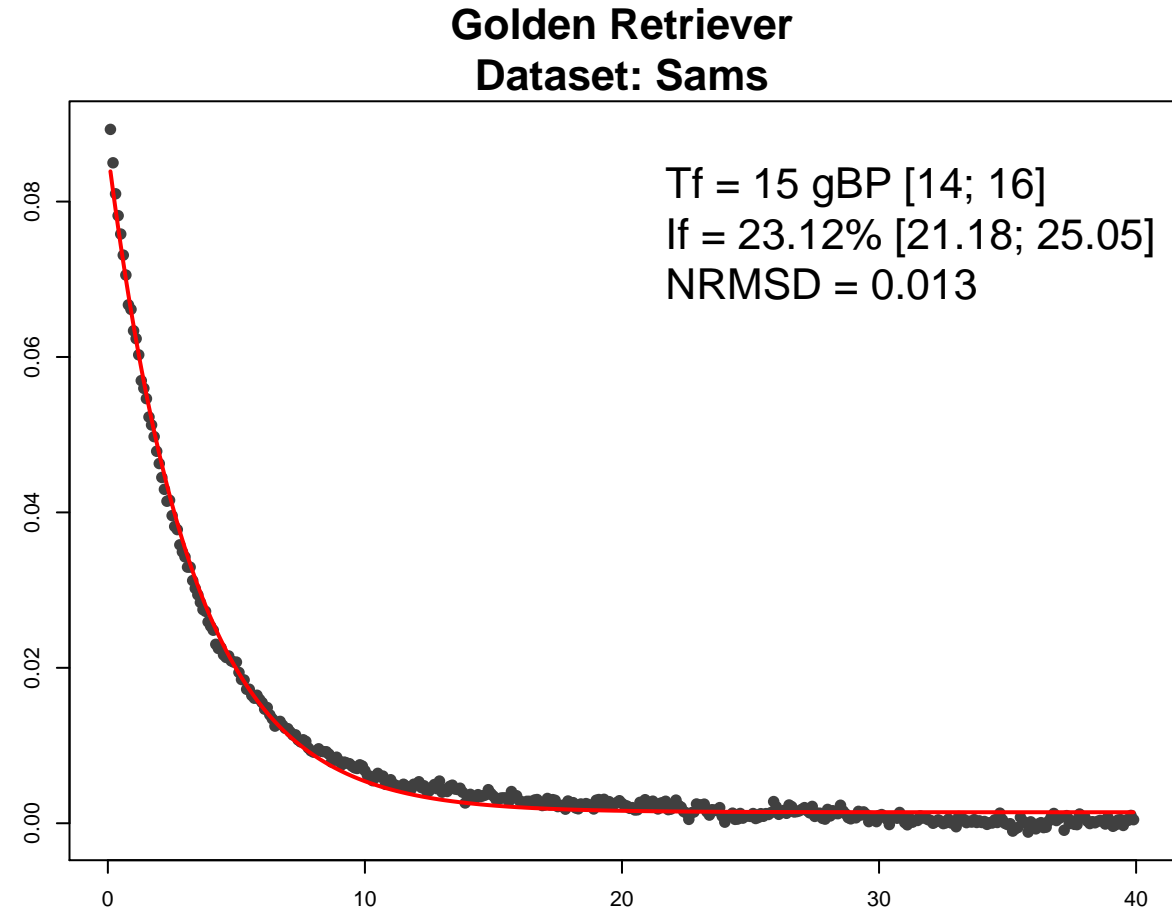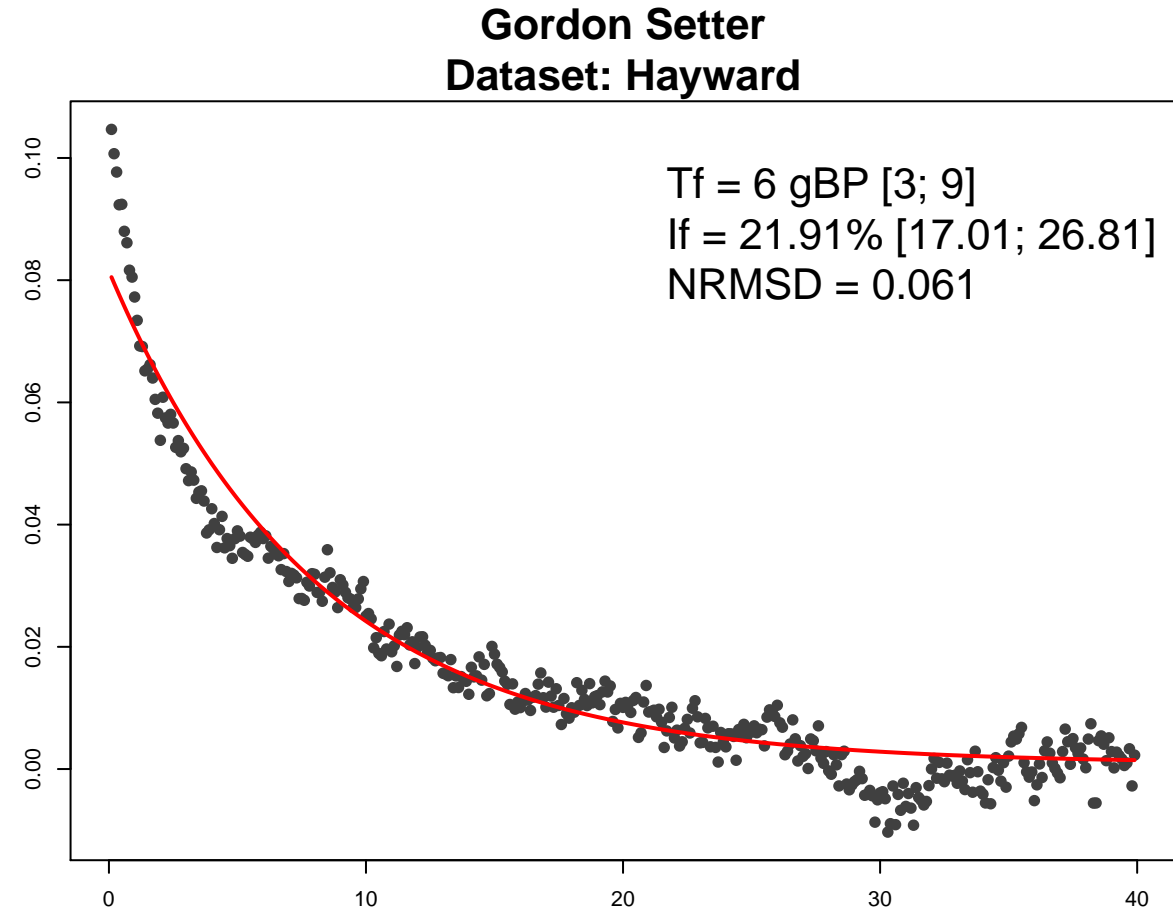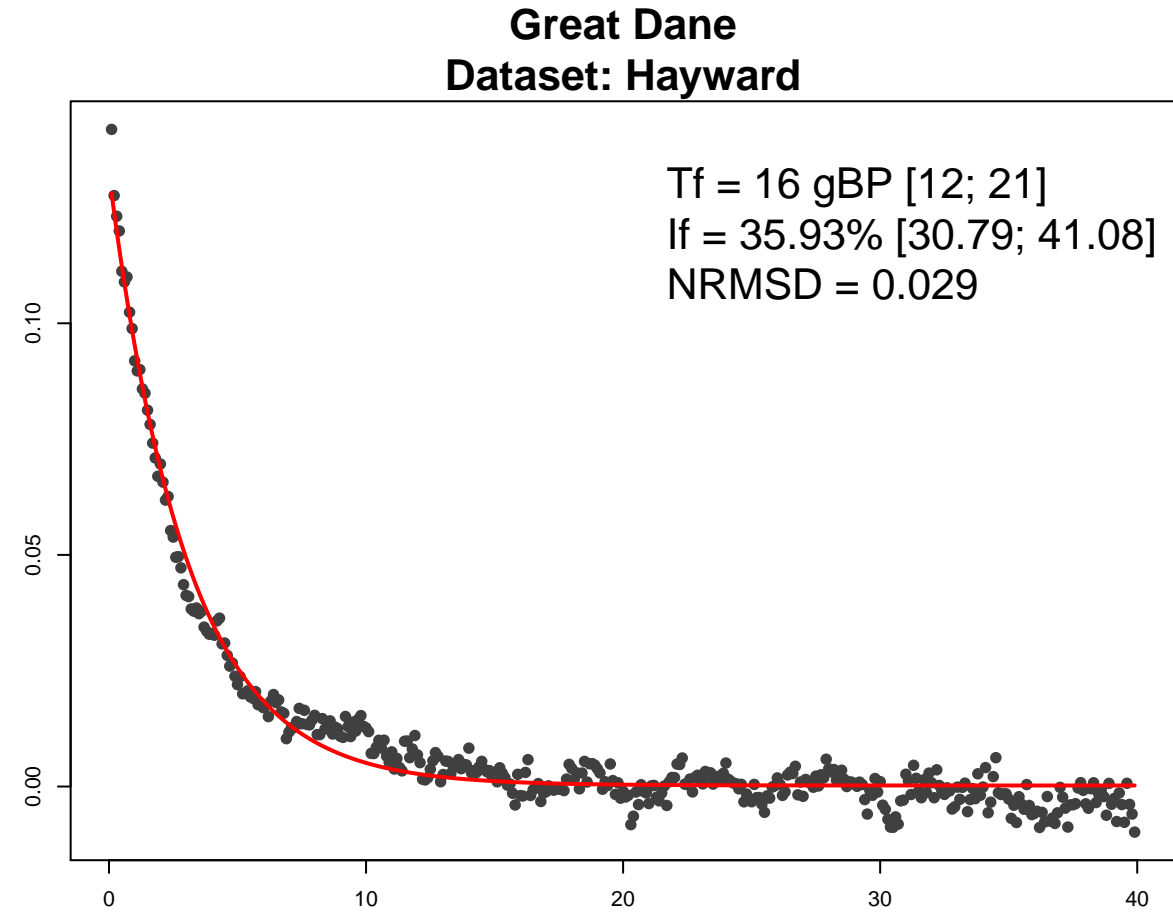

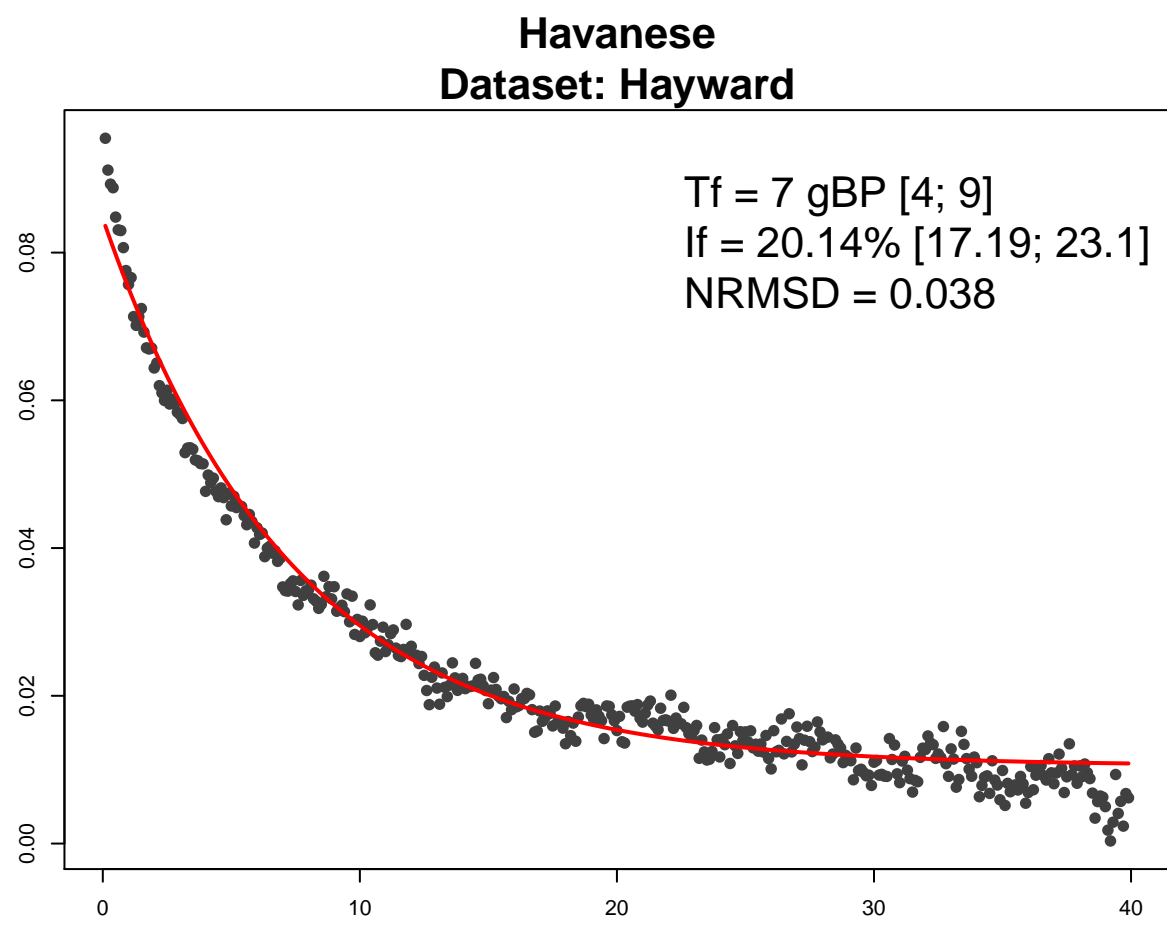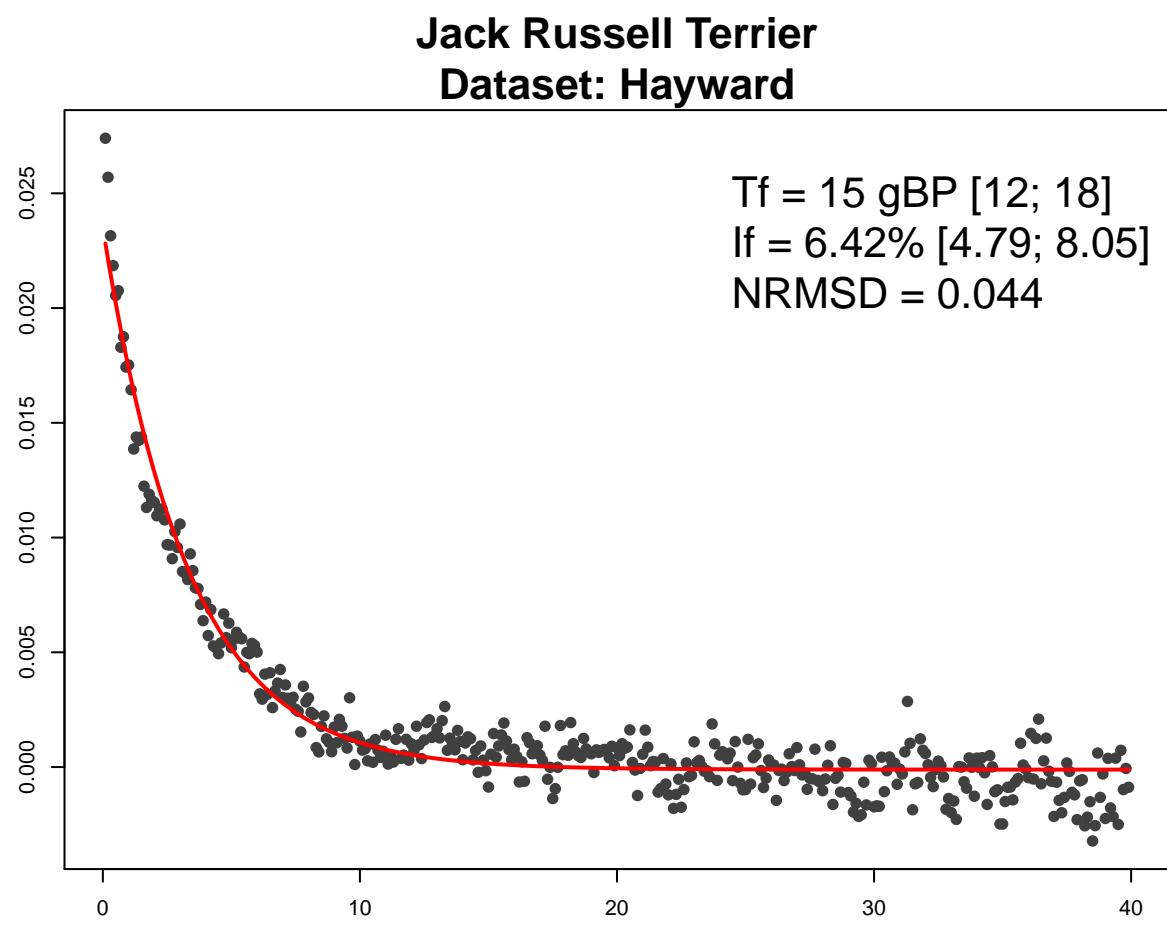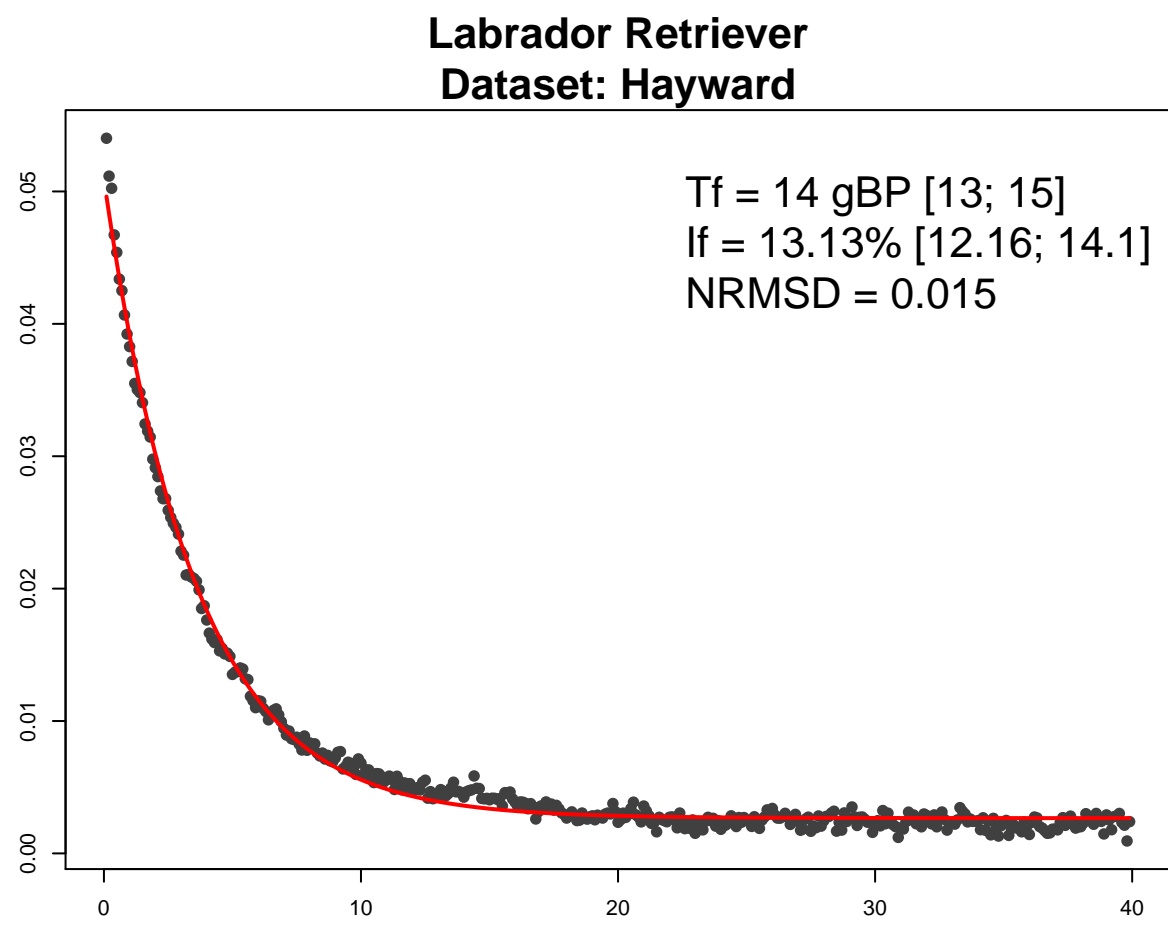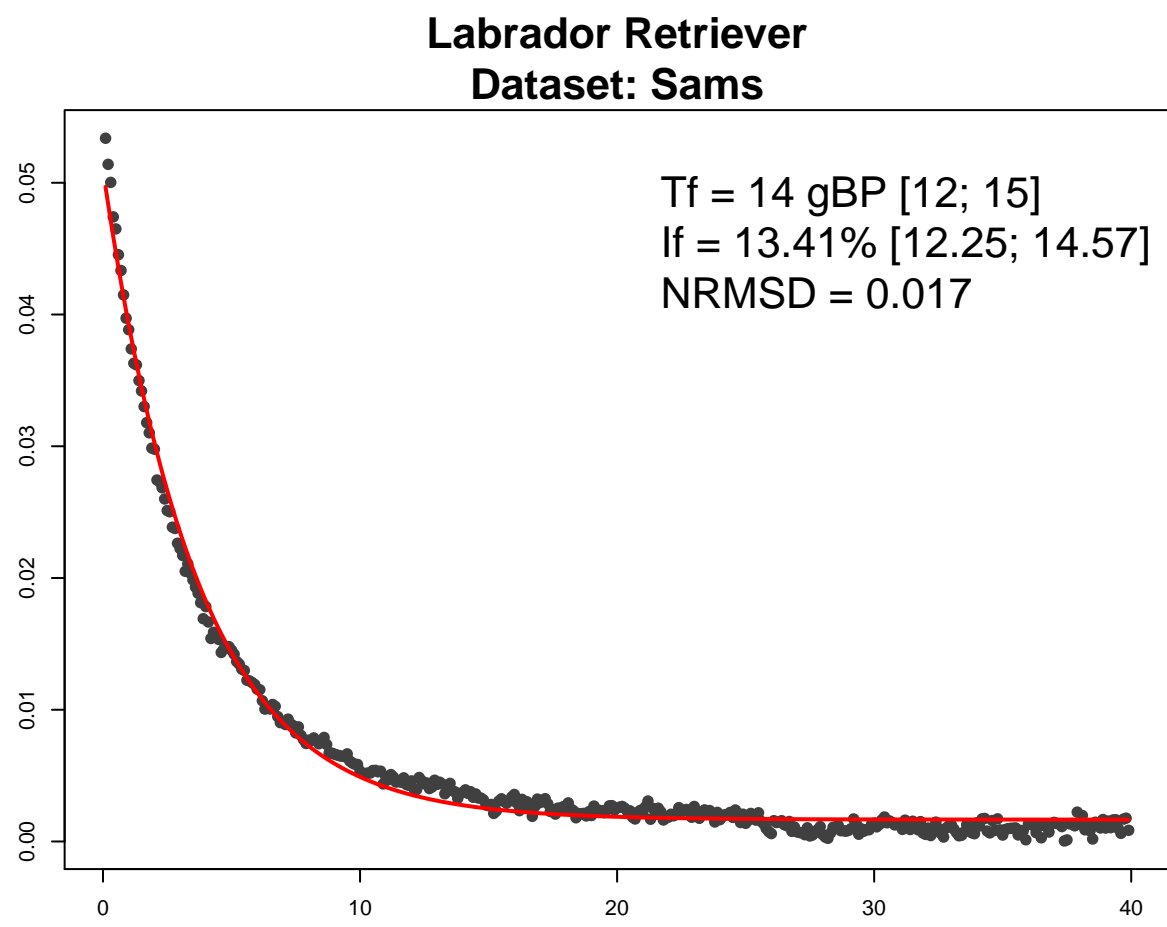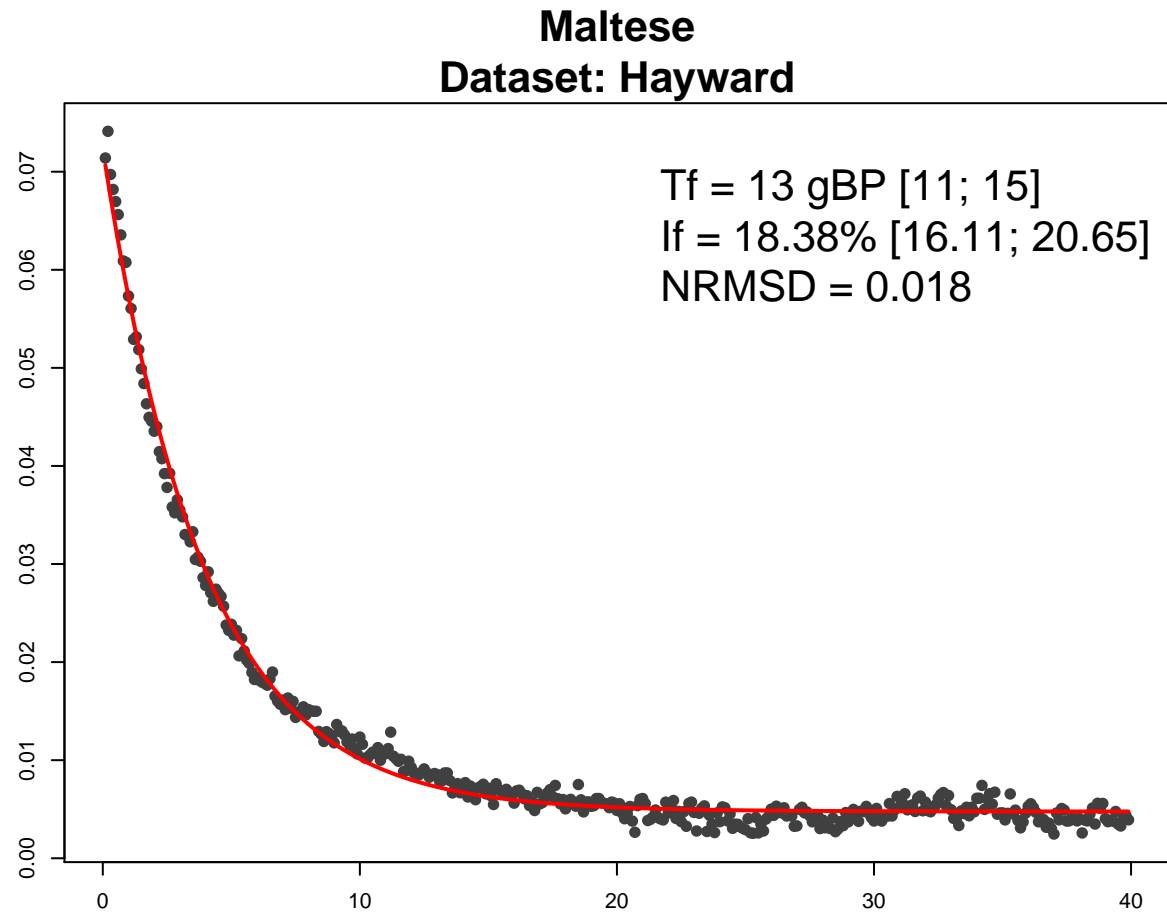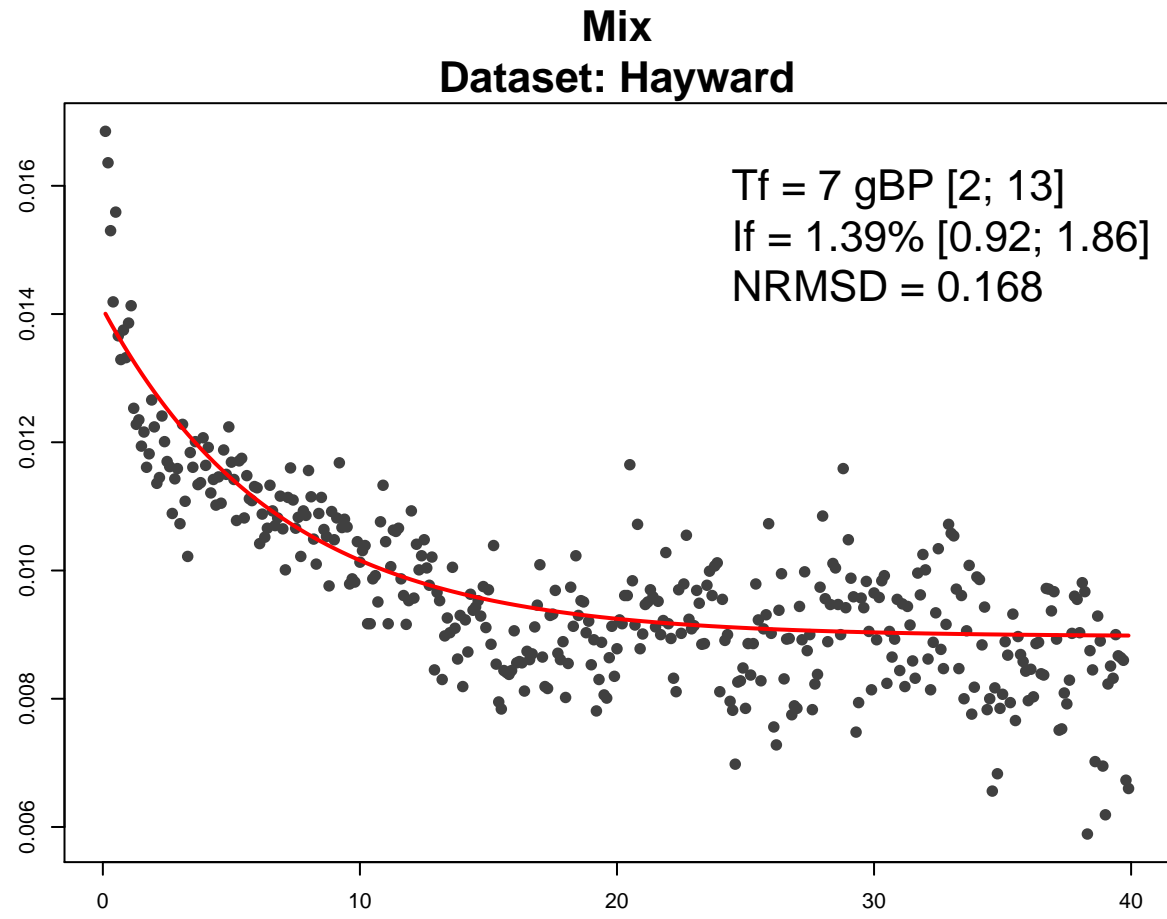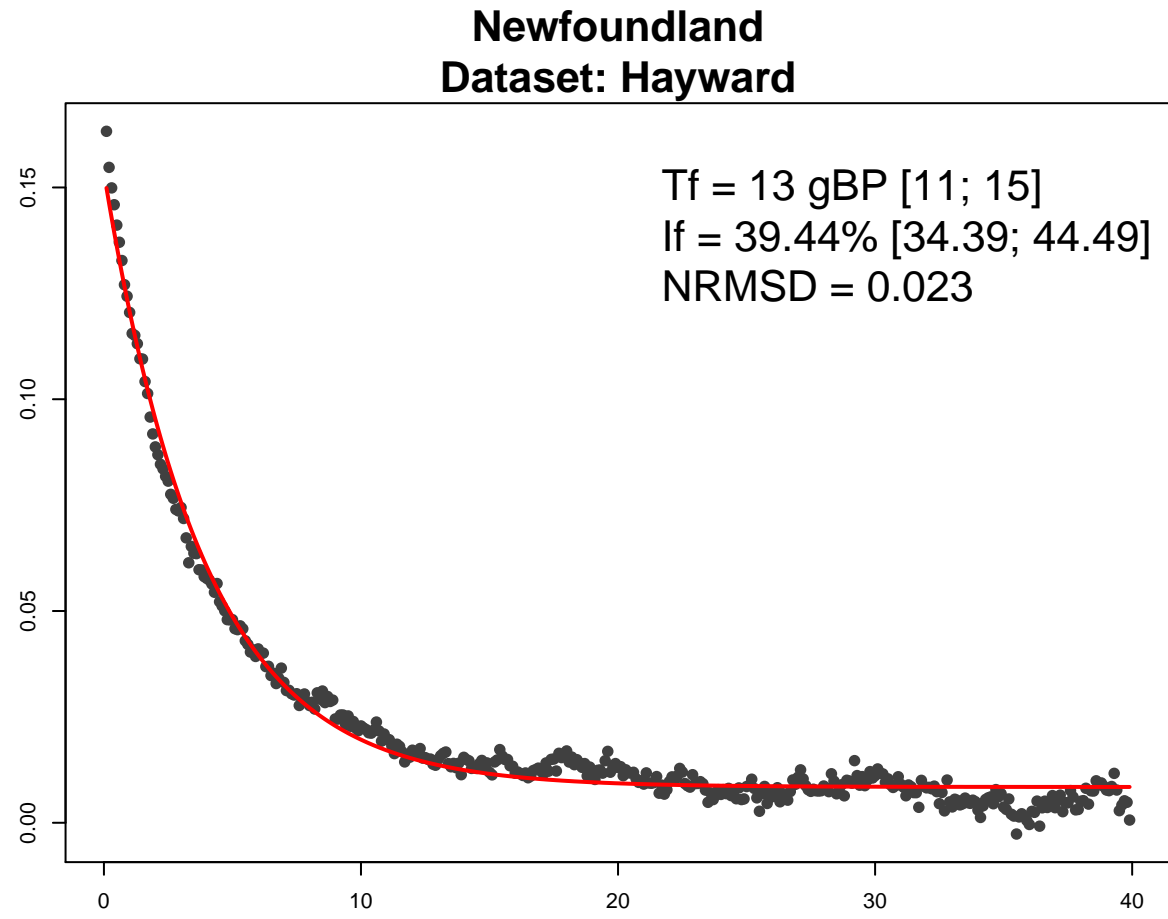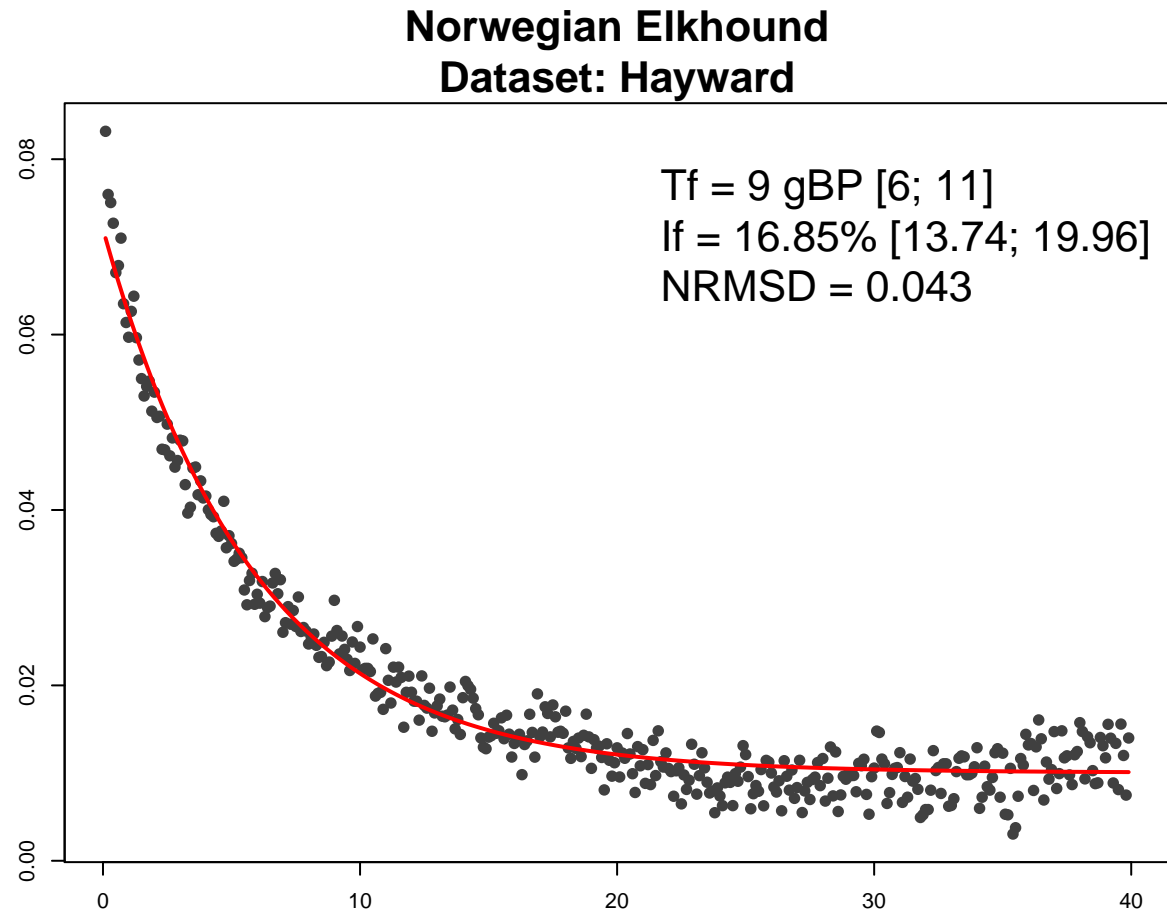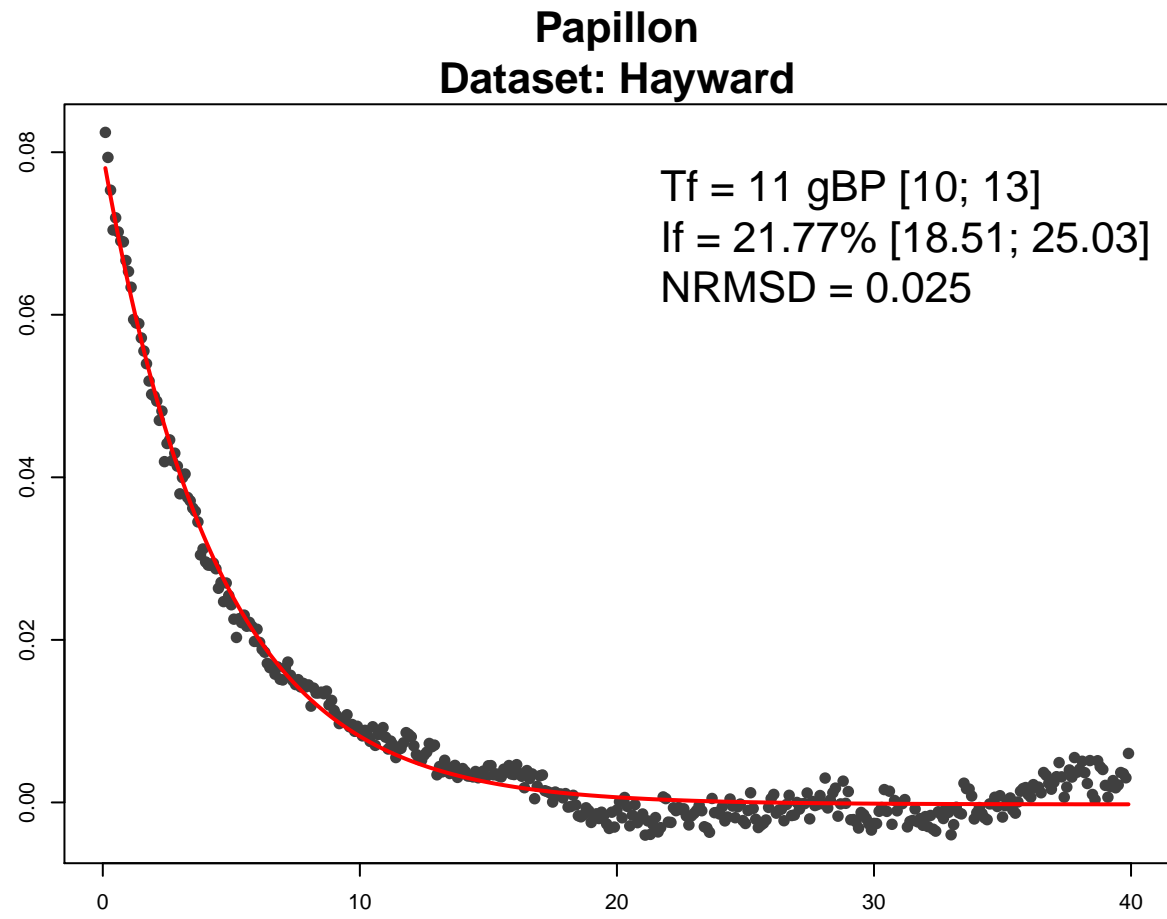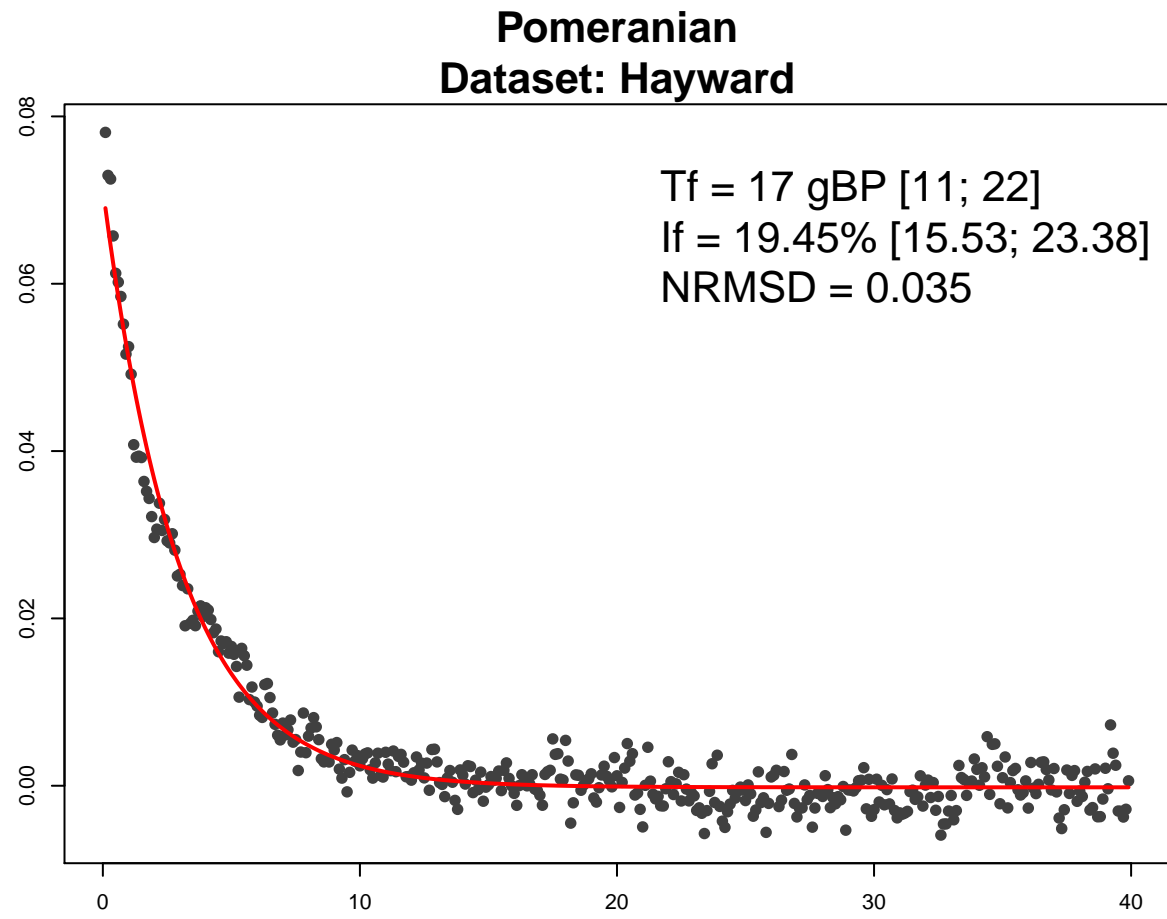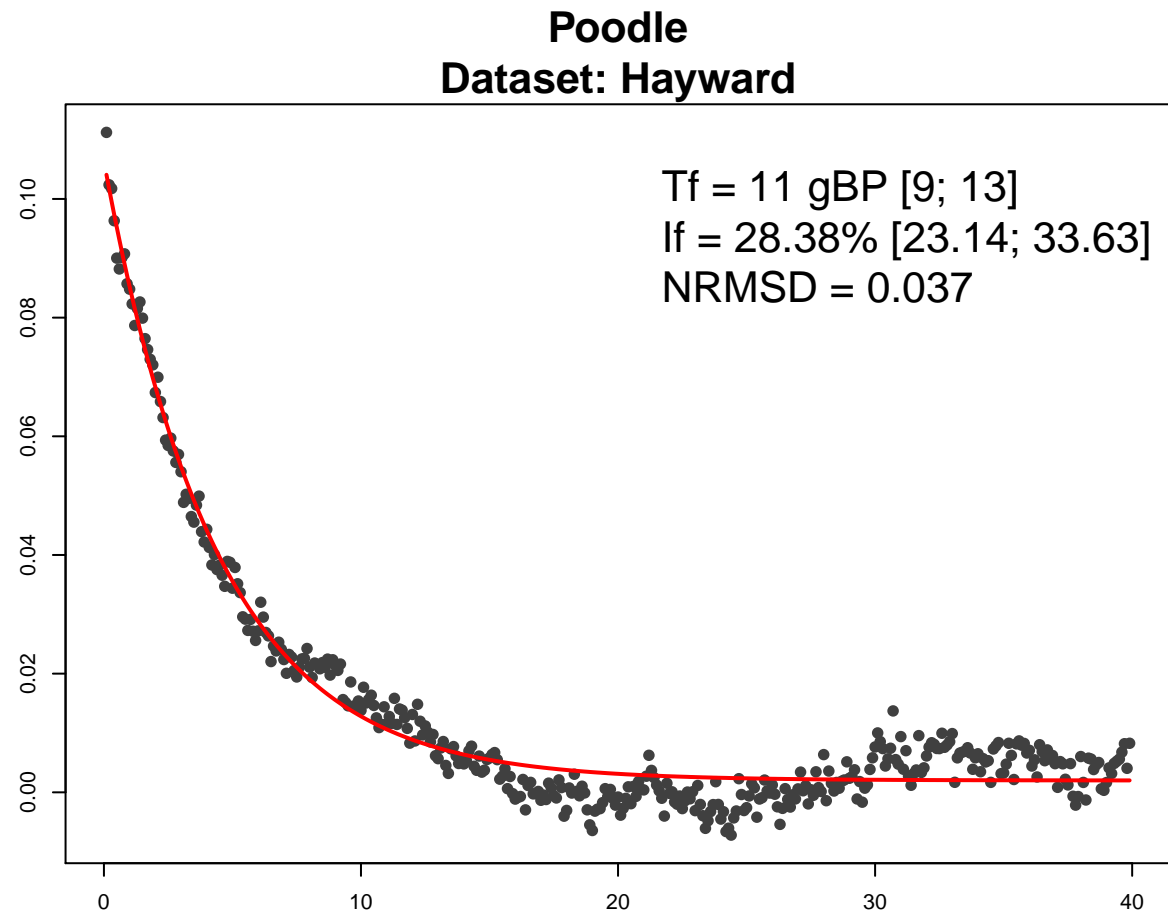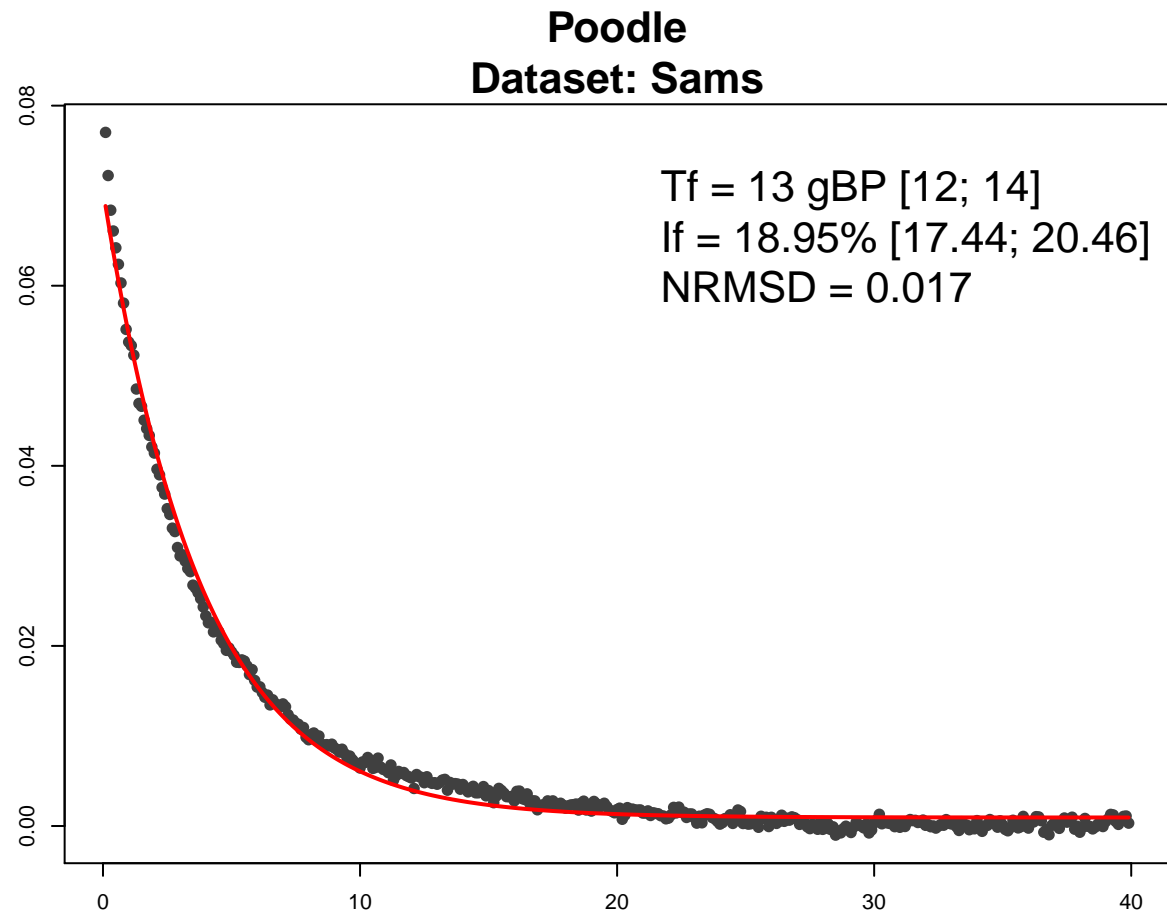

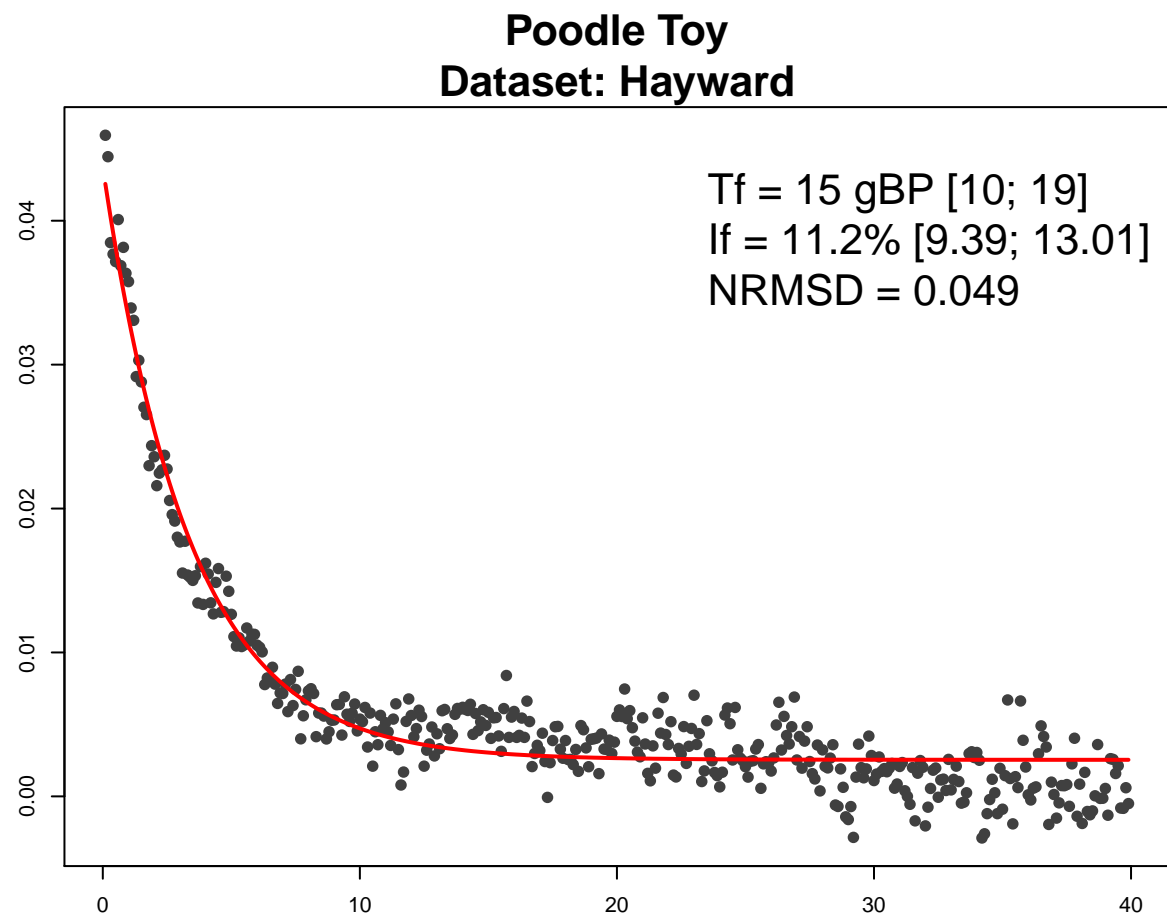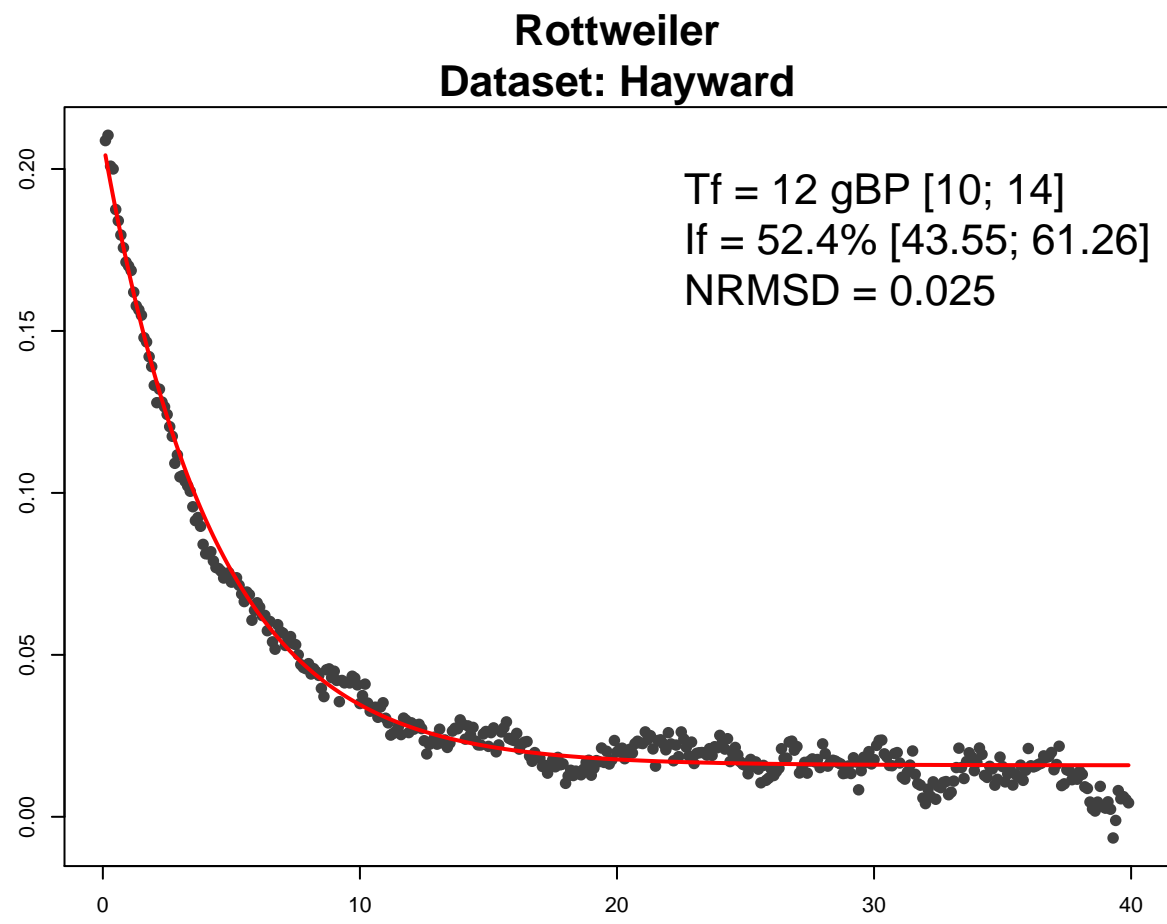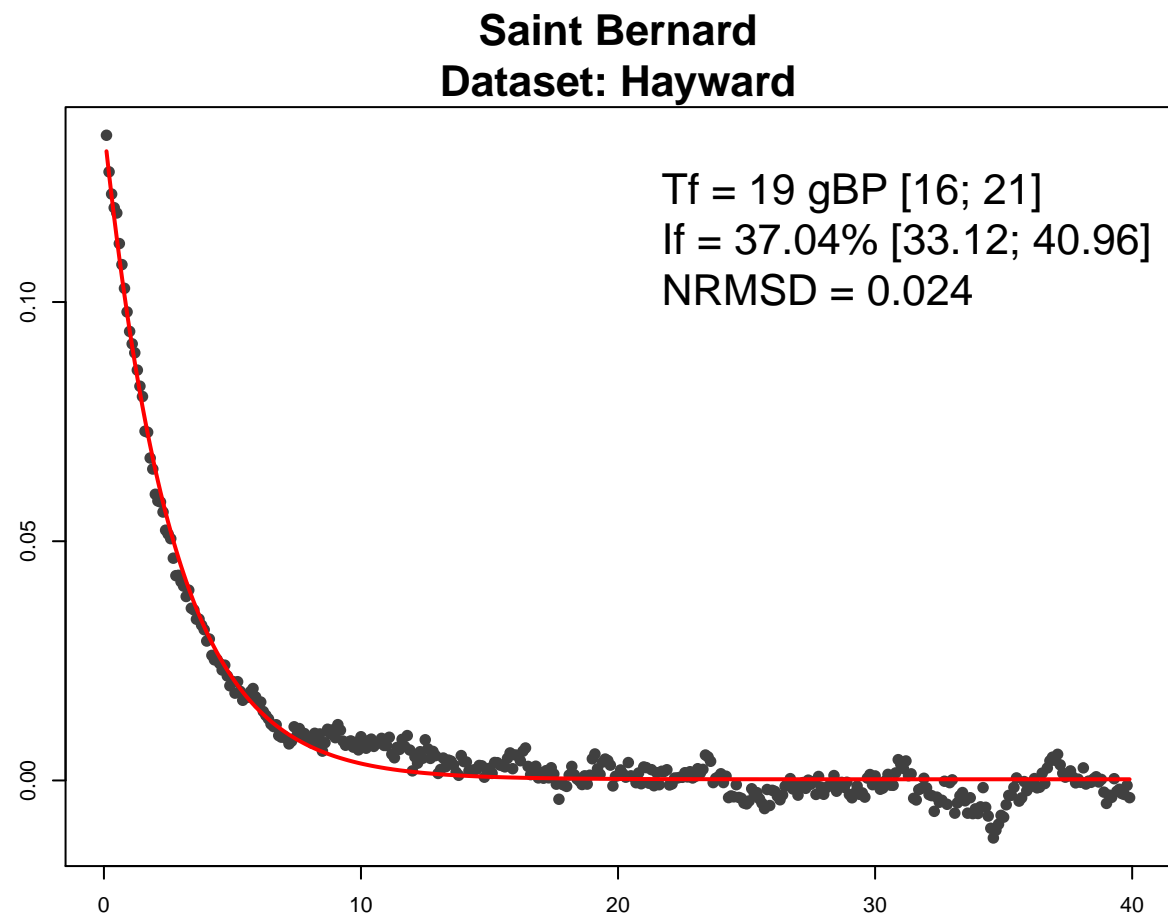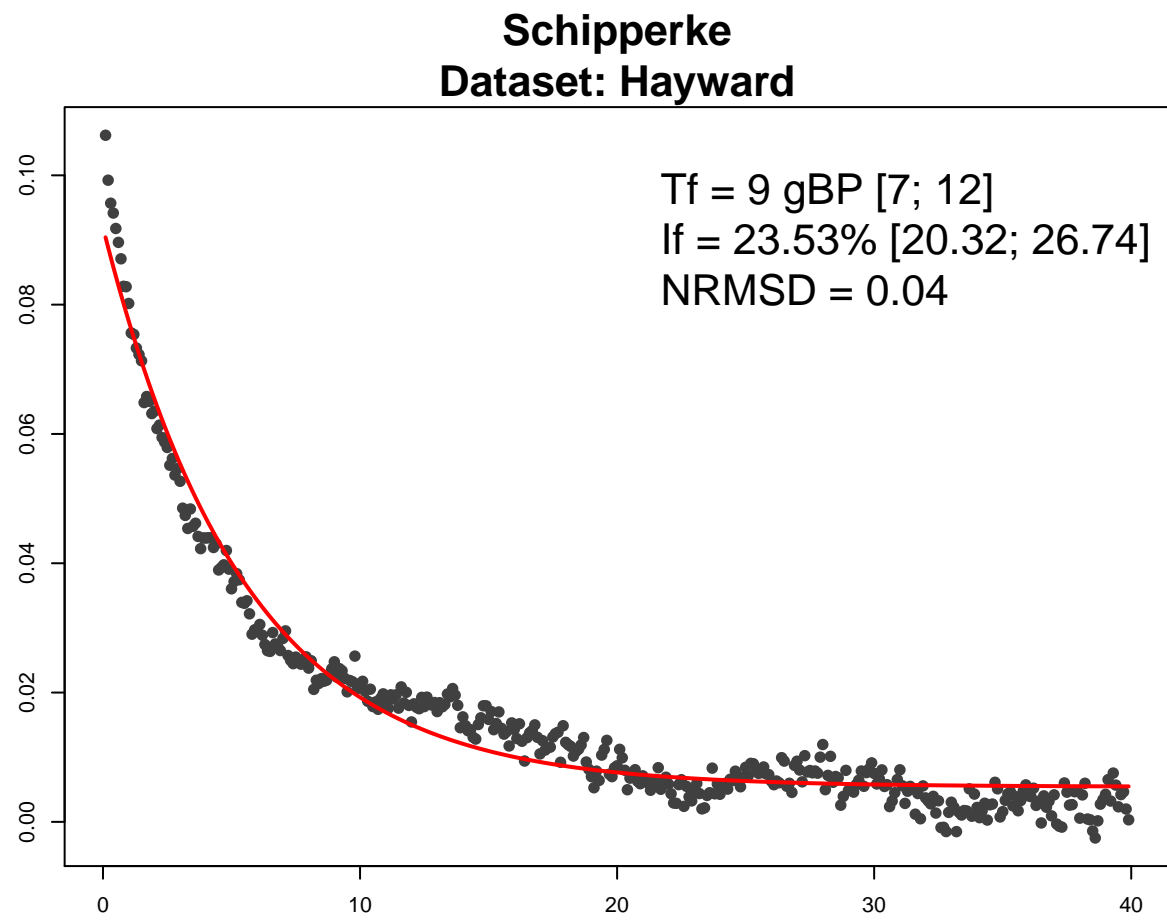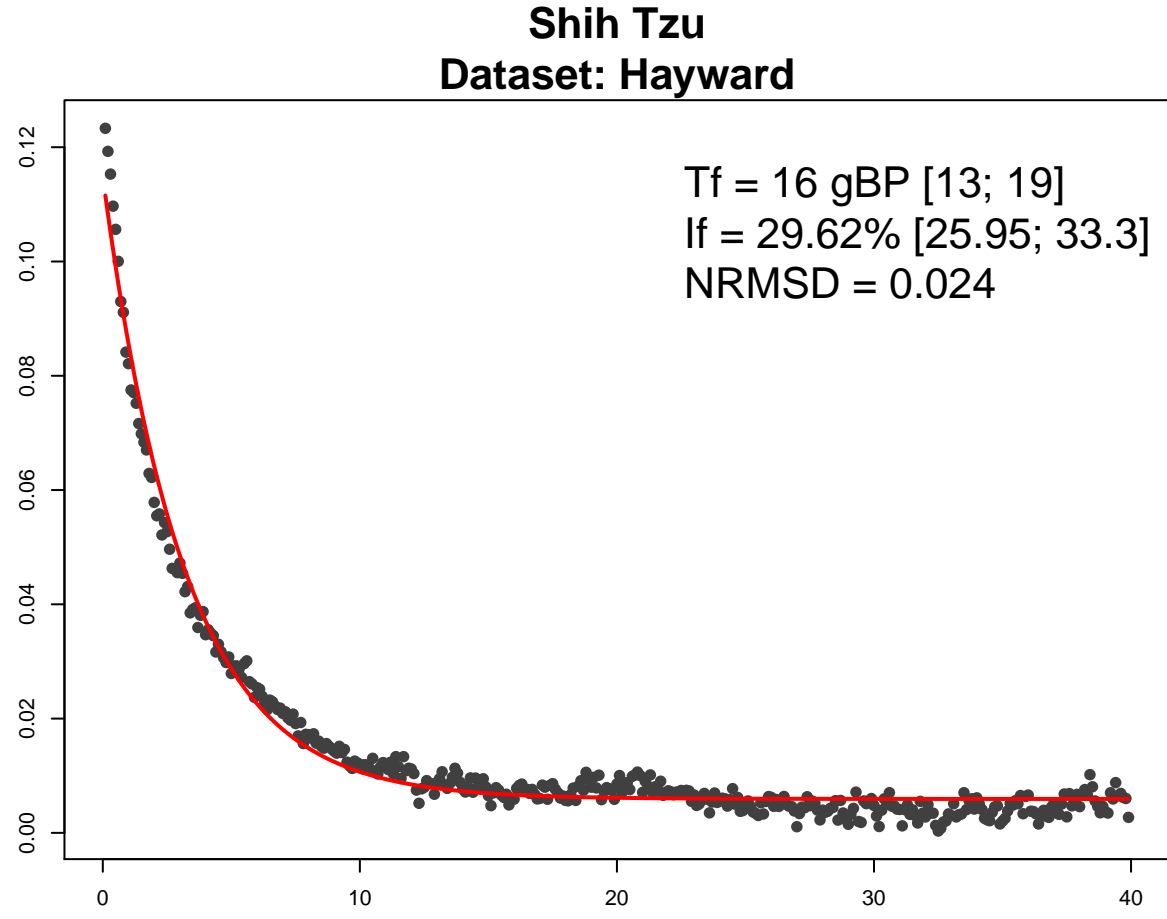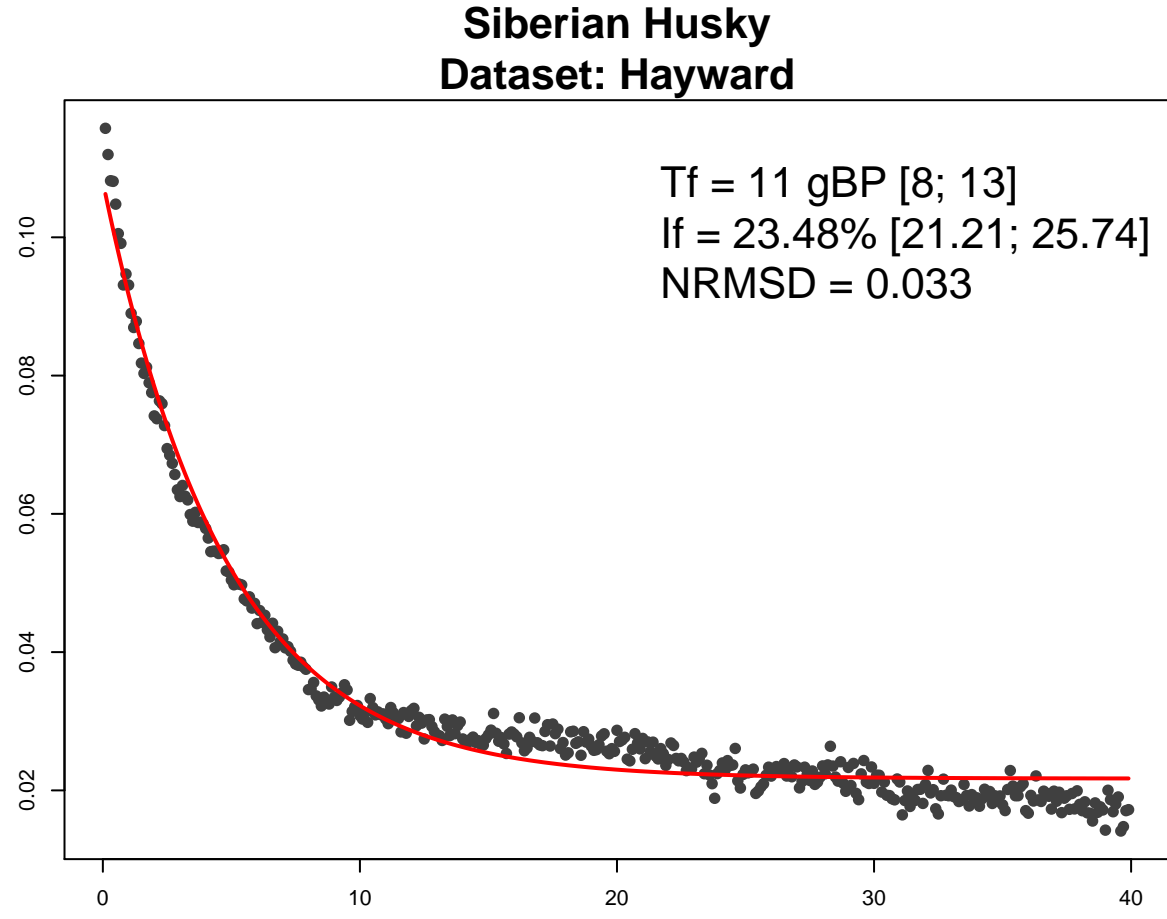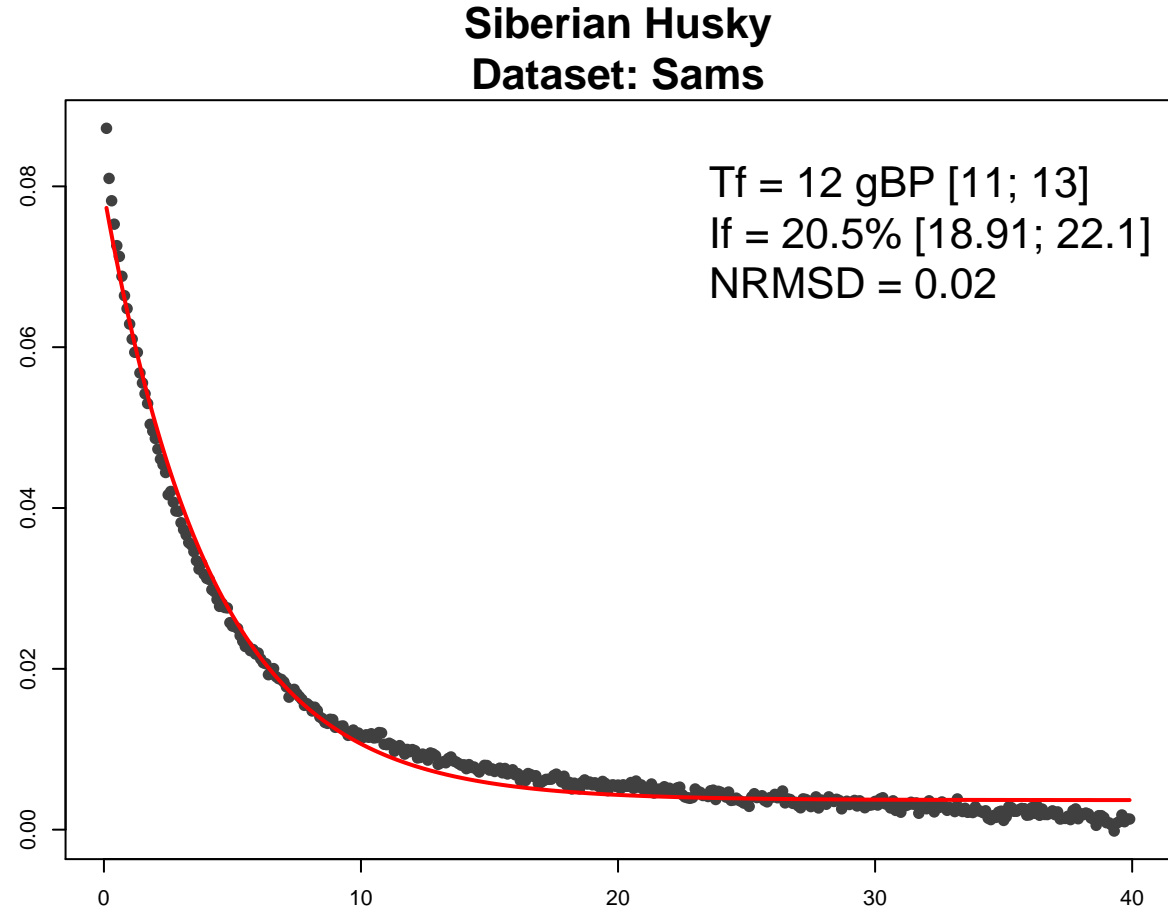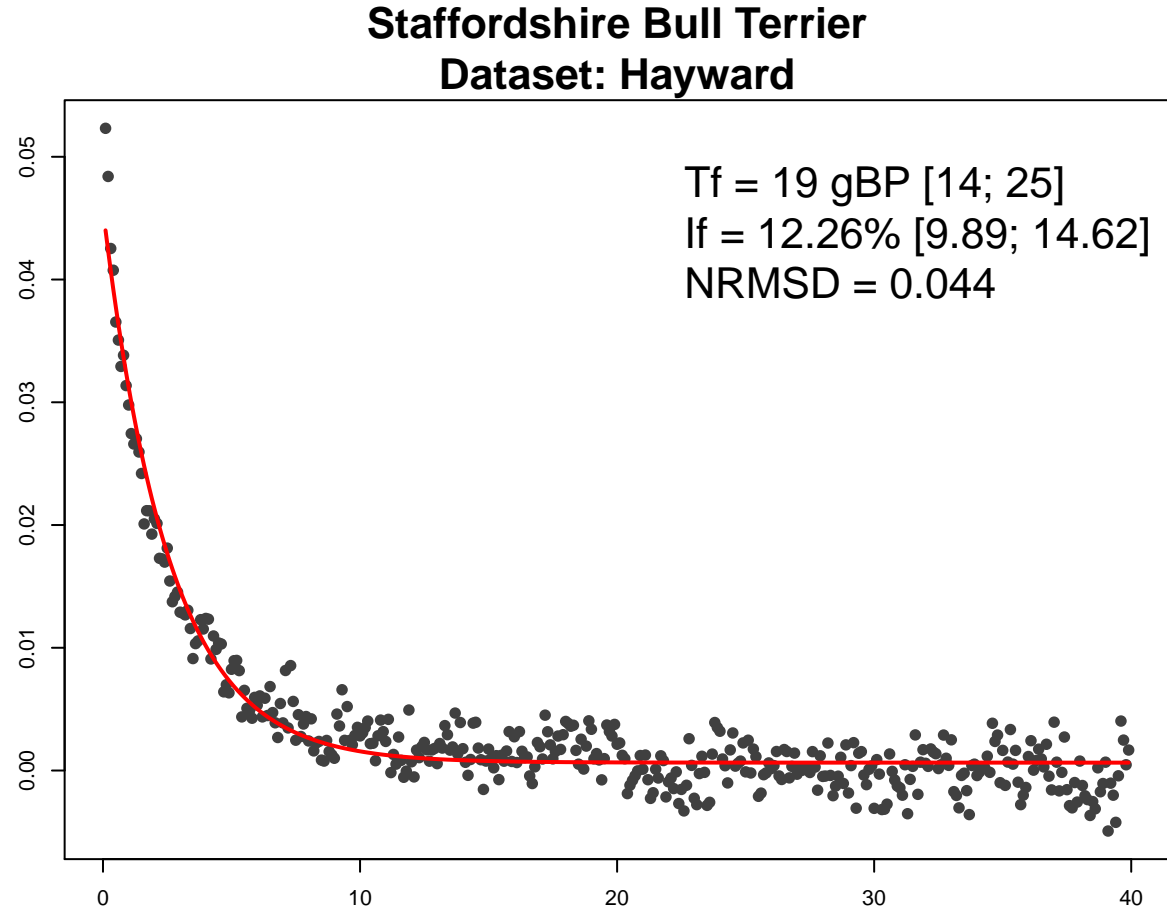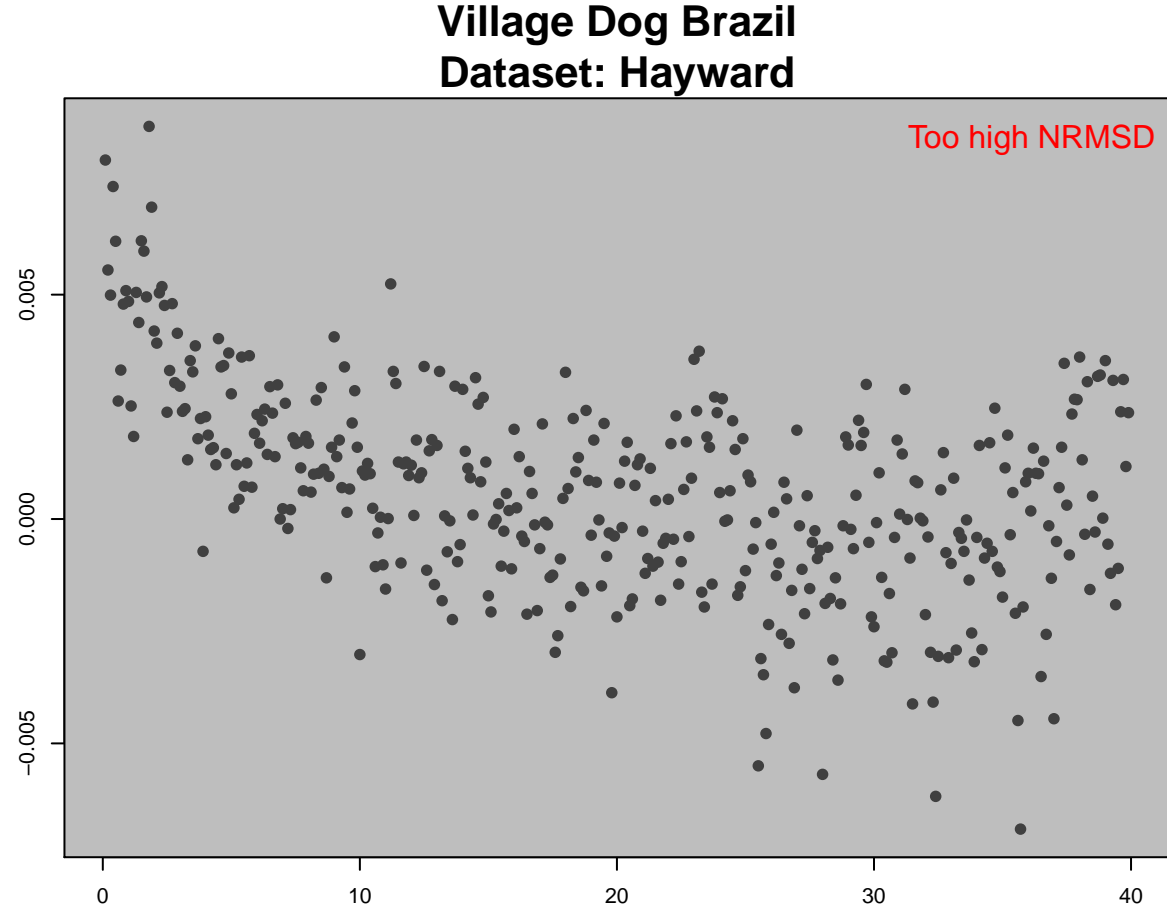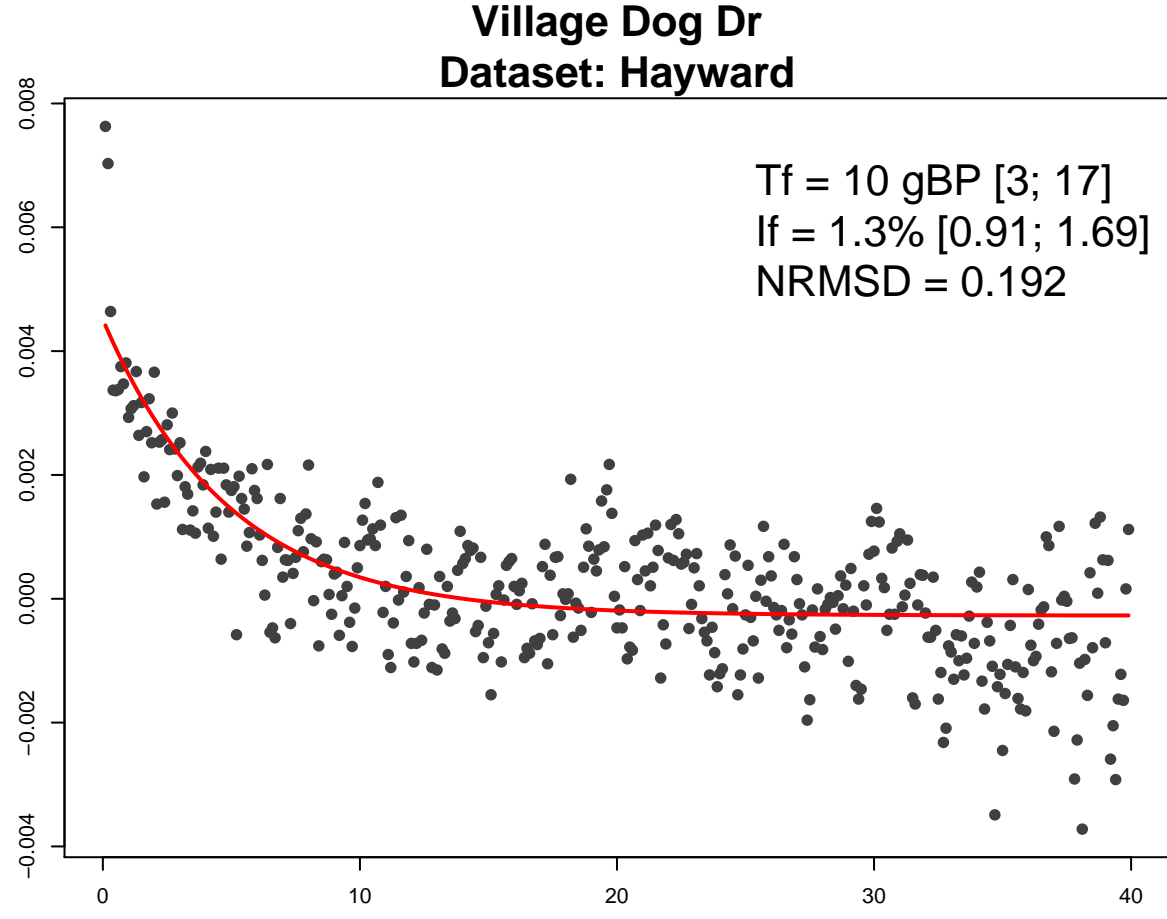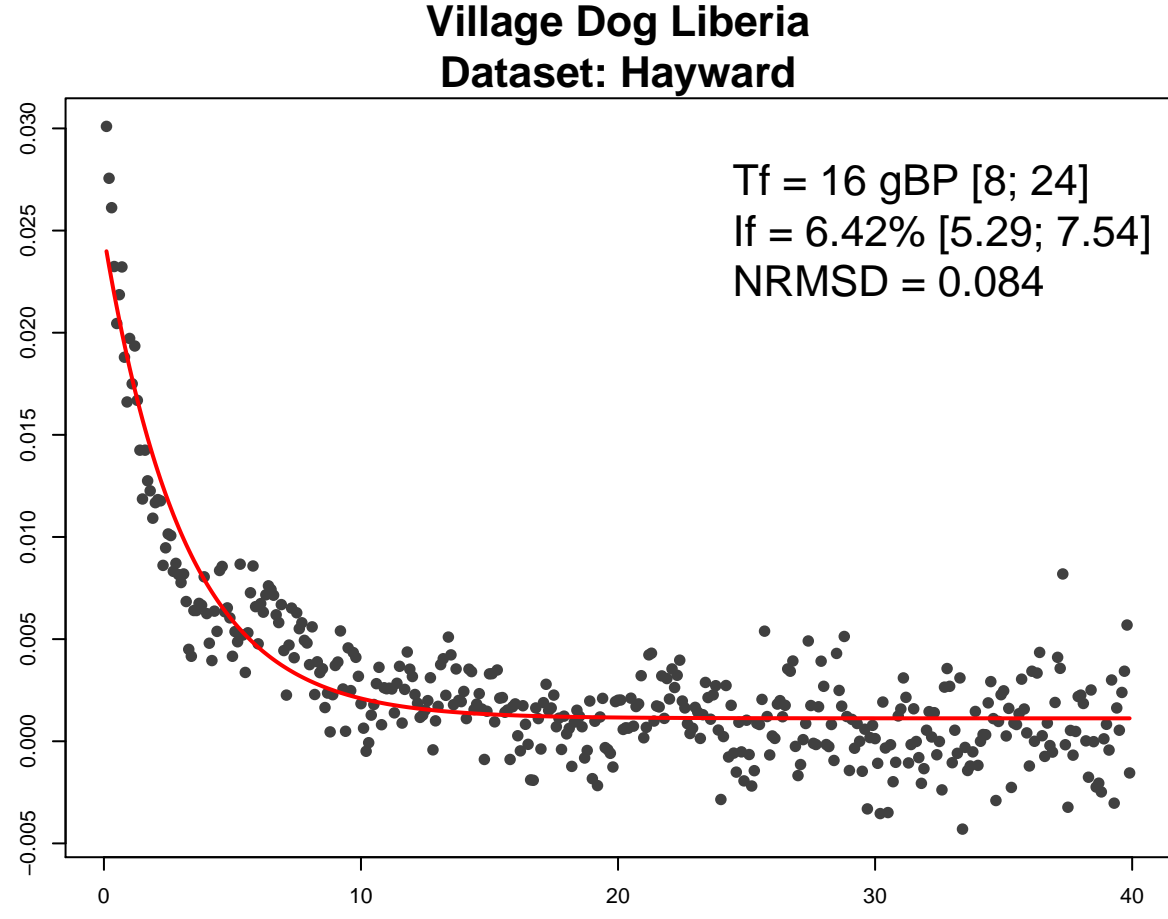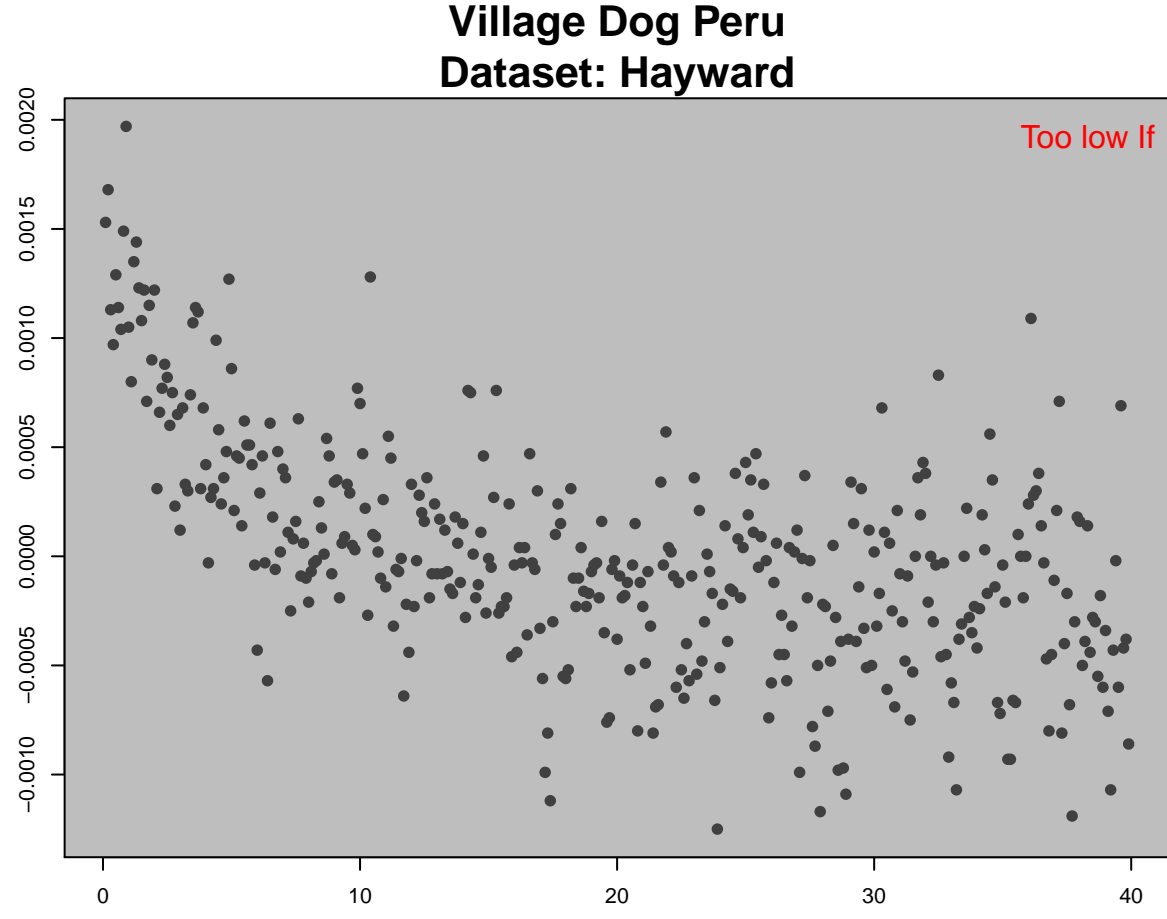

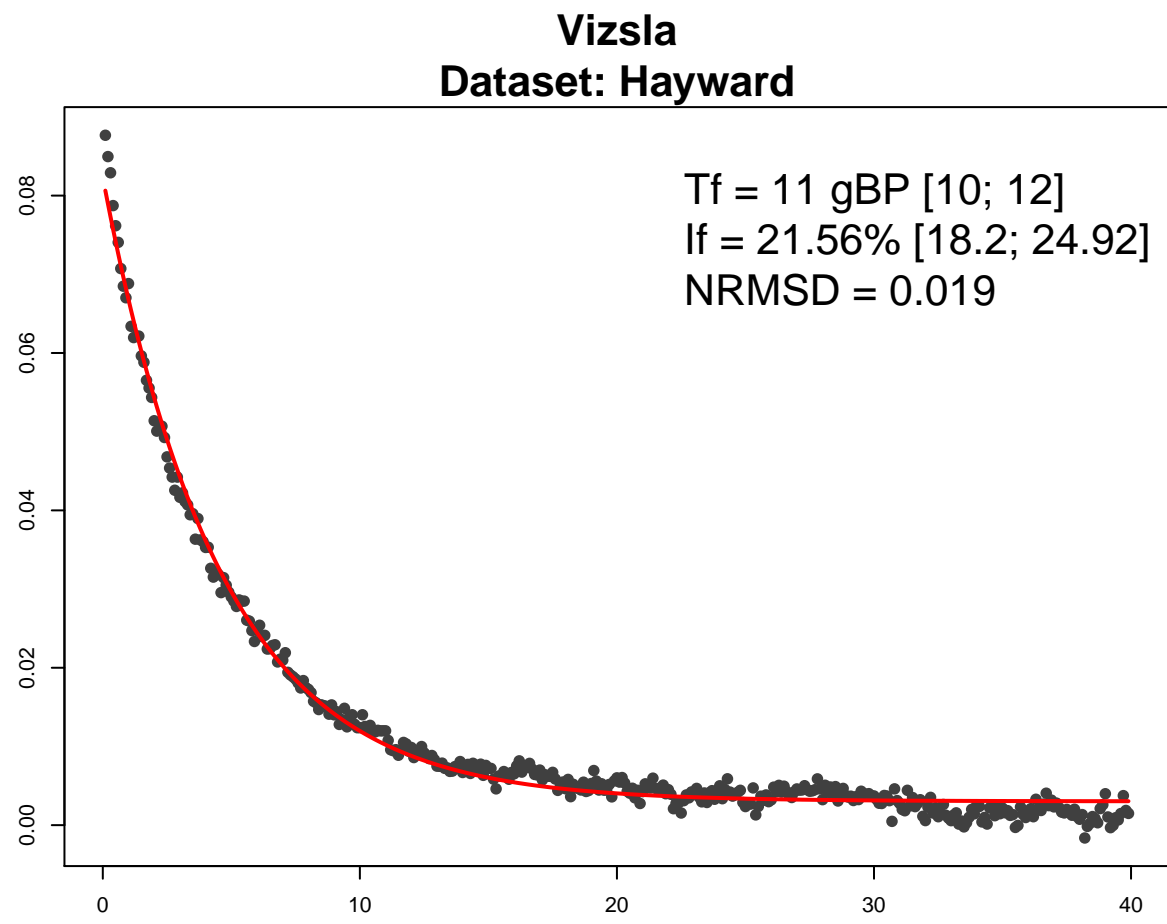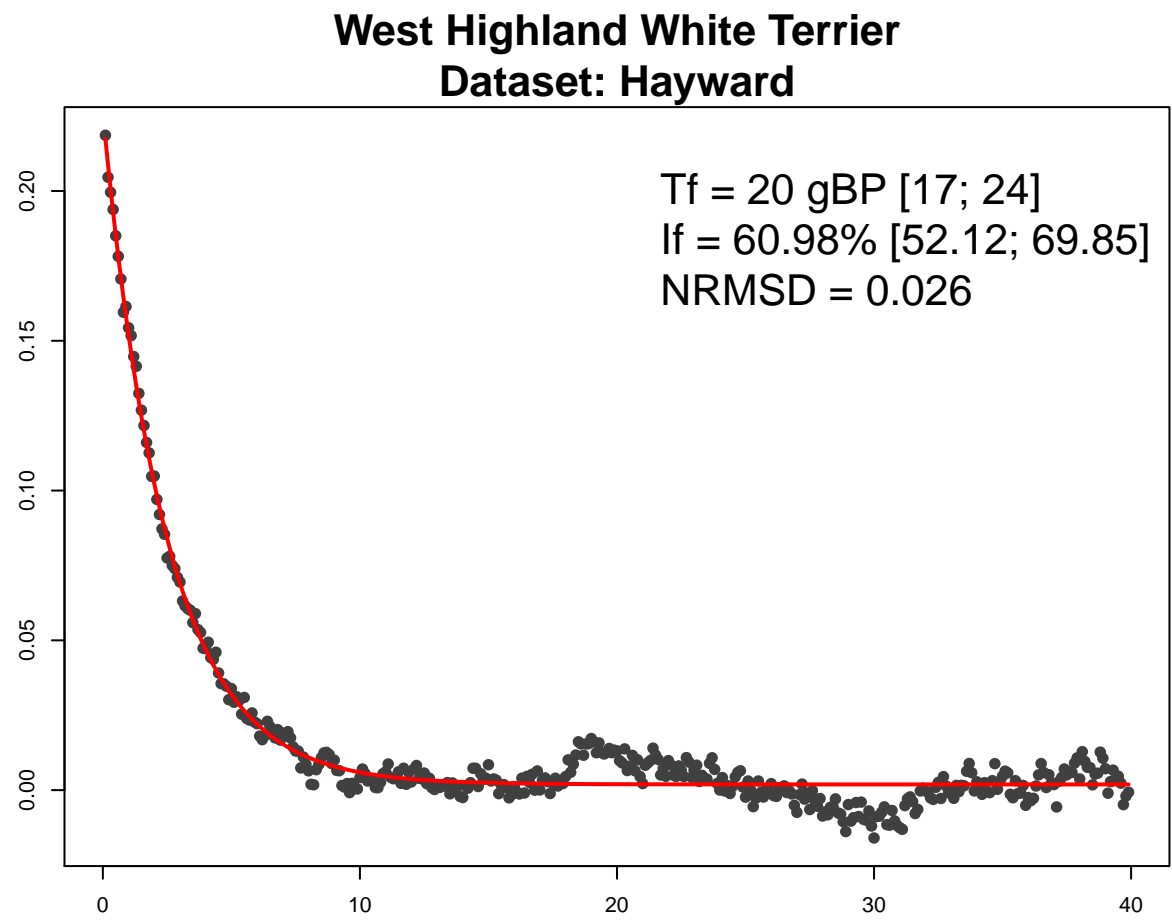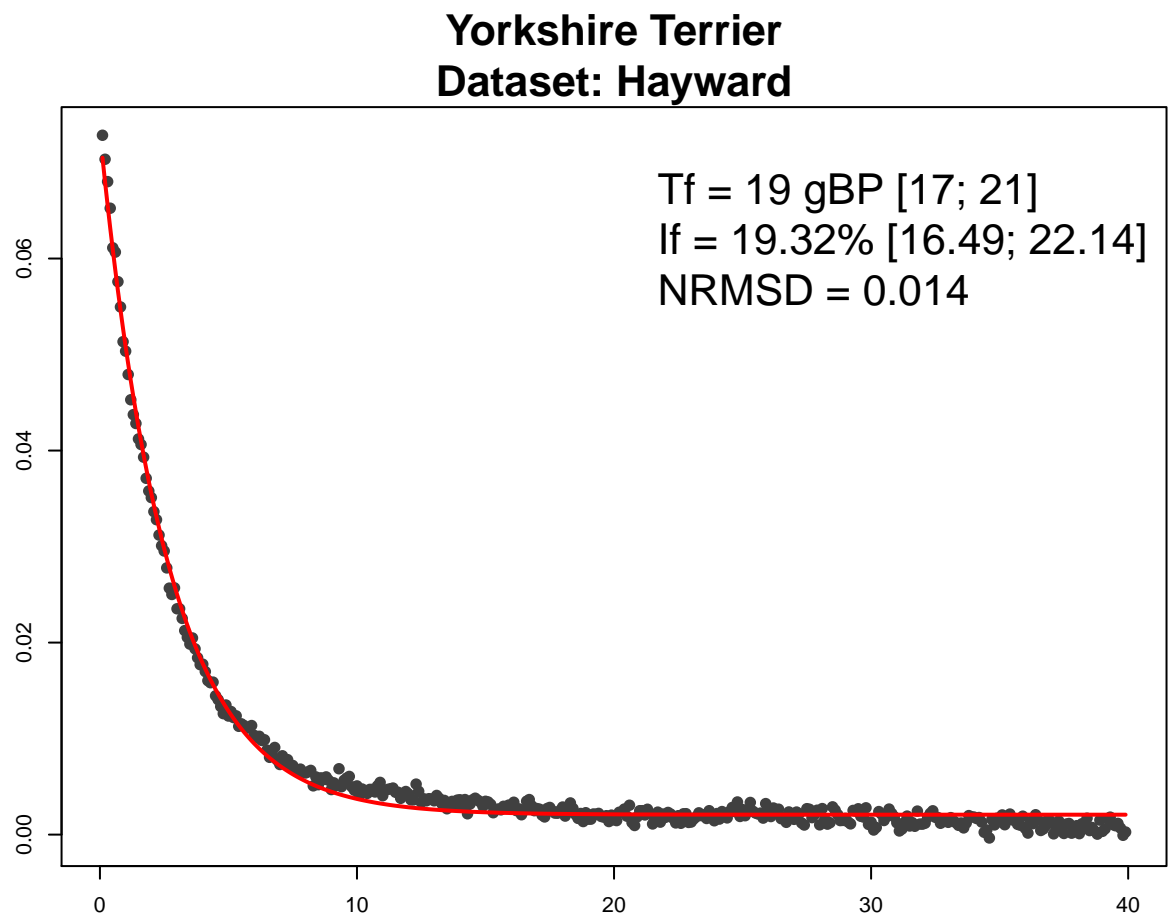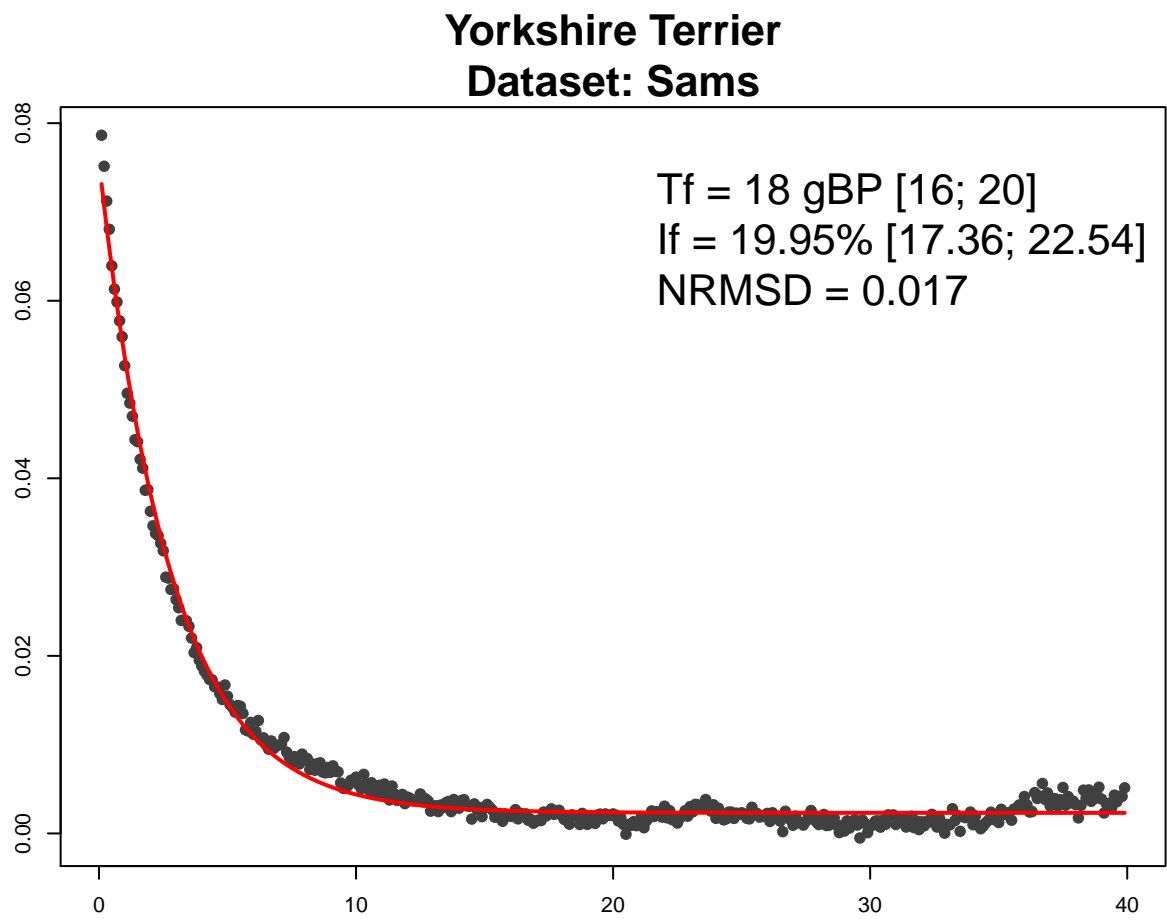

Supplement: S6 Fig — The X-axis represents the genetic distance (in cM) and the Y-axis represents the average allele sharing correlation. The legend shows the mean and 95% confidence interval for the founder age (Tf) and the founder intensity (If), as well as the NRMSD (see Methods). The panels are grayed when the exponential fitting failed or when the evidence for the founder event was not significant (see Methods). The specific reason is highlighted in red in the legend. (PDF) [file pgen.1010243.s006.pdf]
