# Supplementary material for: Reconstructing the history of founder events using genome-wide patterns of allele sharing across individuals
Source: PLoS Genet. 2022 Jun 23;18(6):e1010243. doi: 10.1371/journal.pgen.1010243 (PMC9223333; doi:10.1371/journal.pgen.1010243)
Supplement: S4 Fig — The X-axis represents the genetic distance (in cM) and the Y-axis represents the average weighted allele sharing covariance. The legend shows the mean and 95% confidence interval for the founder age (Tf) in generations before the sampling age of the ancient specimens (gBS), the founder intensity (If), as well as the NRMSD (see Methods). The panels are grayed when the exponential fitting failed or when the evidence for the founder event was not significant (see Methods, here using the standard maximum founder age of 200 gBS). The specific reason is highlighted in red in the legend. (PDF) [file pgen.1010243.s004.pdf]

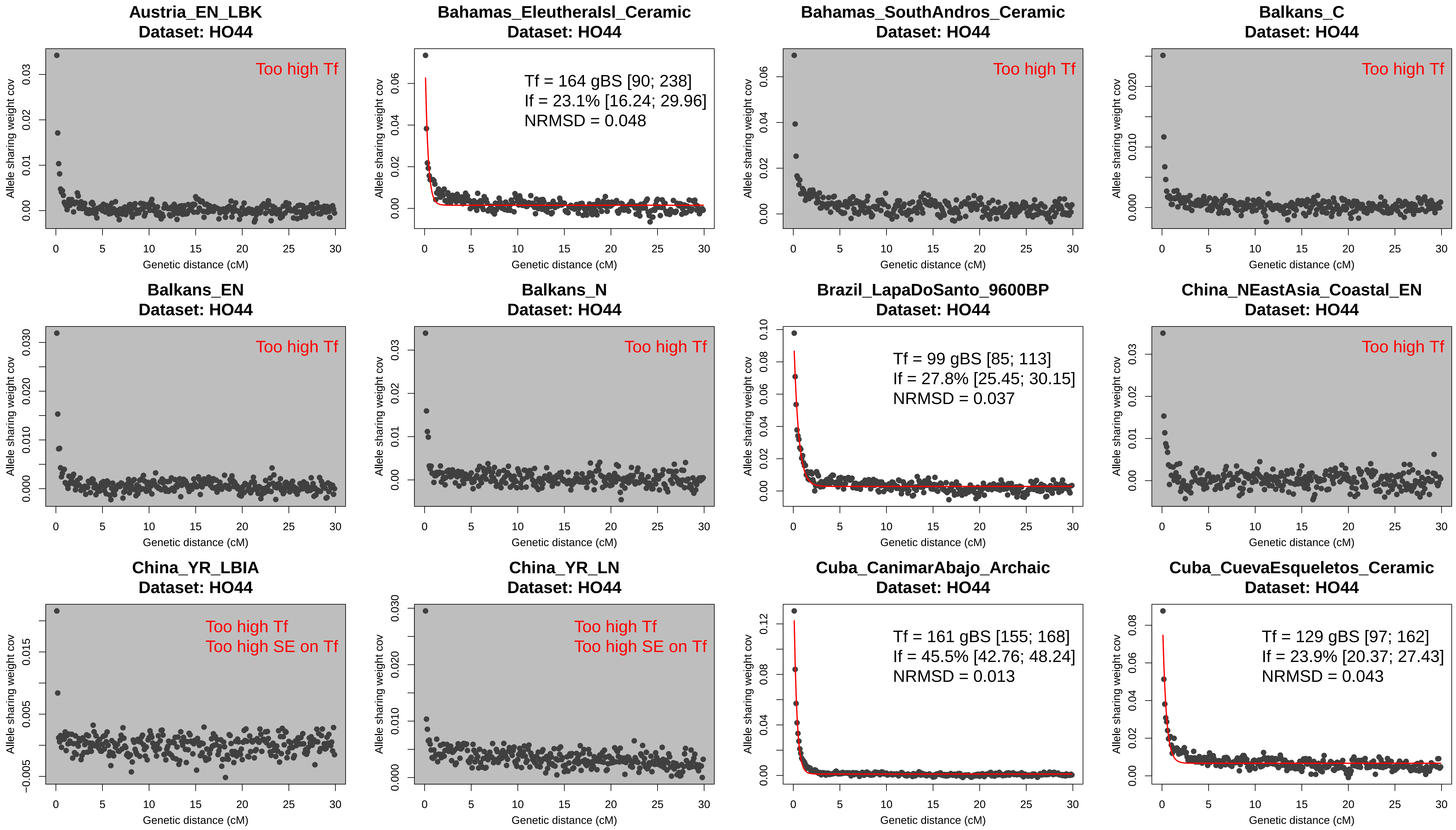
Austria_EN_LBK
Dataset: HO44
Too high Tf
Bahamas_EleutheraIsl_Ceramic
Dataset: HO44
Tf = 164 gBS [90; 238]
If = 23.1% [16.24; 29.96]
NRMSD = 0.048
Bahamas_SouthAndros_Ceramic
Dataset: HO44
Too high Tf
Balkans_C
Dataset: HO44
Too high Tf
Balkans_EN
Dataset: HO44
Too high Tf
Balkans_N
Dataset: HO44
Too high Tf
Brazil_LapaDoSanto_9600BP
Dataset: HO44
Tf = 99 gBS [85; 113]
If = 27.8% [25.45; 30.15]
NRMSD = 0.037
China_NEastAsia_Coastal_EN
Dataset: HO44
Too high Tf
China_YR_LBIA
Dataset: HO44
Too high Tf
Too high SE on Tf
China_YR_LN
Dataset: HO44
Too high Tf
Too high SE on Tf
Cuba_CanimarAbajo_Archaic
Dataset: HO44
Tf = 161 gBS [155; 168]
If = 45.5% [42.76; 48.24]
NRMSD = 0.013
Cuba_CuevaEsqueletos_Ceramic
Dataset: HO44
Tf = 129 gBS [97; 162]
If = 23.9% [20.37; 27.43]
NRMSD = 0.043
Allele sharing weight cov
Genetic distance (cM)

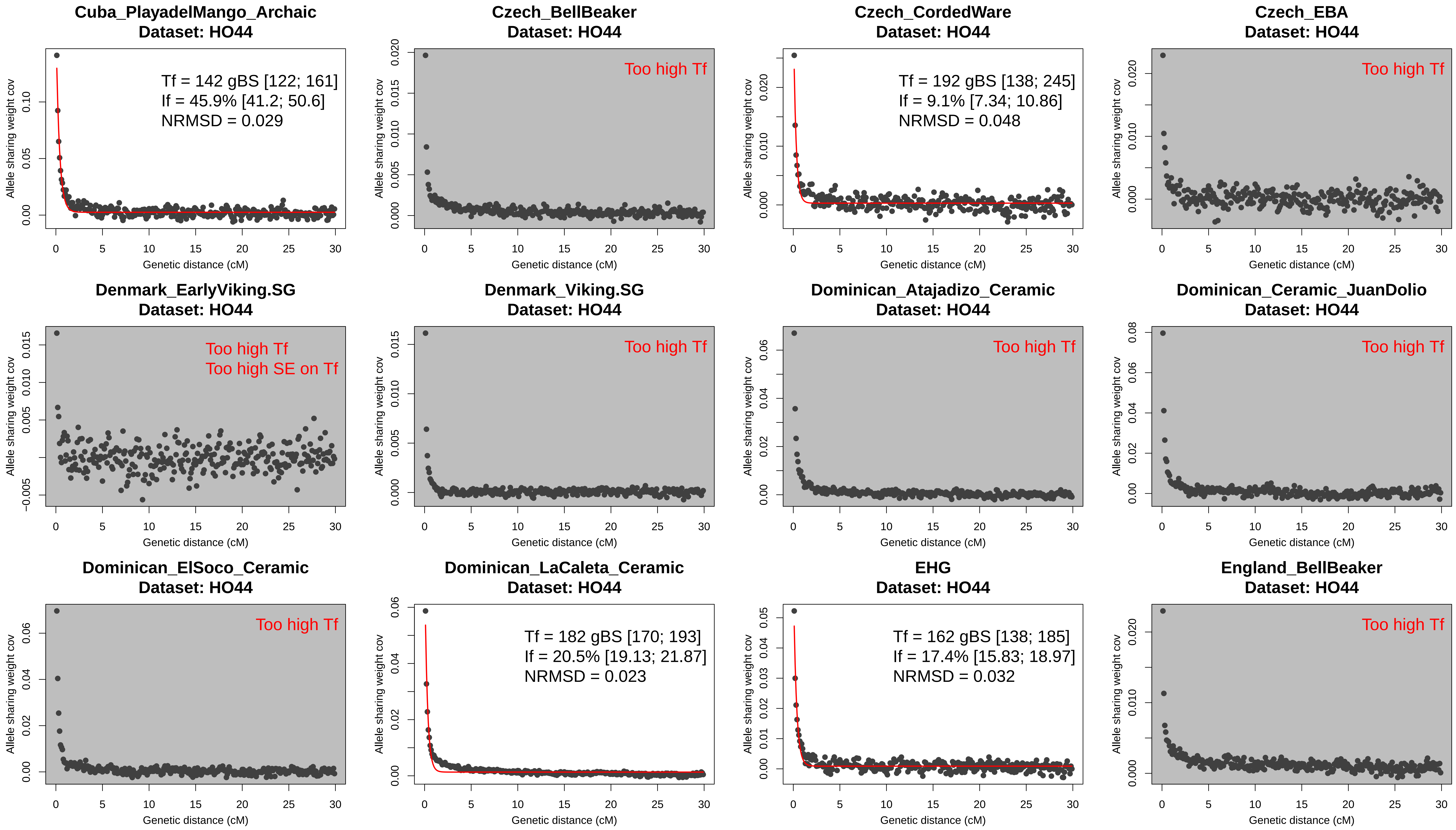
Cuba_PlayadelMango_Archaic
Dataset: HO44
Tf = 142 gBS [122; 161]
If = 45.9% [41.2; 50.6]
NRMSD = 0.029
Czech_BellBeaker
Dataset: HO44
Too high Tf
Czech_CordedWare
Dataset: HO44
Tf = 192 gBS [138; 245]
If = 9.1% [7.34; 10.86]
NRMSD = 0.048
Czech_EBA
Dataset: HO44
Too high Tf
Denmark_EarlyViking.SG
Dataset: HO44
Too high Tf
Too high SE on Tf
Denmark_Viking.SG
Dataset: HO44
Too high Tf
Dominican_Atajadizo_Ceramic
Dataset: HO44
Too high Tf
Dominican_Ceramic_JuanDolio
Dataset: HO44
Too high Tf
Dominican_ElSoco_Ceramic
Dataset: HO44
Too high Tf
Dominican_LaCaleta_Ceramic
Dataset: HO44
Tf = 182 gBS [170; 193]
If = 20.5% [19.13; 21.87]
NRMSD = 0.023
EHG
Dataset: HO44
Tf = 162 gBS [138; 185]
If = 17.4% [15.83; 18.97]
NRMSD = 0.032
England_BellBeaker
Dataset: HO44
Too high Tf
Allele sharing weight cov
Genetic distance (cM)

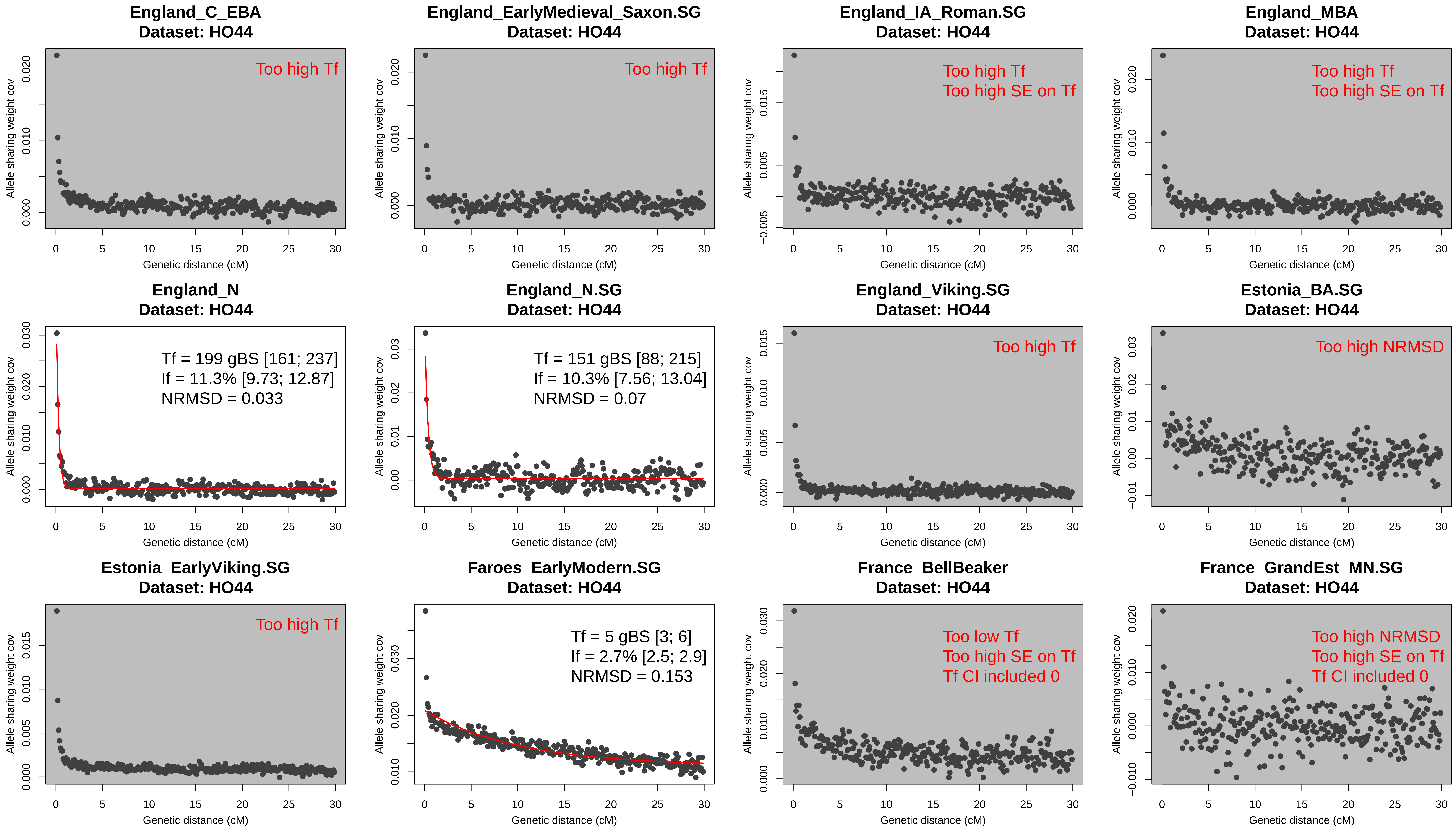
England_C_EBA
Dataset: HO44
Too high Tf
England_EarlyMedieval_Saxon.SG
Dataset: HO44
Too high Tf
England_IA_Roman.SG
Dataset: HO44
Too high Tf
Too high SE on Tf
England_MBA
Dataset: HO44
Too high Tf
Too high SE on Tf
England_N
Dataset: HO44
Tf = 199 gBS [161; 237]
If = 11.3% [9.73; 12.87]
NRMSD = 0.033
England_N.SG
Dataset: HO44
Tf = 151 gBS [88; 215]
If = 10.3% [7.56; 13.04]
NRMSD = 0.07
England_Viking.SG
Dataset: HO44
Too high Tf
Estonia_BA.SG
Dataset: HO44
Too high NRMSD
Estonia_EarlyViking.SG
Dataset: HO44
Too high Tf
Faroes_EarlyModern.SG
Dataset: HO44
Tf = 5 gBS [3; 6]
If = 2.7% [2.5; 2.9]
NRMSD = 0.153
France_BellBeaker
Dataset: HO44
Too low Tf
Too high SE on Tf
Tf CI included 0
France_GrandEst_MN.SG
Dataset: HO44
Too high NRMSD
Too high SE on Tf
Tf CI included 0
Allele sharing weight cov
Genetic distance (cM)

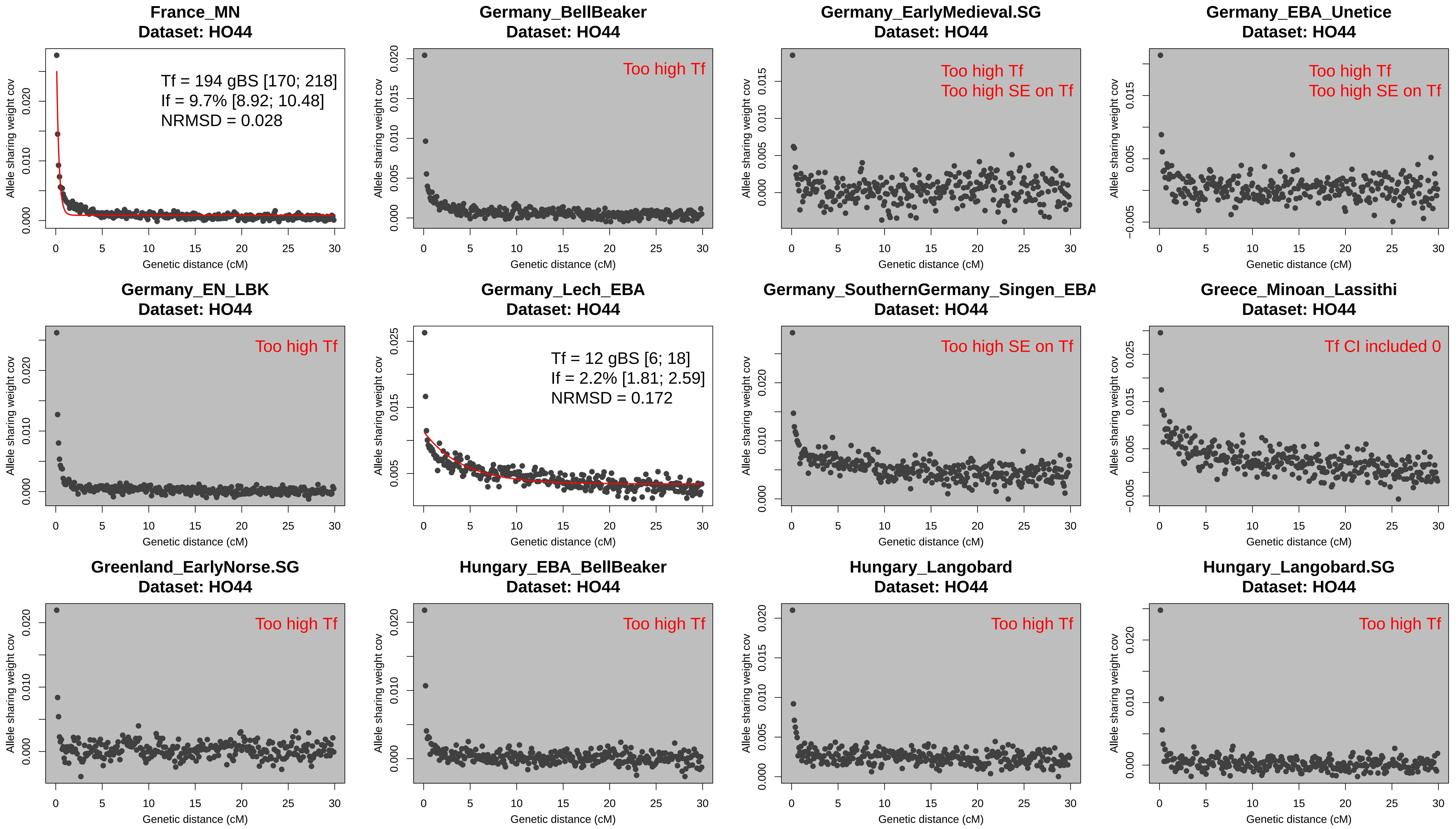
France_MN
Dataset: HO44
Tf = 194 gBS [170; 218]
If = 9.7% [8.92; 10.48]
NRMSD = 0.028
Germany_BellBeaker
Dataset: HO44
Too high Tf
Germany_EarlyMedieval.SG
Dataset: HO44
Too high Tf
Too high SE on Tf
Germany_EBA_Unetice
Dataset: HO44
Too high Tf
Too high SE on Tf
Germany_EN_LBK
Dataset: HO44
Too high Tf
Germany_Lech_EBA
Dataset: HO44
Tf = 12 gBS [6; 18]
If = 2.2% [1.81; 2.59]
NRMSD = 0.172
Germany_SouthernGermany_Singen_EBA
Dataset: HO44
Too high SE on Tf
Greece_Minoan_Lassithi
Dataset: HO44
Tf CI included 0
Greenland_EarlyNorse.SG
Dataset: HO44
Too high Tf
Hungary_EBA_BellBeaker
Dataset: HO44
Too high Tf
Hungary_Langobard
Dataset: HO44
Too high Tf
Hungary_Langobard.SG
Dataset: HO44
Too high Tf
Allele sharing weight cov
Genetic distance (cM)

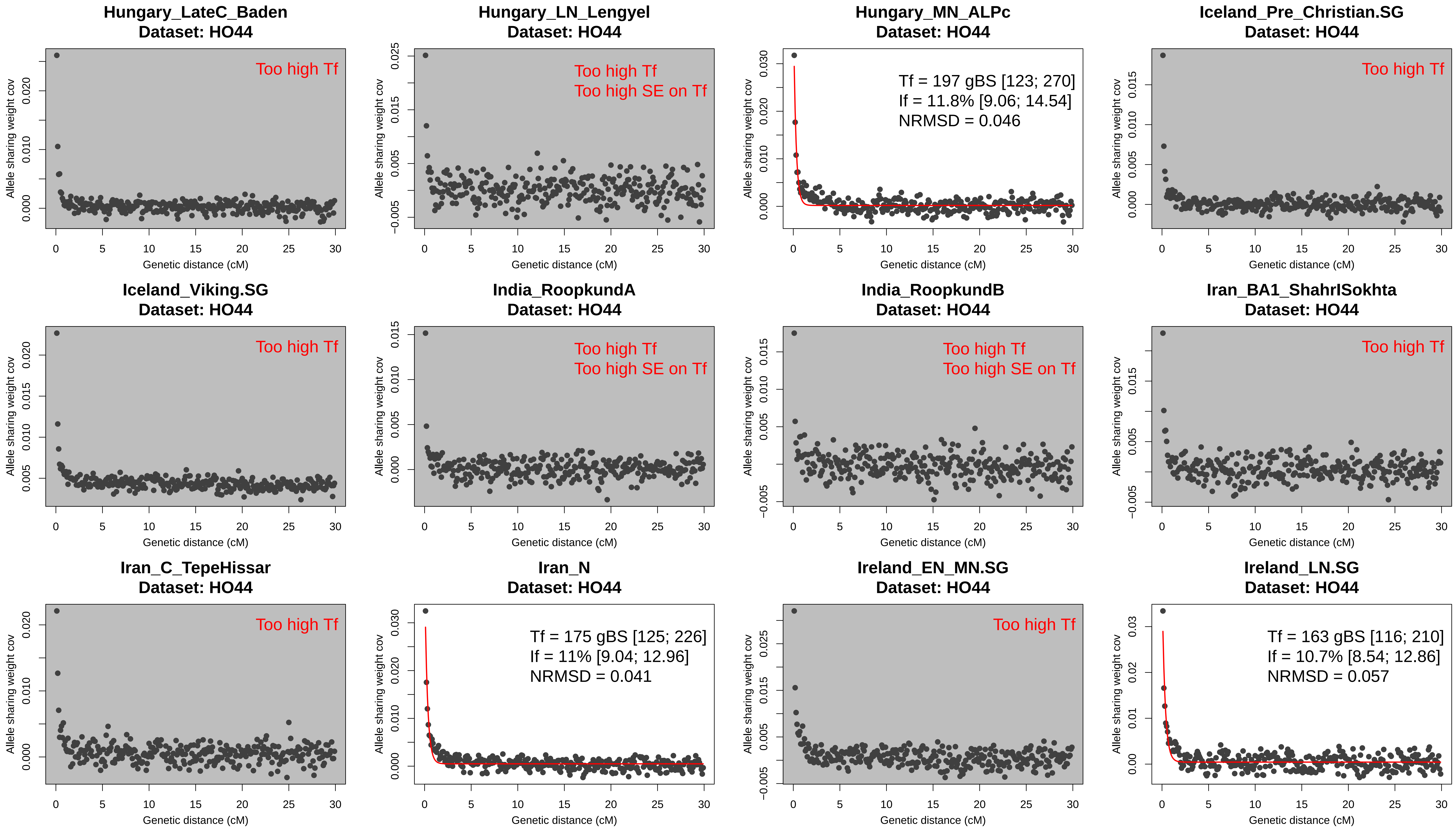
Hungary_LateC_Baden
Dataset: HO44
Too high Tf
Hungary_LN_Lengyel
Dataset: HO44
Too high Tf
Too high SE on Tf
Hungary_MN_ALPc
Dataset: HO44
Tf = 197 gBS [123; 270]
If = 11.8% [9.06; 14.54]
NRMSD = 0.046
Iceland_Pre_Christian.SG
Dataset: HO44
Too high Tf
Iceland_Viking.SG
Dataset: HO44
Too high Tf
India_RoopkundA
Dataset: HO44
Too high Tf
Too high SE on Tf
India_RoopkundB
Dataset: HO44
Too high Tf
Too high SE on Tf
Iran_BA1_ShahrISokhta
Dataset: HO44
Too high Tf
Iran_C_TepeHissar
Dataset: HO44
Too high Tf
Iran_N
Dataset: HO44
Tf = 175 gBS [125; 226]
If = 11% [9.04; 12.96]
NRMSD = 0.041
Ireland_EN_MN.SG
Dataset: HO44
Too high Tf
Ireland_LN.SG
Dataset: HO44
Tf = 163 gBS [116; 210]
If = 10.7% [8.54; 12.86]
NRMSD = 0.057
Allele sharing weight cov
Genetic distance (cM)

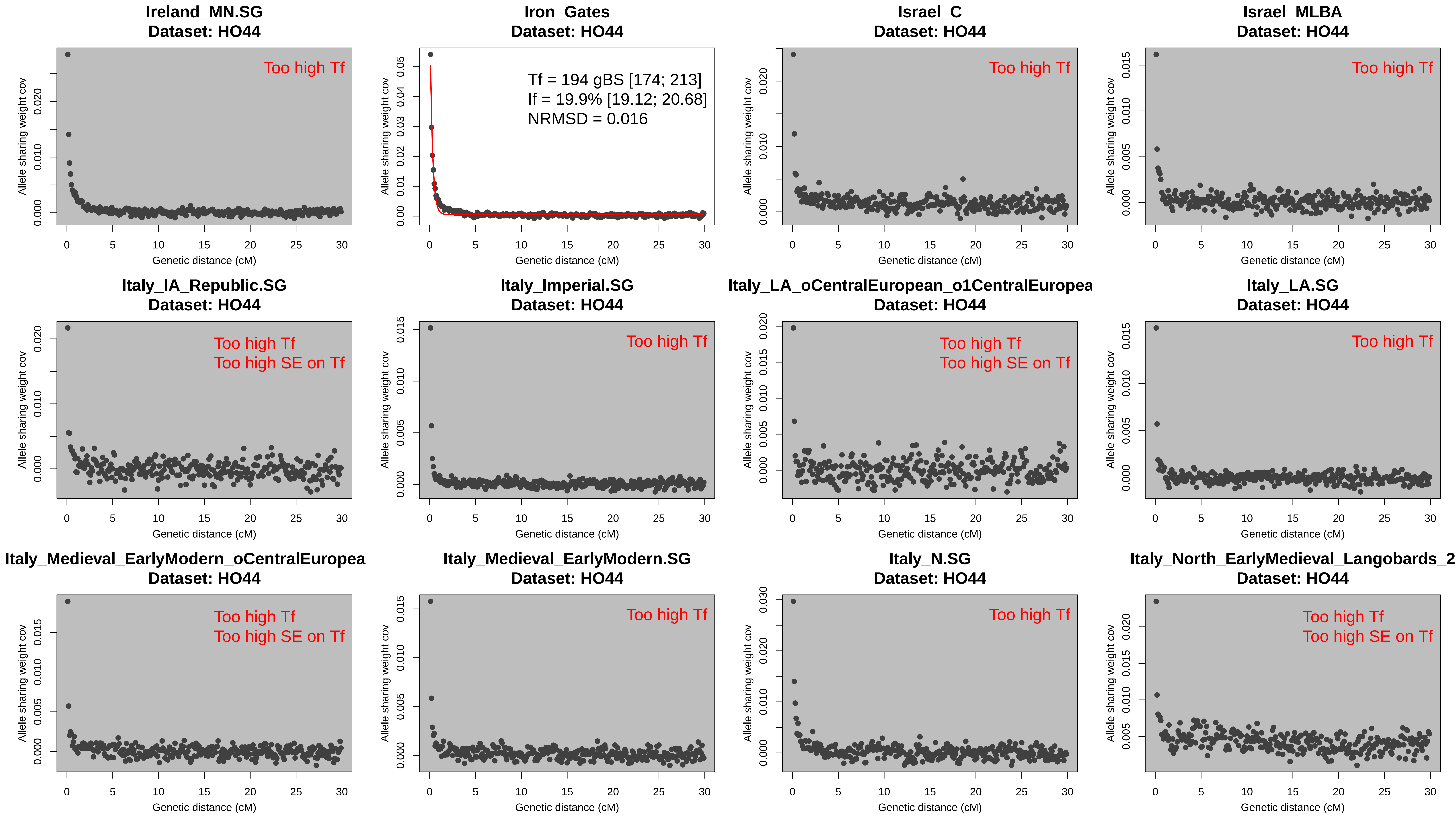

Ireland_MN.SG
Dataset: HO44
Too high Tf
Iron_Gates
Dataset: HO44
Tf = 194 gBS [174; 213]
If = 19.9% [19.12; 20.68]
NRMSD = 0.016
Israel_C
Dataset: HO44
Too high Tf
Israel_MLBA
Dataset: HO44
Too high Tf
Italy_IA_Republic.SG
Dataset: HO44
Too high Tf
Too high SE on Tf
Italy_Imperial.SG
Dataset: HO44
Too high Tf
Italy_LA_oCentralEuropean_o1CentralEuropea
Dataset: HO44
Too high Tf
Too high SE on Tf
Italy_LA.SG
Dataset: HO44
Too high Tf
Italy_Medieval_EarlyModern_oCentralEuropea
Dataset: HO44
Too high Tf
Too high SE on Tf
Italy_Medieval_EarlyModern.SG
Dataset: HO44
Too high Tf
Italy_N.SG
Dataset: HO44
Too high Tf
Italy_North_EarlyMedieval_Langobards_2
Dataset: HO44
Too high Tf
Too high SE on Tf
Allele sharing weight cov
Genetic distance (cM)

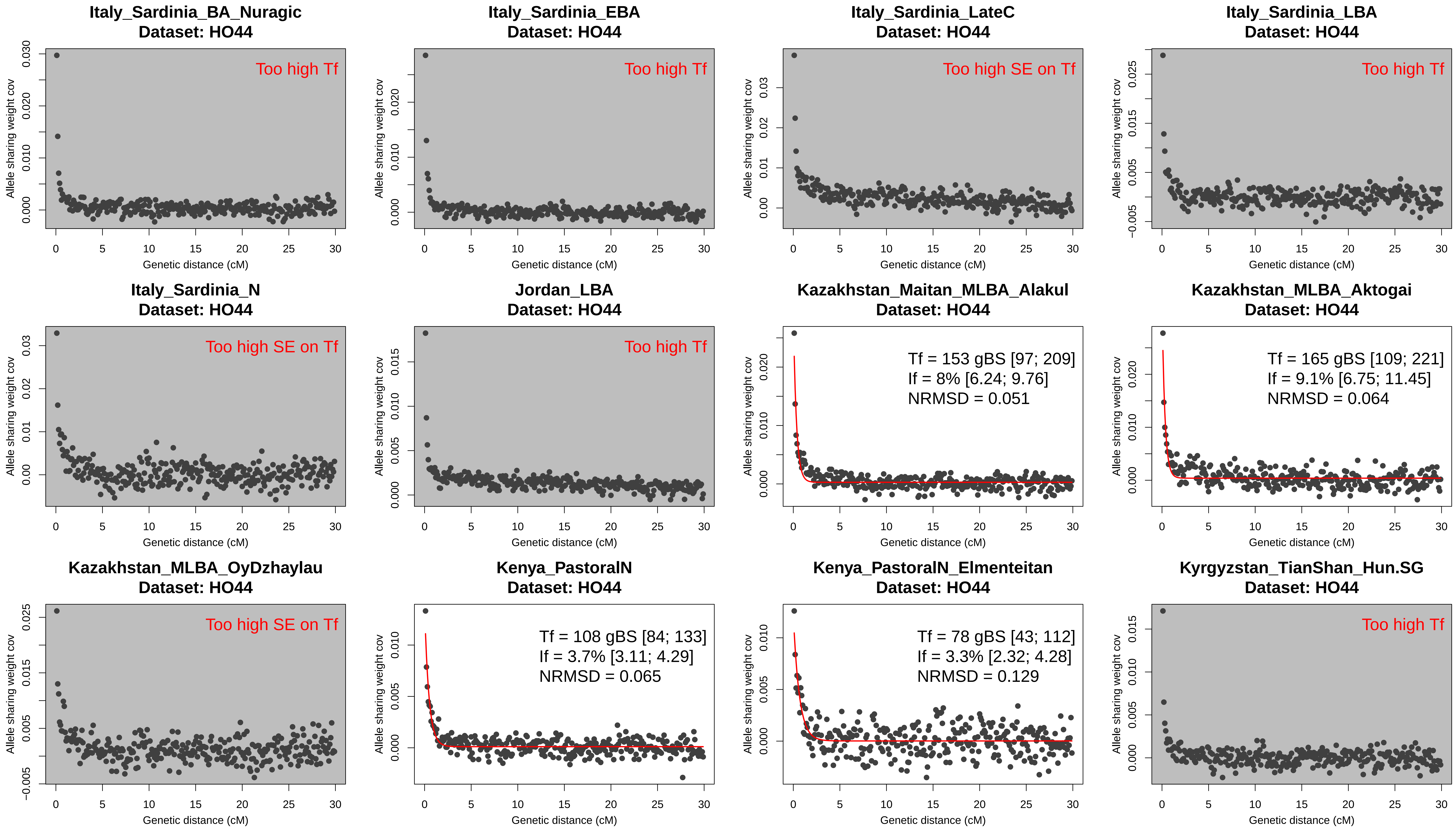
Italy_Sardinia_BA_Nuragic
Dataset: HO44
Too high Tf
Italy_Sardinia_EBA
Dataset: HO44
Too high Tf
Italy_Sardinia_LateC
Dataset: HO44
Too high SE on Tf
Italy_Sardinia_LBA
Dataset: HO44
Too high Tf
Italy_Sardinia_N
Dataset: HO44
Too high SE on Tf
Jordan_LBA
Dataset: HO44
Too high Tf
Kazakhstan_Maitan_MLBA_Alakul
Dataset: HO44
Tf = 153 gBS [97; 209]
If = 8% [6.24; 9.76]
NRMSD = 0.051
Kazakhstan_MLBA_Aktogai
Dataset: HO44
Tf = 165 gBS [109; 221]
If = 9.1% [6.75; 11.45]
NRMSD = 0.064
Kazakhstan_MLBA_OyDzhaylau
Dataset: HO44
Too high SE on Tf
Kenya_PastoralN
Dataset: HO44
Tf = 108 gBS [84; 133]
If = 3.7% [3.11; 4.29]
NRMSD = 0.065
Kenya_PastoralN_Elmenteitan
Dataset: HO44
Tf = 78 gBS [43; 112]
If = 3.3% [2.32; 4.28]
NRMSD = 0.129
Kyrgyzstan_TianShan_Hun.SG
Dataset: HO44
Too high Tf
Allele sharing weight cov
Genetic distance (cM)

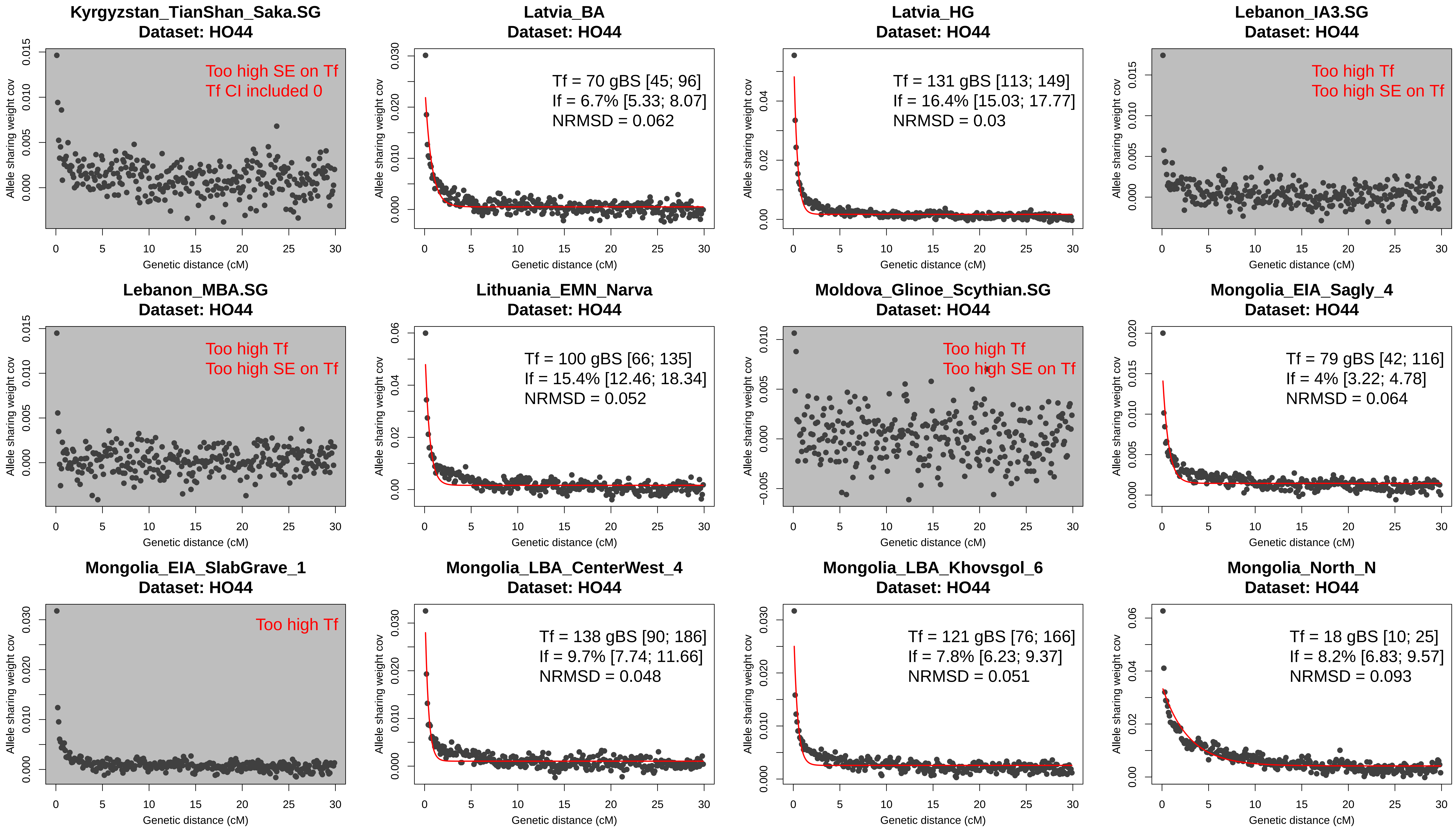
Kyrgyzstan_TianShan_Saka.SG
Dataset: HO44
Too high SE on Tf
Tf CI included 0
Latvia_BA
Dataset: HO44
Tf = 70 gBS [45; 96]
If = 6.7% [5.33; 8.07]
NRMSD = 0.062
Latvia_HG
Dataset: HO44
Tf = 131 gBS [113; 149]
If = 16.4% [15.03; 17.77]
NRMSD = 0.03
Lebanon_IA3.SG
Dataset: HO44
Too high Tf
Too high SE on Tf
Lebanon_MBA.SG
Dataset: HO44
Too high Tf
Too high SE on Tf
Lithuania_EMN_Narva
Dataset: HO44
Tf = 100 gBS [66; 135]
If = 15.4% [12.46; 18.34]
NRMSD = 0.052
Moldova_Glinoe_Scythian.SG
Dataset: HO44
Too high Tf
Too high SE on Tf
Mongolia_EIA_Sagly_4
Dataset: HO44
Tf = 79 gBS [42; 116]
If = 4% [3.22; 4.78]
NRMSD = 0.064
Mongolia_EIA_SlabGrave_1
Dataset: HO44
Too high Tf
Mongolia_LBA_CenterWest_4
Dataset: HO44
Tf = 138 gBS [90; 186]
If = 9.7% [7.74; 11.66]
NRMSD = 0.048
Mongolia_LBA_Khovsgol_6
Dataset: HO44
Tf = 121 gBS [76; 166]
If = 7.8% [6.23; 9.37]
NRMSD = 0.051
Mongolia_North_N
Dataset: HO44
Tf = 18 gBS [10; 25]
If = 8.2% [6.83; 9.57]
NRMSD = 0.093
Allele sharing weight cov
Genetic distance (cM)

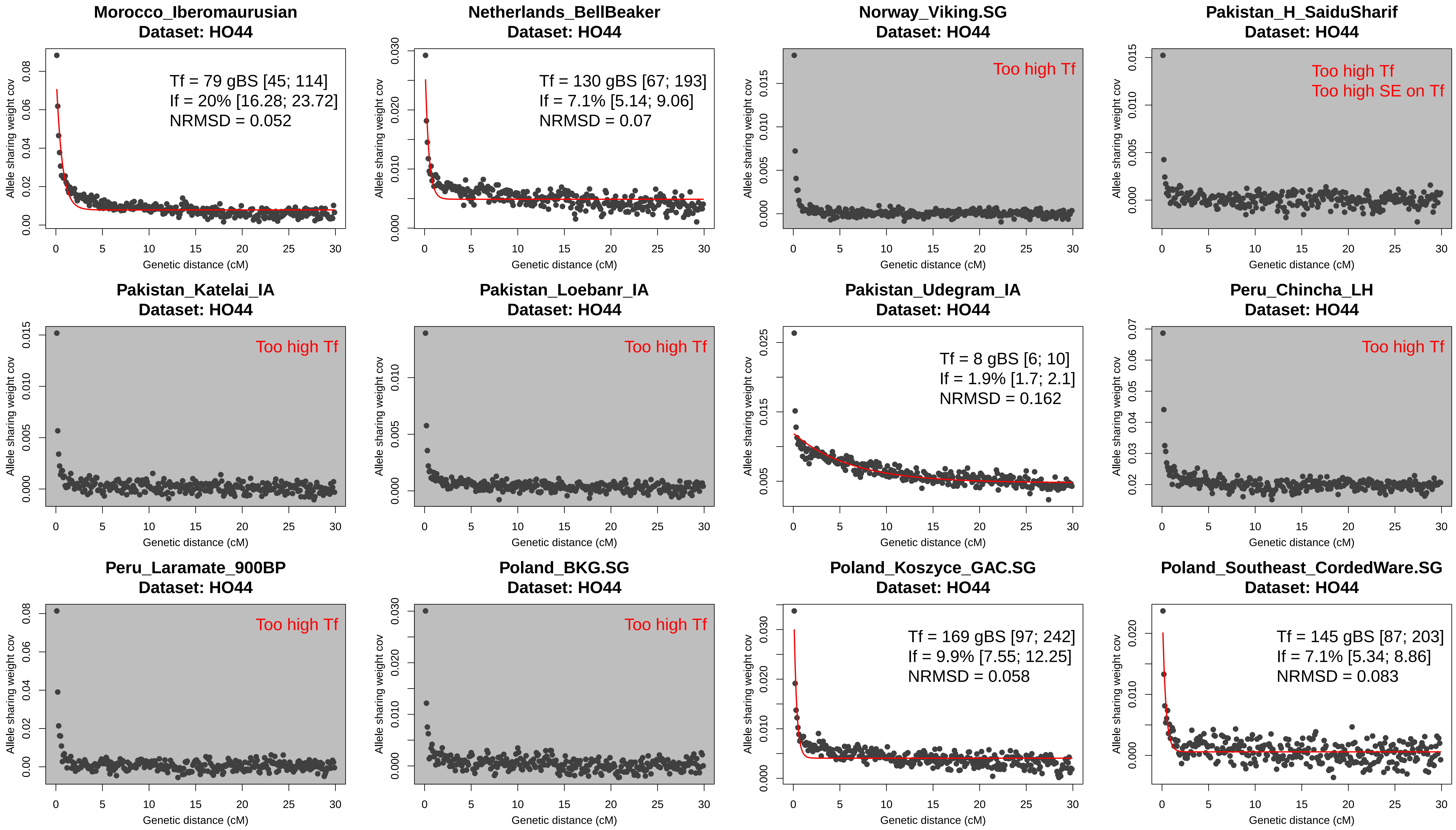

Morocco_Iberomaurusian
Dataset: HO44
Tf = 79 gBS [45; 114]
If = 20% [16.28; 23.72]
NRMSD = 0.052
Netherlands_BellBeaker
Dataset: HO44
Tf = 130 gBS [67; 193]
If = 7.1% [5.14; 9.06]
NRMSD = 0.07
Norway_Viking.SG
Dataset: HO44
Too high Tf
Pakistan_H_SaiduSharif
Dataset: HO44
Too high Tf
Too high SE on Tf
Pakistan_Katelai_IA
Dataset: HO44
Too high Tf
Pakistan_Loebanr_IA
Dataset: HO44
Too high Tf
Pakistan_Udegram_IA
Dataset: HO44
Tf = 8 gBS [6; 10]
If = 1.9% [1.7; 2.1]
NRMSD = 0.162
Peru_Chincha_LH
Dataset: HO44
Too high Tf
Peru_Laramate_900BP
Dataset: HO44
Too high Tf
Poland_BKG.SG
Dataset: HO44
Too high Tf
Poland_Koszyce_GAC.SG
Dataset: HO44
Tf = 169 gBS [97; 242]
If = 9.9% [7.55; 12.25]
NRMSD = 0.058
Poland_Southeast_CordedWare.SG
Dataset: HO44
Tf = 145 gBS [87; 203]
If = 7.1% [5.34; 8.86]
NRMSD = 0.083
Allele sharing weight cov
Genetic distance (cM)

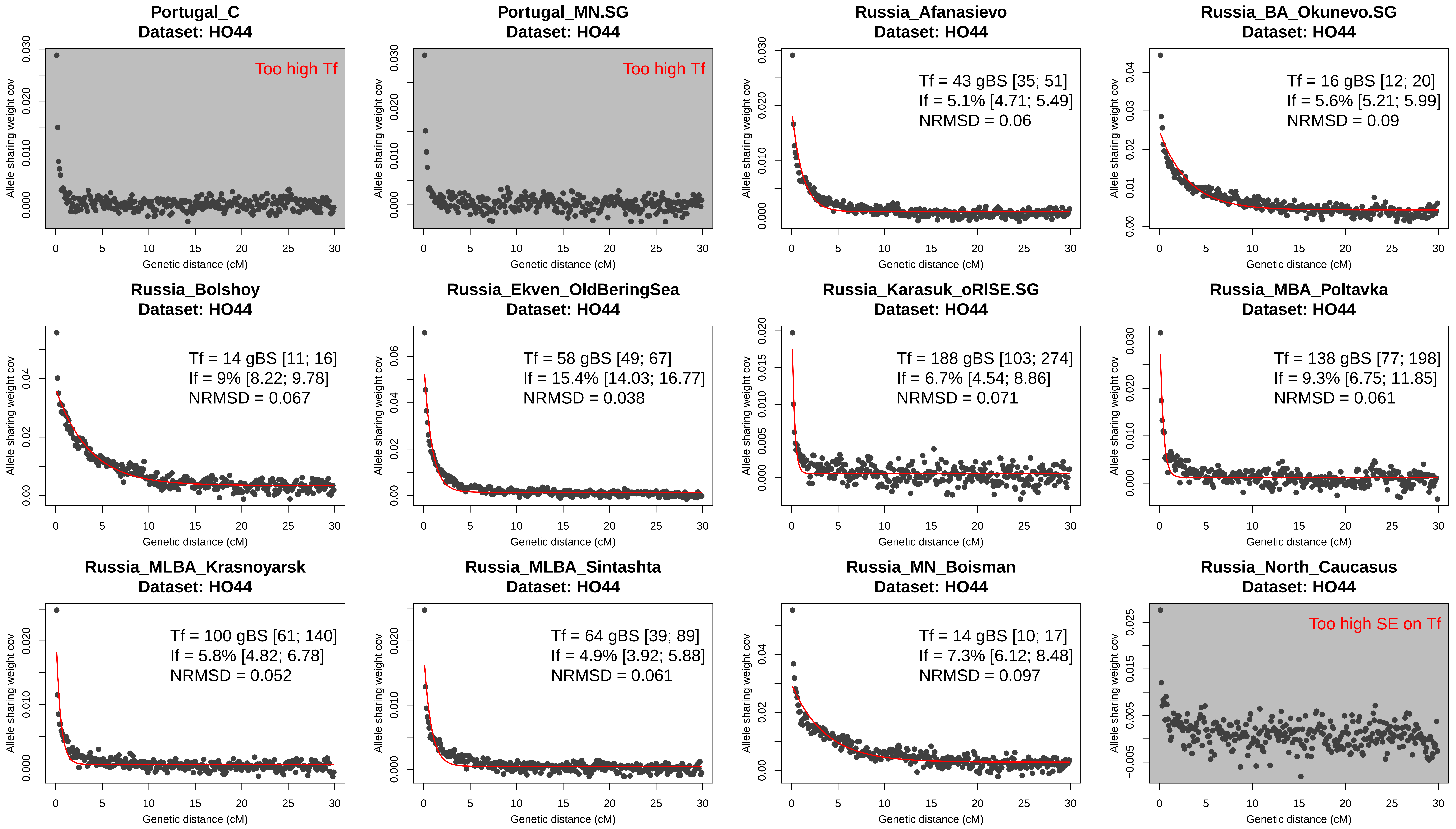
Portugal_C
Dataset: HO44
Too high Tf
Portugal_MN.SG
Dataset: HO44
Too high Tf
Russia_Afanasievo
Dataset: HO44
Tf = 43 gBS [35; 51]
If = 5.1% [4.71; 5.49]
NRMSD = 0.06
Russia_BA_Okunevo.SG
Dataset: HO44
Tf = 16 gBS [12; 20]
If = 5.6% [5.21; 5.99]
NRMSD = 0.09
Russia_Bolshoy
Dataset: HO44
Tf = 14 gBS [11; 16]
If = 9% [8.22; 9.78]
NRMSD = 0.067
Russia_Ekven_OldBeringSea
Dataset: HO44
Tf = 58 gBS [49; 67]
If = 15.4% [14.03; 16.77]
NRMSD = 0.038
Russia_Karasuk_oRISE.SG
Dataset: HO44
Tf = 188 gBS [103; 274]
If = 6.7% [4.54; 8.86]
NRMSD = 0.071
Russia_MBA_Poltavka
Dataset: HO44
Tf = 138 gBS [77; 198]
If = 9.3% [6.75; 11.85]
NRMSD = 0.061
Russia_MLBA_Krasnoyarsk
Dataset: HO44
Tf = 100 gBS [61; 140]
If = 5.8% [4.82; 6.78]
NRMSD = 0.052
Russia_MLBA_Sintashta
Dataset: HO44
Tf = 64 gBS [39; 89]
If = 4.9% [3.92; 5.88]
NRMSD = 0.061
Russia_MN_Boisman
Dataset: HO44
Tf = 14 gBS [10; 17]
If = 7.3% [6.12; 8.48]
NRMSD = 0.097
Russia_North_Caucasus
Dataset: HO44
Too high SE on Tf
Allele sharing weight cov
Genetic distance (cM)

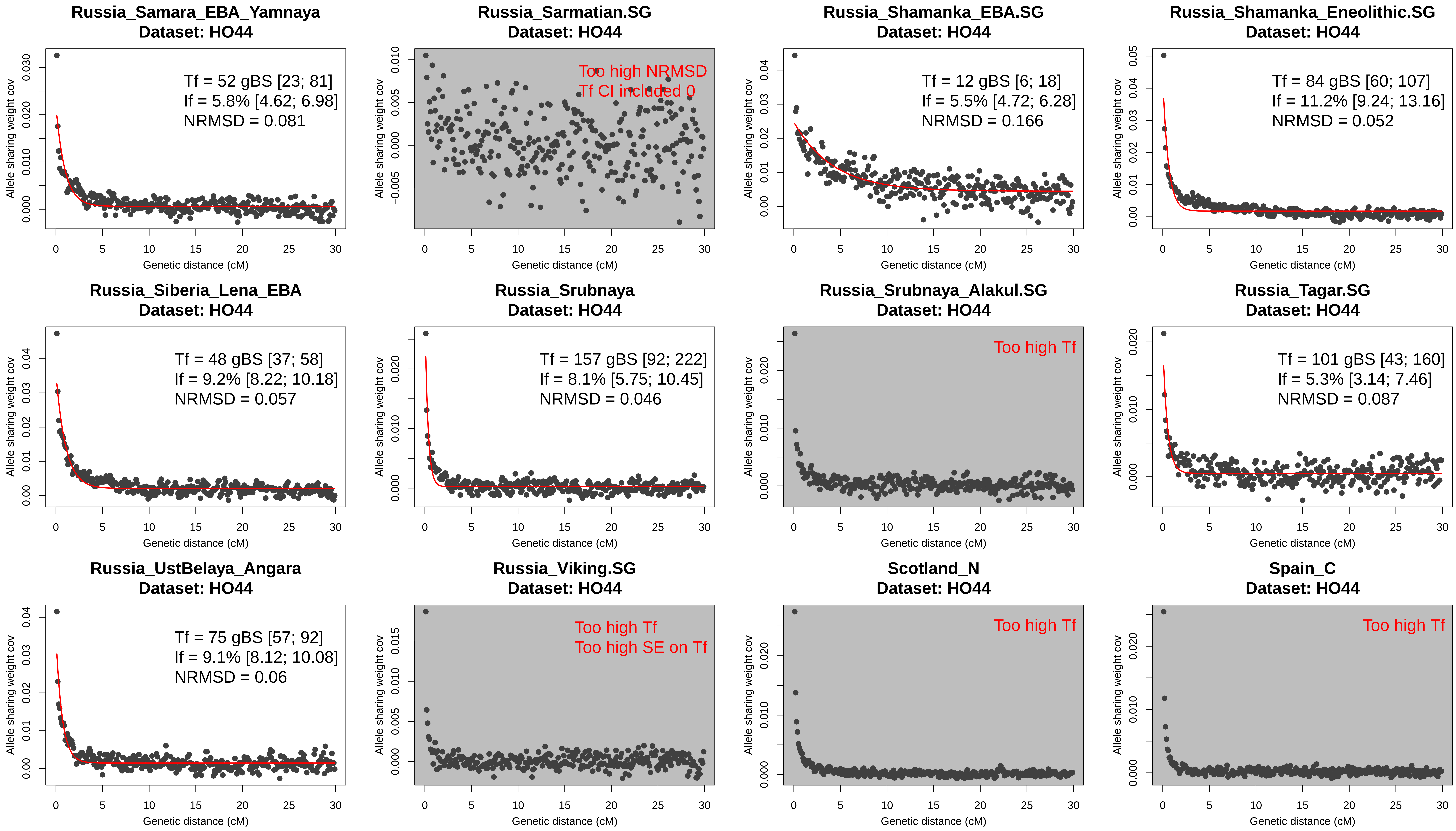
Russia_Samara_EBA_Yamnaya
Dataset: HO44
Tf = 52 gBS [23; 81]
If = 5.8% [4.62; 6.98]
NRMSD = 0.081
Russia_Sarmatian.SG
Dataset: HO44
Too high NRMSD
Tf CI included 0
Russia_Shamanka_EBA.SG
Dataset: HO44
Tf = 12 gBS [6; 18]
If = 5.5% [4.72; 6.28]
NRMSD = 0.166
Russia_Shamanka_Eneolithic.SG
Dataset: HO44
Tf = 84 gBS [60; 107]
If = 11.2% [9.24; 13.16]
NRMSD = 0.052
Russia_Siberia_Lena_EBA
Dataset: HO44
Tf = 48 gBS [37; 58]
If = 9.2% [8.22; 10.18]
NRMSD = 0.057
Russia_Srubnaya
Dataset: HO44
Tf = 157 gBS [92; 222]
If = 8.1% [5.75; 10.45]
NRMSD = 0.046
Russia_Srubnaya_Alakul.SG
Dataset: HO44
Too high Tf
Russia_Tagar.SG
Dataset: HO44
Tf = 101 gBS [43; 160]
If = 5.3% [3.14; 7.46]
NRMSD = 0.087
Russia_UstBelaya_Angara
Dataset: HO44
Tf = 75 gBS [57; 92]
If = 9.1% [8.12; 10.08]
NRMSD = 0.06
Russia_Viking.SG
Dataset: HO44
Too high Tf
Too high SE on Tf
Scotland_N
Dataset: HO44
Too high Tf
Spain_C
Dataset: HO44
Too high Tf
Allele sharing weight cov
Genetic distance (cM)

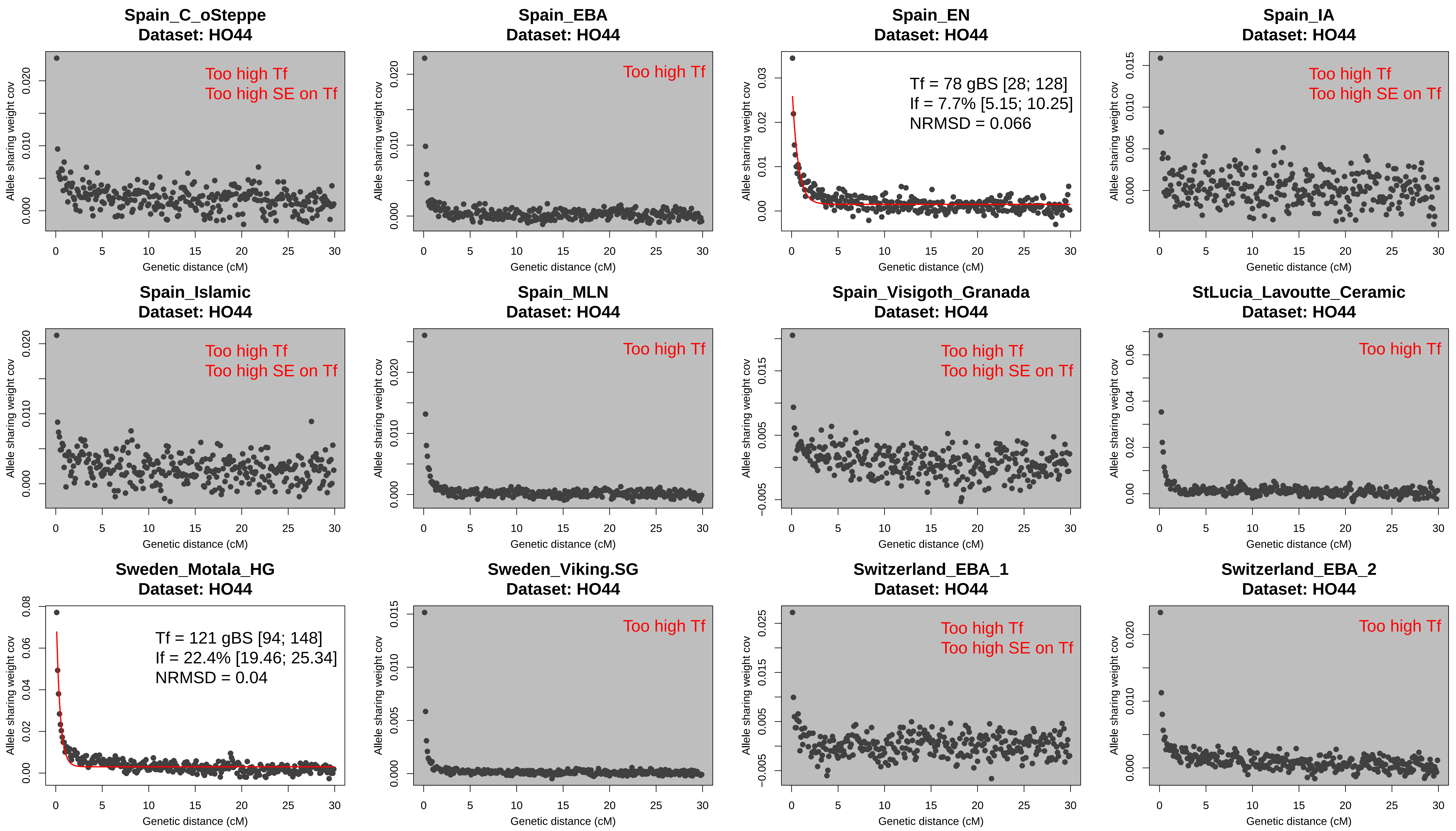
Spain_C_oSteppe
Dataset: HO44
Too high Tf
Too high SE on Tf
Spain_EBA
Dataset: HO44
Too high Tf
Spain_EN
Dataset: HO44
Tf = 78 gBS [28; 128]
If = 7.7% [5.15; 10.25]
NRMSD = 0.066
Spain_IA
Dataset: HO44
Too high Tf
Too high SE on Tf
Spain_Islamic
Dataset: HO44
Too high Tf
Too high SE on Tf
Spain_MLN
Dataset: HO44
Too high Tf
Spain_Visigoth_Granada
Dataset: HO44
Too high Tf
Too high SE on Tf
StLucia_Lavoutte_Ceramic
Dataset: HO44
Too high Tf
Sweden_Motala_HG
Dataset: HO44
Tf = 121 gBS [94; 148]
If = 22.4% [19.46; 25.34]
NRMSD = 0.04
Sweden_Viking.SG
Dataset: HO44
Too high Tf
Switzerland_EBA_1
Dataset: HO44
Too high Tf
Too high SE on Tf
Switzerland_EBA_2
Dataset: HO44
Too high Tf
Allele sharing weight cov
Genetic distance (cM)

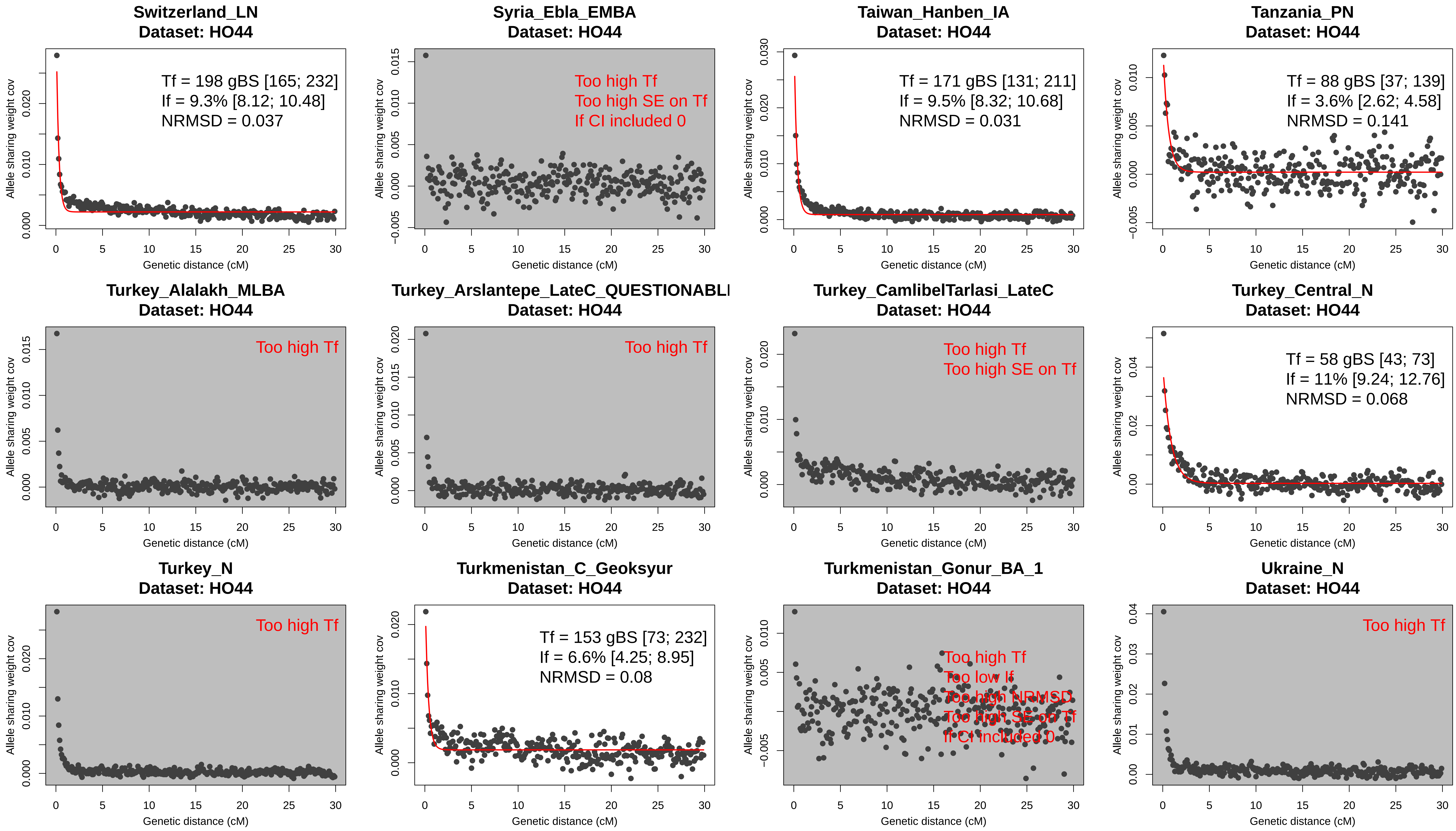

Switzerland_LN
Dataset: HO44
Tf = 198 gBS [165; 232]
If = 9.3% [8.12; 10.48]
NRMSD = 0.037
Allele sharing weight cov
Genetic distance (cM)
Syria_Ebla_EMBA
Dataset: HO44
Too high Tf
Too high SE on Tf
If CI included 0
Allele sharing weight cov
Genetic distance (cM)
Taiwan_Hanben_IA
Dataset: HO44
Tf = 171 gBS [131; 211]
If = 9.5% [8.32; 10.68]
NRMSD = 0.031
Allele sharing weight cov
Genetic distance (cM)
Tanzania_PN
Dataset: HO44
Tf = 88 gBS [37; 139]
If = 3.6% [2.62; 4.58]
NRMSD = 0.141
Allele sharing weight cov
Genetic distance (cM)
Turkey_Alalakh_MLBA
Dataset: HO44
Too high Tf
Allele sharing weight cov
Genetic distance (cM)
Turkey_Arslantepe_LateC_QUESTIONABLI
Dataset: HO44
Too high Tf
Allele sharing weight cov
Genetic distance (cM)
Turkey_CamlibelTarlasi_LateC
Dataset: HO44
Too high Tf
Too high SE on Tf
Allele sharing weight cov
Genetic distance (cM)
Turkey_Central_N
Dataset: HO44
Tf = 58 gBS [43; 73]
If = 11% [9.24; 12.76]
NRMSD = 0.068
Allele sharing weight cov
Genetic distance (cM)
Turkey_N
Dataset: HO44
Too high Tf
Allele sharing weight cov
Genetic distance (cM)
Turkmenistan_C_Geoksyur
Dataset: HO44
Tf = 153 gBS [73; 232]
If = 6.6% [4.25; 8.95]
NRMSD = 0.08
Allele sharing weight cov
Genetic distance (cM)
Turkmenistan_Gonur_BA_1
Dataset: HO44
Too high Tf
Too low If
Too high NRMSD
Too high SE on Tf
If CI included 0
Allele sharing weight cov
Genetic distance (cM)
Ukraine_N
Dataset: HO44
Too high Tf
Allele sharing weight cov
Genetic distance (cM)

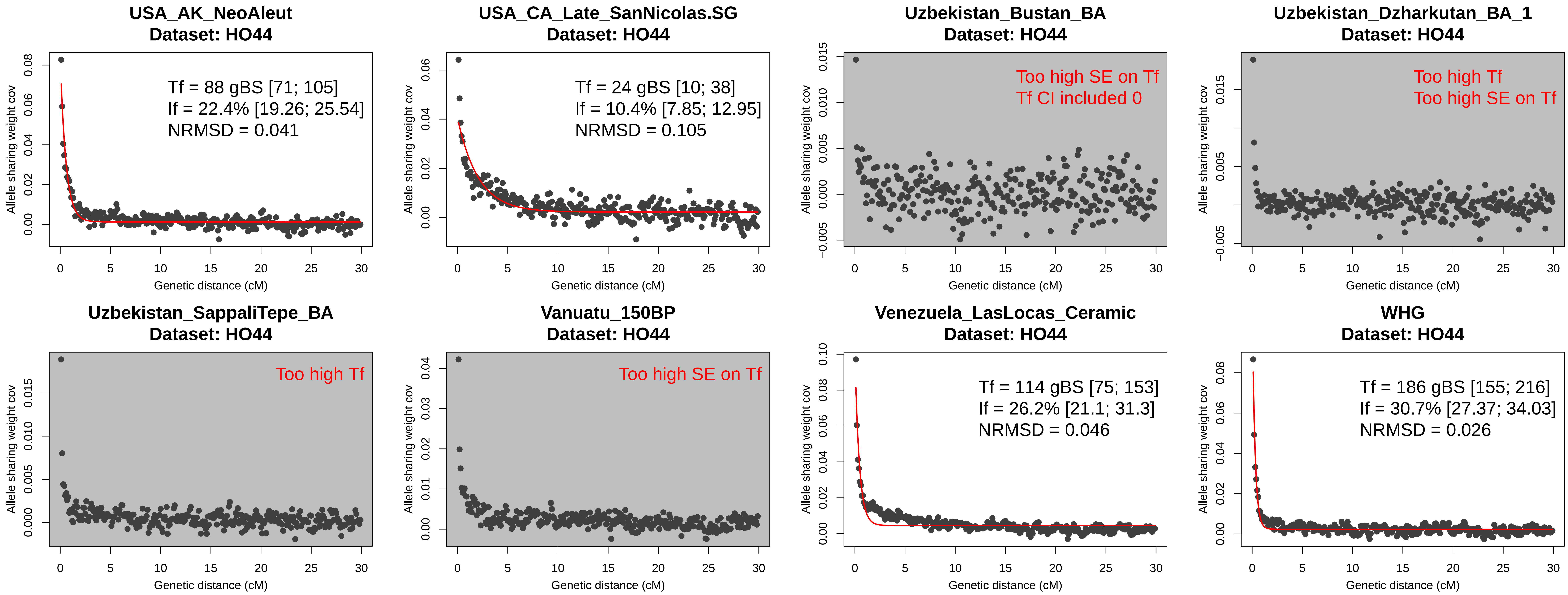
USA_AK_NeoAleut
Dataset: HO44
Tf = 88 gBS [71; 105]
If = 22.4% [19.26; 25.54]
NRMSD = 0.041
USA_CA_Late_SanNicolas.SG
Dataset: HO44
Tf = 24 gBS [10; 38]
If = 10.4% [7.85; 12.95]
NRMSD = 0.105
Uzbekistan_Bustan_BA
Dataset: HO44
Too high SE on Tf
Tf CI included 0
Uzbekistan_Dzharkutan_BA_1
Dataset: HO44
Too high Tf
Too high SE on Tf
Uzbekistan_SappaliTepe_BA
Dataset: HO44
Too high Tf
Vanuatu_150BP
Dataset: HO44
Too high SE on Tf
Venezuela_LasLocas_Ceramic
Dataset: HO44
Tf = 114 gBS [75; 153]
If = 26.2% [21.1; 31.3]
NRMSD = 0.046
WHG
Dataset: HO44
Tf = 186 gBS [155; 216]
If = 30.7% [27.37; 34.03]
NRMSD = 0.026
Allele sharing weight cov
Genetic distance (cM)
