# Supplementary material for: Reconstructing the history of founder events using genome-wide patterns of allele sharing across individuals
Source: PLoS Genet. 2022 Jun 23;18(6):e1010243. doi: 10.1371/journal.pgen.1010243 (PMC9223333; doi:10.1371/journal.pgen.1010243)
Supplement: S7 Fig — We represented the distribution of the radiocarbon ages for the surveyed ancient populations from the HO44 dataset (gray histogram) as well as the distribution of the founder ages estimated across populations with evidence of founder event (yellow histogram). The X-axis is in years BP. The founder ages reported in the plot were converted from generations to years before present by using a generation time of 28 years [29,30]) and by adding the radiocarbon sample date. Despite a higher population sampling towards the present (left), we observe a shift of the founder age distribution towards the more distant past (right). (PDF) [file pgen.1010243.s007.pdf]

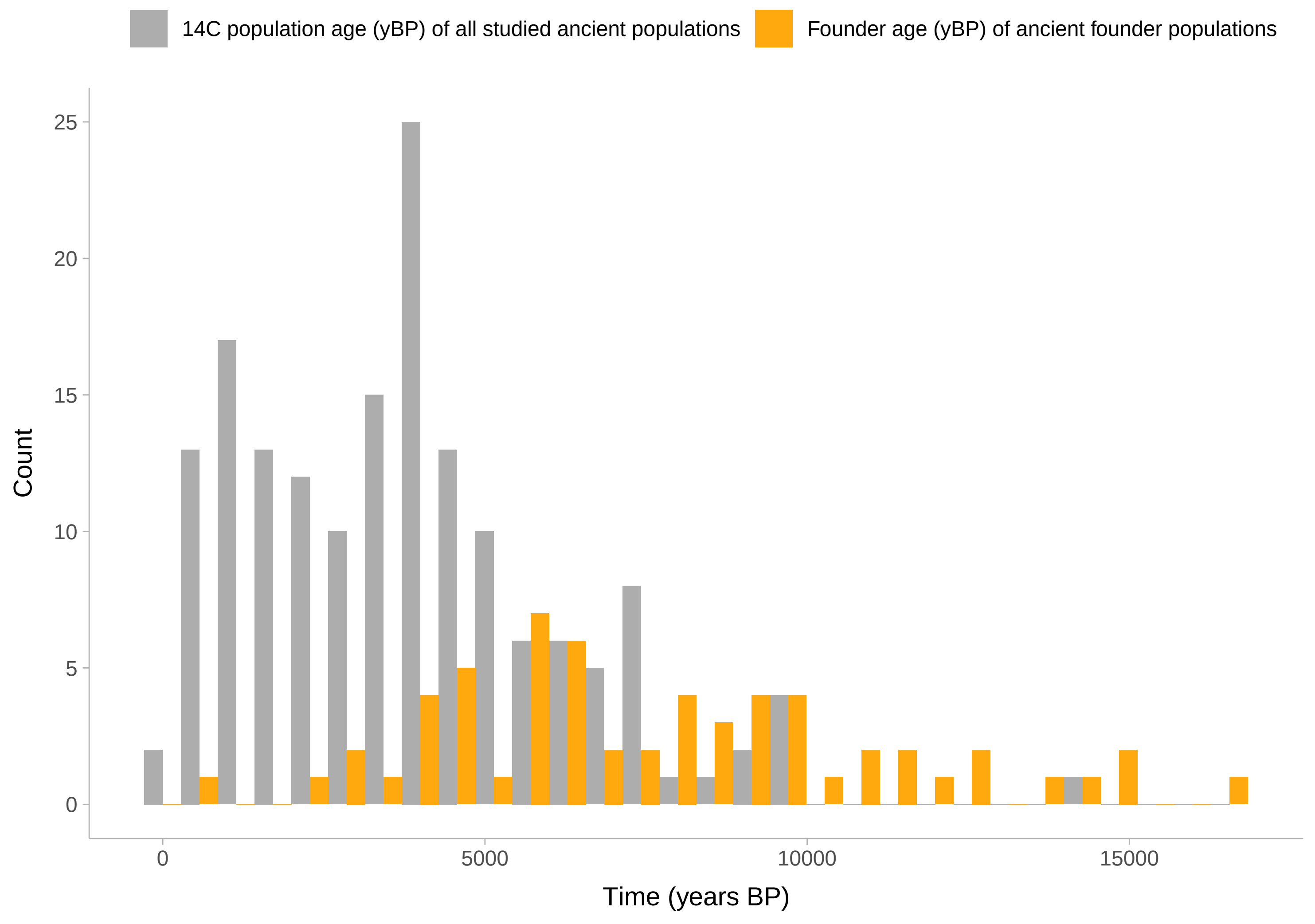
14C population age (yBP) of all studied ancient populations
Founder age (yBP) of ancient founder populations
Count
25
20
15
10
5
0
0
5000
10000
15000
Time (years BP)
