# Supplementary material for: Reconstructing the history of founder events using genome-wide patterns of allele sharing across individuals
Source: PLoS Genet. 2022 Jun 23;18(6):e1010243. doi: 10.1371/journal.pgen.1010243 (PMC9223333; doi:10.1371/journal.pgen.1010243)
Supplement: S1 Text — (PDF) [file pgen.1010243.s014.pdf]

Supplementary Online Materials

Reconstructing the history of founder events using genome-wide patterns of allele sharing across individuals

Rémi Tournebize, Gillian Chu, Priya Moorjani

S1. Implementation of *ASCEND* 1

S2. Simulations to test the performance of *ASCEND* 9

- **S2.1 Single-generation epoch model** 9
- **S2.2 Multi-generation epoch model** 13
- **S2.3 Two-epoch bottleneck model** 15
- **S2.4 Model with founder event and admixture** 17
  - **S2.4.1 Admixture occurred *before* the population bottleneck** 17
  - **S2.4.2 Admixture occurred *after* the population bottleneck** 20
- **S2.5 Gradual exponential growth model** 23
- **S2.6 No recovery founder event model** 25
- **S2.7 Robustness of the inference to data quality issues** 27
  - **S2.7.1 Impact of sample size** 27
  - **S2.7.2 Impact of missing data** 29
  - **S2.7.3 Impact of ancient DNA data features** 31
  - **S2.7.4 Impact of pseudo-haploid genotypes** 34
- **S2.8 Null model: No recent founder event in the target population** 37
- **S2.9. Comparison between Naive and FFT implementation** 39
- **S2.10. *msprime* commands** 40

S3. Data curation for human datasets 47

S4. Comparison of overlapping groups across human datasets 49

S5. Comparison of our results with published estimates 51

- **S5.1 Comparison of founder ages with Reich *et al.* (2009)** 51
- **S5.2. Comparison of founder intensity and IBD scores from Nakatsuka et al. (2017)** 56
- **S5.3. *IBDNe* analysis** 59

S6. History of founder events in dogs 62

S7. Reference 64

S1. Implementation of *ASCEND*

We implemented *ASCEND* (Allele Sharing Correlation for the Estimation of Nonequilibrium Demography) in two ways: a Naive approach and a Fast Fourier Transform (FFT) approach. We illustrate these two methods with an example containing three SNPs (say, s_1, s_2 and s_3) and three individuals (referred to as, A, B and C).

S1.1 Naive implementation of *ASCEND*

In the Naive implementation, we first construct an allele sharing matrix whose elements are counts (c_i^π) of the alleles shared between a pair π of individuals at each SNP i. Specifically, this is a $N \times P$ matrix, where N is the number of SNPs and P is the number of individual pairs.

$$\begin{array}{c} \\ s_1 \\ s_2 \\ s_3 \end{array} \begin{array}{c} \begin{array}{ccc} A,B & A,C & B,C \end{array} \\ \begin{bmatrix} c_1^\alpha & c_1^\beta & c_1^\gamma \\ c_2^\alpha & c_2^\beta & c_2^\gamma \\ c_3^\alpha & c_3^\beta & c_3^\gamma \end{bmatrix} \end{array}$$

For simplicity, we consider that the three SNPs are equally spaced, $d = 10^{-5}$ Morgans apart, with the genetic positions of s_1, s_2 and s_3 as $1 \cdot 10^{-5}$, $2 \cdot 10^{-5}$ and $3 \cdot 10^{-5}$ Morgans respectively.

The Naive implementation loops over the SNP pairs, and calculates the following equation:

$$\boxed{z(d) = \frac{1}{|S_d|} \sum_{S_d} r([c_i^\alpha, c_i^\beta, ...], [c_j^\alpha, c_j^\beta, ...])} \tag{1}$$

where S_d is the set of SNP pairs, located d Morgans apart, $|S_d|$ is the size of this set. To simplify the computations, we standardize the allele sharing vectors:

$$s_i' = \frac{s_i - \bar{s}_i}{\sigma_{s_i}} \tag{2}$$

And we then compute the Pearson's population correlation coefficient as follows, considering the genetic distance bin $[1 \cdot 10^{-5}; 3 \cdot 10^{-5}]$ Morgans for our application:

$$\begin{aligned} z &= \frac{1}{3} \cdot \Big(r(s'_1, s'_2) + r(s'_1, s'_3) + r(s'_2, s'_3) \Big) \\ &= \frac{1}{3} \cdot \Big(\frac{cov(s'_1, s'_2)}{\sigma_{s'_1} \sigma_{s'_2}} + \frac{cov(s'_1, s'_3)}{\sigma_{s'_1} \sigma_{s'_3}} + \frac{cov(s'_2, s'_3)}{\sigma_{s'_2} \sigma_{s'_3}} \Big) \end{aligned} \tag{3}$$

Since the s'_i vectors are standardized, i.e., with mean = 0 and variance = 1, this equation reduces to:

$$\begin{aligned} z &= \frac{1}{3} \cdot \Big(cov(s'_1, s'_2) + cov(s'_1, s'_3) + cov(s'_2, s'_3) \Big) \\ &= \frac{1}{3} \cdot \Big(\frac{\sum s'_1 s'_2}{3} - \bar{s'_1} \bar{s'_2} + \frac{\sum s'_1 s'_3}{3} - \bar{s'_1} \bar{s'_3} + \frac{\sum s'_2 s'_3}{3} - \bar{s'_2} \bar{s'_3} \Big) \\ &= \frac{1}{9} \cdot \Big(\sum s'_1 s'_2 + \sum s'_1 s'_3 + \sum s'_2 s'_3 \Big) \end{aligned} \tag{4}$$

Thus:

$$\boxed{9z = (c_1^{\alpha'} c_2^{\alpha'} + c_1^{\beta'} c_2^{\beta'} + c_1^{\gamma'} c_2^{\gamma'}) + (c_1^{\alpha'} c_3^{\alpha'} + c_1^{\beta'} c_3^{\beta'} + c_1^{\gamma'} c_3^{\gamma'}) + (c_2^{\alpha'} c_3^{\alpha'} + c_2^{\beta'} c_3^{\beta'} + c_2^{\gamma'} c_3^{\gamma'})} \tag{5}$$

S1.2 FFT implementation of *ASCEND*

In brief, the FFT approach provides a significant speedup to Naive implementation because it performs an FFT convolution to compute pairwise LD. To implement this, we calculate the autocorrelation of alleles shared between each pair of individuals along equally-spaced mesh points (the "grid mesh")

in the genome. The main implementation difference with the Naive implementation is the assignment of SNPs to these pre-defined mesh points, which we describe below.

S1.2.1 Grid mesh

The use of FFT in *ASCEND* requires dividing the genome into equally-spaced bins such that data points are distributed evenly located δ Morgans apart across the genome (Fig A). To improve accuracy, δ should be smaller than the genetic bin size d used in the Naive approach, i.e. $\delta = d/m$ where $m \in \mathbb{N}^*$ is defined as the number of grid points per genetic distance bin. Typically, δ is set to be very small, say 10^{-5} Morgans.

Each SNP, located at a particular genetic position g Morgans, is then assigned to the nearest mesh point with genetic position g_m so that $g \in [g_m; g_m + \delta[$. In the case where multiple SNPs map to the same mesh point g_m, only the SNP with the lowest genetic position is retained, the others are excluded (note that if δ is small, this exclusion would affect only a very minor proportion of the SNPs in the dataset). Given the density of markers, some regions $[g_m;\ g_m + \delta[$ may not contain any SNPs. These mesh regions are considered as "missing" and discussed in the next section.

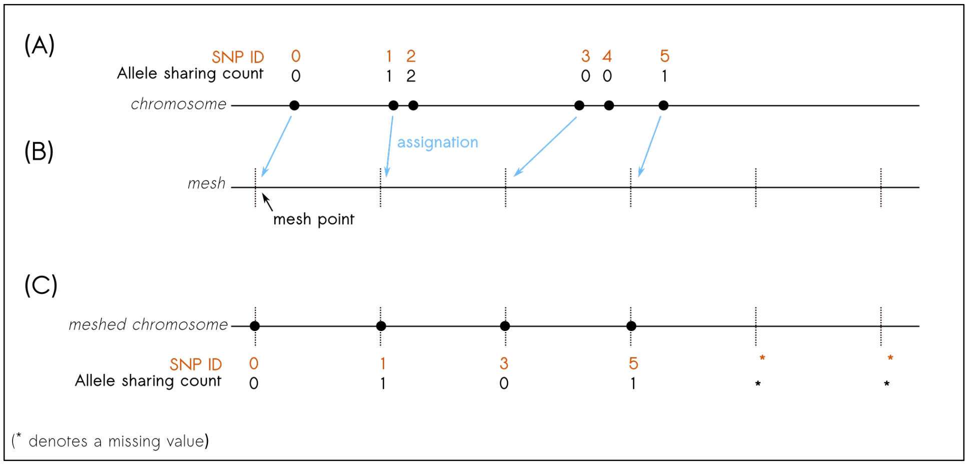

Fig A: **Representation of the mesh procedure.** **(A)** A chromosome segment with SNPs (with IDs 0, 1, 2, 3, 4, 5) and their associated allele sharing count for a given pair of individuals. **(B)** A mesh is placed upon the chromosome (vertical dotted lines) and each SNP is assigned to a mesh point (the first SNP is on the right of the mesh point). **(C)** The resulting "meshed chromosome" with the associated vector of allele sharing counts that will be used for the FFT computation. Comparison of (A) and (C) shows the difference between the non-meshed and meshed allele sharing vectors.

S1.2.2 Computing allele sharing correlation using FFT

In the FFT implementation, we loop over all possible mesh points and all pairs of individuals. In each loop, we estimate the autocorrelation of the allele sharing vector, instead of the Pearson's correlation coefficient as in the Naive implementation. Thus, $z(d)$ becomes:

$$\boxed{z(d) = \frac{1}{m\binom{n}{2}} \sum_{\tau=1}^{m} \sum_{\pi=1}^{\binom{n}{2}} r_{A_\pi A_\pi}(\tau)} \tag{6}$$

Where n is the number of individuals, m is the number of mesh points in the distance bin d, A_π is the vector of allele sharing for the pair of individuals indexed by π (the length of A_π is equal to the number of mesh points), and $r_{A_\pi A_\pi}(\tau)$ is the convolution function with a shift of τ Morgans. Note that

here we are summing over the convolution coefficients for the values of τ which leads to the mesh points being spaced by $[d; d + \Delta d[$. The convolution computation is performed in Python using the function *correlate* from SciPy v1.5.2 in *fft* mode.

Let us now consider the allele sharing matrix that was described earlier for the Naive approach. Since the SNPs in our toy example are equally spaced (at 10^{-5} Morgans intervals), they meet the criteria for FFT and hence we use them as mesh points for the computation of autocorrelation. Below we describe the transposed and standardized version of the allele sharing matrix for FFT:

$$\begin{array}{c} \\ A,B \\ A,C \\ B,C \end{array} \begin{array}{c} \begin{array}{ccc} s_1 & s_2 & s_3 \end{array} \\ \begin{bmatrix} c_1^{\alpha\prime} & c_2^{\alpha\prime} & c_3^{\alpha\prime} \\ c_1^{\beta\prime} & c_2^{\beta\prime} & c_3^{\beta\prime} \\ c_1^{\gamma\prime} & c_2^{\gamma\prime} & c_3^{\gamma\prime} \end{bmatrix} \end{array}$$

The sum of autocorrelation coefficients over all individual pairs is calculated as such:

$$\begin{aligned} &\text{For } \tau = 1,\ z = \frac{1}{3} \cdot \frac{1}{3}\Big((c_1^{\alpha\prime} c_2^{\alpha\prime} + c_2^{\alpha\prime} c_3^{\alpha\prime}) + (c_1^{\beta\prime} c_2^{\beta\prime} + c_2^{\beta\prime} c_3^{\beta\prime}) + (c_1^{\gamma\prime} c_2^{\gamma\prime} + c_2^{\gamma\prime} c_3^{\gamma\prime})\Big) \\ &\text{For } \tau = 2,\ z = \frac{1}{3} \cdot \frac{1}{3}\Big((c_1^{\alpha\prime} c_3^{\alpha\prime}) + (c_1^{\beta\prime} c_3^{\beta\prime}) + (c_1^{\gamma\prime} c_3^{\gamma\prime})\Big) \end{aligned} \tag{7}$$

Summing over the shifts τ and rearranging the terms, we get:

$$\boxed{9z = (c_1^{\alpha\prime} c_2^{\alpha\prime} + c_1^{\beta\prime} c_2^{\beta\prime} + c_1^{\gamma\prime} c_2^{\gamma\prime}) + (c_1^{\alpha\prime} c_3^{\alpha\prime} + c_1^{\beta\prime} c_3^{\beta\prime} + c_1^{\gamma\prime} c_3^{\gamma\prime}) + (c_2^{\alpha\prime} c_3^{\alpha\prime} + c_2^{\beta\prime} c_3^{\beta\prime} + c_2^{\gamma\prime} c_3^{\gamma\prime})} \tag{8}$$

This shows that, within the limits of resolution of the mesh grid, the FFT (Eq. 8) converges to the Naive implementation (Eq. 5). This identity is valid if and only if the SNPs are equally spaced. If not, then Eq. 8 is an approximation of Eq. 5. Note, Eq. 8 is asymptotically equal to Eq. 5 if m tends towards infinity. When the allele sharing value is missing for a

particular mesh point, we assign to it the value of 0 so that the convolution is equal to 0 and has no impact on the final summing of convolution coefficients for a given shift τ. However, when taking the average of the convolution coefficients for all τ within a particular genetic distance bin d, we make sure to not include these missing mesh points at the denominator so that the average autocorrelation of allele sharing is not biased.

Our analyses show that the FFT implementation can lead to runtime speed up by a hundred-fold, allowing the analysis of population genome-wide data in a few minutes, rather than hours using the Naive approach. We note that the smaller the mesh, the lower the approximation (but the higher the runtime and memory usage) and the closer the convergence between the Naive and FFT implementations. Empirically, we find that using a resolution of 10^{-3} cM leads to nearly equal allele sharing correlation values between the FFT and Naive implementations.

S1.3 Assessment of the fitted decay curves

The allele sharing correlation decay curves in empirical data can be noisy, either because of technical issues (such as dataset size) or population history (for example, there is a high variance in coalescent rates in case of long, weak bottlenecks). This can impede reliable inference of the founder event parameters as it is challenging to reliably fit an exponential model in noisy data.

In order to systematically assess if we can make reliable inference in real data, we calculated the Normalized Root-Mean-Square Deviation (NRMSD) between the empirical and fitted decay curves for each population. Specifically, we calculated the NRMSD as follows:

$$NRMSD = \frac{1}{max(\hat{z}) - min(\hat{z})} \sqrt{\frac{\sum^{D} (z_o - \hat{z})^2}{D}} \tag{9}$$

where z_o and $\hat{z}$ are the empirical allele sharing correlation and the fitted allele sharing correlation values for all genetic distances considered. D is the number of genetic distance bins.

We calculated NRMSD for each population of the present-day HO37 human populations and the distribution of these values is shown in Fig B. Based on visual inspection, we suggest that the value of NRMSD=0.29 at the right tail of the NRMSD distribution is an appropriate threshold above which the empirical decay curves become too noisy to be reliably interpreted (Fig C).

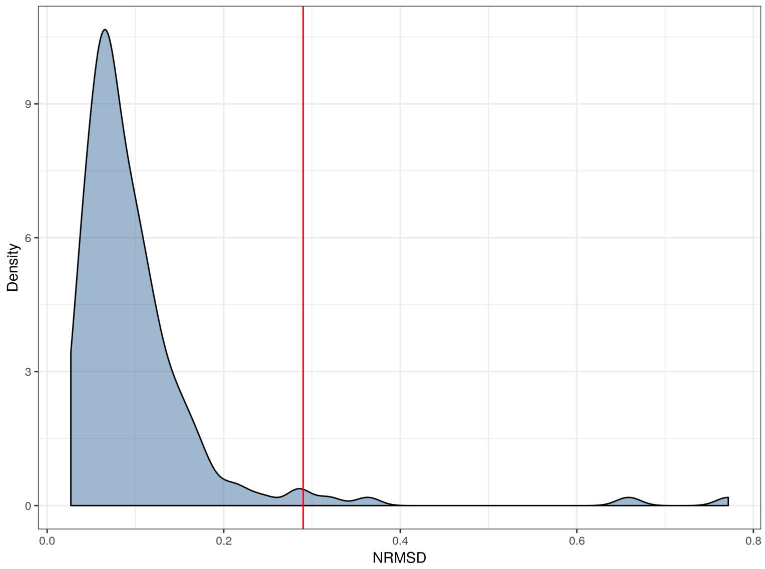

Fig B: Density of the NRMSD computed as the normalized residual between the empirical and fitted decay curves, for all the present-day HO37 human populations. To decide the threshold, we focused on present-day human populations as they have a better data quality than ancient DNA samples. The red vertical line represents the value NRMSD=0.29, which we used as the threshold to exclude populations from our analysis, because inspection of fitted curves above this threshold suggest the results are too noisy to make reliable inference (see Fig C below).

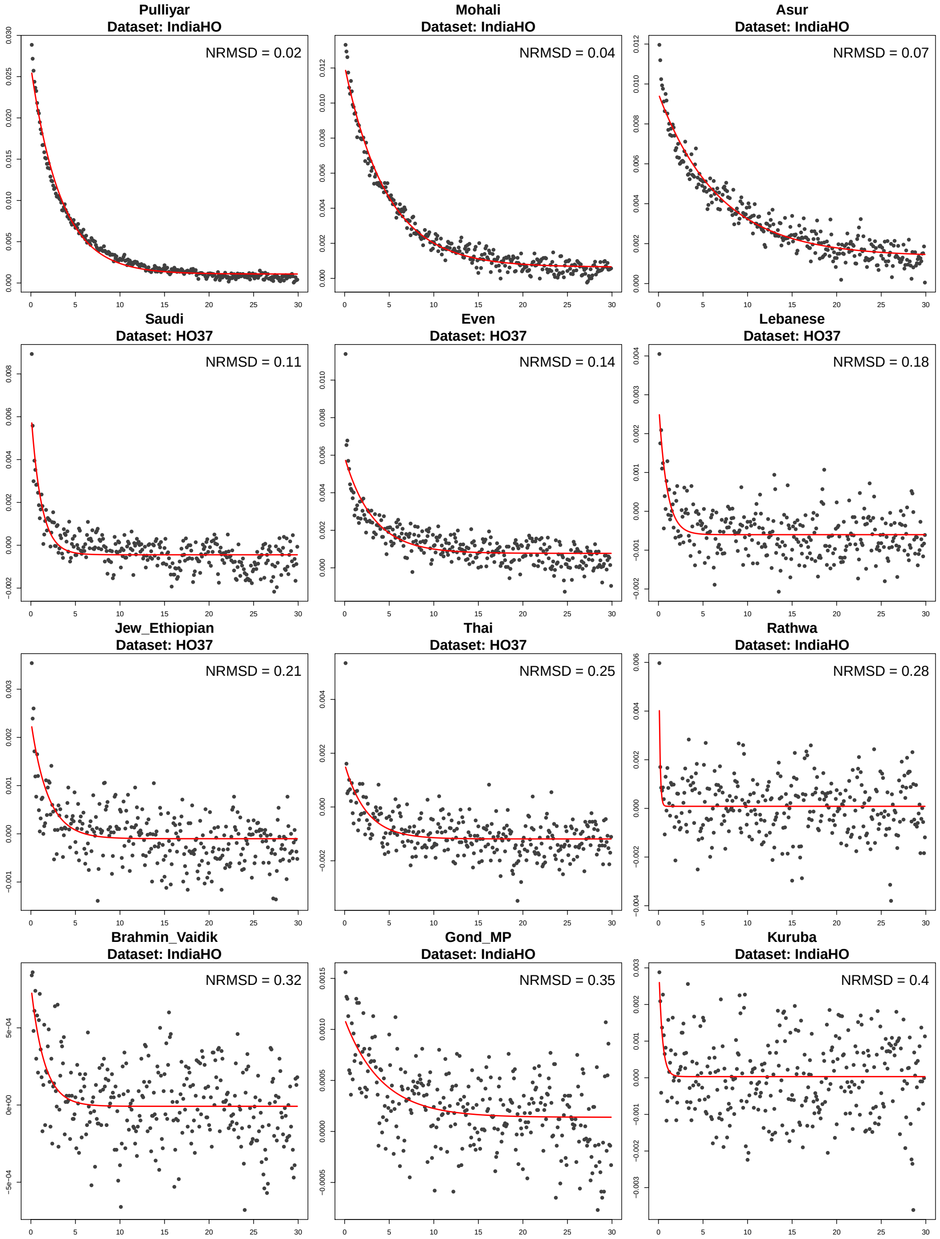

Fig C: Allele sharing correlation curves for selected populations with NRMSD values ranging from NRMSD=0.02 to 0.40. Based on visual inspection, we exclude populations with an NRMSD>0.29. The three plots on the last row have a NRMSD above this threshold and hence were excluded from further analysis.

S2. Simulations to test the performance of *ASCEND*

In order to characterize the accuracy of the method in estimating the age and intensity of founder events, we simulated datasets using *msprime* (Kelleher et al. 2016) under a variety of demographic models.

For all simulations, we generated data for two populations (target A, outgroup O) that diverged 1,800 generations ago (similar to Africans and Europeans) with effective population sizes of N_o=12,500 (Fig D). Unless stated otherwise, we simulated 20 chromosomes of size 50 megabases each for sample size of $n = 30$ haploid individuals in each population, assuming a mutation rate of 1.2×10^{-8} per base pair per generation (Jonsson et al. 2017) and a recombination rate of 1.0×10^{-8} per base pair per generation (Halldorsson et al. 2019). We assumed a uniform recombination rate across the chromosomes. We combined two haploid chromosomes at random to generate one diploid chromosome.

For the inference, unless stated otherwise, we computed $z_w(d)$ as the correlation between pairs of individuals in the target population A, and then subtracted the cross-population correlation $z_c(d)$ estimated across pairs of individuals in (A, O) to remove the effects of ancestral allele sharing (Fig D). We computed the correlation in windows of genetic distance of 0.1 cM, starting from 0.1 cM to 30 cM. For the FFT implementation, we set the number of sub-bins to 100. We report the estimated average T_f and I_f and the uncertainty based on chromosome jackknife, where one chromosome is dropped in each run (Methods).

S2.1 Single-generation epoch model

In the single generation epoch model, the target population A experienced a severe bottleneck happening T_f generations ago (ranging between 10 to 300 generations ago), such that the population size reduced to N_f=5 for a single generation. After T_f, the population recovered to the original population size of N_o (Fig D).

Applying *ASCEND* to this simulated dataset, we accurately recovered the age and intensity of the founder events that occurred up to 200 generations

ago (Fig E). We observed a slight underestimation of the dates beyond 200 generations, largely because the exponential fitting starts at 0.1 cM (to guard against fine-scale errors in the recombination map in real data (Sankaraman et al. 2012)) which can lead to lower resolution for older dates.

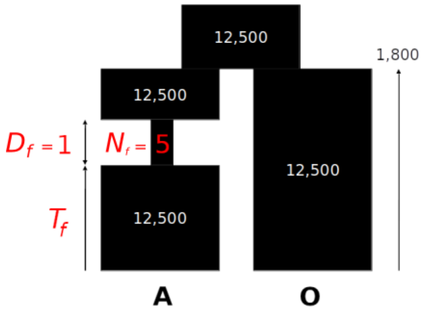

Fig D - Demographic model used for single-generation epoch model. In the figure, we show two simulated populations (A, O) that diverged 1,800 generations ago. The target population A experienced a severe bottleneck T_f generations ago, such that the population size reduced to N_f=5 for the duration D_f of a single generation.

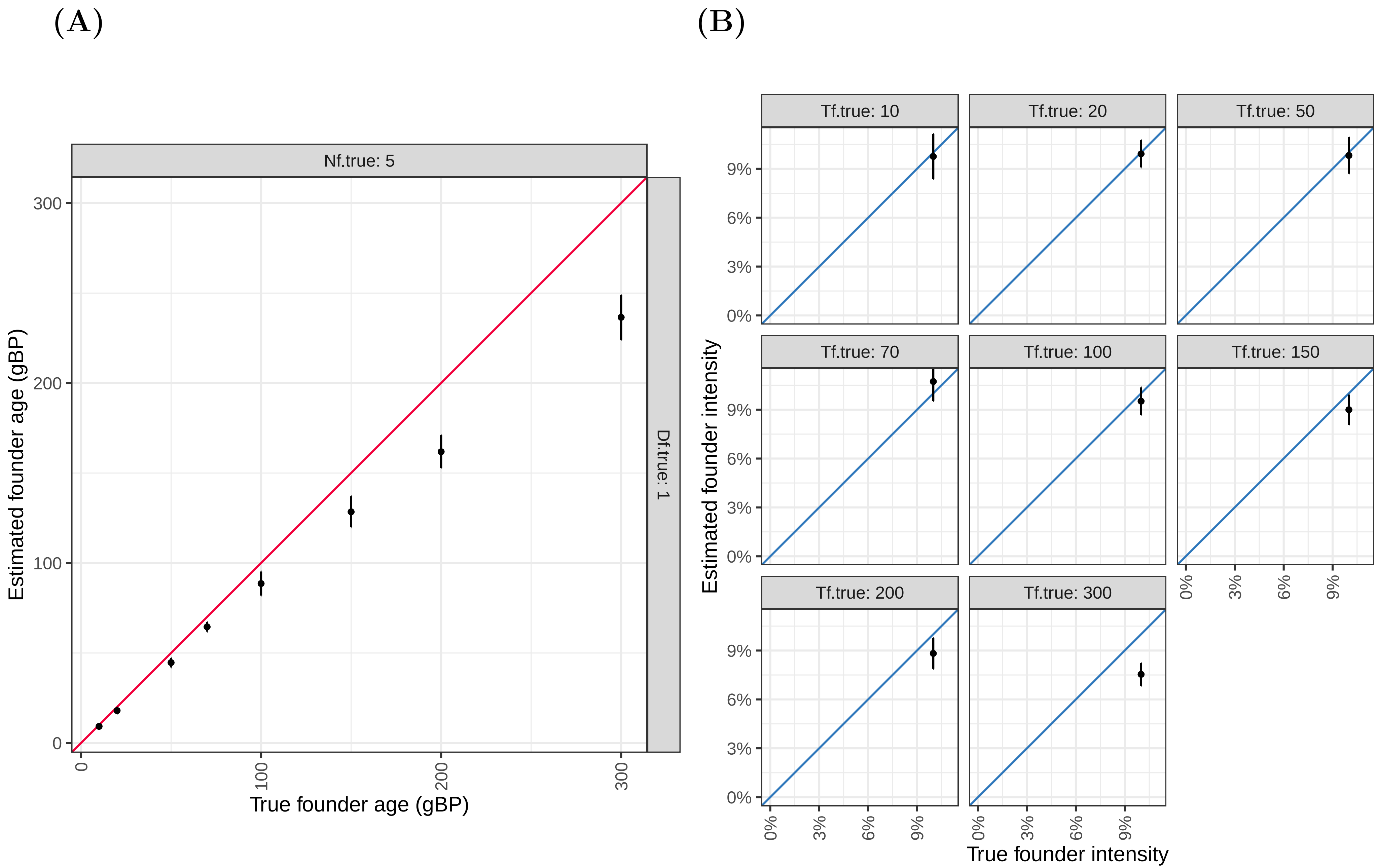

Fig E Performance of *ASCEND* for the single-generation epoch model: **(A)** Founder age T_f and **(B)** Founder intensity I_f.
The x-axis shows the true simulated parameter values and the y-axis shows the parameter values estimated by *ASCEND* (gBP = generations before present). The diagonal represents the expectation under the model. N_f refers to the population size during the bottleneck and D_f to the duration of the bottleneck.

We checked the stability of the inferred parameter estimates by performing 10 replicates for the same simulation history. We observed qualitatively similar results across all replicates (Table A and Table B).

Table A. Comparison of estimated **founder age** across 10 replicates of the same simulation model (single-generation epoch model).

Simulated founder age	95% CI for single replicate	95% CI for 10 replicates
10	8-10	9-10
150	120-138	122-134
300	227-248	222-244

Note: The true age is shown in column 1, the estimated 95% CI of founder age for a single simulation in shown in column 2 and the 2.5%-97.5% percentiles of the point estimates of founder age inferred from 10 replicates is shown in column 3.

Table B. Comparison of estimated **founder intensity** across 10 replicates of the same simulation model (single-generation epoch model).

Simulated founder age	95% CI for single replicate	95% CI for 10 replicates
10	8.5%-11.7%	8.5%-11.6%
150	8.4%-10.8%	9.1%-10.8%
300	7.5%-9.1%	7.8%-9.1%

Note: The simulated founder ages are shown in column 1, the estimated 95% CI of founder intensity for a single simulation in shown in column 2 and the 2.5%-97.5% percentiles of the point estimates of founder intensity inferred from 10 replicates is shown in column 3.

S2.2 Multi-generation epoch model

We simulated data for a population A that experienced a bottleneck for a duration D_f ranging from 10 to 30 generations (Fig F). During the bottleneck period, the population size reduced to N_f, ranging from 200 to 1,000.

Applying *ASCEND* to this dataset, we observed that the estimated age and intensity of the founder event was accurate up to 200 generations ago, even in case of less intense founder events where the population size was $N_f = 1,000$ (Fig G). However, we note that beyond 200 generations, *ASCEND* underestimated the age of the founder event. The estimated intensity appeared to be relatively unbiased for all ages.

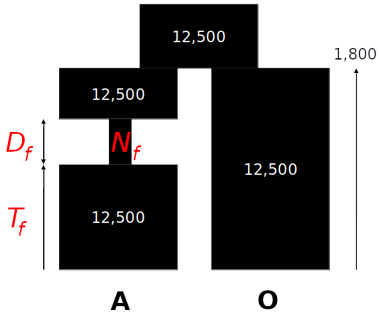

Fig F - Demographic model for multi-generation epoch model. In the figure, we show two simulated populations (A, O) that diverged 1,800 generations ago. The target population A experienced a severe bottleneck T_f generations ago, such that the population size reduced to N_f for a duration of D_f generations.

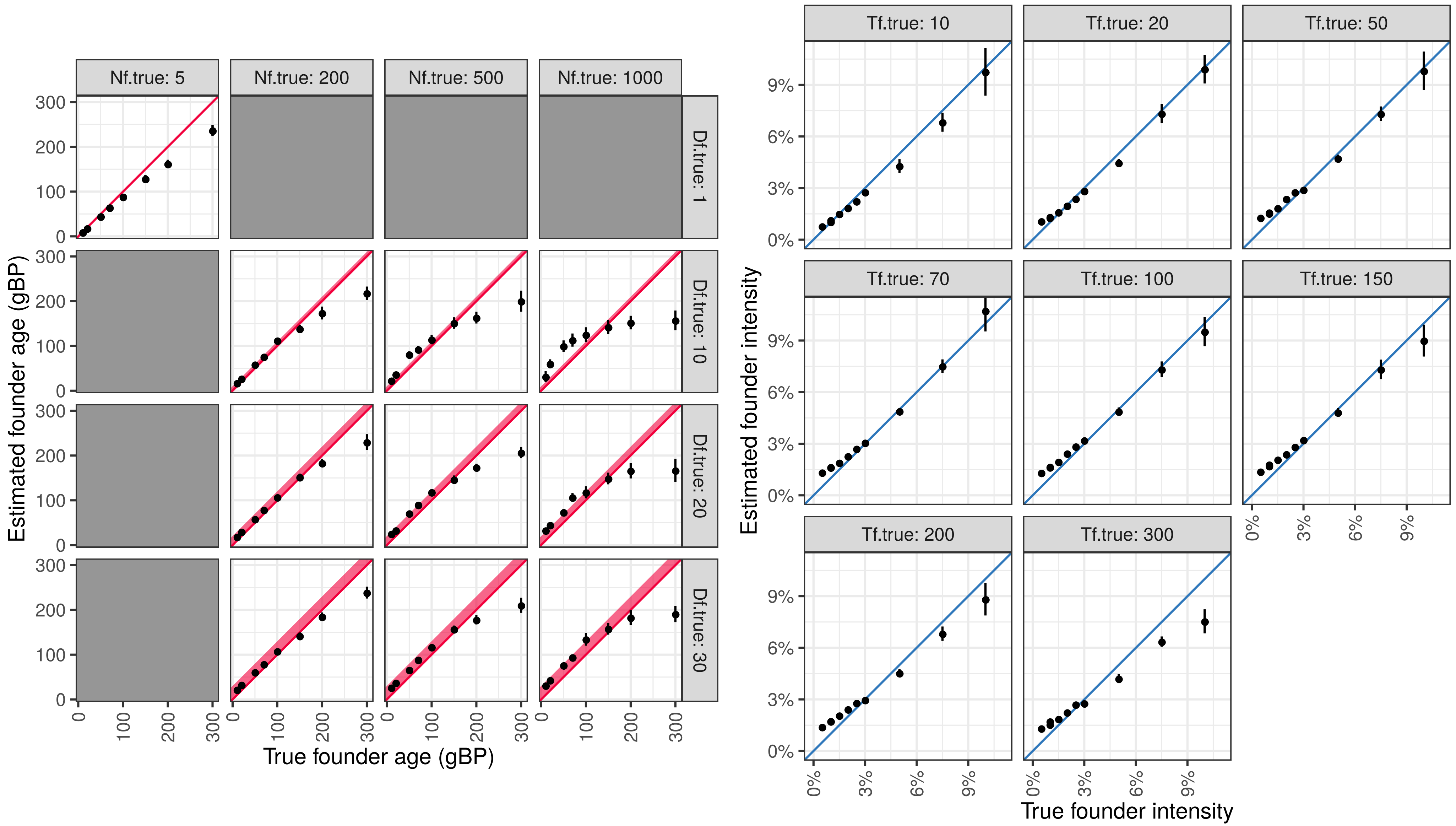

Fig G - Performance of *ASCEND* for the multi-generation epoch model: **(A)** Founder age T_f and **(B)** Founder intensity I_f.
The x-axis shows the true simulated parameter values and the y-axis shows the parameter values estimated by *ASCEND* (gBP = generations before present). The diagonal represents the expectation under the model. N_f refers to the population size during the bottleneck and D_f refers to the duration of the bottleneck. Grey boxes indicate cases where no data were simulated.

S2.3 Two-epoch bottleneck model

In real data, population history can be more complex, involving multiple bottlenecks separated by long periods of time. To study the behavior of *ASCEND* under this setup, we generated data for a target population A that experienced two founder events, one $T_f + \Delta T$ generations ago (with T_f ranging between 10 and 200 generations) where population size reduced to $N_f = 5$ during a single generation, then the population recovered to its original size N_o. This was followed by a second bottleneck T_f generations ago where the population size reduced to N_f (ranging from 5 to 500) before recovering to N_o again (Fig H). The parameter ΔT (noted as the $TimeBetweenFEs$ in the Fig I) represents the time lapsed between the two bottlenecks.

Fig I shows that we reliably recovered the intensity of the strongest founder event (here, the oldest one with $I_f = 10\%$). For severe bottlenecks ($N_f = 5$), we reliably recovered the age of the most recent founder event, but for less severe bottlenecks ($N_f \geq 200$), the estimated age is roughly the weighted average of the two founder ages, weighted by their respective intensities.

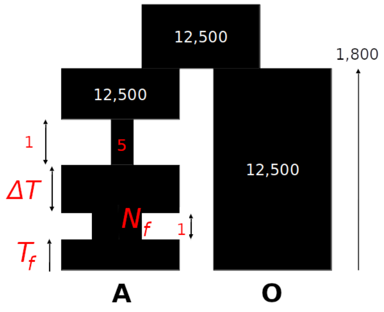

Fig H - Demographic model for the two-epoch bottleneck model. The target population A experienced two founder events, one $T_f + \Delta T$ generations ago where population size reduced to $N_f = 5$ for one generation, then the population recovered to its original size N_o. This was followed by a second bottleneck T_f generations ago where the population size reduced to N_f for a single generation, before recovering to N_o again. The parameter ΔT represents the time lapsed between the two bottlenecks.

(A)

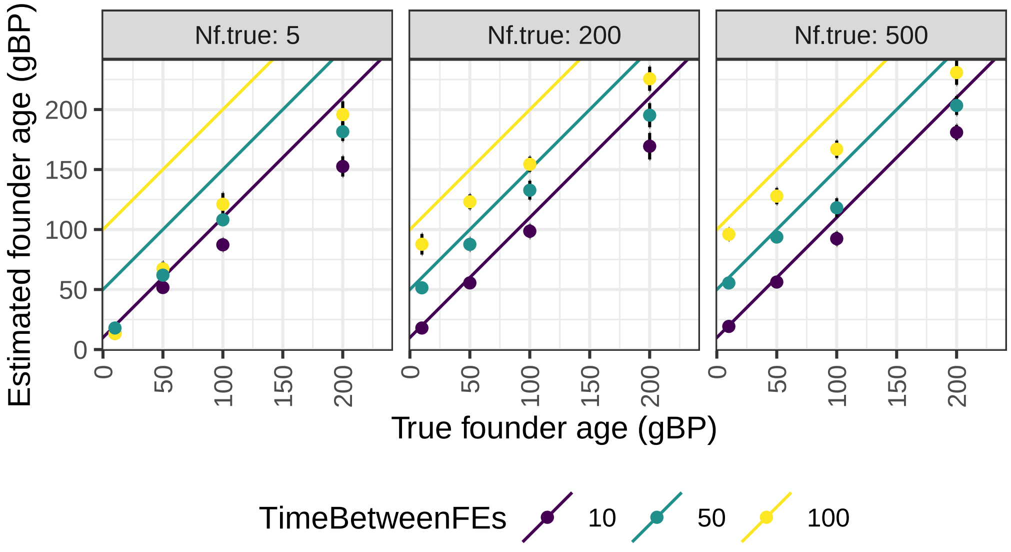

(B)

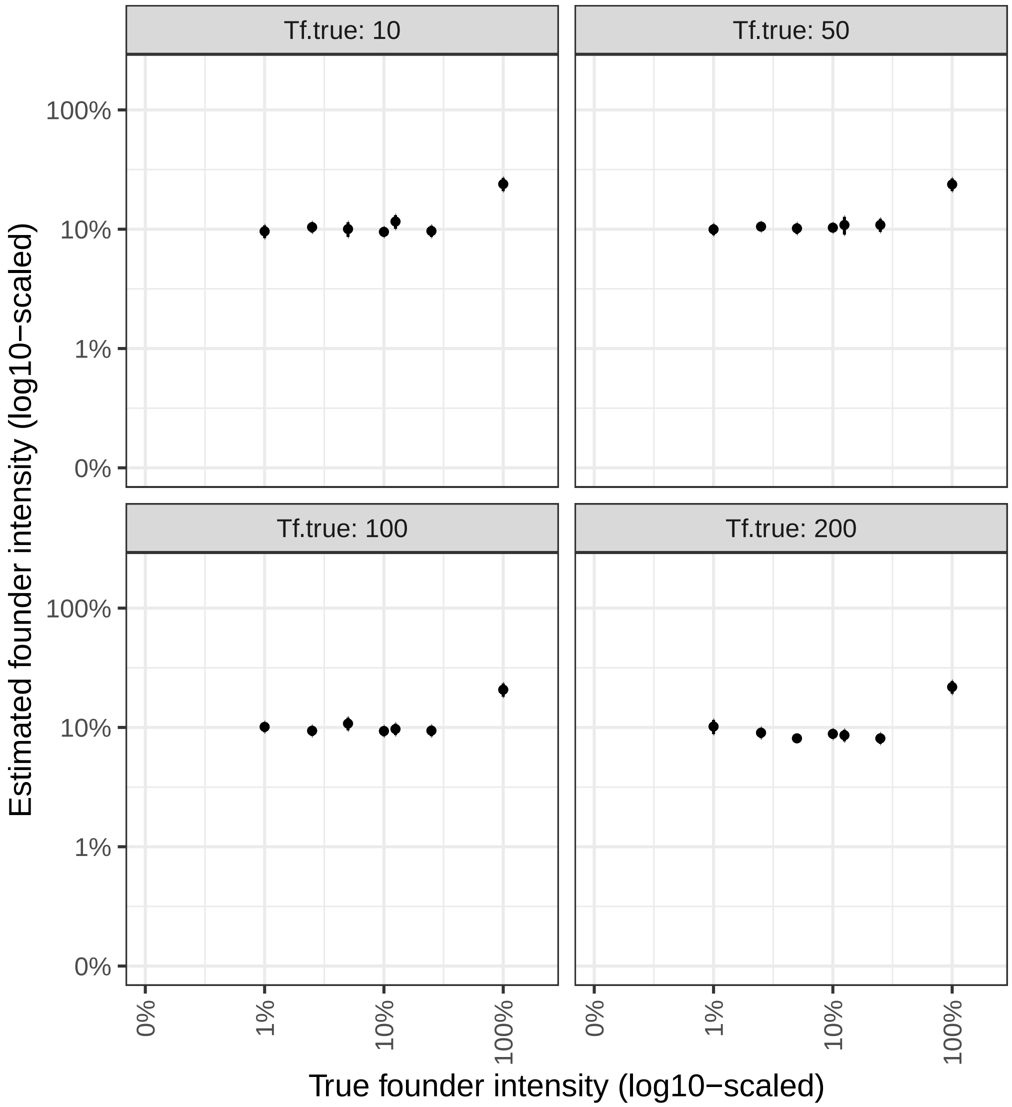

Fig I - Performance of *ASCEND* for the two-epoch bottleneck model: **(A)** Founder age T_f and **(B)** Founder intensity I_f.
The x-axis shows the true simulated parameter values and the y-axis shows the parameter values estimated by *ASCEND* (gBP = generations before present).
The red line indicates the timing of most recent founder event. N_f is the size of the last founder event.
The diagonal lines (in purple, green and yellow) represent the age of the oldest founder event (as a function of the time elapsed between the two events, *TimeBetweenFEs*).

S2.4 Model with founder event and admixture

Like a founder event, admixture introduces long-range allele correlation across the genome in the target population. Thus, under the scenario where a population has experienced both admixture and founder event, it is possible that admixture can confound the inference of the founder event.

S2.4.1 Admixture occurred *before* the population bottleneck

We simulated a target population A that derived ancestry from two ancestral populations A' and B', with ancestry proportions of 60% and 40% respectively. The two ancestral populations diverged 1,800 generations ago and the admixture occurred 110 generations ago. The target population A then experienced a severe bottleneck T_f generations ago (ranging between 10 to 100 generations) where the population size reduced to $N_f = 5$ (Fig J).

Applying *ASCEND* to the target population and using one of the ancestral groups as the outgroup (to compute cross-population allele sharing), we observed that the admixture had no impact on the inference of the parameters (age, intensity) of the founder event (Fig K).

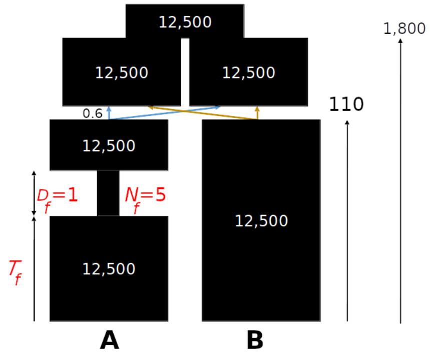

Fig J - Demographic model for admixture and founder event scenario. The target population A derived ancestry from two ancestral populations A' and B' (that are ancestral to present-day groups, A and B), with ancestry proportions of 60% and 40% respectively. The two ancestral populations diverged 1,800 generations ago and the admixture occurred 110 generations ago. The target population A then experienced a severe bottleneck T_f generations ago where the population size reduced to $N_f = 5$ for a duration D_f of single generation.

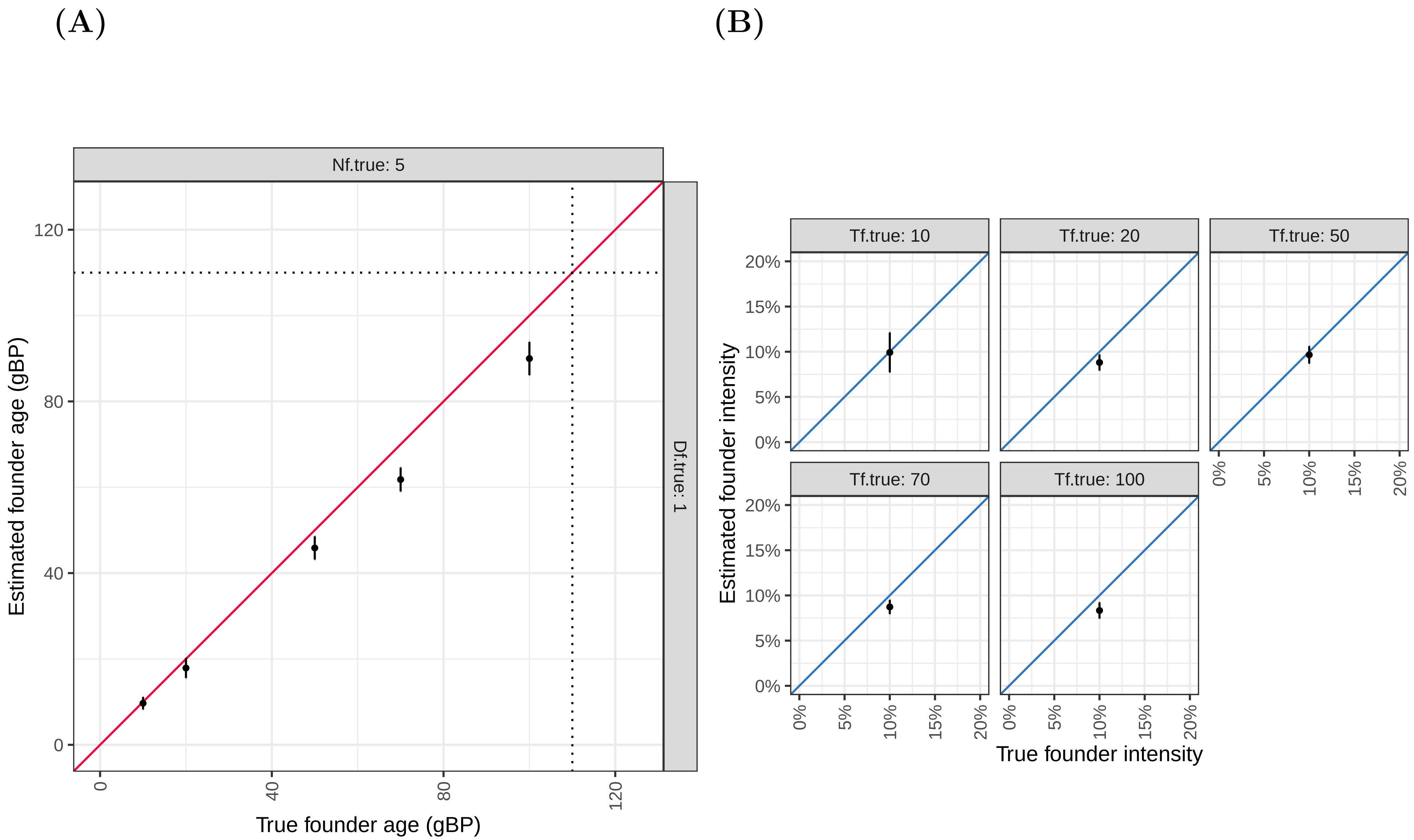

Fig K - Performance of *ASCEND* for the model with admixture and founder event: **(A)** Founder age T_f and **(B)** Founder intensity I_f.
The x-axis shows the true simulated parameter values and the y-axis shows the parameter values estimated by *ASCEND* (gBP = generations before present).
The diagonal represents the expectation under the model. N_f refers to the population size during the bottleneck and D_f refers to the duration of the bottleneck.
The dotted black line in (A) shows the time of admixture (110 generations).

S2.4.2 Admixture occurred *after* the population bottleneck

The case where admixture occurs after the population bottleneck implies that the founder event occurred in the history of the ancestral population(s). This scenario is hard to interpret, both conceptually and technically, because the admixed population has a mosaic genome with chromosomal segments from diverse ancestral populations. Using data from the target population, it is difficult to reliably infer if the founder event occurred in the target population or one or both of the ancestral groups. To explore the effect of founder events in one or both of the ancestral (source) populations of a target admixed population (Fig L), we simulated a target population A that derived ancestry from two source populations S_1 and S_2, with ancestry proportions of 40% and 60% respectively. The two ancestral populations diverged 1,800 generations ago and the admixture occurred 20 generations ago. We considered various scenarios whereby the source population S_1 experienced no bottleneck ($T_{f1} = -1$) or a severe bottleneck with intensity 10% occurring either at $T_{f1} = 30$, 50, 70, 90, 110 or 130 generations before present. Likewise, the source population S_2 could have experienced no bottleneck ($T_{f2} = -1$), or a bottleneck with intensity 10% at any of the same ages $T_{f2} = 30$, 50, 70, 90, 110 or 130 generations BP. Therefore, there are 6 scenarios where populations S_1 and S_2 experienced a founder event at the same time. In all other scenarios, founder events happened at different times between the two source populations.

Applying *ASCEND* to the target population A and using an outgroup B (that diverged 1,800 generations BP) to compute cross-population allele sharing, we observed as expected that the estimated intensity in the target was lower than simulated since admixture increases diversity in a population. The inferred timing was biased depending on the number, the source population(s) experiencing the founder event(s) and the proportion of admixture. For very low admixture scenarios, we were able to recover the timing of the founder event. However, for higher proportions of admixture, the timing was underestimated or similar to the time of admixture (Fig M). When both ancestral populations S_1 and S_2 had recent founder events, we found that the date inferred using the target A tended to reflect the date of the most recent founder event. Given the challenges in interpreting the dates, we recommend that when there is evidence of recent admixture in the target population, the users should first perform local ancestry inference in the admixed population

and apply *ASCEND* to genomic regions inferred to derive from each ancestral population separately.

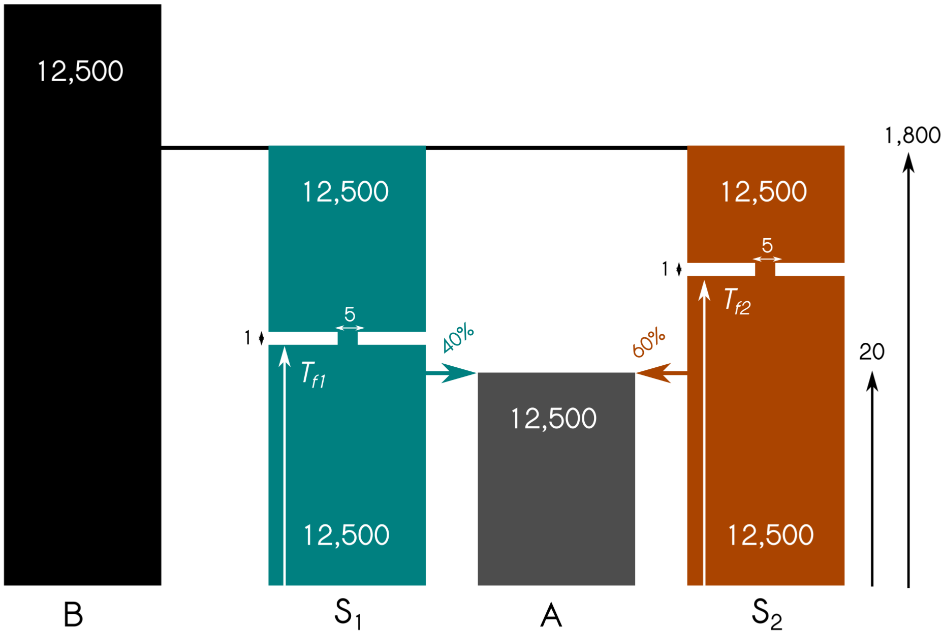

Fig L - Demographic model for founder event happening in the source populations of an admixed target population A. The target population A derived ancestry from two source populations S_1 and S_2, with ancestry proportions of 40% and 60% respectively. The two ancestral populations diverged 1,800 generations ago and the admixture occurred 20 generations ago. The source population S_1 experienced **no** bottleneck *or* a severe bottleneck T_{f1} generations ago where the population size reduced to $N_{f1} = 5$ for a duration $D_{f1} = 1$. The source population S_2 experienced **no** bottleneck *or* a severe bottleneck T_{f2} generations ago where the population size reduced to $N_{f2} = 5$ for a duration $D_{f2} = 1$.

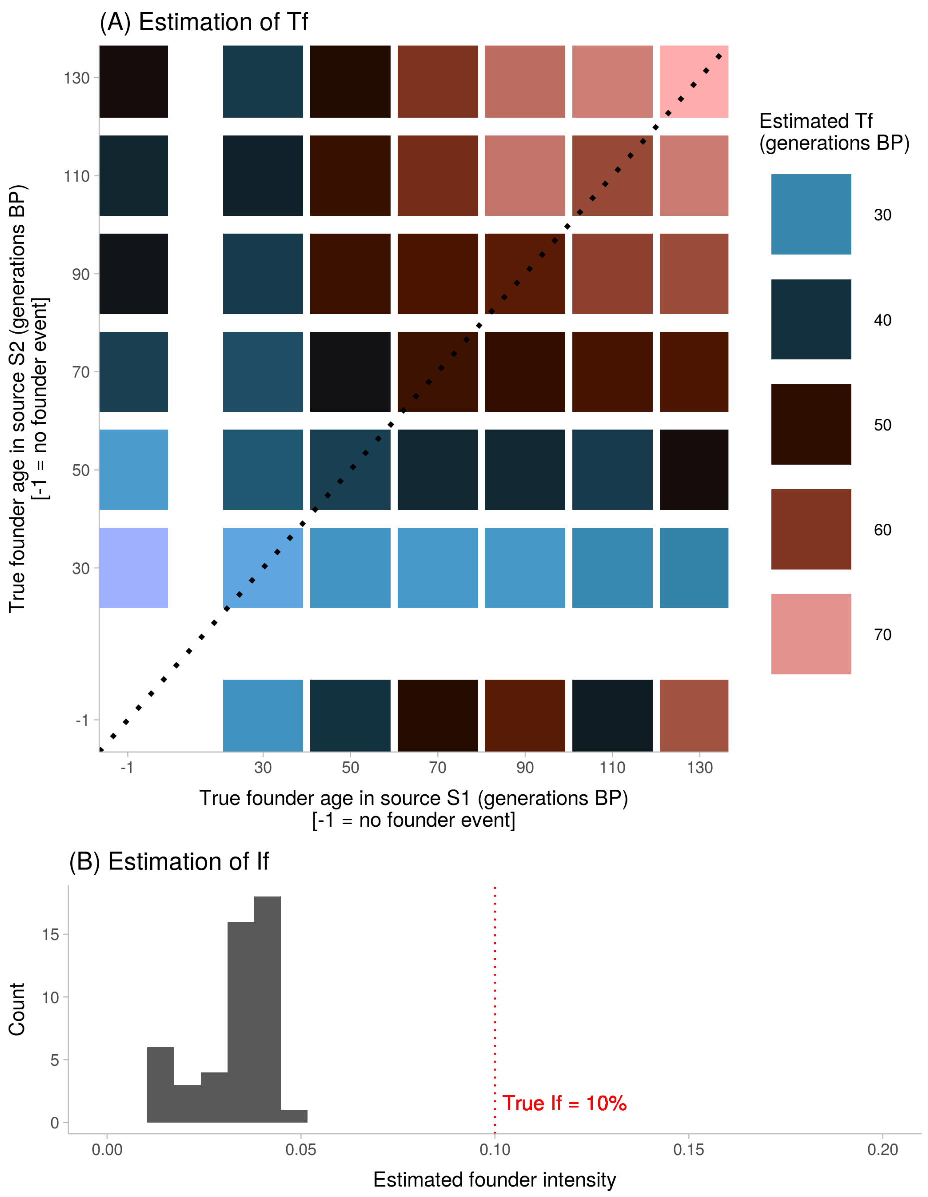

Fig M - Performance of *ASCEND* for the model with founder event in the source populations of an admixed target population: **(A)** founder age T_f and **(B)** founder intensity I_f. **(A)** The x-axis shows the true simulated age of the founder event T_{f1} in the source population S_1 (in generations BP). The y-axis shows the true simulated age of the founder T_{f2} event in the source population S_2. Note that the value of -1 means that no bottleneck was simulated. The shade of the tiles is proportional to the "founder age" estimated by *ASCEND*. (B) Histogram of the "founder intensity" estimated by *ASCEND* across all simulations performed under the model L. The red dotted line represents the true intensity of the founder events simulated in one or both of the source populations S_1 and S_2. The mode of the distribution of the estimated I_f is $\approx 4\%$.

S2.5 Gradual exponential growth model

In the gradual exponential growth model, we generated data for a target population A that experienced a founder event T_f generations ago (from 10 to 300 generations) where the population size reduced to N_f (from 5 to 1,000). This population then exponentially recovered with a rate λ to reach $N_o = 12,500$ at present (Fig N). The population size at time t after the founder event is thus equal to $N_f \cdot e^{\lambda t}$.

Applying *ASCEND* under the standard setup, which assumes the epoch model of founder event, we observed that *ASCEND* tended to underestimate the founder age and to overestimate the founder intensity. However, we note that under the gradual epoch model, our parameters of age and intensity are ill-defined as our two parameter model does not capture important summary statistics such as rate of exponential increase in population size. Our inferred parameters can be assumed as the harmonic mean of the age and intensity over the duration the bottleneck (Fig O). By leveraging additional moments (such as variance) of the two-point allele sharing statistics, we may be able to infer the rate of recovery of the bottleneck and improve the reliability of the parameter estimation.

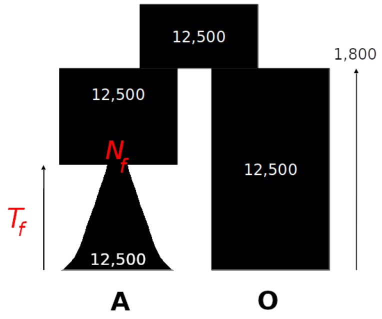

Fig N - Demographic model for gradual exponential growth model. The target population A experienced a founder event T_f generations ago where the population size reduced to N_f. This population then exponentially recovered with a rate λ to reach $N_o = 12,500$ at present.

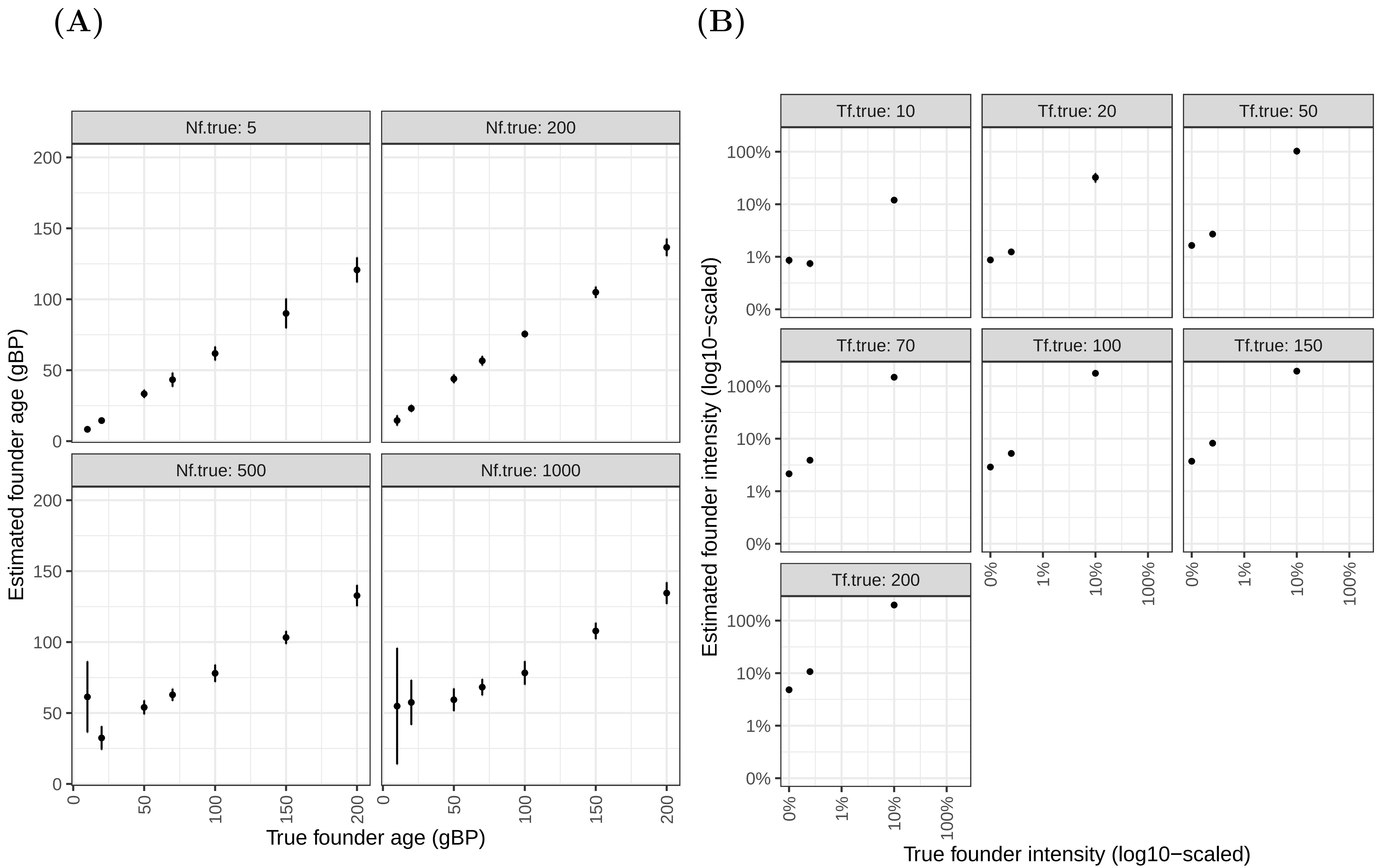

Fig O - Performance of *ASCEND* for the gradual exponential growth model: **(A)** Founder age T_f and **(B)** Founder intensity I_f.
The x-axis shows the true simulated parameter values and the y-axis shows the parameter values estimated by *ASCEND* (gBP = generations before present).
N_f refers to the population size during the bottleneck.

S2.6 No recovery founder event model

In this model, we assumed that the target population A experienced a founder event T_f generations ago (from 10 to 200 generations) where population size reduced to N_f (from 5 to 1,000). Unlike the previous simulations, the target population A did not recover after the bottleneck and maintained a low effective population size of N_f till present (Fig P).

Applying *ASCEND*, we inferred that the founder age was systematically underestimated. Here, we defined the true founder intensity I_f as $(T_f)/(2N_f)$ (instead of $(D_f)/(2N_f)$) since the bottleneck period extends over T_f generations in this model. We found that I_f was reliably estimated (Fig Q).

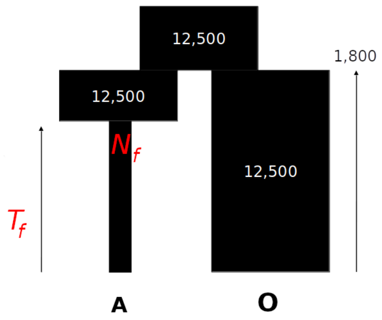

Fig P - Demographic model for no recovery after the founder event. The target population A experienced a founder event T_f generations ago where population size reduced to N_f. This historically low population size of N_f is maintained to present.

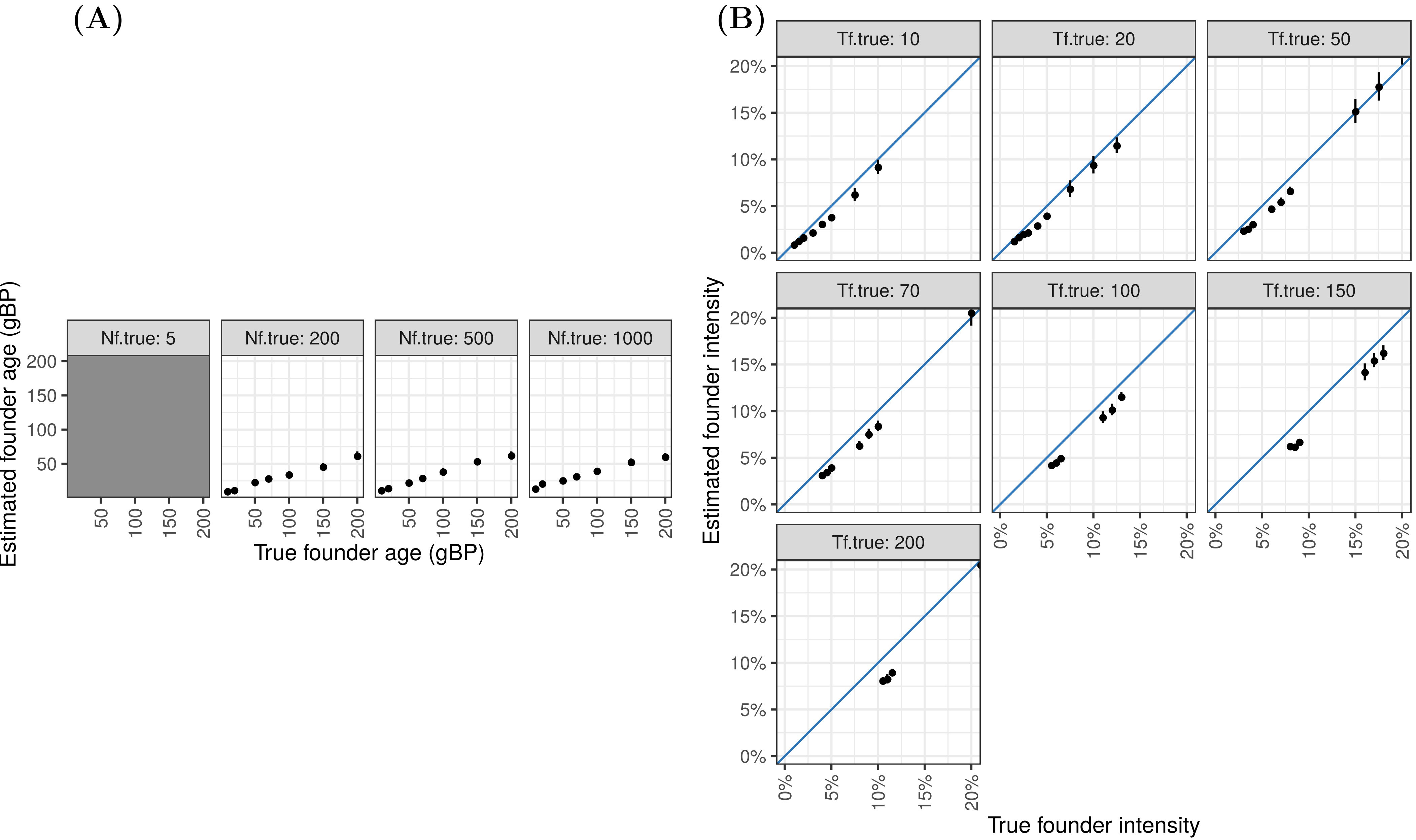

Fig Q - Performance of *ASCEND* for the no recovery founder event model: **(A)** Founder age T_f and **(B)** Founder intensity I_f. The x-axis shows the true simulated parameter values and the y-axis shows the parameter values estimated by *ASCEND* (gBP = generations before present). The diagonal represents the expectation under the model. N_f refers to the population size during the bottleneck and I_f is equal to $(T_f)/(2N_f)$.

S2.7 Robustness of the inference to data quality issues

S2.7.1 Impact of sample size

To investigate the impact of sample size of the target population on the inference of the founder event parameters, we simulated data under the single-generation epoch model (described in S2.1, Fig C). We varied the sample size (n) of the target population between $5 - 30$ diploid individuals. The sample size of the outgroup population O remained unchanged ($n = 15$ diploids) as generally there is less constraint for data from reference populations.

We observed that we could reliably estimate the age of the founder event in the target population for all sample sizes. We note however that the estimation of founder intensity works reliably for sample sizes greater than 5, what motivated the use of a minimum cutoff of 5 diploids for all analyses in real data.

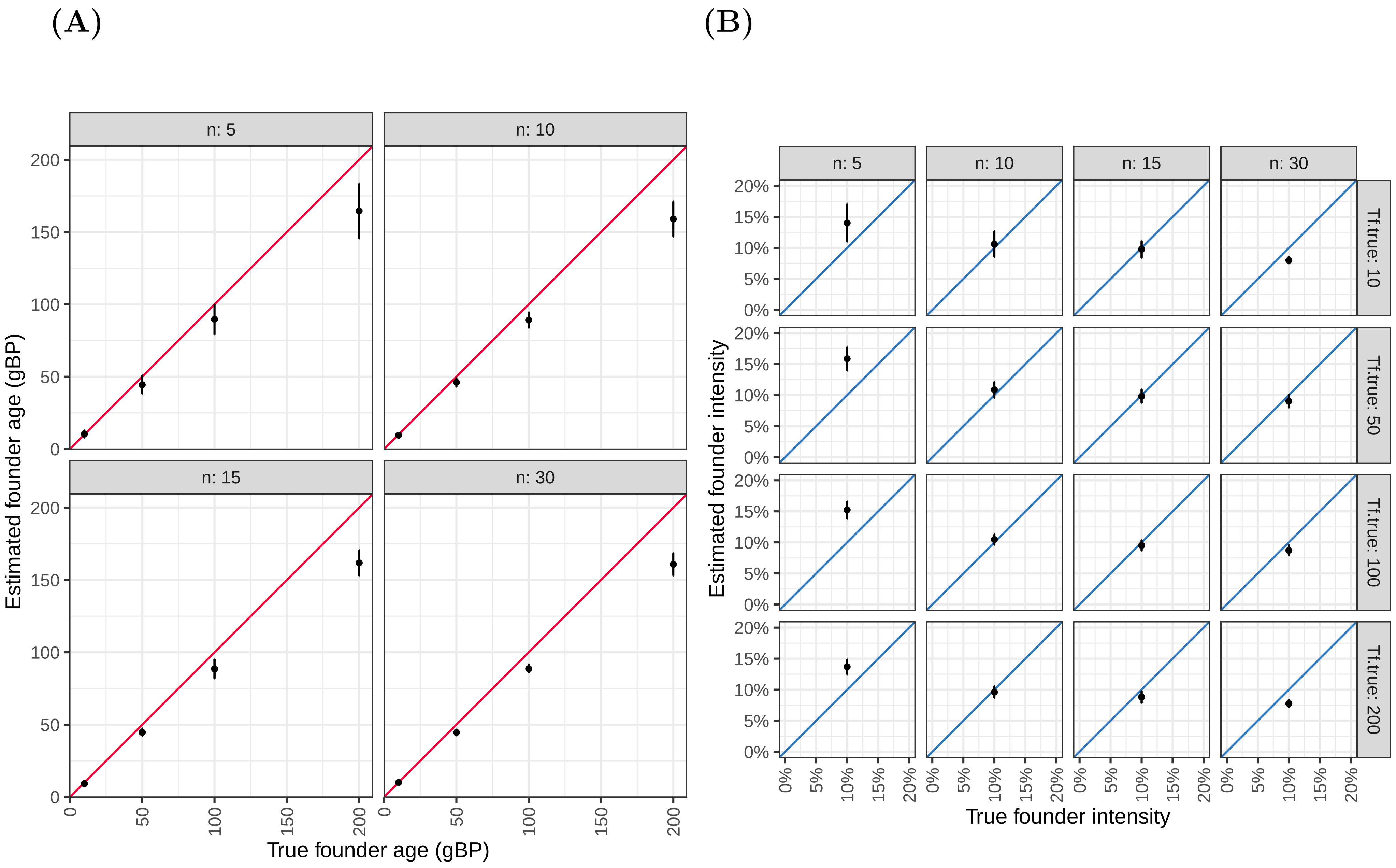

Fig R - Impact of sample size on the inference of founder parameters: **(A)** Founder age T_f and **(B)** Founder intensity I_f.
The x-axis shows the true simulated parameter values and the y-axis shows the parameter values estimated by *ASCEND* (gBP = generations before present). The diagonal represents the expectation under the model. n refers to the diploid sample size of the target population.

S2.7.2 Impact of missing data

To investigate the impact of missing genotypes in the target and outgroup population on the inference of the founder event parameters, we simulated data for the single-generation epoch model (described in S2.1, Fig C) and set some genotypes to missing with a rate ranging from 20% to 90%.

We observed that the inference was robust to large proportion of missing data ($< 90\%$) and both age and intensity were accurately inferred (Fig S). With greater that 90% missing genotypes, the inference is unstable and founder intensity can be overestimated (Fig S).

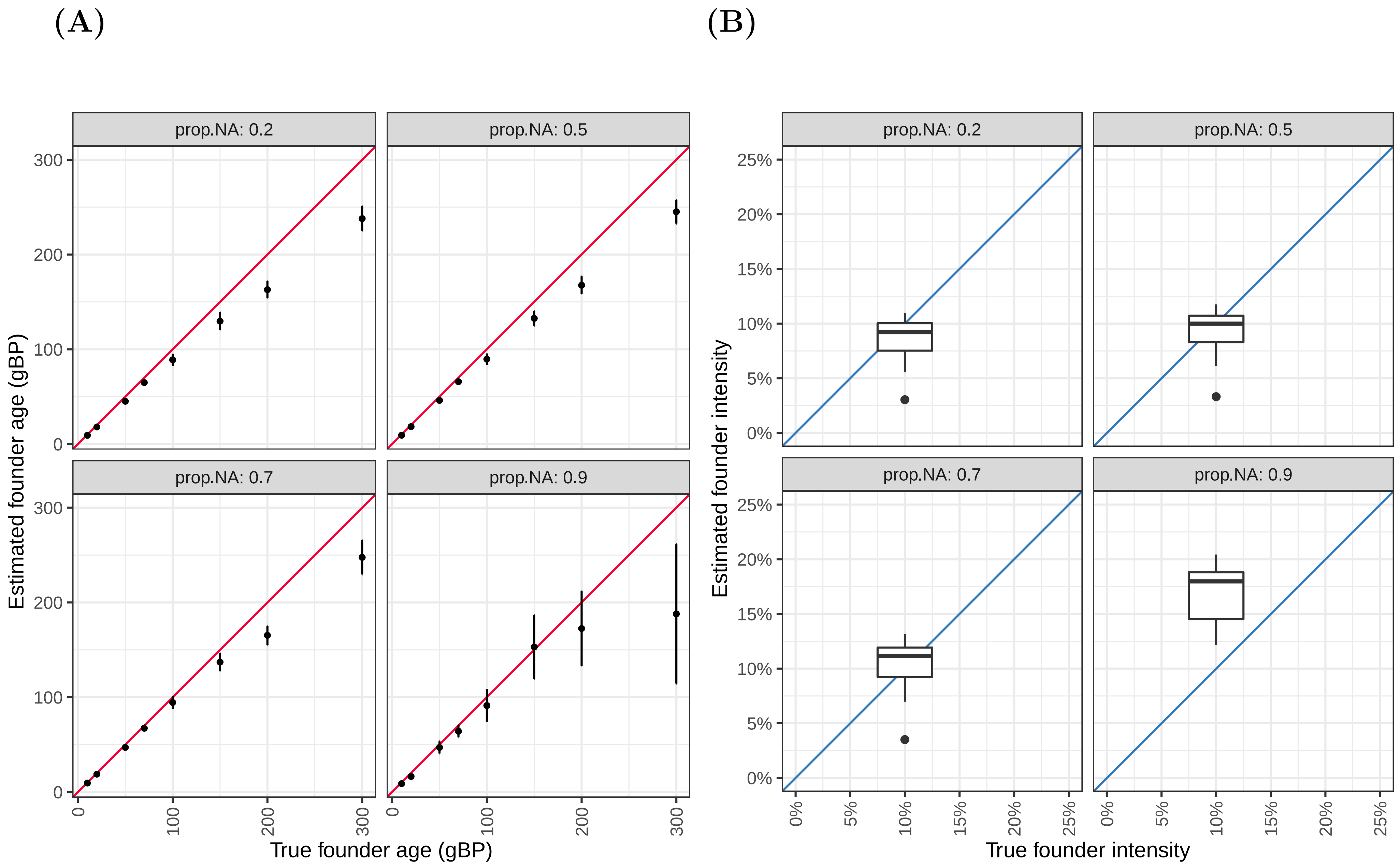

Fig S - Impact of missing data on the inference of founder parameters: **(A)** Founder age T_f and **(B)** Founder intensity I_f.
The x-axis shows the true simulated parameter values and the y-axis shows the parameter values estimated by *ASCEND* (gBP = generations before present). The diagonal represents the expectation under the model. *prop.NA* refers to the proportion of missing data.

S2.7.3 Impact of missing data: special case of ancient DNA

Ancient DNA specimens tend to have low quality of data due to poor preservation and high degradation of DNA with time. Thus, ancient DNA datasets often have high proportion of missing data and limited or low coverage. To avoid bias, it is common practice to make pseudo-haploid genotype calls using a random allele observed in the reads at each site in the genome.

To test the robustness of our inference to ancient DNA data properties, we simulated data for the single-generation epoch model (described in S2.1, Fig C) and then resampled this dataset to mimic the features of ancient genomes, namely (i) sample size was set to 5 individuals in the target population; (ii) missing genotypes: we simulated 500,000 SNPs and set 20% to 90% genotypes to missing (coded as 9 in eigenstrat files); (iii) for all sites, we used pseudo-haploid genotype calls. As described in Methods, we computed the founder parameters without subtracting the cross-population statistics.

We first applied the allele sharing correlation statistic and found that the estimated age was unbiased, though the founder intensity was underestimated using pseudo-haploid genotypes (Fig T). This is expected because pseudo-haploid data lack heterozygous sites which leads to underestimate the frequency of crossing-overs at short distances and inflate the variance in allele sharing correlation across the genome. We show that by applying a correction based on sample heterozygosity (i.e., using the weighted allele sharing correlation, cf. Methods), we could obtain nearly unbiased estimates for the founder intensity even in datasets with large proportion of missing genotypes (Fig U).

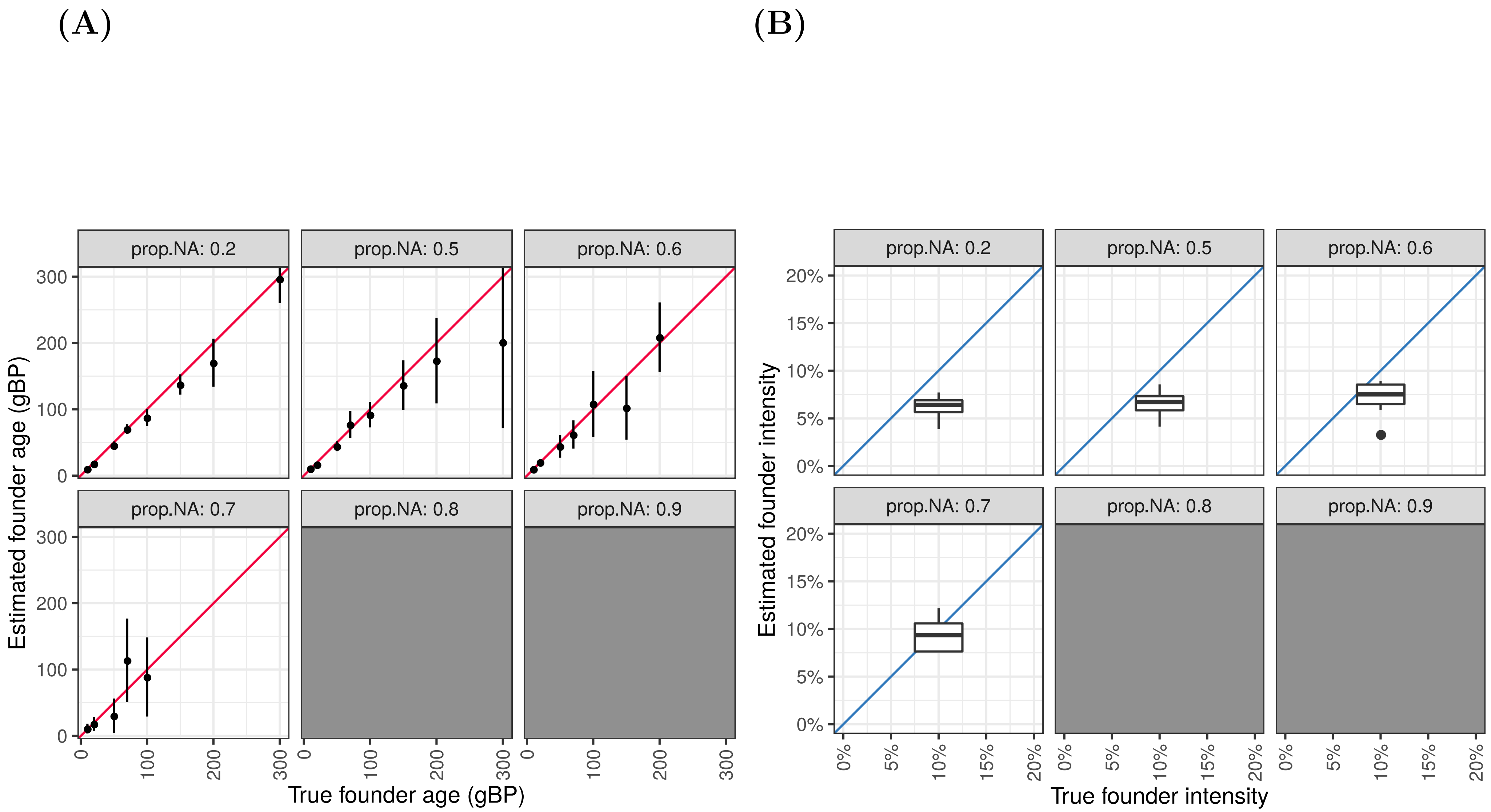

Fig T - Using the **allele sharing correlation**: **(A)** Founder age T_f and **(B)** Founder intensity I_f
The x-axis shows the true simulated parameter values and the y-axis shows the parameter values estimated by *ASCEND* (gBP = generations before present). The diagonal shows
prop.NA refers to the proportion of missing data.
Grey boxes indicate cases where founder events failed to be detected by *ASCEND* due to high proportion of missing data.

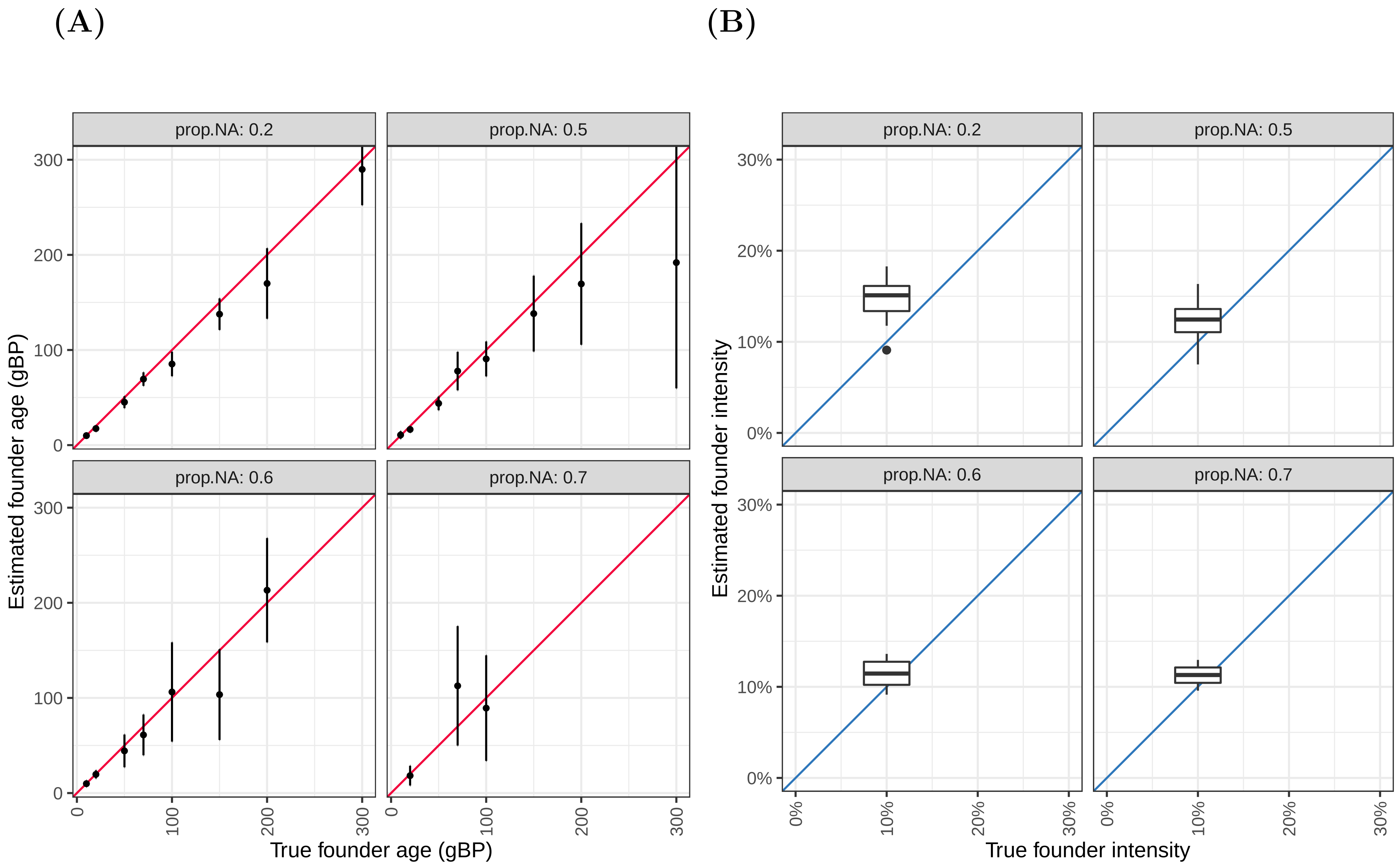

Fig U - Using the **weighted allele sharing covariance**: **(A)** Founder age T_f and **(B)** Founder intensity I_f

The x-axis shows the true simulated parameter values and the y-axis shows the parameter values estimated by *ASCEND* (gBP = generations before present). The diagonal shows
prop.NA refers to the proportion of missing data.

S2.7.4 Impact of pseudo-haploid genotypes

While pseudo-haploid genotypes are commonly used in ancient DNA applications, they can also be used for low coverage present-day samples. In our dataset, this is true for a number of populations labeled as *.SG* (S1 Table). Thus, to more rigourously study the impact of using pseudo-haploid genotypes, we pseudo-haploidized the diploid genotypes we had simulated under the multi-generation bottleneck model (Notes S2.2). The results (Fig W) show that *ASCEND* performs reliably on pseudo-haploid data for both the estimation of founder age and founder intensity when using the weighted allele sharing covariance (instead of the allele sharing correlation, which tends to underestimate the founder intensity, cf. Fig V).

Further, we compared the estimates of founder parameters for diploid and pseudo-haploid genotypes in empirical data using two populations of Onge individuals from IndiaHO and HO37 ("*Onge.SG*"). Using diploid genotypes (IndiaHO), we inferred the founder intensity was $20.63\% \pm 0.54\%$. Using the "*Onge.SG*" samples from HO37 and the allele sharing correlation statistic, we inferred the founder instensity was biased ($8.12\% \pm 0.4\%$). However, when applying the weighted allele sharing covariance statistic, we recovered estimates ($21.20\% \pm 1.10\%$) which are similar to the diploid case. These results show that the method is robust to the use of pseudo-haploid genotypes.

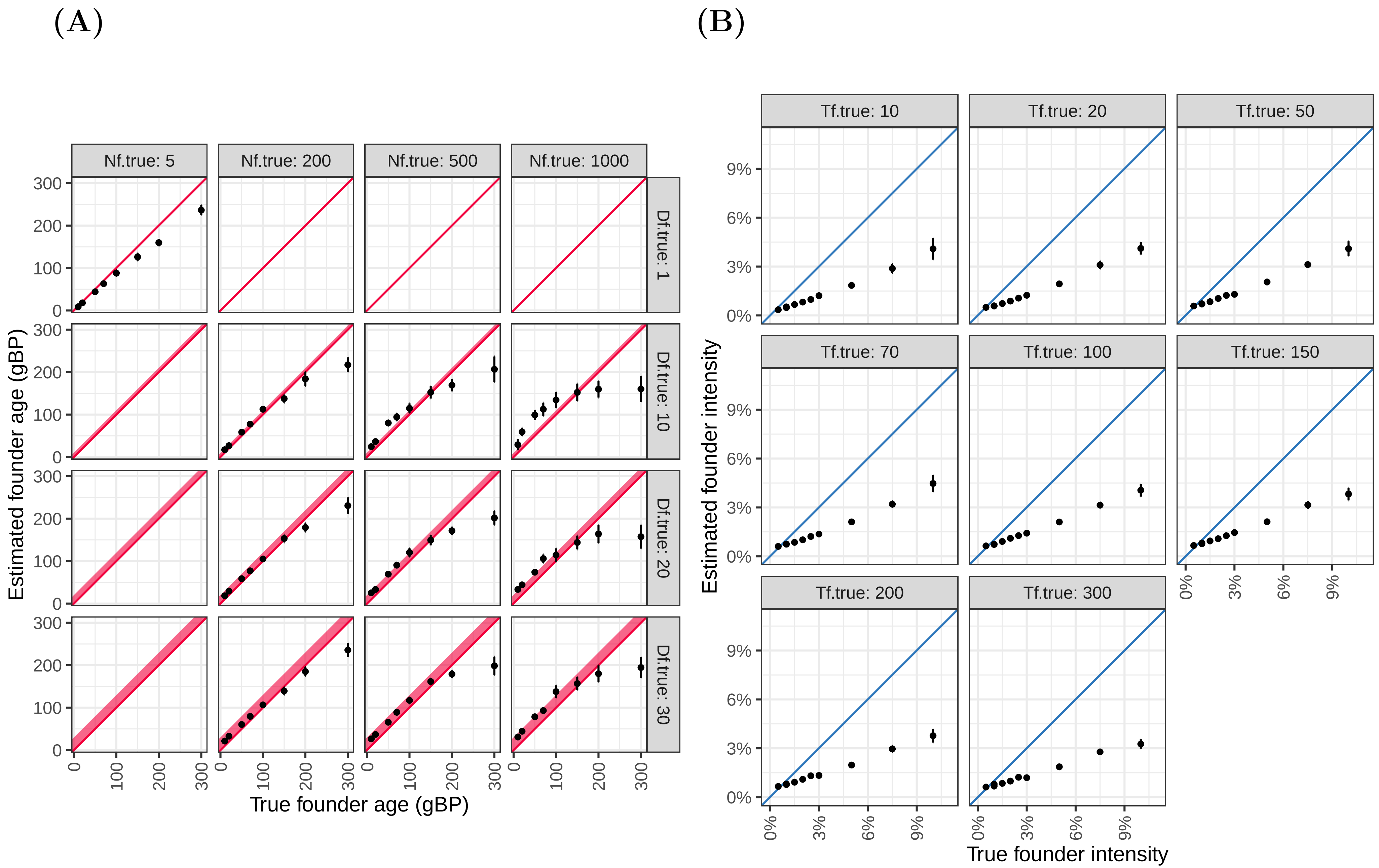

Fig V - Accuracy using pseudo-haploid data and the **correlation** function: **(A)** Founder age T_f and **(B)** Founder intensity I_f

The x-axis shows the true simulated parameter values and the y-axis shows the parameter values estimated by *ASCEND* (gBP = generations before present). The diagonal shows

prop.NA refers to the proportion of missing data.

Grey boxes indicate cases where founder events failed to be detected by *ASCEND* due to high proportion of missing data.

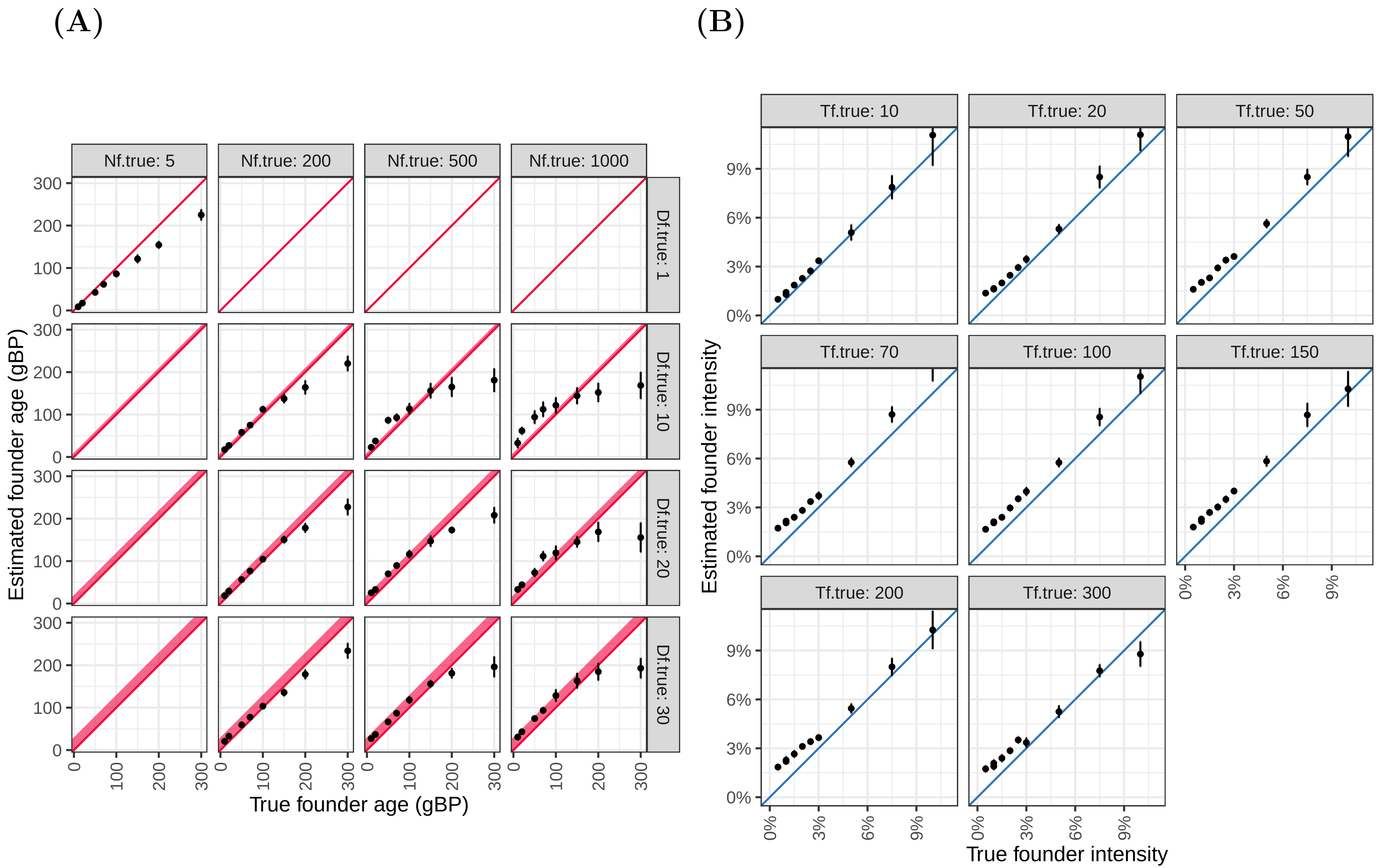

Fig W - Accuracy using pseudo-haploid data and the **weighted covariance** function: **(A)** Founder age T_f and **(B)** Founder intensity I_f
The x-axis shows the true simulated parameter values and the y-axis shows the parameter values estimated by *ASCEND* (gBP = generations before present). The diagonal shows
prop.NA refers to the proportion of missing data.
Grey boxes indicate cases where founder events failed to be detected by *ASCEND* due to high proportion of missing data.

S2.8 Null model: No recent founder event in the target population

We investigated the performance of *ASCEND* in scenarios without a recent population bottleneck in target populations. We generated data for a three-population human demographic model which includes Yoruba Africans (YRI) and two non-African groups, namely North-Western Europeans (CEU) and Han Chinese (CHB). Briefly, we assumed Yorubas have a constant population size and non-Africans share a history of out-of Africa bottleneck, followed by a recent expansion (Fig X). As for other simulation studies, we assumed a generation of time of 28 years and a mutation rate of 1.2×10^{-8} (Kong et al. 2012).

Applying *ASCEND* to each target population and using a random subset of 15 individuals from the other populations as outgroup (as done for empirical analyses to compute cross-population allele sharing), we observed no significant evidence of founder event in either population (Table C). This suggests that *ASCEND* has a low false discovery rate and provides reliable results in the absence of a founder event.

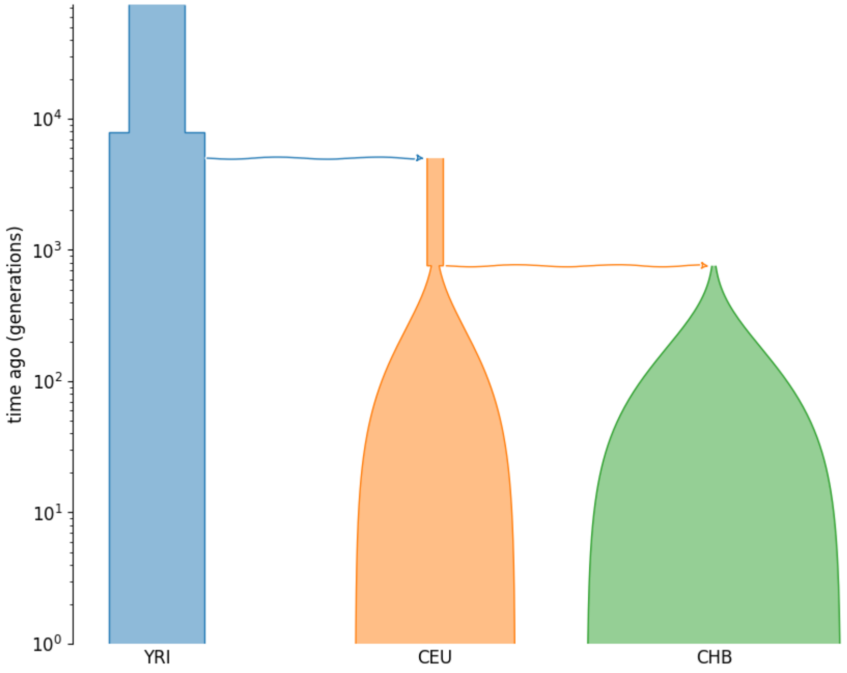

Fig X - Demographic model of constant (YRI) or expanding populations (CEU, CHB). Time is in generations and scaled in log10 units.

Table C. Inferred founder parameters in CEU, YRI and CHB based on model in Fig X.

Population	Significant	Tf	If	NRMSD	Comments
CEU	**No**	353 ± 34	$3.3\% \pm 0.3\%$	0.03	$T_f > 200$ gBP
CHB	**No**	431 ± 23	$5.4\% \pm 0.3\%$	0.02	$T_f > 200$ gBP
YRI	**No**	141 ± 15	$0.4\% \pm 0.4\%$	0.05	$I_f < 0.5\%$

Note: The column "Significant" indicates if the founder event was significant based on criteria described in Methods and "Comments" indicates the reason why the founder event estimation failed. The inferred T_f is the founder age (in generations) with associated standard errors; I_f the is founder intensity with associated standard errors.

S2.9. Comparison between Naive and FFT implementation

Using the data simulated under the S2.1 and S2.2 models, we estimated the founder parameters using both the Naïve and the FFT approach with default parameters and a mesh size of 100 (Table D). The results are nearly identical between the two approaches. However, the speed-up using FFT is around hundred-fold compared to the Naïve approach.

Table D - Comparison of founder parameters (founder age T_f, founder intensity I_f) estimated using the Naïve and the FFT approaches for data simulated under the single generation (S2.1) and multi-generation (S2.2) epoch models.

Founder age (T_f) (in generations)			N_f	D_f	Founder intensity (I_f)		
**True**	**FFT estimate** (mean ± SE)	**NAIVE estimate** (mean ± SE)	**True**	**True**	**True**	**FFT estimate** (mean ± SE)	**NAIVE estimate** (mean ± SE)
10	9 ± 1	9 ± 1	5	1	10%	9.8% ± 0.7%	9.8% ± 0.7%
10	9 ± 1	9 ± 1	5	1	10%	9.8% ± 0.7%	9.8% ± 0.7%
100	89 ± 3	89 ± 3	5	1	10%	9.5% ± 0.4%	9.5% ± 0.4%
100	89 ± 3	89 ± 3	5	1	10%	9.5% ± 0.4%	9.5% ± 0.4%
150	128 ± 4	128 ± 4	5	1	10%	9% ± 0.5%	9% ± 0.5%
150	128 ± 4	128 ± 4	5	1	10%	9% ± 0.5%	9% ± 0.5%
200	162 ± 5	163 ± 4	5	1	10%	8.8% ± 0.5%	8.8% ± 0.5%
200	162 ± 5	163 ± 4	5	1	10%	8.8% ± 0.5%	8.8% ± 0.5%
50	45 ± 1	45 ± 1	5	1	10%	9.8% ± 0.6%	9.8% ± 0.6%
50	45 ± 1	45 ± 1	5	1	10%	9.8% ± 0.6%	9.8% ± 0.6%
10	31 ± 6	32 ± 6	1000	10	0.5%	0.8% ± 0%	0.8% ± 0%
10	33 ± 3	33 ± 2	1000	20	1%	1.1% ± 0%	1.2% ± 0%
10	31 ± 2	31 ± 2	1000	30	1.5%	1.5% ± 0%	1.5% ± 0%
10	17 ± 1	17 ± 1	200	10	2.5%	2.2% ± 0.1%	2.3% ± 0.1%
10	19 ± 1	18 ± 1	200	20	5%	4.3% ± 0.2%	4.3% ± 0.2%
10	22 ± 1	22 ± 1	200	30	7.5%	6.8% ± 0.3%	6.9% ± 0.3%
10	23 ± 2	23 ± 2	500	10	1%	1% ± 0%	1.1% ± 0%
10	25 ± 1	25 ± 1	500	20	2%	1.9% ± 0%	1.9% ± 0%
10	27 ± 1	26 ± 1	500	30	3%	2.8% ± 0.1%	2.8% ± 0.1%
100	125 ± 8	125 ± 8	1000	10	0.5%	1.3% ± 0.1%	1.3% ± 0.1%
100	118 ± 7	115 ± 6	1000	20	1%	1.7% ± 0%	1.7% ± 0%
100	134 ± 7	132 ± 7	1000	30	1.5%	2% ± 0.1%	2% ± 0.1%
100	112 ± 3	112 ± 2	200	10	2.5%	2.8% ± 0.1%	2.9% ± 0.1%
100	107 ± 2	107 ± 2	200	20	5%	4.9% ± 0.1%	4.9% ± 0.1%
100	108 ± 2	108 ± 2	200	30	7.5%	7.3% ± 0.2%	7.4% ± 0.2%
100	114 ± 5	116 ± 5	500	10	1%	1.6% ± 0%	1.7% ± 0%
100	118 ± 3	119 ± 4	500	20	2%	2.4% ± 0.1%	2.5% ± 0.1%
100	117 ± 3	116 ± 3	500	30	3%	3.2% ± 0.1%	3.2% ± 0.1%
150	142 ± 8	142 ± 7	1000	10	0.5%	1.4% ± 0%	1.4% ± 0%
150	149 ± 7	147 ± 6	1000	20	1%	1.7% ± 0.1%	1.7% ± 0.1%
150	158 ± 7	156 ± 7	1000	30	1.5%	2.1% ± 0%	2.1% ± 0%
150	138 ± 5	137 ± 5	200	10	2.5%	2.8% ± 0.1%	2.9% ± 0.1%
150	152 ± 4	150 ± 4	200	20	5%	4.8% ± 0.1%	4.9% ± 0.1%

150	142 ± 4	139 ± 4	200	30	7.5%	7.3% ± 0.3%	7.3% ± 0.3%
150	151 ± 6	154 ± 7	500	10	1%	1.8% ± 0.1%	1.8% ± 0.1%
150	146 ± 5	147 ± 6	500	20	2%	2.4% ± 0.1%	2.4% ± 0.1%
150	157 ± 4	155 ± 4	500	30	3%	3.2% ± 0.1%	3.3% ± 0.1%
200	152 ± 8	147 ± 8	1000	10	0.5%	1.4% ± 0%	1.4% ± 0%
200	166 ± 9	167 ± 8	1000	20	1%	1.7% ± 0%	1.8% ± 0%
200	183 ± 9	184 ± 9	1000	30	1.5%	2.1% ± 0.1%	2.1% ± 0.1%
200	174 ± 7	171 ± 6	200	10	2.5%	2.8% ± 0.1%	2.8% ± 0.1%
200	183 ± 4	180 ± 4	200	20	5%	4.5% ± 0.1%	4.5% ± 0.1%
200	185 ± 5	184 ± 5	200	30	7.5%	6.8% ± 0.2%	6.8% ± 0.2%
200	163 ± 7	165 ± 7	500	10	1%	1.7% ± 0.1%	1.8% ± 0.1%
200	174 ± 4	170 ± 4	500	20	2%	2.4% ± 0%	2.4% ± 0%
200	178 ± 5	177 ± 6	500	30	3%	3% ± 0%	3% ± 0.1%
50	100 ± 7	97 ± 6	1000	10	0.5%	1.3% ± 0%	1.3% ± 0%
50	73 ± 4	73 ± 4	1000	20	1%	1.6% ± 0%	1.6% ± 0%
50	76 ± 3	77 ± 2	1000	30	1.5%	1.8% ± 0%	1.9% ± 0%
50	59 ± 2	59 ± 2	200	10	2.5%	2.8% ± 0.1%	2.8% ± 0.1%
50	58 ± 1	58 ± 2	200	20	5%	4.7% ± 0.1%	4.8% ± 0.1%
50	61 ± 2	62 ± 2	200	30	7.5%	7.3% ± 0.2%	7.4% ± 0.2%
50	81 ± 4	80 ± 3	500	10	1%	1.5% ± 0%	1.5% ± 0%
50	71 ± 2	71 ± 2	500	20	2%	2.4% ± 0.1%	2.4% ± 0.1%
50	66 ± 3	66 ± 2	500	30	3%	2.9% ± 0.1%	2.9% ± 0.1%

Note: The column labeled "**True**" indicates the true values of the founder event parameters that were used in the simulations; N_f = population size during the founder event; D_f = duration of founder event. For FFT, we used a mesh size of 100.

S2.10. *msprime* commands

Below we include the msprime command lines used for simulating demographic models reported in *S2.1-S2.8*. If not stated otherwise, the *msprime* commands reported below are given using the *msprime* 0.7 syntax.

S2.1 Single-generation epoch model

T_f: (variable) parameter provided by the user. Note that we simulated four populations, but only the first one and the last one (the target and outgroup populations respectively) were used for the inference.

```
L = 50e6
recomb_rate = 1e-8
mut_rate = 1.2e-8
No = 12500
Nf = 5
Df = 1

pop_conf = [
    msprime.PopulationConfiguration(sample_size=30, initial_size = No),
    msprime.PopulationConfiguration(sample_size=30, initial_size = No),
    msprime.PopulationConfiguration(sample_size=30, initial_size = No),
    msprime.PopulationConfiguration(sample_size=30, initial_size = No)]

migr_matrix = [[0, 0, 0, 0],
               [0, 0, 0, 0],
               [0, 0, 0, 0],
```

```
               [0, 0, 0, 0]]

demo = [
       msprime.PopulationParametersChange(time = Tf, initial_size = Nf, population_id = 0),
       msprime.PopulationParametersChange(time = Tf+Df, initial_size = No, population_id = 0),
       msprime.MassMigration(time = 200, source = 1, destination = 0, proportion = 1),
       msprime.MassMigration(time = 1000, source = 2, destination = 0, proportion = 1),
       msprime.MassMigration(time = 1800, source = 3, destination = 0, proportion = 1)]

tree = msprime.simulate(population_configurations = pop_conf,
                            migration_matrix = migr_matrix,
                            length = L,
                            recombination_rate = recomb_rate,
                            mutation_rate = mut_rate,
                            demographic_events = demo)
```

S2.2 Multi-generation epoch model

Tf, **Nf**, **Df**: parameters set by the user (variables). Note that we simulated four populations, but only the first one and the last one (the target and outgroup populations respectively) were used for the inference.

```
L = 50e6
recomb_rate = 1e-8
mut_rate = 1.2e-8
No = 12500

pop_conf = [
    msprime.PopulationConfiguration(sample_size=30, initial_size = No),
    msprime.PopulationConfiguration(sample_size=30, initial_size = No),
    msprime.PopulationConfiguration(sample_size=30, initial_size = No),
    msprime.PopulationConfiguration(sample_size=30, initial_size = No)]

migr_matrix = [[0, 0, 0, 0],
               [0, 0, 0, 0],
               [0, 0, 0, 0],
               [0, 0, 0, 0]]

demo = [
       msprime.PopulationParametersChange(time = Tf, initial_size = Nf, population_id = 0),
       msprime.PopulationParametersChange(time = Tf+Df, initial_size = No, population_id = 0),
       msprime.MassMigration(time = 200, source = 1, destination = 0, proportion = 1),
       msprime.MassMigration(time = 1000, source = 2, destination = 0, proportion = 1),
       msprime.MassMigration(time = 1800, source = 3, destination = 0, proportion = 1)]

tree = msprime.simulate(population_configurations = pop_conf,
                            migration_matrix = migr_matrix,
                            length = L,
                            recombination_rate = recomb_rate,
                            mutation_rate = mut_rate,
                            demographic_events = demo)
```

S2.3 Two-epoch bottleneck model

Tf, **Nf**, **Df**: parameters set by the user (variable).

```
L = 50e6
recomb_rate = 1e-8
mut_rate = 1.2e-8
No = 12500

pop_conf = [
    msprime.PopulationConfiguration(sample_size=30, initial_size = No),
    msprime.PopulationConfiguration(sample_size=30, initial_size = No)]

migr_matrix = [[0, 0], [0, 0]]

demo = [
    msprime.PopulationParametersChange(time = Tf, initial_size = Nf, population_id = 0),
    msprime.PopulationParametersChange(time = Tf+1, initial_size = No, population_id = 0),
    msprime.PopulationParametersChange(time = Tf+Df, initial_size = 5, population_id = 0),
    msprime.PopulationParametersChange(time = Tf+Df+1, initial_size = No, population_id = 0),
```

```
    msprime.MassMigration(time = 1800, source = 1, destination = 0, proportion = 1)]

tree = msprime.simulate(population_configurations = pop_conf,
                            migration_matrix = migr_matrix,
                            length = L,
                            recombination_rate = recomb_rate,
                            mutation_rate = mut_rate,
                            demographic_events = demo)
```

S2.4 Model with founder event and admixture

S2.4.1 Admixture occurred before the population bottleneck

Tf, **Ta**: parameters set by the user (variable). Note that we simulated four populations, but only the first one and the last one (the target and outgroup populations respectively) were used for the analysis.

```
L = 50e6
recomb_rate = 1e-8
mut_rate = 1.2e-8
No = 12500
prop_admix = 0.4
Nf = 5
Df = 1

pop_conf = [
    msprime.PopulationConfiguration(sample_size=30, initial_size = No),
    msprime.PopulationConfiguration(sample_size=30, initial_size = No),
    msprime.PopulationConfiguration(sample_size=0, initial_size = No),
    msprime.PopulationConfiguration(sample_size=30, initial_size = No)]

migr_matrix = [[0, 0, 0, 0],
               [0, 0, 0, 0],
               [0, 0, 0, 0],
               [0, 0, 0, 0]]

demo = [
     msprime.PopulationParametersChange(time = Tf, initial_size = Nf, population_id = 0),
     msprime.PopulationParametersChange(time = Tf+Df, initial_size = No, population_id = 0),

     msprime.MassMigration(time = Ta, source = 0, destination = 2, proportion = prop_admix),
     msprime.MassMigration(time = Ta, source = 1, destination = 2, proportion = prop_admix),

     msprime.MassMigration(time = 1800, source = 0, destination = 3, proportion = 1),
     msprime.MassMigration(time = 1800, source = 1, destination = 3, proportion = 1),
     msprime.MassMigration(time = 1800, source = 2, destination = 3, proportion = 1)]

tree = msprime.simulate(population_configurations = pop_conf,
                            migration_matrix = migr_matrix,
                            length = L,
                            recombination_rate = recomb_rate,
                            mutation_rate = mut_rate,
                            demographic_events = demo)
```

S2.4.2 Admixture occurred after the population bottleneck

Tf1, Tf2: parameters set by the user (variable): founder ages in the source population named pop2 and pop3 in the commands below (named S1 and S2 respectively in Notes S2.4.2). The admixed target population is named pop0 in the commands below (named A in Notes S2.4.2). The commands allow generating a Demography object under the *msprime* 1.0 syntax.

```
Args = dict()
Args[“No”] = 12500
Args[“T_split”] = 1800
Args[“T_admix”] = 20
Args[“p_admix”] = 0.4
Args[“Nf1”], Args[“Nf1”] = 5, 5
Args[“Df1”], Args[“Df2”] = 1, 1
```

```
Demo = msp.Demography()
for i in range(5):
    Demo.add_population(name = "pop_"+str(i),
                        default_sampling_time = 0.,
                        growth_rate = 0.,
                        initially_active = True,
                        initial_size = Args["No"])
Demo.add_population_split(time = Args["T_split"], derived = ["pop_2"], ancestral = "pop_4")
Demo.add_population_split(time = Args["T_split"], derived = ["pop_3"], ancestral = "pop_4")
assert Args["p_admix"] >= 0 and Args["p_admix"] <= 1, "p_admix should be between 0 and 1"
if Args["Tf1"]!=-1: assert Args["T_split"] > (Args["Tf1"]+Args["Df1"]), "T_split > Tf+Df"
if Args["Tf1"]!=-1: assert Args["T_admix"] < Args["Tf1"], "T_admix < Tf"
if Args["Tf2"]!=-1: assert Args["T_split"] > (Args["Tf2"]+Args["Df2"]), "T_split > Tf+Df"
if Args["Tf2"]!=-1: assert Args["T_admix"] < Args["Tf2"], "T_admix < Tf"
Demo.add_mass_migration(time = Args["T_admix"]-1, source = "pop_0", dest = "pop_2", proportion
= Args["p_admix"])
Demo.add_mass_migration(time = Args["T_admix"]-1, source = "pop_1", dest = "pop_2", proportion
= Args["p_admix"])
Demo.add_population_split(time = Args["T_admix"], derived = ["pop_0", "pop_1"], ancestral =
"pop_3")
if Args["Tf1"] != -1:
    Demo.add_population_parameters_change(time = Args["Tf1"], initial_size = Args["Nf1"],
growth_rate = 0., population = "pop_2")
    Demo.add_population_parameters_change(time = Args["Tf1"]+Args["Df1"], initial_size =
Args["No"], growth_rate = 0., population = "pop_2")
if Args["Tf2"] != -1:
    Demo.add_population_parameters_change(time = Args["Tf2"], initial_size = Args["Nf2"],
growth_rate = 0., population = "pop_3")
    Demo.add_population_parameters_change(time = Args["Tf2"]+Args["Df2"], initial_size =
Args["No"], growth_rate = 0., population = "pop_3")
Demo.sort_events()
```

S2.5 Gradual exponential growth model

Tf, **Nf**: parameters set by the user (variable).

```
L = 50e6
recomb_rate = 1e-8
mut_rate = 1.2e-8
No = 12500
Df = 1
alpha = 1/(Tf*1.) * np.log(No/(Nf*1.))

pop_conf = [
    msprime.PopulationConfiguration(sample_size=30, initial_size = No, growth_rate = alpha),
    msprime.PopulationConfiguration(sample_size=30, initial_size = No)]

migr_matrix = [[0, 0],
               [0, 0]]

demo = [
    msprime.PopulationParametersChange(time = Tf, initial_size = No, population_id = 0,
growth_rate = 0),
    msprime.MassMigration(time = 1800, source = 1, destination = 0, proportion = 1)]


tree = msprime.simulate(population_configurations = pop_conf,
                            migration_matrix = migr_matrix,
                            length = L,
                            recombination_rate = recomb_rate,
                            mutation_rate = mut_rate,
                            demographic_events = demo)
```

S2.6 No recovery founder event model

Tf, **Nf**, **Df**: parameters set by the user (variable).

Note that we simulated four populations, but only the first one and the last one (the target and outgroup populations respectively) were used for the analysis.

```
L = 50e6
recomb_rate = 1e-8
mut_rate = 1.2e-8
No = 12500
Df = 1

pop_conf = [
    msprime.PopulationConfiguration(sample_size=30, initial_size = Nf),
    msprime.PopulationConfiguration(sample_size=30, initial_size = No),
    msprime.PopulationConfiguration(sample_size=30, initial_size = No),
    msprime.PopulationConfiguration(sample_size=30, initial_size = No)]

migr_matrix = [[0, 0, 0, 0],
               [0, 0, 0, 0],
               [0, 0, 0, 0],
               [0, 0, 0, 0]]

demo = [
       msprime.PopulationParametersChange(time = Tf, initial_size = Nf, population_id = 0),
       msprime.PopulationParametersChange(time = Tf+Df, initial_size = No, population_id = 0),
       msprime.MassMigration(time = 200, source = 1, destination = 0, proportion = 1),
       msprime.MassMigration(time = 1000, source = 2, destination = 0, proportion = 1),
       msprime.MassMigration(time = 1800, source = 3, destination = 0, proportion = 1)]

tree = msprime.simulate(population_configurations = pop_conf,
                            migration_matrix = migr_matrix,
                            length = L,
                            recombination_rate = recomb_rate,
                            mutation_rate = mut_rate,
                            demographic_events = demo)
```

S2.7.1 Impact of sample size

Tf, **sample_size**: parameters set by the user (variable).

Note that we simulated four populations, but only the first one and the last one (the target and outgroup populations respectively) were used for the analysis.

```
L = 50e6
recomb_rate = 1e-8
mut_rate = 1.2e-8
No = 12500
Nf = 5
Df = 1

pop_conf = [
    msprime.PopulationConfiguration(sample_size=sample_size*2, initial_size = No),
    msprime.PopulationConfiguration(sample_size=0, initial_size = No),
    msprime.PopulationConfiguration(sample_size=0, initial_size = No),
    msprime.PopulationConfiguration(sample_size=30, initial_size = No)]

migr_matrix = [[0, 0, 0, 0],
               [0, 0, 0, 0],
               [0, 0, 0, 0],
               [0, 0, 0, 0]]

demo = [
       msprime.PopulationParametersChange(time = Tf, initial_size = Nf, population_id = 0),
       msprime.PopulationParametersChange(time = Tf+Df, initial_size = No, population_id = 0),
       msprime.MassMigration(time = 200, source = 1, destination = 0, proportion = 1),
       msprime.MassMigration(time = 1000, source = 2, destination = 0, proportion = 1),
       msprime.MassMigration(time = 1800, source = 3, destination = 0, proportion = 1)]

tree = msprime.simulate(population_configurations = pop_conf,
                            migration_matrix = migr_matrix,
                            length = L,
                            recombination_rate = recomb_rate,
                            mutation_rate = mut_rate,
                            demographic_events = demo)
```

S2.7.2 Impact of missing data

We used the same data as generated from S2.1, but randomly converted a proportion of genotypes (based on user input) to missing.

S2.7.3 Impact of ancient DNA data features

Tf: parameter set by the user (variable).
Note that we simulated four populations, but only the first one and the last one (the target and outgroup populations respectively) were used for the analysis.

```
L = 50e6
recomb_rate = 1e-8
mut_rate = 1.2e-8
No = 12500
Df = 1
Nf = 5

pop_conf = [
    msprime.PopulationConfiguration(sample_size=10, initial_size = No),
    msprime.PopulationConfiguration(sample_size=0, initial_size = No),
    msprime.PopulationConfiguration(sample_size=0, initial_size = No),
    msprime.PopulationConfiguration(sample_size=30, initial_size = No)]

migr_matrix = [[0, 0, 0, 0],
               [0, 0, 0, 0],
               [0, 0, 0, 0],
               [0, 0, 0, 0]]

demo = [
       msprime.PopulationParametersChange(time = Tf, initial_size = Nf, population_id = 0),
       msprime.PopulationParametersChange(time = Tf+Df, initial_size = No, population_id = 0),
       msprime.MassMigration(time = 200, source = 1, destination = 0, proportion = 1),
       msprime.MassMigration(time = 1000, source = 2, destination = 0, proportion = 1),
       msprime.MassMigration(time = 1800, source = 3, destination = 0, proportion = 1)]

tree = msprime.simulate(population_configurations = pop_conf,
                            migration_matrix = migr_matrix,
                            length = L,
                            recombination_rate = recomb_rate,
                            mutation_rate = mut_rate,
                            demographic_events = demo)
```

To generate pseudo-haploid genotypes, we further processed the *EIGENSTRAT .geno* file by choosing a random allele at each heterozygous site and replaced the existing heterozygous genotype by two copies of the random allele. Further, depending on the user-defined proportion of missing sites, we marked sites as missing (i.e., with a value of 9 in the EIGENSTRAT format).

S2.8 Null model: No recent founder event in the target population

Here, we simulate a model of 3 human populations (YRI, CEU and CHB) under a model where YRI has a constant size and CEU and CHB experience population expansion after an out-of-Africa bottleneck. We did not include migration between the three populations. This model is derived from the OutOfAfrica_3G09 scenario from stdpopsim [1]. The commands hereunder allow generating a Demography object under the *msprime* 1.0 syntax.

```
L = 50e6
recomb_rate = 1e-8
mut_rate = 1.2e-8
generation_time = 28

Ne_factor = np.true_divide(2.35e-8, mut_rate)
N_A = 7300 * Ne_factor
N_B = 2100 * Ne_factor
N_AF = 12300 * Ne_factor
N_EU0 = 1000 * Ne_factor
```

```
N_AS0 = 510 * Ne_factor
T_AF = 220e3 / generation_time
T_B = 140e3 / generation_time
T_EU_AS = 21.2e3 / generation_time
r_EU = 0.004
r_AS = 0.0055
N_EU = N_EU0 / math.exp(-r_EU * T_EU_AS)
N_AS = N_AS0 / math.exp(-r_AS * T_EU_AS)
Demo = msp.Demography()
Demo.add_population(name = "YRI",
                        default_sampling_time = 0.,
                        growth_rate = 0.,
                        initially_active = True,
                        initial_size = N_AF)
Demo.add_population(name = "CEU",
                        default_sampling_time = 0.,
                        growth_rate = r_EU,
                        initially_active = True,
                        initial_size = N_EU)
Demo.add_population(name = "CHB",
                        default_sampling_time = 0.,
                        growth_rate = r_AS,
                        initially_active = True,
                        initial_size = N_AS)
Demo.add_population_parameters_change(time = T_EU_AS, initial_size = N_B, growth_rate = 0.,
population = "CEU")
Demo.add_population_parameters_change(time = T_AF, initial_size = N_A, growth_rate = 0.,
population = "YRI")
Demo.add_mass_migration(time = T_EU_AS, source = "CHB", dest = "CEU", proportion = 1.0)
Demo.add_mass_migration(time = T_B, source = "CEU", dest = "YRI", proportion = 1.0)
Demo.sort_events()
```

S3. Data curation for human datasets

We applied *ASCEND* to three human datasets that we describe below:

Human Origins Dataset (HO37): This dataset comprises 5,637 present-day individuals from 530 groups genotyped for 597,573 autosomal SNP positions using the Affymetrix Human Origins array (v37.2 release of the Allen Ancient DNA Resource, AADR [2]).

IndiaHO: This dataset comprises 1,662 individuals from 249 ethno-linguistic groups from India genotyped on the Human Origins array at 499,158 SNPs and was released in [7].

Human Origins Dataset (HO44): This dataset comprises 5,225 ancient individuals genotyped for 1,233,013 autosomal SNP positions using the Affymetrix Human Origins array (v44.3 release of AADR [2]).

During curation, we removed 3,754 individuals (1,976 groups) based on the following criteria (S1 Table) (note that some individuals can overlap multiple criteria):

(1) To reliably calculate allele sharing correlation, we removed groups with less than 5 individuals (2,994 individuals, 1,862 groups),
(2) Groups with post-filtering sample sizes of less than 5 individuals (608 individuals, 95 groups),
(3) Groups that were labeled as “Ignore” in the original dataset or marked as duplicates, relatives or low-quality samples (126 individuals, 16 groups),
(4) Groups with samples generated by whole genome amplification, indicated with suffix “.WGA” (52 individuals, 15 groups).

For the groups that were retained post-filtering, we removed 881 additional individuals (216 groups) which had either more than 80% of missing genotypes or were identified as close relatives using the following criteria:

(i) a pairwise genomic sharing (π) greater than 0.45 with another individual in the dataset. We note π is an estimator of the proportion of genome shared identical-by-descent (IBD) and values of 0.45 are expected for first-degree relatives, or
(ii) both $\pi > 0.125$ (as expected for third-degree relatives) and at least one segment of IBD that is greater than 65 cM (almost half the length of an average chromosome) with another individual in the dataset. The latter condition was only applied for diploid samples as phasing and hence IBD detection is challenging for pseudo-haploid and ancient DNA samples.

For the calculation of pairwise π, we used PLINK v1.90b6.2 *genome* module with the following command line:

```
plink --file XX --genome --out XX
```

For the detection of IBD segments, we first phased the samples from each dataset using EAGLE 2.4.1 with default parameters [6] with the 1000 Genomes Project Phase 3 phased samples as a reference panel to increase the phasing accuracy [3]:

```
eagle \
      --vcfTarget=XX.QQ.vcf.gz \
      --vcfRef=1000Genomes.QQ.vcf.gz \
      --outPrefix=XX.QQ.phased \
      --geneticMapFile=genetic_map_hg19.txt.gz \
      --chrom=QQ \
      --numThreads=4 \
      --allowRefAltSwap \
      2>&1 | tee XX.QQ.phasing.log
```

The IBD segments were called using GERMLINE 1.5.3 [5] with default parameters and the -*genotype* extension mode.

```
germline -input XX.QQ.phased.012.ped XX.QQ.phased.012.cM.map \
      -output XX.QQ.phased.012 \
      -bits 75 \
      -err_hom 0 \
      -err_het 0 \
      -min_m 3 \
      -g_extend
```

We applied the *HaploScore* algorithm [4] to remove false positive IBD segments with the recommended genotype error of 0.75%, switch error of 0.3% and the threshold matrix for a mean overlap of 80% as suggested by Durand et al. (2014) [4].

```
python2.7 haploscore.py \
      XX.QQ.phased.012.match \
      XX.QQ.phased.012.ped
      XX.QQ.phased.012.cM.map \
      XX.QQ.phased.012.haploscore \
      --genotype_error 0.0075 \
      --switch_error 0.003
      --filter 0.8 \
      --threshold_file chr21.scorethresh.txt
```

After filtering, our dataset contained 2,310 present-day from 184 groups for the HO37 dataset, 1,253 individuals from 116 populations in the IndiaHO dataset (we also excluded the 12 patients affected by Progressive Pseudorheumatoid Dysplasia (PPD) to limit the analysis to unaffected samples) and 1,947 ancient individuals from 164 groups for the HO44 dataset (S1 Table).

S4. Comparison of overlapping groups across human datasets

In our inference of founder event distribution among present-day humans, we used two datasets: HO37 and IndiaHO (Notes S3). A total of 19 populations were present in both datasets (Table E). For each of these shared populations, we compared the founder event parameters estimated using each dataset independently. For the subset of populations where we detected the founder events in both datasets, we found that for all except one population (Irula) the results were statistically consistent (with overlapping 95% confidence intervals) (Table E).

Table E - Comparison of the estimated founder age and intensity obtained for present-day human populations that are present in two datasets (HO37 and IndiaHO).

Population	Sample sizes+		Founder age (in generations)		Founder intensity	
	IndiaHO	HO37	IndiaHO	HO37	IndiaHO	HO37
**Balochi**	18	20	34 ± 5	34 ± 4	0.88% ± 0.08%	0.86% ± 0.07%
**Bengali**	7	7	--	--	--	--
**Bahmin_UP**	5	10	--	--	--	--
**Brahui**	19	21	22 ± 1	20 ± 1	1.16% ± 0.04%	1.17% ± 0.05%
**Burusho**	20	23	12 ± 1	13 ± 1	0.82% ± 0.03%	0.81% ± 0.04%
**GujaratiB**	5	5	--	--	--	--
**GujaratiC**	5	5	--	--	--	--
**GujaratiD**	5	5	75 ± 24	123 ± 30	2.19% ± 0.42%	3.62% ± 0.52%
**Hazara**	11	16	15 ± 3	--	0.64% ± 0.08%	--
**Irula**	15	10	34 ± 2	19 ± 2	3.05% ± 0.11%	5.50% ± 0.30%
**Jew_Ashkenazi**	7	7	43 ± 8	37 ± 7	2.01% ± 0.23%	1.7% ± 0.19%
**Jew_Cochin**	13	5	--	10 ± 1	--	2.39% ± 0.18%
**Kalash**	15	16	17 ± 1	16 ± 1	5.62% ± 0.13%	5.62% ± 0.14%
**Kusunda**	7	7	6 ± 1	7 ± 1	2.62% ± 0.16%	3.43% ± 0.24%
**Makrani**	18	19	20 ± 2	15 ± 2	0.84% ± 0.05%	0.94% ± 0.05%
**Onge§**	7	6	20 ± 1	22 ± 2	20.63% ±0.54%	21.20% ± 1.10%
**Pathan**	18	17	--	--	--	--
**Punjabi**	8	9	41 ± 15	68 ± 16	0.89% ± 0.18%	1.28% ± 0.18%
**Sindhi_Pakistan**	17	14	--	--	--	--

Notes.
(in red) Results shown in red significantly differ across the two datasets compared.
\+ Indicates the post-filtering sample size.
-- Indicates that we were unable to obtain a reliable fit due to one of the criteria: the 95% confidence interval of either T_f or I_f included 0; NRMSD>0.29; T_f>200 generations; I_f<0.5% or the standard error of T_f>50 generations.
§ For Onge, the samples in HO37 include pseudo-haploid genotypes and hence we use the weighted ancestry allele sharing covariance (see Methods) to infer the founder event parameters.

To investigate the cause of discrepancy in the estimates for Irula, we merged the IndiaHO and HO37 datasets with the 1000 Genomes Project Phase 3 dataset [3] and performed a principal component analysis (PCA) using *smartpca* [8]. We computed the eigenvectors using three populations from the 1000 Genomes Project: Han Chinese in Beijing, China (CHB) as a proxy for East-Asian ancestry, Northern and Western Europeans (CEU) as a proxy to West Eurasian ancestry and Indian Telugu (ITU) as a proxy for South Asian ancestry and projected the Irula individuals onto these eigenvectors. We found that the Irula individuals from the two datasets were fairly heterogeneous in relationship to the West Eurasians and South Asians (Fig Y). This could be due to substructure or sampling bias in the two studies or recent gene flow in some individuals, which would partially explain the differences in founder parameters in Table E.

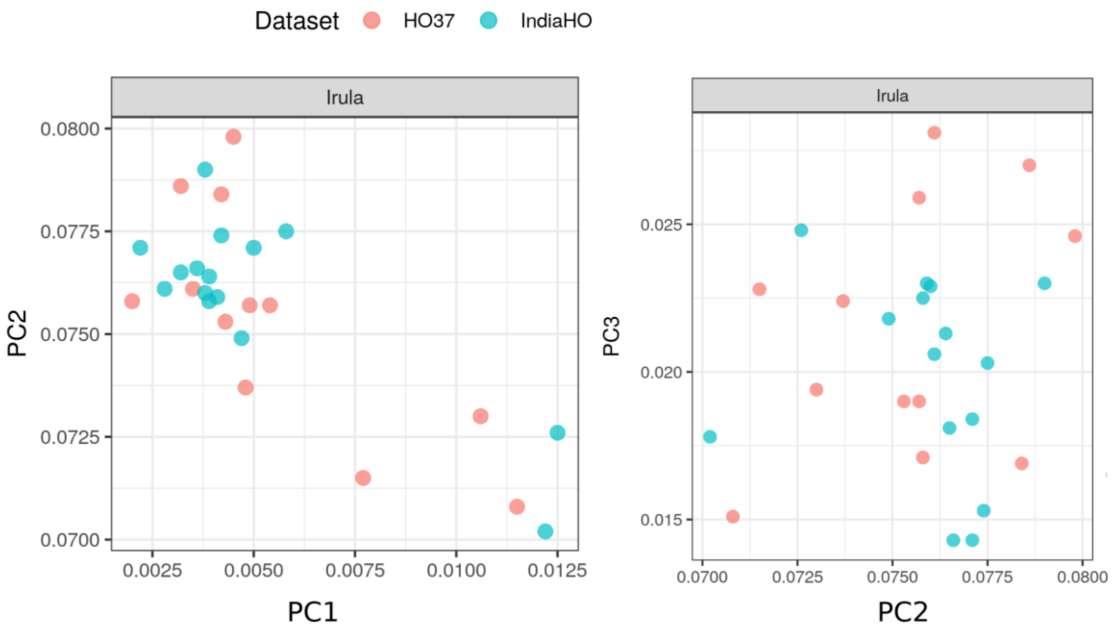

Fig Y – Principal Component Analysis (PCA) of Irula individuals from IndiaHO and HO37 datasets. We performed PCA using three populations from the 1000 Genomes Project: Han Chinese in Beijing, China (CHB) as a proxy for East-Asian ancestry, Northern and Western Europeans (CEU) as a proxy to West Eurasian ancestry and Indian Telugu (ITU) as a proxy for South Asian ancestry and projected the Irula samples onto these eigenvectors, using the individuals from IndiaHO (blue) and HO37 (red) datasets.

S5. Comparison of our results with published estimates

S5.1 Comparison of founder ages with Reich *et al.* (2009)

ASCEND is an extension of the allele sharing correlation statistic introduced in [11] that was applied to the founder ages in 25 Indian groups. To compare the performance of *ASCEND* with the previous study, we applied *ASCEND* to the original dataset from [11] except for one group (Siddis) for which some samples were missing.

To reliably compare the results, we matched the setup in Reich et al. and separately analyzed the populations on the Indian Cline (the cline formed by the relatedness of Indian groups to west Eurasian populations) and populations that fall off the Indian Cline including Austro-Asiatic speakers, Tibeto-Burmese speakers and Andamanese groups. To correct for allele sharing inherited from the ancestral population, Reich et al. subtracted the allele sharing correlation obtained by comparisons across different outgroups, picking the closest match among the groups. For populations on the Indian Cline, we matched the ancestry proportion across target and outgroup populations as described in the original study. However, for populations not on the Indian Cline, details were less clearly described but we followed the same setup (as described below). Specifically, we ran the analysis as follows:

(**i**) For each population on the Indian Cline, we computed the within-population allele sharing correlation across individuals in the target population and then subtracted the cross-population correlation computed between the individuals in the target and individuals on the Indian Cline that best matched the proportion of ANI ancestry estimated in the target population, using the following categories similar to [11]:

- 65% ± 5% ANI ancestry: Meghawal, Vaish and Kashmiri Pandit;
- 58% ± 5% ANI ancestry: Velama, Srivastava, Meghawal and Vaish;
- 53% ± 5% ANI ancestry: Lodi, Naidu, Tharu, Velama and Srivastava;
- 47% ± 5% ANI ancestry: Bhil, Satnami, Kurumba, Kamsali, Vysya, Lodi, Naidu and Tharu;
- 42% ± 5% ANI ancestry: Mala, Madiga, Chenchu, Bhil, Satnami, Kurumba, Kamsali and Vysya.

If a target population was present in two categories, then we used the reference populations present in both the categories of outgroups.

(**ii**) Austroasiatic and Tibeto-Burmese speakers (Aonaga, Hallaki, Kharia, Nysha, Sahariya, Santhal), we computed the within-population allele sharing correlation across individuals in the target population and then subtracted the cross-population correlation computed between the individuals in the target and 15 random individuals sampled from the other off-cline populations (excluding the target population).

(**iii**) Andamanese groups (Great Andamanese, Onge). For each focal population, we used the other population as the outgroup to compute the cross-population correlation.

(**iv**) We removed monomorphic SNPs and computed allele sharing correlations between genetic distances from (a) 0.1 to 10 cM with a bin size of 0.1 cM (as done in Reich et al.) and (b) from 0.1 to 30 cM with a bin size of 0.1 cM as it appeared from the decay plots that the allele sharing correlation had not decayed to 0 by 10 cM.

Out of 24 Indian groups, we obtained significant evidence of founder events in 11 populations using *ASCEND*, compared to the 12 groups reported in Reich et al. For the overlapping 11 groups, we found the founder ages were consistent for all populations, except one population (Table F). Our dates of founder events were much more recent in Sahariya (39 [32–38]), compared to Reich et al. that estimated it at 108 generations ago. We note the exponential fit for Sahariya in Reich et al. appears to be very noisy (based on visual inspection) suggesting the inconsistency in the dates is likely driven by poor fit in the original study.

For many populations, visual inspection of the decay curves suggested that the allele sharing correlation had not decayed all the way to 0 in the Reich et al. study (the intercept was > 0 at 10 cM) indicating that there were remnant signals across the genome which were not captured by the fit. To account for long range allele sharing correlation in the samples, we ran *ASCEND* from 0.1–30 cM with a bin size of 0.1 cM (Fig AA). For most groups, we observed the larger distance was useful to capture the full signal of the founder event. The results between the two settings were consistent for all groups except Onge, though the estimated ages were systematically lower for 0.1–10 cM as longer haplotypes present in the samples were not captured (Fig Z). Thus, for other analyses of South Asian individuals (as for all human analyses presented in this study), we used the 0.1–30 cM setting to capture most of the signal of the founder events.

Table F - Comparison of founder age estimates (in generations) based on Reich et al. (2009) [11] and *ASCEND*.

Population	Cline*	Reich et al. (2009)+		This study (using *ASCEND-FFT*)		
		Sample size	Founder age+	Sample size	Founder age Mean [95% CI] Range: **0.1-10 cM**	Founder age Mean [95% CI] Range: **0.1-30 cM**
Aonaga	NIC	4	120	4	100 [68-132]	93 [73-112]
Bhil	IC	7	40	7	--	19 [4–35]
Chenchu	IC	6	10	6	10 [8-13]	9 [7–10]
Great_Andamanese	NIC	7	14	7	10 [5-16]	5 [3-6]
Hallaki	NIC	7	32	7	22 [9-36]	12 [4-19]
Kamsali	IC	4	--	4	--	--
Kashmiri_Pandit	IC	5	--	5	--	--
Kharia	NIC	6	42	6	44 [15-72]	14 [5-22]
Kurumba	IC	9	--	9	--	--
Lodi	IC	5	--	5	--	--
Madiga	IC	4	--	4	--	--
Mala	IC	3	--	3	--	--
Meghawal	IC	5	59	5	46 [20-71]	53 [28–77]
Naidu	IC	4	--	4	--	--
Nysha	NIC	4	134	4	124 [89-160]	106 [64-148]
Onge	NIC	9	39	9	35 [32-38]	26 [24-28]
Sahariya	NIC	4	108	4	39 [32-38]	39 [20-57]
Santhal	NIC	7	--	7	126 [28-225]	118 [12-224]
Satnami	IC	4	--	4	--	--
Siddi	IC	4	8	2	NA	NA
Srivastava	IC	2	--	2	--	--
Tharu	IC	9	--	9	--	--
Vaish	IC	4	--	4	--	--
Velama	IC	4	88	4	82 [51-112]	59 [17–101]
Vysya	IC	5	108	5	92 [42-142]	72 [23–121]

Note:

(in red) Results shown in red significantly differ across the two studies.

-- indicates that we were unable to obtain a reliable fit due to one of the criteria: the 95% confidence interval of either T_f or I_f included 0; NRMSD>0.29; T_f>200 generations; I_f<0.5% or the standard error of T_f>50 generations.

\+ only mean founder ages were reported in Reich et al. (2009) and hence confidence intervals are not shown.

NA For Siddi, sample size differed from the original study and was less than 4, hence *ASCEND* was not applied to this group.

* IC = population is on the Indian Cline as reported in Reich et al.; NIC = population is not on the Indian Cline.

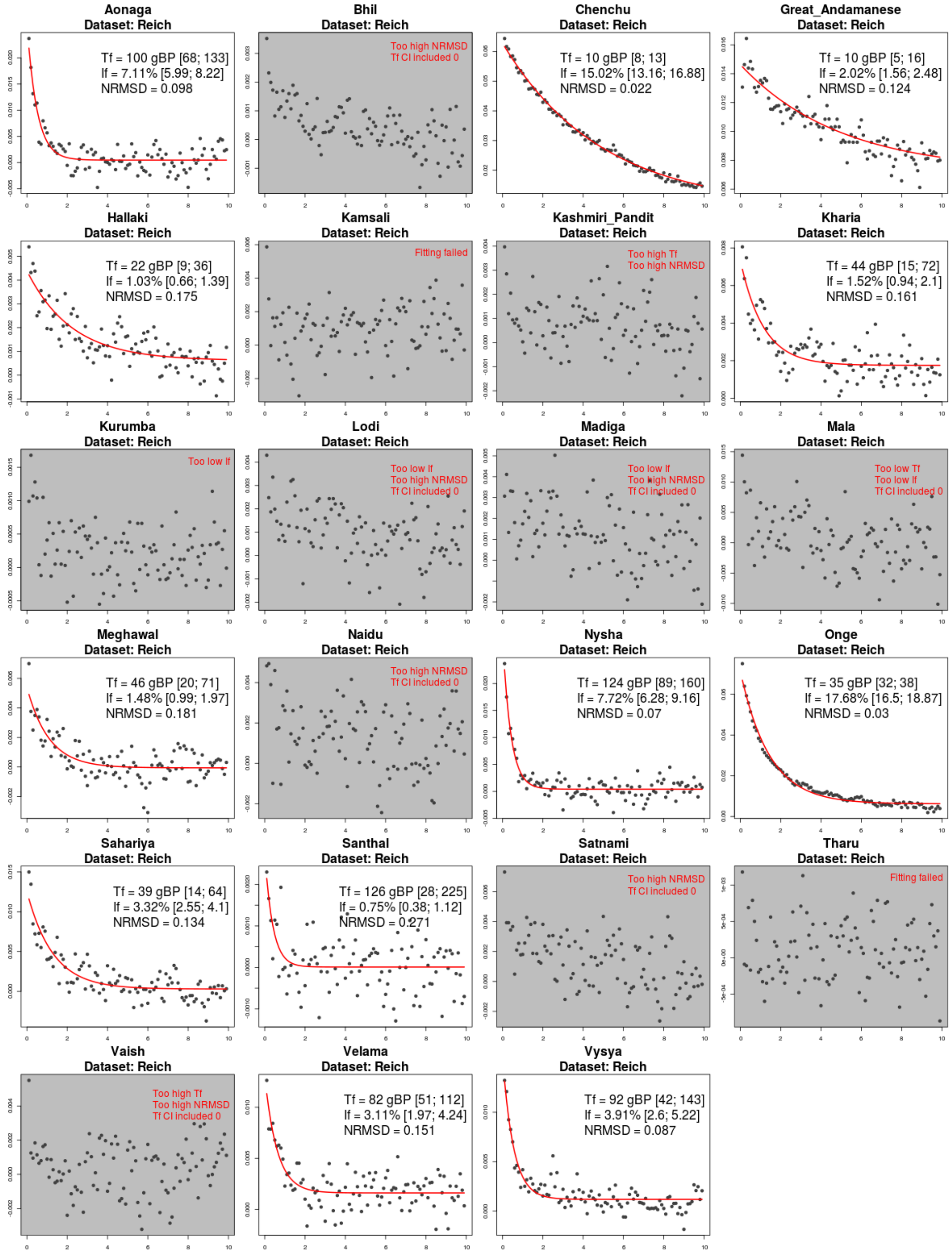

Fig Z - Allele sharing correlation curves for all the samples from Reich et al. that were analyzed with *ASCEND* considering a **0.1–10 cM** distance range. The parameters of *ASCEND* and analysis setup matched those in [11]. The *x*-axis shows the genetic distance in cM and the *y*-axis shows the mean allele sharing correlation. The black points represent the values calculated on the empirical dataset. The red curve represents the exponential fit. For each population panel, we report the estimated mean with the 95% confidence interval for the founder age T_f (in generations) and the founder intensity I_f, and the value of NRMSD (a measure of the quality of the fitted exponential). The panel is grayed if the exponential fit failed or if the inference was unreliable (i.e., the 95% confidence interval of either T_f or I_f included 0; NRMSD>0.29; T_f>200 generations; I_f<0.5% or the standard error of T_f>50 generations).

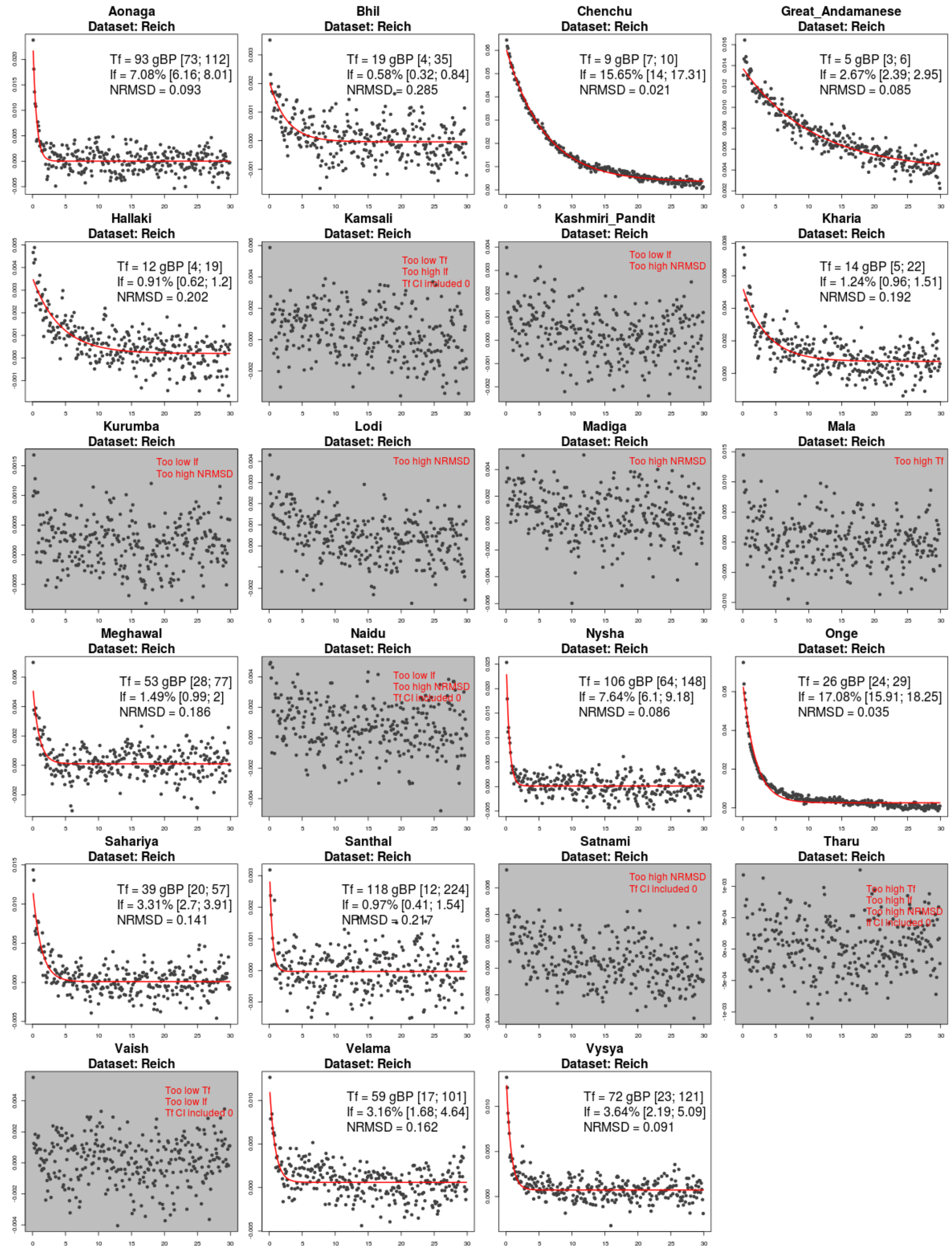

Fig AA - Allele sharing correlation curves for all the samples from Reich et al. that were analyzed with *ASCEND* considering a **0.1–30 cM** distance range. The *x*-axis shows the genetic distance in cM and the *y*-axis shows the mean allele sharing correlation. The black points represent the values calculated on the empirical dataset. The red curve represents the exponential fit. For each population panel, we report the estimated mean with the 95% confidence interval for the founder age T_f (in generations) and the founder intensity I_f, and the value of NRMSD (a measure of the quality of the fitted exponential). The panel is grayed if the exponential fit failed or if the inference was unreliable (i.e., the 95% confidence interval of either T_f or I_f included 0; NRMSD>0.29; T_f>200 generations; I_f<0.5% or the standard error of T_f>50 generations).

S5.2. Comparison of founder intensity and IBD scores from Nakatsuka et al. (2017)

Nakatsuka et al. (2017) introduced the Identity-By-Descent (IBD) score to measure the relative strength of a founder event. Specifically, the IBD score measures the average length of IBD segments that fall within the range of 3–20 cM and which are shared across pairs of individuals in a population, normalized by the number of pairwise comparisons (related to the sample size). To obtain a relative IBD score, this estimate is divided by the IBD score calculated in Finns or Ashkenazi Jews [7]. Using this approach, Nakatsuka et al. identified 81 South Asian groups in the IndiaHO dataset that had an IBD score higher than estimated in Finns and Ashkenazi Jews (AJ).

To infer the strength of the founder event, *ASCEND* estimates the founder intensity (I_f) which is related to the population size and the duration of the founder event. This parameter qualitatively captures the same signal as the IBD score, although I_f is an absolute measure of founder strength compared to the IBD score which is reported as a ratio to the score estimated in a reference population. To compare the two estimates, we applied *ASCEND* to 116 groups in the IndiaHO dataset that had a sample size of at least 5 individuals (using default parameters in *ASCEND*). Among these 116 groups, we obtained significant evidence for a founder event in 66 populations (Methods). We observed that the strength of the founder event estimated using I_f in *ASCEND* and the IBD score in [7] were strongly correlated (Pearson's r=0.95, $P<10^{-5}$) (Fig AB, Table G).

Among the 65 populations (excluding the AJ used for comparison) with significant founder events, 16 groups had more extreme founder events (with significantly higher intensity) than AJ compared to 32 groups reported in Nakatsuka et al. As *ASCEND* only focuses on founder events that occurred recently (within the past 200 generations), it is expected that we miss some founder events captured by Nakatsuka et al.

Table G - Comparison of the IBD scores published in Nakatsuka et al. (2017) with the founder intensities calculated with *ASCEND* for 66 populations that had evidence of founder event according to both methods. The populations are sorted by increasing order of IBD score.

**Population**	**Nakatsuka et al. (2017)**	**This study (using *ASCEND*)**
	**IBD Score [95% CI]**	**If [95% CI]**
Kondh_TN	0.05 [0–0.11]	1.9% [1.3%–2.6%]
Thakur	0.11 [0–0.15]	0.5% [0.3%–0.7%]
Rajbanshi	0.23 [0–0.32]	1.2% [0.8%–1.6%]
Meena	0.27 [0–0.48]	1.2% [0.6%–1.7%]
Bhil	0.28 [0–0.42]	0.6% [0.4%–0.7%]
Kunabi	0.36 [0–0.65]	1.6% [0.7%–2.5%]
Khairwar	0.38 [0–0.56]	1.8% [1.4%–2.2%]
Handigodu	0.45 [0–0.61]	1.3% [1.1%–1.5%]
Panta_Kapu	0.46 [0–0.53]	0.6% [0.5%–0.8%]
Makrani	0.56 [0–0.64]	0.8% [0.7%–0.9%]
Patel	0.64 [0–0.83]	1.3% [0.9%–1.8%]
Bhumij_Orissa	0.64 [0–0.86]	1.7% [1.2%–2.2%]
Balochi	0.7 [1–0.81]	0.9% [0.7%–1%]

Magar	0.71 [1–0.8]	1.5% [1.4%–1.7%]
GujaratiD	0.78 [1–1]	2.2% [1.4%–3%]
Hazara	0.84 [1–1.01]	0.6% [0.5%–0.8%]
Kamboj	0.86 [1–0.94]	1.1% [1%–1.2%]
Jew_Ashkenazi	0.88 [1–1.16]	2% [1.6%–2.5%]
Minero	0.88 [1–1.2]	0.7% [0.6%–0.9%]
Brahmin_Catholic_Goa	0.89 [1–1.04]	0.6% [0.5%–0.7%]
Brahmin_Catholic_Kumta	0.9 [1–1.09]	0.6% [0.4%–0.8%]
Newar	0.9 [0–1.4]	1.7% [1%–2.4%]
Nadar	0.92 [1–1.07]	1.3% [1.1%–1.4%]
Oswal_Jain	0.98 [1–1.4]	1.7% [1.5%–2%]
Punjabi	1.05 [1–1.28]	0.9% [0.5%–1.3%]
Kondakamari	1.12 [1–1.34]	1.6% [1.4%–1.7%]
Hallaki	1.15 [1–1.43]	0.8% [0.6%–1%]
Manjhi_Jharkhand	1.21 [1–1.67]	1.6% [1.1%–2.1%]
Vysya	1.22 [1–1.31]	2.5% [2.4%–2.7%]
Arunthathiyar	1.26 [1–1.44]	0.8% [0.6%–0.9%]
Garasia	1.28 [1–1.83]	1.4% [1.1%–1.8%]
Ho_Orissa	1.32 [1–1.43]	1.1% [0.9%–1.2%]
Jogi	1.47 [1–1.92]	1% [0.9%–1.2%]
Sahariya_MP	1.54 [1–1.93]	1.7% [1.2%–2.1%]
Batudi	1.55 [1–2.01]	1.4% [1%–1.7%]
Yerukali	1.62 [1–1.98]	2% [1.6%–2.3%]
Kallar	1.68 [1–1.88]	1.2% [1.1%–1.2%]
Brahui	1.82 [2–2.02]	1.2% [1.1%–1.2%]
Burusho	1.85 [2–2.1]	0.8% [0.8%–0.9%]
Kharia	1.87 [1–2.25]	0.8% [0.7%–1%]
Reddy_Telangana	1.96 [1–2.57]	1% [0.8%–1.2%]
Bharia	2.18 [2–2.78]	1.5% [1.2%–1.8%]
Kumhar	2.27 [2–2.5]	3.1% [3%–3.3%]
Irula	2.47 [2–2.79]	3% [2.8%–3.3%]
Havik	2.98 [2–3.52]	2.7% [2.2%–3.1%]
Juang	3.07 [3–3.4]	2.6% [2.4%–2.8%]
Hindumalayali	3.68 [3–4.66]	2.4% [1.9%–2.9%]
Asur	4.17 [3–4.87]	2.2% [1.9%–2.5%]
Parhaiya	4.38 [3–5.32]	4.2% [3.5%–4.9%]
Yadav_Pondicherry	4.4 [4–5.04]	1.4% [1.2%–1.6%]
Gorait	4.68 [4–5.82]	1.6% [1.4%–1.8%]
Palliyar	5.11 [5–5.51]	3% [2.9%–3.2%]
Kotwalia	5.55 [4–6.92]	2.7% [2.2%–3.3%]
Kurumans	6.49 [5–7.78]	5.9% [5.4%–6.5%]
Bhunjiya	6.53 [5–7.94]	3.9% [3.4%–4.4%]
Kusunda	7.29 [6–8.33]	2.6% [2.3%–2.9%]
Kanjad	7.8 [6–9.27]	3.2% [2.7%–3.6%]
Mohali	9.12 [8–10.04]	3.1% [2.9%–3.4%]
Gujjar	11.63 [9–14.59]	4.6% [3.8%–5.5%]
Kalash	12.45 [11–13.64]	5.6% [5.4%–5.9%]

Pulliyar	16.71 [15–18.59]	6.8% [6.5%–7.1%]
Hakki_Pikki	25.56 [22–29.57]	11.2% [10.3%–12.2%]
Narikuravar	25.71 [23–28.82]	10.6% [9.3%–12%]
Ulladan	25.89 [23–28.61]	12.4% [11.7%–13.1%]
Onge	31.1 [28–33.89]	20.6% [19.6%–21.7%]
Malaikuravar	34.13 [29–38.98]	14.7% [12.7%–16.7%]

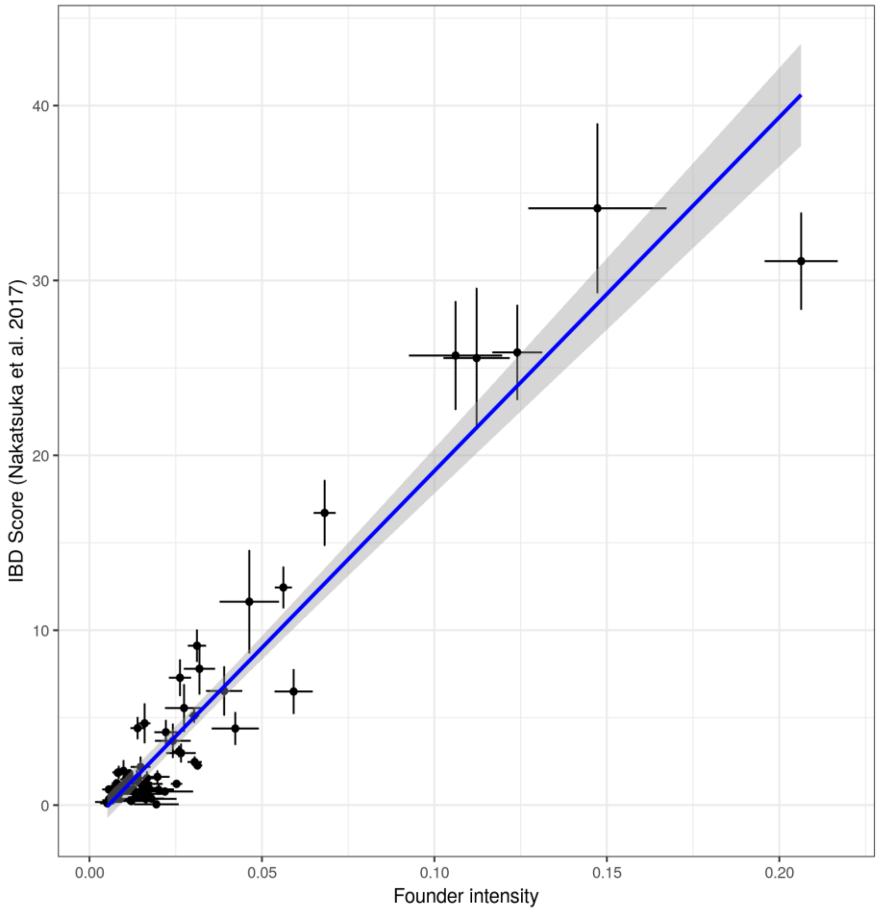

Fig AB - Comparison of the IBD scores published in Nakatsuka et al. (2017) with the founder intensities estimated using *ASCEND*. We report results for the comparison of 66 populations for which we obtained reliable estimates using both methods. The bars represent the 95% confidence intervals and the blue line is the linear regression line (Pearson's r=0.95, P<10^{-5}).

S5.3. *IBDNe* analysis

IBDNe is a non-parametric approach that leverages the distribution of IBD segments to recover the T_{MRCA} and estimate population size at various ages in the past [9]. To investigate the performance of *IBDNe* using small sample sizes as available in our study, we applied *IBDNe* to 58 populations from the IndiaHO dataset which had a sample size greater or equal to 5 (S1 Table). We identified IBD segments for all samples using the approach described earlier (Methods, Notes S3). For each population, we ran *IBDNe* v.6a4 using the HapMap Phase 2 recombination map. To minimize the noise in the inference, we removed 58 populations that had less than 20 called IBD segments. We set the parameters as default, i.e., filtering out close relatives, performing 1,000 iterations, 80 bootstraps for the variance calculation, minimum IBD segment length of 2 cM, a length of 0.2 cM trimmed from each end of each chromosome and limiting to founder events that occurred between 2–300 generations.

```
cat $POP.ibd | java -jar ibdne.07May18.6a4.jar \
map=genetic.map out=$POP filtersamples=true nits=1000 \
nboots=80 mincm=2 trimcm=0.2 gmin=2 gmax=300
```

Out of the 58 populations, four failed the *IBDNe* analysis, leaving 54 groups for which we could estimate the N_e trajectory. We found that with the exception of Sindhis from Pakistan, all of the 54 populations showed evidence of population decline in the last hundred generations with decreasing N_e towards the recent past (Fig AC). The estimated effective population sizes were fairly high (ranging between 10–10^{28}) over the past 50 generations. All populations except one had effective population sizes (not census sizes) >10^6 by 35 generations ago (Fig AC) and monotonically increasing thereafter, which is unexpected and extremely large. These estimates seem in contradiction with the results we obtained using *ASCEND* and with further evidence from previous studies which reported strong bottlenecks in South Asians using the same samples (Table H) [7]. These large population sizes at recent timescales are likely due to computational phasing errors (switch errors) in sparse datasets with few samples. Moreover, small sample sizes constrain the number and type of IBD tracts detected (smaller segments are more likely to be missed), leading to biased IBD distributions and in turn, inaccurate estimation of population size variation over time. Similar patterns were noted previously in the analysis of Ashkenazi Jews and Maasai using *DoRIS* [10]. This empirically demonstrates the challenges of inferring population bottlenecks when applying IBD-based methods to sparse datasets.

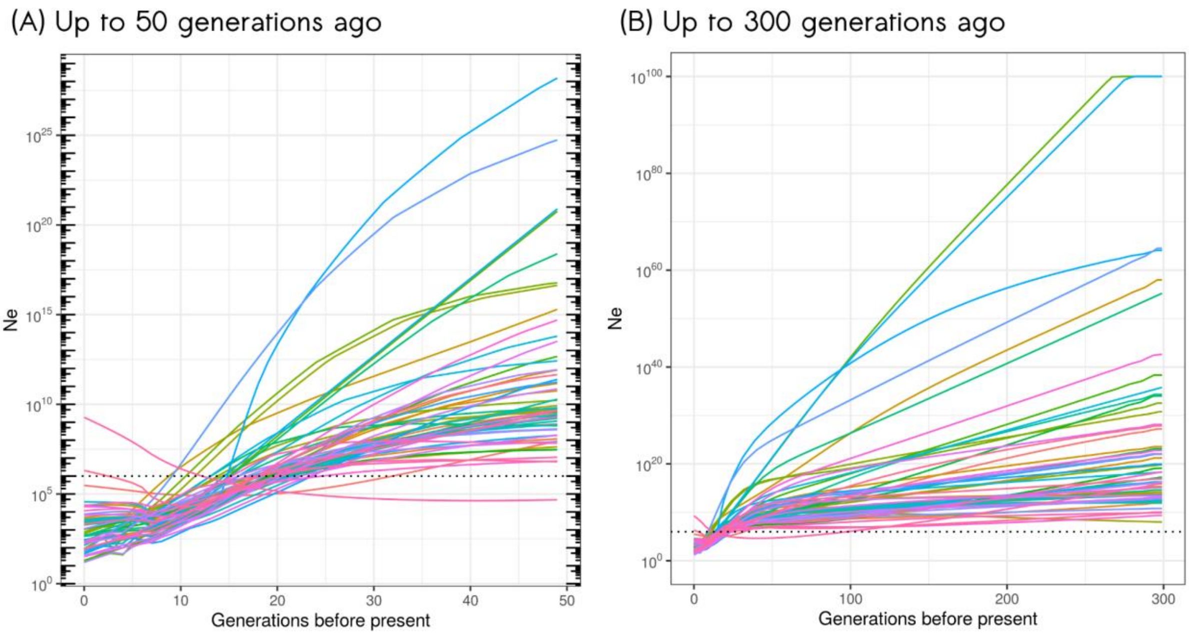

Fig AC - Effective population size trajectories as inferred with *IBDNe* for 54 populations from the phased IndiaHO dataset. The *y*-scale is in logarithmic scale in both plots. The horizontal dotted line is located at N_e = 10^6. **(A)** Representing the trajectories from present up to 50 generations BP (i.e., zoom towards the present). **(B)** Representing the trajectories from present up to 300 generations BP (i.e., full temporal scale of inference).

Table H - This table lists the 54 populations from IndiaHO that were analyzed with *IBDNe*. We provide the arithmetic mean, minimum and maximum values of the inferred N_e trajectories during the last 300 generations. Populations are sorted by increasing order of minimum N_e (i.e., equivalent to a decreasing order of bottleneck strength).

		$\mathbf{N_e}$ (last 200 generations)		
**Population**	**Number of IBD segments**	**Mean**	**Minimum**	**Maximum**
**Malaikuarvar**	288	6.31E+20	16	1.28E+22
**Gujjar**	125	1.09E+99	18	1.00E+100
**Hakki_Pikki**	215	6.24E+36	20	2.23E+38
**Onge**	556	1.11E+14	33	1.23E+15
**Narikuravar**	156	6.00E+26	36	1.36E+28
**Kotwalia**	63	7.21E+98	46	1.00E+100
**Hindumalayali**	31	1.98E+53	57	1.70E+55
**Kanjad**	141	1.14E+34	61	6.26E+35
**Lodhi**	35	6.07E+62	62	3.21E+64
**Kusunda**	125	4.41E+20	65	9.88E+21
**Bharia**	42	1.92E+13	75	5.63E+13
**Gorait**	87	9.36E+30	81	3.46E+32
**Yadav_Pondicherry**	346	6.02E+15	84	7.89E+16
**Bhunjiya**	61	6.59E+19	85	1.61E+21
**Parhaiya**	39	9.56E+40	92	4.08E+42
**Kharia**	144	1.38E+17	137	1.92E+18
**Mohali**	836	1.02E+13	182	2.61E+13
**Kurumans**	44	5.39E+18	183	8.83E+19
**Reddy_Telangana**	33	6.49E+16	197	2.33E+18
**Chaurasia**	25	1.88E+29	218	6.12E+30
**Minero**	48	7.36E+14	219	1.63E+16
**Asur**	189	5.89E+12	232	5.66E+13
**Hazara**	54	6.31E+15	243	1.80E+17
**Kalash**	790	5.91E+11	287	1.88E+12
**Havik**	20	5.45E+21	437	1.36E+23
**Ho_Orissa**	203	3.08E+13	462	3.99E+14
**Jogi**	32	6.92E+32	489	2.19E+34
**Hallaki**	25	4.50E+17	491	1.35E+19
**Brahmin_Tiwari**	25	1.87E+56	582	9.69E+57
**Gond_MP**	51	3.07E+18	610	6.37E+19
**Chakkiliyan**	66	2.80E+09	764	8.82E+09
**Arunthathiyar**	143	1.13E+14	1080	1.99E+15
**Handigodu**	24	3.18E+32	1100	1.08E+34
**Makrani**	62	6.14E+11	1150	6.51E+12
**Juang**	423	3.82E+11	1390	1.02E+12
**Burusho**	267	1.50E+13	1740	1.45E+14
**Irula**	124	1.05E+15	1890	1.70E+16
**Vishwabrahmin**	36	9.89E+08	2050	1.09E+10
**Kallar**	427	5.04E+12	2360	3.81E+13
**Kumhar**	474	3.82E+62	2380	1.17E+64
**Brahui**	134	5.57E+11	2450	4.36E+12
**Brahmin_Catholic_Goa**	40	1.74E+22	2770	3.72E+23
**Kondakamari**	87	1.27E+15	2820	1.97E+16
**Nadar**	59	1.21E+12	3010	1.20E+13
**Balochi**	53	6.16E+10	5010	7.87E+11
**Magar**	184	1.17E+10	5420	6.23E+10
**Panta_Kapu**	29	2.60E+21	5500	5.74E+22
**Kamboj**	344	5.28E+11	5940	3.30E+12
**Brahmin_Vaidik**	88	1.27E+14	9980	2.06E+15
**Pathan**	20	2.25E+08	10100	2.29E+09
**Sikh_Jatt**	205	1.82E+09	19100	8.01E+09
**Vysya**	223	3.11E+26	21400	7.39E+27
**Sindhi_Pakistan**	20	2.55E+14	42500	7.84E+15
**Agarwal**	103	5.83E+25	77700	1.66E+27

S6. History of founder events in dogs

To study founder events in dogs, we applied *ASCEND* to two publicly available genetic datasets: (i) the **Sams dataset** comprising 1,792 individuals from 11 breeds genotyped on 175,123 SNPs (https://figshare.com/articles/dataset/Supplementary_Material_for_Sams_and_Boyko_2018/7330151) [13], and (ii) the **Hayward dataset** comprising 4,342 individuals from 198 breeds genotyped on 160,723 SNPs (https://datadryad.org/stash/dataset/doi:10.5061/dryad.266k4) [12]. We used the genetic positions from the CanFam3.1 genetic map [16].

For both datasets, we filtered out SNPs and individuals with missingness greater than 1% and 5% respectively. To remove close relatives, we computed the pairwise genetic sharing π between all pairs of individuals using PLINK v1.90b6.2 *genome* module and we excluded one individual for each pair of individuals with π greater than 45%.

```
plink --vcf XX.vcf --genome --min 0.05 --double-id --dog
```

To control for inbreeding, we excluded any individual with large regions of runs of homozygosity (ROH) longer than 30.2 Mbp (~30 cM) which represents roughly half the size of an average chromosome in dogs. To this end, we ran PLINK *homozyg* module:

```
plink --vcf XX.vcf --homozyg group --homozyg-kb 30000 --
homozyg-match 0.99 --out XX.ROH --double-id --dog
```

After applying this filter, we considered only those populations with a sample size equal or greater than 5 individuals. We analyzed 42 unique dog breeds (1,097 individuals) across the two datasets (S5 Table, S6 Fig for all the decay curves). Overall, we found that 40 out of the 42 dog breeds have evidence of a significant founder event, with a mean at 25.3% [21.1%–29.6%] and a maximum of 77.7% estimated in Boxers (Fig 4). The founder ages range from 6 generations (Gordon Setter) to 24 generations (Bulldog) or 18–120 years ago assuming a generation time of 3–5 years [14,15]. We compared the founder parameter estimates for 10 overlapping breeds present in both datasets and obtained statistically similar results (Fig AD, S6 Table). Both the founder age and founder intensity were highly correlated across the two datasets (Pearson’s r>0.90, P<0.0005).

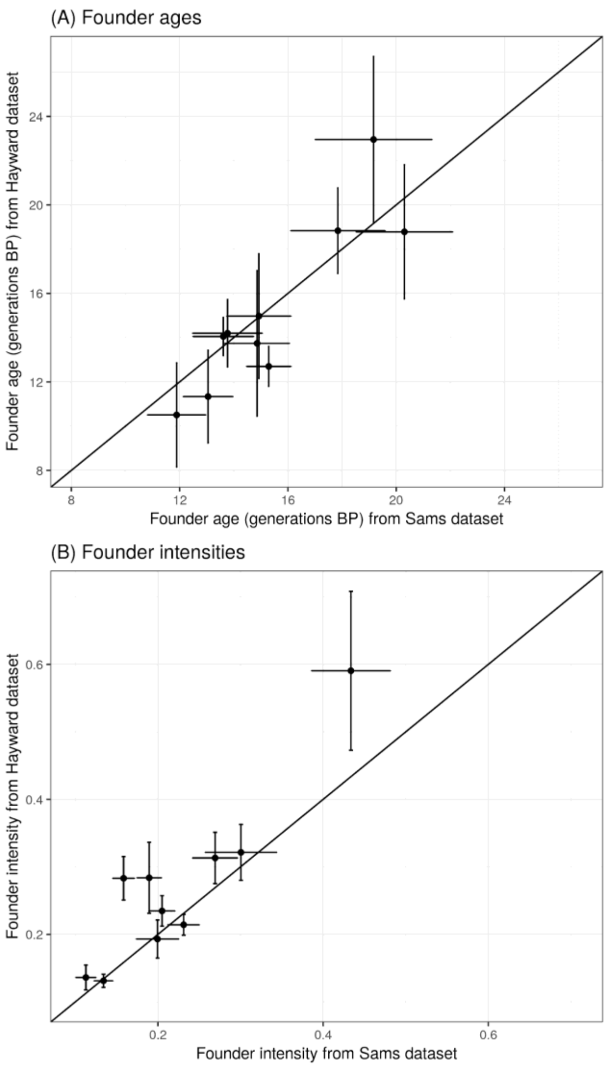

Fig AD - Comparison in the estimates of founder ages **(A)** and founder intensities **(B)** for the 10 dog breeds present in both the Sams (*x*-axis) and Hayward (*y*-axis) datasets. The black oblique line is the *y*=*x* diagonal.

S7. Reference

1. Adrion, J. R., Cole, C. B., Dukler, N., Galloway, J. G., Gladstein, A. L., Gower, G., ... & Kern, A. D. (2020). A community-maintained standard library of population genetic models. Elife, 9, e54967.
2. Allen Ancient DNA Resource (AADR). https://reich.hms.harvard.edu/allen-ancient-dna-resource-aadr-downloadable-genotypes-present-day-and-ancient-dna-data
3. 1000 Genomes Project Consortium, Adam Auton, Lisa D. Brooks, Richard M. Durbin, Erik P. Garrison, Hyun Min Kang, Jan O. Korbel, et al. 2015. "A Global Reference for Human Genetic Variation." Nature 526 (7571): 68–74.
4. Durand, Eric Y., Nicholas Eriksson, and Cory Y. McLean. 2014. "Reducing Pervasive False-Positive Identical-by-Descent Segments Detected by Large-Scale Pedigree Analysis." Molecular Biology and Evolution 31 (8): 2212–22.
5. Gusev, Alexander, Jennifer K. Lowe, Markus Stoffel, Mark J. Daly, David Altshuler, Jan L. Breslow, Jeffrey M. Friedman, and Itsik Pe'er. 2009. "Whole Population, Genome-Wide Mapping of Hidden Relatedness." Genome Research 19 (2): 318–26.
6. Loh, Po-Ru, Petr Danecek, Pier Francesco Palamara, Christian Fuchsberger, Yakir A Reshef, Hilary K Finucane, Sebastian Schoenherr, et al. 2016. "Reference-Based Phasing Using the Haplotype Reference Consortium Panel." Nature Genetics 48 (11): 1443–48.
7. Nakatsuka, Nathan, Priya Moorjani, Niraj Rai, Biswanath Sarkar, Arti Tandon, Nick Patterson, Gandham Srilakshmi Bhavani, et al. 2017. "The Promise of Discovering Population-Specific Disease-Associated Genes in South Asia." Nature Genetics 49 (9): 1403–7.
8. Patterson, Nick, Alkes L. Price, and David Reich. 2006. "Population Structure and Eigenanalysis." PLoS Genetics 2 (12): e190.
9. Browning, Sharon R., and Brian L. Browning. 2015. "Accurate Non-Parametric Estimation of Recent Effective Population Size from Segments of Identity by Descent." American Journal of Human Genetics 97 (3): 404–18.
10. Palamara PF, Lencz T, Darvasi A, Pe'er I. 2012. Length distributions of identity by descent reveal fine-scale demographic history. Am J Hum Genet 91: 809–822.
11. Reich, D., Thangaraj, K., Patterson, N., Price, A. L., & Singh, L. (2009). Reconstructing Indian population history. Nature, 461(7263), 489–494.
12. Hayward, Jessica J., Marta G. Castelhano, Kyle C. Oliveira, Elizabeth Corey, Cheryl Balkman, Tara L. Baxter, Margret L. Casal, et al. 2016. "Complex Disease and Phenotype Mapping in the Domestic Dog." Nature Communications 7 (January): 10460.
13. Sams, Aaron J., and Adam R. Boyko. 2019. "Fine-Scale Resolution of Runs of Homozygosity Reveal Patterns of Inbreeding and Substantial Overlap with Recessive Disease Genotypes in Domestic Dogs." G3 9 (1): 117–23.
14. Vonholdt, Bridgett M., Daniel R. Stahler, Douglas W. Smith, Dent A. Earl, John P. Pollinger, and Robert K. Wayne. 2008. "The Genealogy and Genetic Viability of Reintroduced Yellowstone Grey Wolves." Molecular Ecology 17 (1): 252–74.
15. Wang, Guo-Dong, Weiwei Zhai, He-Chuan Yang, Ruo-Xi Fan, Xue Cao, Li Zhong, Lu Wang, et al. 2013. "The Genomics of Selection in Dogs and the Parallel Evolution between Dogs and Humans." Nature Communications 4: 1860.
16. Auton A, Rui Li Y, Kidd J, Oliveira K, Nadel J, Holloway JK, et al. Genetic recombination is targeted towards gene promoter regions in dogs. PLoS Genet. 2013;9: e1003984.
