# Supplementary material for: Reconstructing the history of founder events using genome-wide patterns of allele sharing across individuals
Source: PLoS Genet. 2022 Jun 23;18(6):e1010243. doi: 10.1371/journal.pgen.1010243 (PMC9223333; doi:10.1371/journal.pgen.1010243)
Supplement: Attachment — Submitted filename: ReviewPlosGenetics.docx [file pgen.1010243.s015.docx]

**Main text:**

**Here the authors present and implement a new method, ASCEND, to estimate the intensity of founder events and timing through allele sharing within groups. This manuscript nicely collates work from different lines of human demography and non-human (dog) demography. Overall, I thought the paper read well and the authors synthesized a lot of information in a clear concise way. I only have one major comment and a series of minor comments that I split up by section below.**

**Major Comment:**

Since the boxer genome is the reference genome for the dogs, I think there is likely an inflation of homozygotes being called in data. Given this, I am wondering if the reason it has the most intense founding events with second largest reported standard errors (Table S5) is just because it is the reference genome? I think this is also important to double check since the authors mention incidence of recessive disease in the discussion. Perhaps the authors could add an additional simulation where reference bias is incorporated? I am wondering if this might be close to what was done when considering the pseudo haploid genomes with aDNA?

**Minor Comments:**

**Main Text**

Minor formatting thing, in the main text authors alternate from no space to a single space between a word and the citation. I think either reads fine as long as it is consistent throughout.

Page 3 of the introduction, when the authors talk about commonly used IBD-based methods. I don’t think that you necessarily need phase information for IBDNe because if you use input from IBDSeq my understanding is that there is no phasing of the haplotypes. I believe this is an attempt to reduce chances of the switch errors that the authors mention. However the drawback is that you can’t call the smaller segments (so you still lose information). What the authors said is correct but maybe rephrase the sentence for readers who may not be completely familiar with IBDNe?

Page 5, add space between equal sign and 10 in the second paragraph.

Page 8, for putting together results about the Jewish population history there might be good supporting information in these two papers *North African Jewish and non-Jewish populations form distinctive, orthogonal clusters* (Campbell et al. 2012 *PNAS*) & *Consanguinity Rates Predict Long Runs of Homozygosity in Jewish Populations* (Kang et al. 2016 *Human Heredity*). The results seem to fit well with their conclusions and potentially provide evidence for being able to distinguish between a founder event and recent inbreeding when considering results from Kang paper.

Page 8, for Island vs. continental group, just for clarification, do the authors think it reliable to use the Oceanian sample since there are only four individuals?

**Methods**

Simulations: How many replicates were run for each of the simulation scenarios? Maybe I missed this somewhere in the supplement or main text, but I did not see it mentioned in the section where all the other simulation parameters were specified.

**Figures**

Figure 3, add the yBP abbreviation to the figure caption. I believe there is a sentence that mentions it already.

Figure 4, can the authors add what the AKC designations to the figure? Maybe below each image?

**Data section**

Add locations for dog data

**Supplementary materials**

For supplementary text, this may have gotten scrambled along the way, but I don’t think any of the associated LaTeX figure or equation numbers rendered correctly. For example all of my figure and equation references in the pdf currently read as Figure (??) or Eqn(??).

Simulations:

- Roughly same comment here as main text, can authors mention the number of simulations for each scenario?
- One more question, can the authors elaborate on the results for the no recovery after founder event specifically when the event was 200 generations ago? It looks like overall, the founder intensity is under-estimated, no matter the scenario (this makes sense). However, it also looks like estimates when Tf was 200 generations all converge into roughly the same estimate? Any thoughts on what could cause this?

For IBDNe analyses: I am wondering if authors considered that one of the potential reasons IBDNe estimates of NE were so large in the recent years is due to the sample size used for IBDNe. When looking at Table S1 the largest sample size for IndiaHO is approximately 40 individuals. For IBDNe I believe the suggestion for accurate estimates of NE is using sample sixes in roughly the hundreds?

Figure S2.1.1, can the authors define gBP in the caption?

For all the figures with allele sharing curves, can the authors add x and y axes labels and increase font sizes for both axes? I know there are a lot of these, but they are very informative for readers who really want to dig into the supplementary information and they are hard to read at the moment.
