# Supplementary material for: Reconstructing the history of founder events using genome-wide patterns of allele sharing across individuals
Source: PLoS Genet. 2022 Jun 23;18(6):e1010243. doi: 10.1371/journal.pgen.1010243 (PMC9223333; doi:10.1371/journal.pgen.1010243)
Supplement: Attachment — Submitted filename: Response_Reviewers.docx [file pgen.1010243.s016.docx]

# Editor Comments

Thank you for submitting your manuscript to PLoS Genetics. It has now been evaluated by three reviewers. All three were quite positive about ASCEND and its potential impact to the field. Further, all were impressed by the detailed simulation studies performed to assess the performance of the method. I concur with their assessment. While the reviewers were generally positive, they had a number of suggestions for additional simulations and clarifications to be made in the text. Reviewer 3 suggests performing simulations without a founder event and assessing the performance of ASCEND. Reviewer 2 suggests examining performance of ASCEND when there has been admixture more recently than the founder event. I think both of these situations are important to investigate with additional simulations. I do not think that you need to consider many scenarios for each of these types of models. Rather, just one or two cases that you think are reasonable should suffice. Please address the other more minor comments from all three reviewers as well. I look forward to receiving your revised manuscript.

We thank the editor and the three reviewers for their positive comments. We have added two additional simulations addressing the reviewers’ concerns (Supplementary Notes S2.4.2 and S2.8). We have also uploaded a tutorial with the software package on github, updated the figures and clarified the text where necessary. We provide a detailed point-by-point response to the reviewer’s comments below and track changes for all edits in the manuscript. We note that for some supplementary notes that were written in LaTEX, we were unable to track changes, though we provide a document comparison for reference.

# Reviewer #1

**Main text:**

**Here the authors present and implement a new method, ASCEND, to estimate the intensity of founder events and timing through allele sharing within groups. This manuscript nicely collates work from different lines of human demography and non-human (dog) demography. Overall, I thought the paper read well and the authors synthesized a lot of information in a clear concise way. I only have one major comment and a series of minor comments that I split up by section below.**

**Major Comment:**

Since the boxer genome is the reference genome for the dogs, I think there is likely an inflation of homozygotes being called in data. Given this, I am wondering if the reason it has the most intense founding events with second largest reported standard errors (Table S5) is just because it is the reference genome? I think this is also important to double check since the authors mention incidence of recessive disease in the discussion. Perhaps the authors could add an additional simulation where reference bias is incorporated? I am wondering if this might be close to what was done when considering the pseudo haploid genomes with aDNA?

We thank the reviewer for highlighting this important point. We have investigated how the SNPs were ascertained for the CanineHD array that we used in our analysis. Indeed, the array includes a subset of SNPs that were ascertained to be heterozygous in Boxers (Boyko et al. 2008; Pickrell et al. 2012; Parker et al. 2017). Further, some minor bias could be expected since the reference genome for dogs is based on a Boxer individual, though a large part of the SNPs were identified based on multi-breed resequencing data. We considered carrying out a simulation study to investigate the robustness of our results to this complex ascertainment bias. However, reproducing the empirical ascertainment bias using simulations would be very challenging because population history likely exacerbates ascertainment issues, and we would thus need a detailed picture of the Boxer history along with a good understanding of the sequencing details, SNP selection criteria, etc. In a recent study, Parker et al. (2017) empirically investigated the impact of ascertainment bias on measuring minor allele frequencies and haplotype sharing in Boxers and found some bias in estimated rates, though they noted that these biases should have minimal impact on other breeds.

Thus, we have added the following statement to the results.

“(Note, a subset of SNPs used in this analysis were ascertained in Boxers and the canid reference genome (CamFam) also derives from a Boxer individual. These factors could lead to increased power to detect founder events in Boxers, though it should have minimal impact on other breeds [[52–54]](https://paperpile.com/c/DwONRu/z0Kl9+hcfsU+EQjzq)).”

**Minor Comments:**

**Main Text**

Minor formatting thing, in the main text authors alternate from no space to a single space between a word and the citation. I think either reads fine as long as it is consistent throughout.

We have now homogenized the spacing between words and citations in the whole manuscript.

Page 3 of the introduction, when the authors talk about commonly used IBD-based methods. I don’t think that you necessarily need phase information for IBDNe because if you use input from IBDSeq my understanding is that there is no phasing of the haplotypes. I believe this is an attempt to reduce chances of the switch errors that the authors mention. However the drawback is that you can’t call the smaller segments (so you still lose information). What the authors said is correct but maybe rephrase the sentence for readers who may not be completely familiar with IBDNe?

We agree with the reviewer and have clarified the text as follows:

“Most commonly used IBD-based methods, e.g. DoRIS [[16]](https://paperpile.com/c/DwONRu/KggJW) and IBDNe [[15]](https://paperpile.com/c/DwONRu/kQl5u), recommend the use of phased data that can be obtained from computational phasing of population data; however, this typically requires large numbers of high quality samples. Errors in computational phasing (“switch errors”) can result in biased estimates of IBD segment lengths, and in turn population size inference in real data [[16]](https://paperpile.com/c/DwONRu/KggJW). As the rate of phasing errors is inversely proportional to the sample size and the length of IBD segments [[17]](https://paperpile.com/c/DwONRu/wlBQY), IBD-based methods tend to be noisy and less accurate at older timescales and when using sparse datasets with small sample sizes or limited coverage such as ancient genomes.”

Page 5, add space between equal sign and 10 in the second paragraph.

Done.

Page 8, for putting together results about the Jewish population history there might be good supporting information in these two papers *North African Jewish and non-Jewish populations form distinctive, orthogonal clusters* (Campbell et al. 2012 *PNAS*) & *Consanguinity Rates Predict Long Runs of Homozygosity in Jewish Populations* (Kang et al. 2016 *Human Heredity*). The results seem to fit well with their conclusions and potentially provide evidence for being able to distinguish between a founder event and recent inbreeding when considering results from Kang paper.

We thank the reviewer for pointing us to these references. We have added some discussion sentences in the results.

“*Jewish communities*: Our dataset includes samples from 11 Jewish groups, including Ashkenazi, Caucasus (Georgian), Middle Eastern (Iranian and Iraqi), African (Moroccan, Libyan, Tunisian and Ethiopian), Sephardi (Turkish) and Indian (Cochin) Jewish communities. We observed significant evidence of founder events in most Jewish groups, except in Ethiopian and Turkish Jews (Figure S2). While many studies have focused on understanding the founder events in AJ, we found that the founder intensity estimated in most other Jewish groups was higher than in AJ, with the exception of Middle Eastern Jews. These results are in line with previous results based on runs of homozygosity (ROH) or IBD analysis [[31,32]](https://paperpile.com/c/DwONRu/Q3wRM+82CQG). The estimated founder ages varied significantly across Jewish communities, ranging between 280–1,300 years ago, highlighting recent population bottlenecks that have impacted the genetic diversity in each group, but still older than what could be expected by consanguineous marriages with the presence of very long runs of homozygosity (ROH) [[32]](https://paperpile.com/c/DwONRu/82CQG). The strongest and most recent founder event was inferred in Cochin Jews, with the inferred timing similar to previous reports [[33]](https://paperpile.com/c/DwONRu/X8n8f)*.*”

Page 8, for Island vs. continental group, just for clarification, do the authors think it reliable to use the Oceanian sample since there are only four individuals?

Sorry if our notation was confusing. The *n*=4 for Oceanians refers to the number of populations, not the number of individuals (which equals 36, in this case). We clarified this in the text.

**Methods**

Simulations: How many replicates were run for each of the simulation scenarios? Maybe I missed this somewhere in the supplement or main text, but I did not see it mentioned in the section where all the other simulation parameters were specified.

The simulations presented in the supplementary notes show the result for each simulation of 20 chromosomes of 50 Mbp each. The results we report include the mean and the SE based on the chromosomal jackknife, where we drop one chromosome in each run. As each chromosome is an independent unit in this case, one can view it as the variation across 20 replicates. Further, for some scenarios including single-generation bottleneck, we ran 10 replicates and found the results were qualitatively similar (within overlapping 95% confidence intervals). To clarify, we have added a paragraph in Supplementary Notes S2 and a line in the manuscript, in *Methods* under the subsection “*Simulations*”:

“We applied *ASCEND* to each simulation and reported the mean and the standard error of the parameters based on a chromosome jackknife, where we drop one chromosome in each run. For some scenarios such as the single-generation bottleneck model, we generated data for 10 replicates and assessed the variation across runs. We found that the inferred estimates of founder age and intensity were qualitatively similar across replicates (within overlapping 95% confidence intervals) (Table S2.1.1 and Table S2.1.2).”

**Figures**

Figure 3, add the yBP abbreviation to the figure caption. I believe there is a sentence that mentions it already.

Done.

Figure 4, can the authors add what the AKC designations to the figure? Maybe below each image?

We included the definition of each group next to the images.

**Data section**

Add locations for dog data

Done.

**Supplementary materials**

For supplementary text, this may have gotten scrambled along the way, but I don’t think any of the associated LaTeX figure or equation numbers rendered correctly. For example all of my figure and equation references in the pdf currently read as Figure (??) or Eqn(??).

Sorry for this formatting issue. We have now fixed this issue.

**Simulations**

Roughly same comment here as main text, can authors mention the number of simulations for each scenario?

We have now clarified this information in the main text and supplement.

One more question, can the authors elaborate on the results for the no recovery after founder event specifically when the event was 200 generations ago? It looks like overall, the founder intensity is under-estimated, no matter the scenario (this makes sense). However, it also looks like estimates when Tf was 200 generations all converge into roughly the same estimate? Any thoughts on what could cause this?

The results of the no recovery models are indeed puzzling. As expected, we find that the single exponential is a poor fit to the output in this case and thus the inferred parameters should be interpreted with caution. In most cases, we find we can reject a single exponential model vs. a sum of exponentials for example, though both models would be incorrect for the no-recover model. We believe future studies that can derive a closed form expression of modeling this scenario will be able to infer the *I_f_* and *T_f_* more reliably.

For IBDNe analyses: I am wondering if authors considered that one of the potential reasons IBDNe estimates of NE were so large in the recent years is due to the sample size used for IBDNe. When looking at Table S1 the largest sample size for IndiaHO is approximately 40 individuals. For IBDNe I believe the suggestion for accurate estimates of NE is using sample sixes in roughly the hundreds?

We agree with the reviewer and we think this is the main reason for the large Ne in recent years also. In sparse datasets with few samples, IBDNe does not work reliably for several reasons: phasing errors (also exacerbated in small sample sizes), unreliable fitting of parameters to the IBD tract length distribution, etc. We have clarified the supplement to highlight all issues.

Figure S2.1.1, can the authors define gBP in the caption?

Done.

For all the figures with allele sharing curves, can the authors add x and y axes labels and increase font sizes for both axes? I know there are a lot of these, but they are very informative for readers who really want to dig into the supplementary information and they are hard to read at the moment.

Done for all decay curves of ancient and present-day humans.

# Reviewer #2

Title: Reconstructing the history of founder events using genome-wide patterns of allele sharing across individuals. Authors: Tournebize et al., 2022

Summary: Propose a new method called ASCEND that quantifies correlation of shared alleles between individuals to infer the age and strength of founder events, apply to several large datasets (humans and canids) to infer recent founder events. The algorithm itself has three steps: (1) at each marker, infer the total number of IBS alleles shared across pairs of individuals; (2) compute correlation in shared allele counts across individuals in a population; (3) measure decay of this correlation with genetic distance; expected to have exponential decay with distance, amplitude of decay is ~ strength of bottleneck.

I have reviewed the math and it’s well described and elegant - I have to congratulate the authors on this massive work comprising both simulations and analyses of empirical data.

Claims: 1) Number, 2) Strength, 3) tempo of bottleneck events over history, 4) timing of bottleneck event (founder age).

The proposed approach addresses the strength, and the timing of most recent bottleneck event, and not the number/tempo/strength of multiple serial bottleneck events. I do like that the authors don’t overplay this in the later results (e.g. testing with a model of two epochs).

Important - comparison of methods perhaps? This would be important for benchmarking - if computational constraints make this intractable (e.g. size of datasets, availability of reliable phase information, linkage maps), perhaps at least comparison of estimated demographic parameters such as the founder size/age would be useful for users.
Resolved - these are very well detailed in the Supplementary material.

We thank the reviewer for carefully reading the manuscript and supplementary material and we are grateful for the positive comments.

In step 2, the assumption here is that individuals that share IBS are IBD? However, IBS could be very well due to population structure? How does ASCEND account for this?
--Maybe addressed by removing closely related individuals in their human analyses? But how do the authors propose that this is achieved for datasets without pedigree information?

We do not use pedigree information and we are therefore measuring IBS. We assume that genome-wide signatures of IBS (beyond a certain threshold) are mainly due to common descent (IBD). To reduce the impact of ancient population structure, we account for ancestral allele sharing by calculating the cross-population allele sharing correlation using an outgroup population or a set of random individuals (not including the target group). Further, to distinguish between consanguinity and IBD, we exclude individuals with excess relatedness which could be indicative of recent familial relationships.

They state that they compute a cross population correlation to account for ancestral allele sharing - but this also is confounded if there’s recent migration - perhaps addressed by the second model of recent gene flow tested (Fig. S2.4.2)? I see that this model is one of admixture prior to the bottleneck event, and not after the bottleneck event; post admixture decay of IBD segments will be accelerated during a bottleneck and therefore would not be expected to affect the number of shared alleles. However, if the admixture event occurs post a bottleneck, as it does with most human populations (e.g. recent admixture in Europeans, or recent admixture in North Africans), IBS is definitely != IBD. I think that this study would benefit from one more simulation study, looking at models of recent admixture (post bottleneck), and assess performance of ASCEND on such data.

We thank the reviewer for highlighting this important point. We had also considered this scenario, however, the interpretation or implementation here is not straightforward. For instance, when the admixture event occurs after a bottleneck, it implies that the founder event occurred in one of the ancestors of the target population. Without prior knowledge of the history or deconvolution of local ancestry in the target group, this is challenging and in particular difficult to interpret when only data from the target is used. For instance, we would use data from African Americans and attempt to recover the signature of the out-of-Africa bottleneck or other founder events in African and European populations.

To demonstrate this by simulations, we generated data for three populations, two source populations (Pop S1 and Pop S2*)* and one target population (Pop 1) that derives ancestry from both sources. In all simulations, the admixture occurred 20 generations ago and the founder events pre-dated the admixture, occurring in either or both of the source populations. As expected, we found the intensity was underestimated since admixture increases diversity. Further, we found the inferred founder age could be biased depending on the number, the source population experiencing the founder event(s) and the proportion of admixture. For scenarios of very limited admixture, we were able to recover the founder age. However, for a higher proportion of admixture, the timing of the founder event(s) was underestimated or similar to the time of admixture. More importantly, it was hard to interpret the timing as it was unclear if it was related to the founder event of population S1 or S2 or Pop 1. We have added the simulation results and some description in Supplementary Note S2.4.2. We note, however, that the results reported in our manuscript are robust to this issue as we observe minimal correlation in the estimated time of admixture and founder age in empirical data.

Further, we added this following paragraph to the discussion:

“Finally, we note that complex demographic scenarios in which admixture events postdate founder events are hard to interpret, as the founder event could have occurred in the target or one of the ancestral populations. In this scenario, the target population has a mosaic genome with chromosomal segments from multiple ancestral groups and the signatures of founder event may be present only in a subset of their genomic regions (if the founder event occurred in one of the ancestral populations). We explored this scenario by generating simulated data where the founder event(s) occurred in one or both ancestral populations of an admixed target population (Notes S2.4.2). As expected, we found the intensity was lower than at the start of the bottleneck as admixture increases diversity. The inferred founder age could be biased depending on the number, the source or ancestral group experiencing the founder event, and the proportion of admixture. For scenarios with very low admixture, we were able to recover the founder age accurately. However, for higher proportions of admixture, the founder age was underestimated or similar to the time of admixture (Figure S2.4.2.2). We note that in practice this bias should not impact the empirical results reported above as we observed minimal correlation between the inferred time of admixture (*GLOBETROTTER* or *ALDER*) and the founder ages (Table S3). However, when there is evidence of recent admixture in the target population which postdates the founder event, it is advisable to perform local ancestry inference in the admixed population and then apply *ASCEND* to genomic regions that are confidently assigned to each ancestral population separately. This will lead to less ambiguity about the source of the founder event (target or one of the ancestral groups) and provide more reliable results.”

I also found the GitHub page for ASCEND to be very detailed. I do think that users will benefit from a vignette/tutorial of some sort though, perhaps even using their example dataset.

We thank the reviewer for positive comments. We have added some tutorials in the “docs/” directory on *ASCEND* GitHub to guide the users.

# Reviewer #3

Tournebize et al present ASCEND - a new method to infer the timing and strength of founder (e.g. bottleneck) events from genotype data. ASCEND computes the correlation in pairwise allele sharing between pairs of sites separated by varying genetic distances and relates this to founder events. It fits a exponential decay of this correlation with distance, to me this is broadly similar to the ALDER/ROLLOFF methods to infer admixture histories, but with different statistics and interpretation. Briefly, the correlation at the smallest genetic distances considered reflects the intensity of the founder event, and the rate of decay in correlation with genetic distance reflects the timing of the founder event.

The manuscript contains quite a number of simulations and analyses, with 6 different simulation scenarios and 184 human populations and 40 dog breeds going . The results are presented in detail in the supplement, and summarized in the main text. Broadly, I found these analyses were conducted with care and an understanding of the many potential pitfalls that can arise from the analysis of large-scale genetic data.

Based on the simulated data, the authors report the ability to infer the timing and intensity of founder events for events with 200 generations, and show the method is decently robust to a range of demographic scenarios containing a founder event.

By applying the method to real data sets from humans and dogs, the authors present evidence for founder events in the history of many populations, with results largely consistent with prior studies. However, one of the main advantages of ASCEND vs competing methods is the reduced data requirements. ASCEND requires many fewer samples, doesn’t require phasing, and can be applied to data sets with missing data or haploid samples - the authors do recommend access to genetic data from an outgroup population not subject to the founder event though - which may be difficult in some cases. For comparison, competing methods (e.g. IBDNe) require access to hundreds of samples and require phased data. However one possible catch is that the authors suggest caution when interpreting results derived from what seem like somewhat common scenarios, e.g. exponential growth following the founder event.

I was able to download ASCEND from the github repo and run an analysis on the example data set. Everything seemed to run fine, after a small bit of debugging due to error when running on compressed data files.

I would encourage the authors to provide version information for the required packages, as I received a number of depreciation warnings when running the code.

We are grateful to the reviewer for downloading and testing the code. We have updated the documentation on GitHub and clarified the data formats needed for the method. We currently support EIGENSTRAT format only (<https://reich.hms.harvard.edu/software/InputFileFormats>). In future releases, we will try to add the functionality for other formats (including compressed formats).

Large issues: Broadly, I feel that authors did a very good job of trying to consider how different demographic scenarios in combination with a founder event could affect the analysis, with the authors testing 6 total scenarios. However, I think there is an important aspect missing here - consideration of false positives. One of the main claims of this manuscript is the ability to detect (and characterize) founder events. The authors go into some detail evaluating how well existing founder events are characterized, but never evaluate the application of ASCEND to data from populations lacking founder events. This is important because researchers want to be relatively sure that when ASCEND reports a founder event, there was one. This could be done by applying ASCEND to simple demographic models with constant / growing populations without founder events, and or maybe something like the 3-population human out of africa model. This more complicated model does have founder events and population growth, but the founder events seem to be older than the focus of ASCEND.

We thank the reviewer for this suggestion. We included a new simulation with a three-population model which includes West Africans (with constant population size) and two non-African populations, namely East Asians and Northern Europeans that have a history of out-of-Africa migration followed by recent population expansions. None of the populations have a recent founder event. Reassuringly, we did not detect any significant founder events in either population (West Africans, East Asians or Northern Europeans). This suggests that ASCEND has a low false discovery rate and gives reliable results in the absence of founder events. We added the following to the manuscript to describe these results:

“We note that in addition to its high sensitivity to detect recent founder events, *ASCEND* also has a low false discovery rate and gives reliable results in the absence of founder events. We simulated a three-population demographic model to represent three modern human populations, including a West African population with constant population size and two non-African groups––Northern Europeans and East Asians––with a history of out-of-Africa bottleneck and recent expansion but no recent founder events (in the past 200 generations). We found that *ASCEND* accurately failed to detect a significant founder event in all three populations using the criteria described in Methods (Notes S2.8).”

Small issues:

Overall, I found the writing clear, but I suggest the notation should be adjusted to improve clarity. These are not large changes, but can aid in reader comprehension
S_d vs S(d) on line 615

We changed all occurrences of S(d) to S_d in the main text and supplementary notes.

d in units of Morgans (line 612) or cM (line 718)

Thank you for pointing out this inconsistency. We changed all occurrences to Morgans for consistency, including in supplementary notes (with appropriate rescaling where necessary). We clarified this information in the text.

“Note that if the genetic distances are given in cM: $\hat{T_{f}}=100\cdot t$”

i indexing “SNPs” (line 702) or individual (line 609)

We thank the reviewer for pointing out this ambiguity. We have fixed the indexing in main text and supplementary notes.

Use of the all the terms “marker” “SNP” “locus”, “variant site” within the methods- do these all refer to the same thing or are there meaningful differences?

We have fixed this issue and, in all places, we refer to variants as “SNPs”.

Line 27: In these few sentences both the terms “human populations” and “surveyed groups” are used, but the distinction is not fully clear.

We replaced “surveyed groups” by “analyzed populations” in this sentence and elsewhere in the manuscript.

Line 52 - It’s not clear to me what “diverse” means here - genetically diverse or something else?

We removed the use of this adjective from most places. When used, we qualify what diverse means––diverse sampling ages, diverse ethno-linguistic groups, etc.

Line 84 - clarify that this is given in generations, not years.

We believe this is a general statement and is applicable both to founder age defined in years or generations. However, we include the units in the *Results* under “*Overview of ASCEND*”.

Line 150 - I am a bit confused about the units for intensity of a founder event. It is often shown in this paper as a percent, but a percent of what? (generations per ind?). For clarity, I suggest presenting it as a decimal, as it appears in your citation #9.

We chose to report the intensity values as percentages for easier readability, since the numbers are small, usually on the order of the hundredth decimal (i.e., 2.1% vs. 0.021). We clarified this in the text at the first presentation of the *I_f_* values.

Line 553 - missing “of”

Done.

Line 568 - ROH is not defined prior to this.

Done.

Line 672 - Make it more clear you assume no recombination during the bottleneck.

We clarified this in the methods.

Line 683 - I was a bit confused by this sentence, also is there a citation for the relation between z and expected time to coalescence?

This is inspired by Nordborg (2000) which derived that the expected time of coalescence for a pair of lineages is 2No under a simple demographic model. We have now added the reference.

Figure 1
Figure 1. I found the resolution a bit lacking, otherwise a nice figure. In panel C, I noticed that the true founder age was not evaluated on the same range of values as in panel B, but it was not clear why.

We replaced Figure 1A by the previous Figure 5. We thank the reviewer for pointing out the range discrepancy between 1B and 1C. We thus removed the panel *T_f_*=70 in Fig C and added a panel for *T_f_*=300, so that we now have the same range in simulated founder ages between 1B and 1C. Also, we made very minor visual changes to Figures 1B and 1C to increase the resolution.

Figure 2. I think the current figure is a nice compromise between visual appeal and data presentation, but it could be improved by the reduction of overplotting, perhaps by adjusting the spacing of the data points.

Thank you for the suggestion. We instead shifted the location of a few populations and connected them to their true location using a shaded arrow. We hope that this modification makes the representation clearer.

Figure 3 - The violin plot outlines add color, but it is unclear what the relative widths of the violins represent within and between plots - do they all contain the same area?

Violin plots are indeed scaled by area, so all violins have the same area within each panel. We clarified this point in the figure legend.

Figure 4
Denoting the breeds / roles solely via pictograms might be difficult for readers not familiar with dogs. The images are great, but I suggest they are accompanied by text labels. Also, I might suggest that the results be presented in a more genetic / evolutionary context by organizing the dog breeds via an evolutionary tree, rather than in a circle plot organized by founder intensity

We included the definition of each group next to the images. Also we specified that ages are given in generations before present. Regarding the ordering of the breeds, we preferred to keep grouping breeds by AKC types and by increasing order of founder intensity within, as we make a point about the distribution of intensity in results.

Figure 5 - Great figure, I used it often to help interpret the results and text. I might
suggest putting this figure first and removing the current Figure 1A.

We thank the reviewer for this suggestion, and we have replaced Figure 1A with Figure 5. We also find that the two figures were somewhat redundant.
